# The *Capparis spinosa* var. *herbacea* genome provides the first genomic instrument for a diversity and evolution study of the Capparaceae family

**DOI:** 10.1093/gigascience/giac106

**Published:** 2022-10-30

**Authors:** Lei Wang, Liqiang Fan, Zhenyong Zhao, Zhibin Zhang, Li Jiang, Mao Chai, Changyan Tian

**Affiliations:** State Key Laboratory of Desert and Oasis Ecology, Xinjiang Institute of Ecology and Geography, Chinese Academy of Sciences, Urumqi 830011, China; University of Chinese Academy of Sciences, Beijing 100049, China; Institute of Cotton Research of the Chinese Academy of Agricultural Sciences, Anyang, Henan 455000, China; Zhengzhou Research Base, State Key Laboratory of Cotton Biology, Zhengzhou University, Zhengzhou 450000, China; State Key Laboratory of Desert and Oasis Ecology, Xinjiang Institute of Ecology and Geography, Chinese Academy of Sciences, Urumqi 830011, China; University of Chinese Academy of Sciences, Beijing 100049, China; Institute of Cotton Research of the Chinese Academy of Agricultural Sciences, Anyang, Henan 455000, China; Zhengzhou Research Base, State Key Laboratory of Cotton Biology, Zhengzhou University, Zhengzhou 450000, China; State Key Laboratory of Desert and Oasis Ecology, Xinjiang Institute of Ecology and Geography, Chinese Academy of Sciences, Urumqi 830011, China; University of Chinese Academy of Sciences, Beijing 100049, China; Institute of Cotton Research of the Chinese Academy of Agricultural Sciences, Anyang, Henan 455000, China; Zhengzhou Research Base, State Key Laboratory of Cotton Biology, Zhengzhou University, Zhengzhou 450000, China; State Key Laboratory of Desert and Oasis Ecology, Xinjiang Institute of Ecology and Geography, Chinese Academy of Sciences, Urumqi 830011, China; University of Chinese Academy of Sciences, Beijing 100049, China

**Keywords:** *Capparis spinosa* var. *herbacea*, genome assembly, population evolution

## Abstract

**Background:**

The caper bush *Capparis spinosa* L., one of the most economically important species of Capparaceae, is a xerophytic shrub that is well adapted to drought and harsh environments. However, genetic studies on this species are limited because of the lack of its reference genome.

**Findings:**

We sequenced and assembled the *Capparis spinosa* var. *herbacea* (Willd.) genome using data obtained from the combination of PacBio circular consensus sequencing and high-throughput chromosome conformation capture. The final genome assembly was approximately 274.53 Mb (contig N50 length of 9.36 Mb, scaffold N50 of 15.15 Mb), 99.23% of which was assigned to 21 chromosomes. In the whole-genome sequence, tandem repeats accounted for 19.28%, and transposable element sequences accounted for 43.98%. The proportion of tandem repeats in the *C. spinosa* var. *herbacea* genome was much higher than the average of 8.55% in plant genomes. A total of 21,577 protein-coding genes were predicted, with 98.82% being functionally annotated. The result of species divergence times showed that *C. spinosa* var. *herbacea* and *Tarenaya hassleriana* separated from a common ancestor 43.31 million years ago.

**Conclusions:**

This study reported a high-quality reference genome assembly and genome features for the Capparaceae family. The assembled *C. spinosa* var. *herbacea* genome might provide a system for studying the diversity, speciation, and evolution of this family and serve as an important resource for understanding the mechanism of drought and high-temperature resistance.

## Background

The caper bush *Capparis spinosa* (NCBI:txid2717819), one of the most economically important species of Capparaceae, is a perennial winter deciduous shrub with a wide range, typically growing in the Mediterranean countries and distributed in Iran, Iraq, Saudi Arabia, and China [1–3]. In China, it is mainly found in Xinjiang, Gansu, and Tibet regions [4]. The *C. spinosa* family Capparaceae from the Mediterranean to Central Asia has been taxonomically revised recently [5]. *C. spinosa* is considered a single species, represented by 4 subspecies—*C. spinosa* subsp. *spinosa*, *C. spinosa* subsp. *rupestris*, *C. spinosa* subsp. *cordifolia*, and *C. spinosa* subsp. *himalayensis*. *C. spinosa* subsp. *spinosa* is widely distributed from the east Mediterranean to China and Nepal and possesses a high degree of heterogeneity in different genetic traits. Within *C. spinosa* subsp. *spinosa* subspecies, some varieties are identified—namely, *C. spinosa* var. *herbacea* and *C. spinosa* var. *atlantica* [6]. *C. spinosa* var. *herbacea* is mainly distributed in Turkey, Kazakhstan, Uzbekistan, Afghanistan, and western China [7]. According to the morphological characteristics of China reported by previous researchers [5], *C. spinosa* var. *herbacea* is mainly distributed in Xinjiang, Tibet, and Gansu in China.

**Figure 1:**
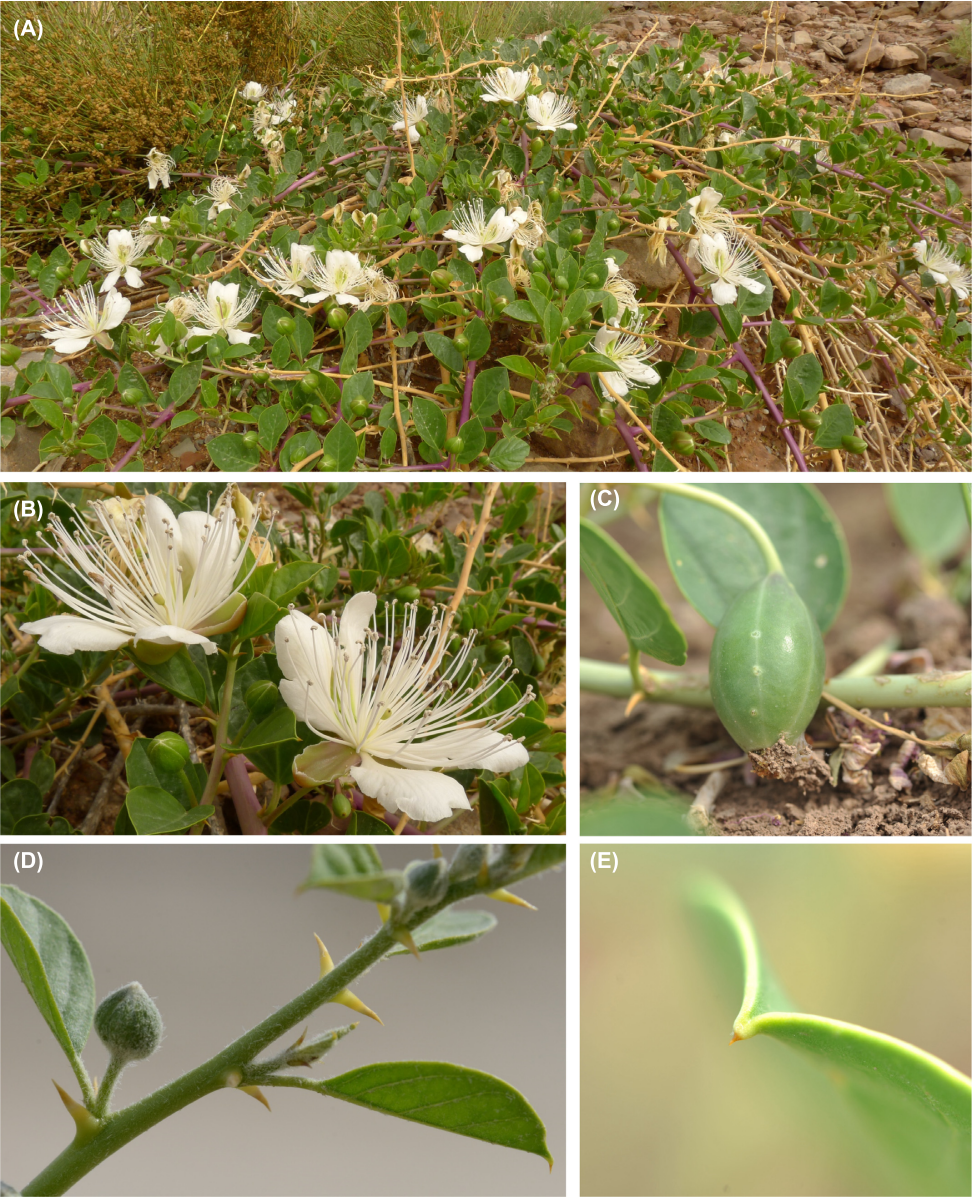
Growth of *Capparis spinosa* var. *herbacea* in wild collection sites. (A) Mature *C. spinosa* var. *herbacea* plant. (B) Flowers. (C) Fruits. (D) Stem. (E) Leaf tip.

As a drought-tolerant crop, *C. spinosa* has an extensive root system and a remarkably high root-to-shoot ratio and thus has a strong ability to find and absorb water from the environment (especially deep in the soil), resulting in significant adaptation to harsh environments [8, 9] (Fig. 1). Besides the roots, other parts of *C. spinosa*, including leaves, buds, fruits, bark, and seeds, contain a variety of bioactive compounds, such as flavonoids, phenolics, alkaloids, glucosinolates, and vitamins that have long been used in the treatment of headaches, toothaches, and kidney disease, and they play a role in preventing disease and reducing the risk of carcinogenesis [10–16]. For example, methanolic extracts prepared from the fruits and flower buds of *C. spinosa* have some anti-inflammatory and antithrombotic effects [17]. *C. spinosa* has a huge agricultural potential because of its medicinal properties and its ability to grow under drought conditions. Thus far, only a few chloroplast genomes [7, 18–20], mitochondrial genomes [21], and Simple Sequence Repeats (SSR) sequences [22] of *Capparis* have been reported, and the lack of genomic information hinders the genetic improvement and effective use of caper plants.

Here, we report a high-quality whole-genome sequence of *C. spinosa* var. *herbacea* using PacBio HiFi sequencing and high-throughput chromosome conformation capture (Hi-C) technology. Detailed information on the *C. spinosa* var. *herbacea* genome can help elucidate the biogeography and evolution of *Capparis* plants, contribute to the understanding of the molecular basis of its resistance to stress, and validate its medicinal uses.

## Analysis

### Genome size estimation

We used a single plant of *Capparis spinosa* var. *herbacea* that was collected from the Xinjiang Institute of Ecology and Geography Chinese Academy of Sciences for whole-genome sequencing. A total of 33.08 Gb genomic short-read data were obtained for the genome survey (Table 1). We generated the 17-mer distribution of sequencing reads from short libraries using the *k*-mer method. The estimated genome size was about 245.97 Mb, and the proportion of repeat sequences and the genome heterozygosity rate were determined to be approximately 49.5% and 0.878%, respectively (Supplementary Fig. S1A). The flow cytometry [23] analysis result was 279.07 Mb (Supplementary Fig. S1B).

**Table 1:** Sequencing data used for *Capparis spinosa* var. *herbacea* genome assembly and annotation

Sequencing type	Application	Sequencing platform	Bases (Gb)	Reads
Genome short reads	Genome survey and assessment	Illumina NovaSeq 6000	33.08	221,078,842
Genome long reads	Contig assembly	PacBio Sequel II	25.46	1,531,982
Hi-C reads	Chromosome construction	Illumina NovaSeq 6000	30.64	204,744,634
Transcriptome long reads	Genome annotation	PacBio Sequel II	1.52	413,148
Transcriptome short reads	Genome annotation	Illumina NovaSeq 6000	11.31	75,789,484

### Genome sequencing and assembly

**Figure 2:**
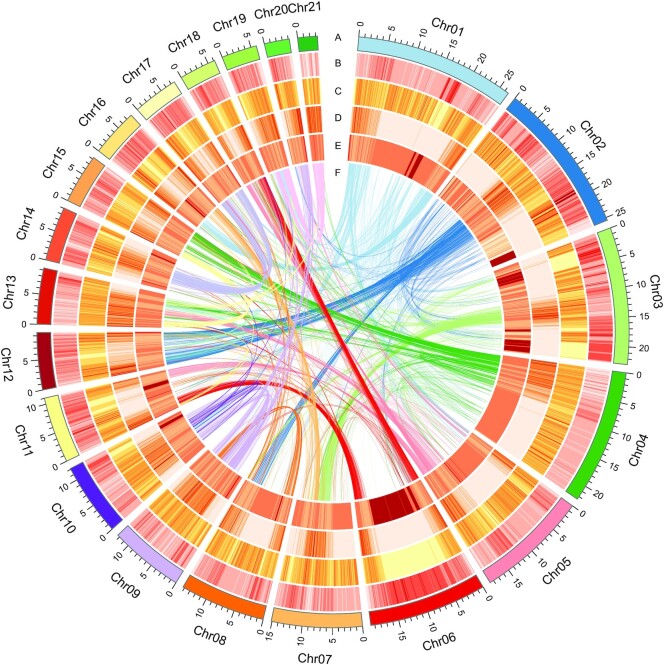
High-quality assembly of 21 chromosomes. (A) Chromosome ideograms. (B) Transposable element (TE) repeat sequence density (window size 100 kb). (C) Tandem repeat sequence density (100-kb window size). (D) Gene density (100-kb window size). (E) GC content (100-kb window size). (F) Relationship between syntenic blocks.

In this study, PacBio circular consensus sequencing (CCS) long reads and Hi-C reads were used for *C. spinosa* var. *herbacea* genome sequencing and assembly. A total of 25.46 Gb PacBio clean long reads with an average read length of 16,618 bp were generated for genome assembly, and 30.64 Gb Hi-C data were generated for auxiliary genome assembly (Table 1, Supplementary Table S1). The primary contigs were assembled with PacBio CCS reads, and a 274.53-Mb genome assembly version was generated with contig N50 of 11.04 Mb (Supplementary Table S1). Hi-C reads were used to generate chromosome-level assembly of the genome (Fig. 2, Supplementary Fig. S2). The final genome assembly of *C. spinosa* var. *herbacea* was 274.53 Mb, consisting of 59 contigs and 29 scaffolds. The contig N50 was 9.36 Mb and the longest contig was 22.51 Mb, while the scaffold N50 was 15.15 Mb and the longest scaffold was 26.66 Mb (Table 2).

**Table 2:** Assembly statistics of the *Capparis spinosa* var. *herbacea* genome

Category	Numbers	N50 (Mb)	Longest (Mb)	Size (Mb)	Percentage of assembly
Contigs	59	9.36	22.51	274.53	100
Scaffold	29	15.15	26.66	274.53	100
Anchored	28	15.15	26.66	274.49	99.98
Anchored and oriented	21	15.15	26.66	272.43	99.23
Gene annotated	21 577	NA	NA	64.26	23.42
Repeat sequence	NA	NA	NA	173.60	63.23

For genome quality assessment, BUSCO analysis of the final scaffold assembly showed that 96.80% complete BUSCO genes (92.80% complete and single-copy BUSCO genes and 4.00% complete and duplicated BUSCO genes) were identified (Supplementary Table S2). Merqury revealed a consensus quality value (QV) of 28.27 and assembly accuracy of 99.85%. CEGMA was used to evaluate the completeness of the final genome assembly, and 98.03% of the CEGMA genes were present in the genome. A total of 98.52% short sequences were successfully aligned to the genome. The genome LTR Assembly Index (LAI) value was 17.19 of the genome assembly. A LAI value greater than 10 and less than 20 indicates that the assembly quality has reached the reference genome level [24]. Thus, these results demonstrate the high quality and completeness of the *C. spinosa* var. *herbacea* genome assembly.

### Identification of genomic repetitive sequences

Moreover, 120,748,115 bp (nearly half of the assembled genome length [43.98%]) of transposable element (TE) repetitive sequences in the genome assembly of *C. spinosa* var. *herbacea* were identified by both homology-based and *de novo* methods (Supplementary Table S3). Retroelement elements constituted the predominant repeat type, accounting for 31.24% of the genome length. The long terminal repeat (LTR) superfamily elements Copia and DNA TEs constituted 29,749,806 and 34,990,312 bp, corresponding to 10.84% and 12.75% of the genome length, respectively. LTR superfamily elements Gypsy and CACTA constituted 11,447,091 and 7,034,814 bp, accounting for 4.17% and 2.56% of the genome length, respectively. The density of Copia elements decreased with the increasing density of genes, whereas the DNA TEs were distributed more evenly across the genome and showed no obvious patterns or relationships with the distribution of genes (Fig. 2).

The total length of the identified tandem repeats (TRs) was 52,920,691 bp, accounting for 19.28% of the total length of the genome. The total length of microsatellites (1–9 bp units) was 43,481,890 bp (15.84%), the total length of minisatellites (10–99 bp units) was 7,039,326 bp (2.56%), and the total length of satellites (≥100 bp units) was 2,399,475 bp (0.87%).

On analyzing the genome distribution features, we found a correlation between the distribution of TR sequences and GC content of the chromosomes of the *C. spinosa* var. *herbacea* genome (Fig. 2C, E; Supplementary Fig. S3A). Spearman rank correlation was used to determine the correlation, and the correlation coefficient was −0.52 and the *P*value was 2.2e-16 (Supplementary Fig. S3B), showing a negative correlation between the distribution of TR sequences in the *C. spinosa* var. *herbacea* genome and the GC content of the sequences.

### Genome coding gene prediction and annotation

A total of 11.31 Gb transcriptome short reads and 1.52 Gb transcriptome long reads were used for gene prediction (Table 1). Combining the results by the 3 methods, 21,577 protein-coding genes were predicted (Table 2, Supplementary Table S4). Over 98.82% of the protein-coding genes were annotated for gene function using the following databases: GO (84.53%), KEGG (76.57%), KOG (59.33%), TrEMBL (98.63%), Pfam (87.75%), Swiss-Prot (84.76%), eggNOG (87.90%), and Nr (98.69%) (Supplementary Table S5), indicating that gene predictions were accurate.

### Dynamic changes of duplicated genes

**Figure 3:**
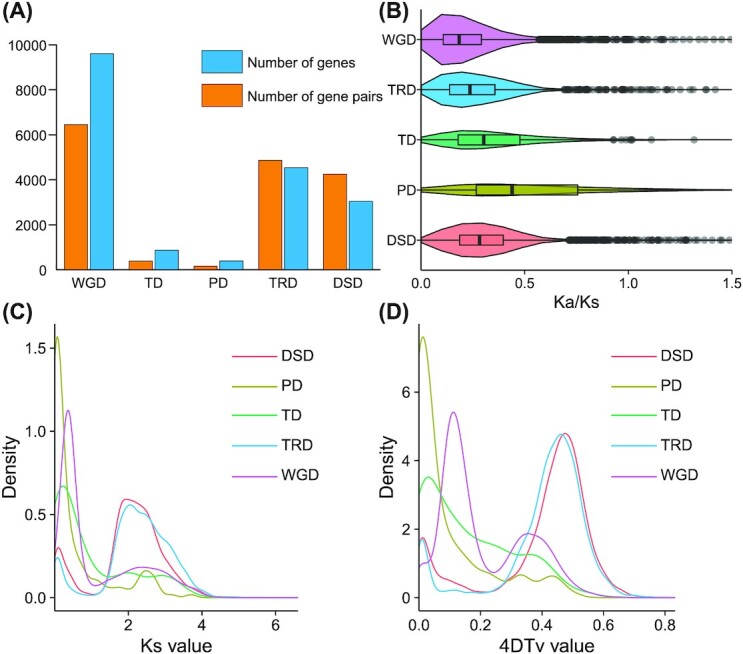
Gene duplication and evolution of *Capparis spinosa* var. *herbacea*. (A) Number of genes and gene pairs of 5 duplication types. (B) Distribution of Ka/Ks of 5 duplication types. (C) Distribution of Ks of 5 duplication types. (D) Distribution of 4DTv of 5 duplication types.

Duplicated genes were classified into 5 categories: whole-genome duplication (WGD), tandem duplication (TD), proximal duplication (PD), transposed duplication (TRD), and dispersed duplication (DSD) (Fig. 3A, Supplementary Table S6). Of the 21,577 genes, 18,432 were identified as duplicated genes, including 9,603 derived from WGD (52.1%), 872 from TD (4.7%), 387 from PD (2.1%), 4,534 from TRD (24.6%), and 3,036 from DSD (16.5%). Ka (number of nonsynonymous substitutions per nonsynonymous site), Ks (number of synonymous substitutions per synonymous site), 4DTv (4-fold degenerate synonymous site), and the Ka/Ks ratio were calculated for the different duplication types. Among the 5 duplication types, the proportion of gene pairs with Ka/Ks >1 in *Arabidopsis thaliana* was PD (5.1%), TD (3.3%), DSD (0.6%), TRD (0.3%), and WGD (0.0%). However, the corresponding ratios in *C. spinosa* var. *herbacea* were PD (13.7%), TD (4.9%), DSD (1.3%), TRD (0.9%), and WGD (1%). PD and TD genes had qualitatively higher Ka/Ks ratios than genes derived from the other duplication types (Fig. 3B). PD with Ka/Ks >1 in *C. spinosa* (13.7%) was significantly higher than that of *A. thaliana* (5.1%). The density distribution of Ks and 4DTv showed that all 5 duplication types of *C. spinosa* var. *herbacea* experienced two duplications (Fig. 3C, D). However, the 5 duplication types had different times when duplication occurred. PD experienced a duplication 3.89 million years ago (MYA) (Ks peak at 0.069, 4DTv peak at 0.013). This also explains the high proportion of positive selection in PD.

GO and KEGG enrichment analysis was performed on the Ka/Ks >1 genes in the 5 duplication types. In GO enrichment analysis, all 5 duplication types exhibited divergent functions. TRD was not enriched to a significant GO term. WGD and DSD were mainly enriched in the GO terms of plastid stroma, chloroplast stroma, obsolete chloroplast part, organellar small ribosomal subunit, and organellar ribosome. PD and TD shared more enriched GO terms related to pyrroline-5-carboxylate reductase activity, L-proline biosynthetic process, ribosomal RNA (rRNA) processing, protein disulfide oxidoreductase activity, peroxisome, cysteine-type peptidase activity, terpene synthase activity, magnesium ion binding, defense response to fungus, rRNA binding, response to wounding, and small ribosomal subunit compared with the other duplication types. KEGG enrichment analysis of PD and TD showed that these genes were mainly enriched in heat shock 70-kDa protein 1/2/6/8, molecular chaperone HtpG, (−)-germacrene D synthase, and KUP system potassium uptake protein, suggesting that the PD and TD genes in *C. spinosa* var. *herbacea* play important roles in environmental stress tolerance (Supplementary Fig. S4).

### Analyses of genome synteny and WGD

To analyze the evolution of the *C. spinosa* var. *herbacea* genome, dot plots of longer syntenic blocks within the *C. spinosa* var. *herbacea* genome were completed. *C. spinosa* var. *herbacea* undergoing WGD was clearly seen at Chr19 and Chr21 (Supplementary Fig. S5A). Moreover, the syntenic blocks and collinear gene pairs between *C. spinosa* var. *herbacea* and *Amborella trichopoda*, *C. spinosa* var. *herbacea* and *A. thaliana*, *C. spinosa* var. *herbacea* and *Theobroma cacao*, *C. spinosa* var. *herbacea* and *Vitis vinifera*, *C. spinosa* var. *herbacea* and *Solanum lycopersicum*, *T. hassleriana* and *C. spinosa* var. *herbacea*, and *Gynandropsis gynandra* and *C. spinosa* var. *herbacea* were implemented, respectively (Supplementary Fig. S5). The syntenic analysis results also showed more collinear gene pairs between *C. spinosa* var. *herbacea* vs. *A. thaliana* and *C. spinosa* var. *herbacea* vs. *T. hassleriana* (Supplementary Table S7), indicating that *C. spinosa* var. *herbacea* has a close evolutionary relationship with *A. thaliana* and *T. hassleriana*. At the same time, it can be seen from the stacking diagram of collinear genes on chromosomes that *C. spinosa* var. *herbacea* underwent WGD alone after divergence from *A. thaliana* (Supplementary Fig. S5D).

**Figure 4:**
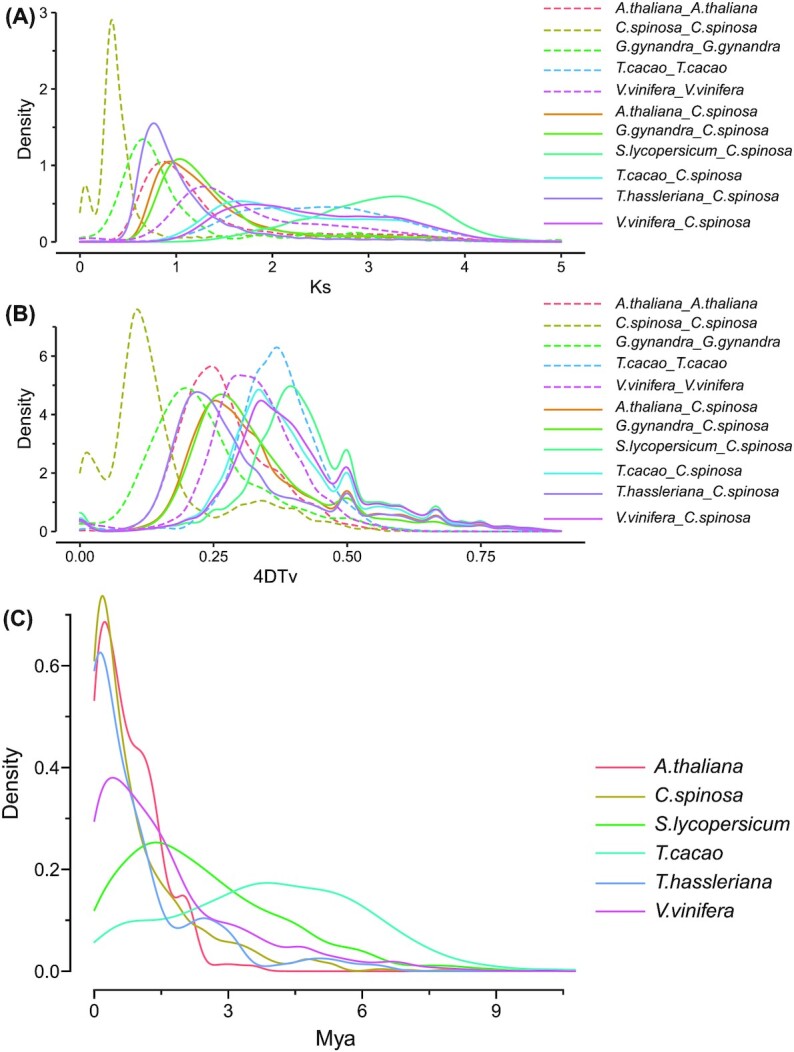
Distribution of Ks, 4DTv, and ages of LTR of *Capparis spinosa* var. *herbacea* and other species. (A) Ks distribution of *C. spinosa* var. *herbacea* and other representative species. (B) 4DTv distribution of *C. spinosa* var. *herbacea* and other representative species. (C) Ages of LTR of *C. spinosa* var. *herbacea* and other species (molecular clock r is 7*10^−9^).

Using the homologous gene pairs identified above, the 4DTv and Ks values were calculated for *C. spinosa* var. *herbacea*, *V. vinifera*, *S. lycopersicum*, *A. thaliana*, *T. hassleriana*, *Gynandropsis gynandra*, and *T. cacao*. The results showed that *C. spinosa* var. *herbacea* separated from *T. cacao*, *G. gynandra*, *A. thaliana*, and *T. hassleriana* in order at 93.10 MYA (Ks peak of 1.644 and 4DTv peak of 0.336), 58.46 MYA (Ks peak of 1.032 and 4DTv peak of 0.266), 53.00 MYA (Ks peak of 0.936 and 4DTv peak of 0.254), and 43.31 MYA (Ks peak of 0.765 and 4DTv peak of 0.220). Besides, *C. spinosa* var. *herbacea* experienced an α WGD event at 18.59 MYA (Ks peak at 0.328) (Fig. 4 A, B).

**Figure 5:**
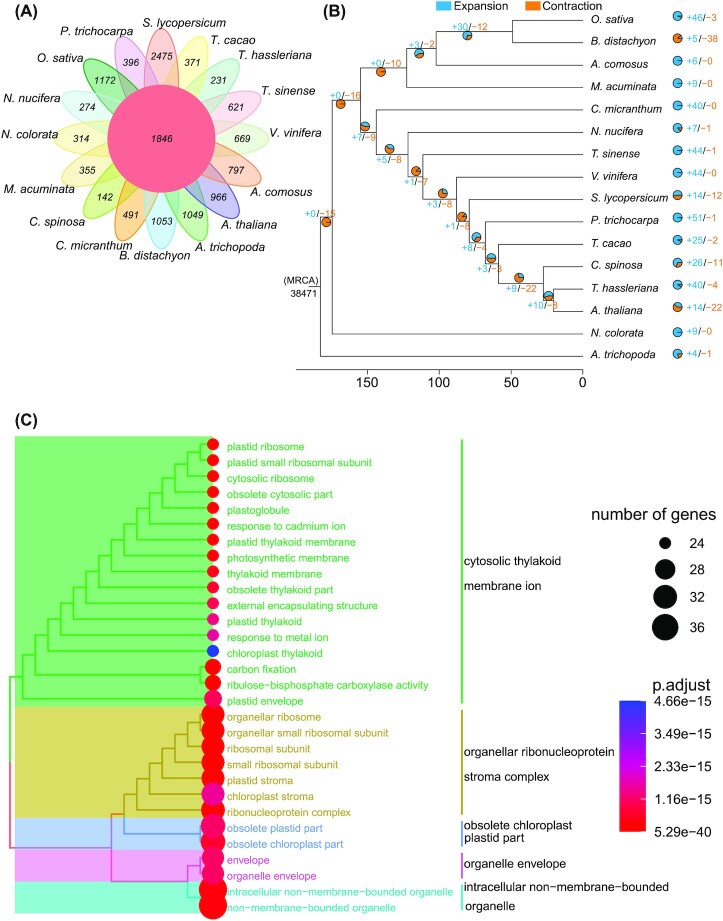
Evolution of the *Capparis spinosa* var. *herbacea* genome. (A) Venn diagram of specific and shared orthologs among 16 species (*O. sativa*, *B. distachyon*, *A. comosus*, *M. acuminata*, *C. micranthum*, *N. nucifera*, *T. sinense*, *V. vinifera*, *S. lycopersicum*, *P. trichocarpa*, *T. cacao*, *C. spinosa* var. *herbacea*, *T. hassleriana*, *A. thaliana*, *N. colorata*, and *A. trichopoda*) identified based on gene family cluster analysis. Each number in the diagram represents the number of gene families within a group. (B) Expansion and contraction of gene families. (C) GO enrichment analysis of genes from expanded families.

We compared the LTR insertion time of *A. thaliana*, *C. spinosa* var. *herbacea*, *S. lycopersicum*, *T. cacao*, *Tarenaya hassleriana*, and *V. vinifera* (Fig. 4C). The results indicated that LTR bursts the time peak of *C. spinosa* var. *herbacea* (peak at 0.178 MYA) between *A. thaliana* (peak at 0.236 MYA) and *T. hassleriana* (peak at 0.132 MYA), which was also consistent with the phylogenetic tree (Fig. 5B).

### Gene family expansion and contraction

Protein sequences of 15 species (*Oryza sativa*, *Brachypodium distachyon*, *Ananas comosus*, *Musa acuminata*, *Cinnamomum micranthum*, *Nelumbo nucifera*, *Tetracentron sinense*, *V. vinifera*, *S. lycopersicum*, *A. trichopoda*, *Nymphaea colorata*, *T. hassleriana*, *A. thaliana*, *T. cacao*, *Populus trichocarpa*), together with C. spinosa var. herbacea, were downloaded for gene family expansion and contraction analysis. As a result, all protein-coding genes were clustered into 49,850 orthogroups based on sequence homology. A total of 1,846 gene families were shared by all 16 species, and 142 *C. spinosa* var. *herbacea* specific gene families were found (Fig. 5A). Moreover, the KEGG enrichment analysis revealed that species-specific genes were enriched in DNA kinase ATPase repair, MFS transporter, peroxin 3, disease resistance protein RPM1, and zinc finger SWIM domain−containing protein 3 (Supplementary Fig. S6A).

Based on the 306 orthogroups of single-copy genes, the phylogenetic tree was constructed and the MCMCTree program in PAML was used to estimate divergence times. The phylogenetic tree identified the closest relationship of *C. spinosa* var. *herbacea* to *T. hassleriana*. Based on the time tree, the number of gene families that experienced expansion or contraction was estimated by computational analysis of gene family evolution (CAFE). The results showed that in almost species, except *B. distachyon* and *A. thaliana*, more gene families experienced expansion rather than contraction. In *C. spinosa* var. *herbacea*, 26 gene families experienced expansion, while 11 gene families underwent contraction (Fig. 5B). GO enrichment analysis of the expanded gene families of *C. spinosa* var. *herbacea* showed that these genes were mainly enriched in chloroplast thylakoid, chloroplast envelope, thylakoid, chloroplast thylakoid membrane, response to abscisic acid, response to the hormone, and so forth (Fig. 5C). Moreover, based on KEGG enrichment analysis, the genes related to photosynthesis, chloroplast thylakoid membrane, and response to abscisic acid of hormone-related pathways were enriched (Supplementary Fig. S6B). The function for these gene families expanded in *C. spinosa* var. *herbacea*, indicating that the expansion of the hormone response pathway and the photosynthesis pathway might have helped *C. spinosa* var. *herbacea* to generate more energy to adapt to arid environments.

## Discussion

It is well known that *Capparis spinosa* has many subspecies and varieties [7, 25], and the identification of samples is often controversial. According to the taxonomic characteristics of *C. spinosa* var. *herbacea*, the branchlets are usually white-tomentose in the upper part and the stipules are straight, horizontal or slightly curved, and yellowish [5]. The samples used for genome sequencing in this study matched the above taxonomic characteristics (Fig. 1D). Besides, the location where the samples were collected in this study is consistent with the geographical distribution of *C. spinosa* var. *herbacea* in China reported by Maurya et al. [7]. Based on the above, there is no dispute that the species used for genome sequencing in this study was *Capparis spinosa* var. *herbacea*.

Currently, genetic research in the Capparaceae family is limited by the lack of its own genomic resources, especially a reference genome. Here, we report a chromosome-level genome assembly of *C. spinosa* var. *herbacea*, with a contig N50 of 9.36 Mb and scaffold N50 of 15.15 Mb, providing the first reference genome for the Capparaceae family. Interestingly, the high TR percentage and GC content of the genome can affect the accuracy of the genome assembly. In this study, the percentage of TR in the *C. spinosa* var. *herbacea* genome was 19.28%, which was much higher than the average value of 8.55% in plants [26]. In addition, we found localized high GC content in the *C. spinosa* var. *herbacea* genome, for example, the GC content of Chr06: 3,700,000–15,800,000 in the *C. spinosa* var. *herbacea* genome was 53.92%, much higher than the genomic GC content of 36.61%, which may affect the assembly accuracy of this segment on Chr06. The effect of assembly quality can be seen at the corresponding Chr06 position in the Hi-C contact map (Supplementary Fig. S2). Although the Illumina Hi-C sequencing favored the anchoring of the scaffolds in the chromosomes, the lack of genetic maps leaves the anchor a bit weak.

Both the chemical systems [27] and chloroplast DNA [28] evidence demonstrated a relatively recent evolutionary relationship between Capparaceae and Brassicaceae and Cleomaceae. The phylogenetic tree of single-copy genes (Fig. 5B) indicated that *C*. *spinosa* var. *herbacea* (Capparaceae) was close to *Arabidopsis thaliana* (Brassicaceae) and *Tarenaya hassleriana* (Cleomaceae) in evolutionary relationship, which was consistent with these findings. WGDs are particularly prevalent in angiosperms and play important roles in the evolutionary history of angiosperms [29]. This *C. spinosa* var. *herbacea* genome assembly can improve the understanding of the timing of WGD events in the Capparaceae family. Because TR genes can affect the distribution of Ks peaks [30], and the *C. spinosa* var. *herbacea* genome had a high proportion of TRs, we calculated Ks and 4DTv separately for the 5 duplication types. The results show that the last duplication of WGD was before that of the other 4 duplication types. Compared with the other duplication types, PD had the highest ratio of Ka/Ks >1, indicating strong positive selection. The peaks of Ks (0.069) and 4DTv (0.013) also confirmed that the duplication of PD was very recent. WGD and Ks results showed that *C. spinosa* var. *herbacea* underwent 3 WGD events (γ-β-α). γ WGD occurred at 128.64–150.54 MYA (Ks peak 2.272–2.659), β WGD occurred at 92.65–102.56 MYA (Ks peak 1.636–1.811), and α WGD occurred at 18.59 MYA (Ks peak 0.328). The α WGD peak was consistent with the results (Ks ∼0.3) reported by Mabry et al. [31] in the Capparaceae family. The separation times of *C. spinosa* var. *herbacea* and *A. thaliana* and *T. hassleriana* were 53.00 MYA (Ks peak 0.936) and 43.31 MYA (Ks peak 0.765).

As a medicinal plant, *C. spinosa* contains various bioactive compounds that have long been used in traditional medicine [10–15], including secondary metabolites such as phenolic compounds and flavonoids, which often play a role in abiotic stress responses and are broadly associated with heat tolerance [6, 32]. The bioactive components of *C. spinosa* from different geographical origins are quite different [33]. The KEGG enrichment analysis of *C. spinosa* var. *herbacea* specific genes (Supplementary Fig. S6A) and expansion genes (Supplementary Fig. S6B) jointly enriched for 2 major classes of DNA repair protein and peroxin 3 pathways, including the KUP system potassium uptake protein, syndetin, FK506-binding protein 4/5, DNA excision repair protein ERCC-6–like, DNA-directed RNA polymerase II subunit RPB1, basic endochitinase B, UDP-sugar pyrophosphorylase, F-type H^+^/Na^+^-transporting ATPase subunit alpha, DNA repair protein REV1, and peroxin 3. The KUP family plays critical roles in K^+^ acquisition and transport, growth and development, and responses to stress [34]. Dehydrin-FK506–binding protein complex could enhance drought tolerance through the ABA-mediated signaling pathway [35]. Besides, specific gene KEGG enrichment analysis also enriched the MFS transporter, disease resistance protein RPM1, and zinc finger SWIM domain−containing protein 3 pathway (Supplementary Table S8).

Over a long period of evolution, *C. spinosa* var. *herbacea* has well adapted itself to drought and high-temperature environments; for instance, 5 genes associated with heat shock protein (HSP) were involved in the top 20 KEGG enriched pathways (Supplementary Fig. S6). The ability of plants to use light energy through photosynthesis declines under stressful conditions, which leads to the production of a large amount of reactive oxygen species because excess light energy has not been used for photosynthesis and ultimately causes photoinhibition and oxidative damage to chloroplasts and other cell structures [36]. *In vivo* and *in vitro* studies showed that when plants are exposed to drought and heat stress, the expression of a series of HSP genes is induced, most of which interact with other proteins in the cell and alter their function, protecting against harmful effects [37–39], and thus finding the enrichment of HSP genes in *C. spinosa* var. *herbacea* is explaining their role in determining drought and high-temperature stress tolerance in *C. spinosa* var. *herbacea*.

In this study, we also presented a chromosome-level genome assembly of *C. spinosa* var. *herbacea* using the combination of PacBio CCS and Hi-C data. The final genome assembly was grouped into 21 chromosomes with a size of 274.53 Mb. The high-quality reference *C. spinosa* var. *herbacea* genome assembled in this study is the first reported genomic resource for the Capparaceae family and can facilitate future studies on the mechanisms of drought and high-temperature resistance in this species, providing a system for studying the diversity, speciation, and evolution of this family.

## Methods

### Plant materials and nucleic acid extraction

The source plant (Fig. 1) is an individual of *Capparis spinosa* var. *herbacea* (Willd.) Fici (NCBI:txid2717819) collected from the wild in Gaochang District, Turpan City, China (42°55ʹ N, 89°10ʹ E), and identified and confirmed by taxonomist Xiyong Wang of Xinjiang Institute of Ecology and Geography, Chinese Academy of Sciences. The material samples of the assembled genome were deposited in the Specimen Museum of Xinjiang Institute of Ecology and Geography, Chinese Academy of Sciences, Urumqi 830011, China (No. XJBI 00108198).

On 14 September 2020, fresh and healthy leaves were harvested and immediately frozen in liquid nitrogen, followed by storage at −80°C in the laboratory before DNA and RNA extraction. Genomic DNA was extracted from the fresh leaf tissue (200 mg) that had been ground in liquid nitrogen using cetyltrimethylammonium bromide buffer (60-minute incubation at 65°C), followed by phenol/chloroform/isoamyl purification (25:24:1) and isopropanol and ethanol precipitation. The resulting purified DNA was resuspended in Tris-EDTA buffer for subsequent sequencing [40]. Total RNA was extracted from the fresh samples of roots, stems, leaves, flowers, and fruits according the instructions of RNAprep Pure Plant Plus Kit (DP441, Tiangen, China). The RNA from the above tissues was mixed in equal amounts and used for RNA sequencing library construction.

### Library construction and sequencing

PacBio library construction and sequencing were performed following the standard protocols provided by PacBio. Genomic DNA was sheared into ∼15-kb fragments by Megaruptor 2. The SMRTbell library was constructed using the SMRTbell Express Template Prep Kit 2.0 (Pacific Biosciences, Menlo Park, CA, USA). Library size and quantity were assessed using the FEMTO Pulse and the Qubit dsDNA HS reagents assay kit (Thermo Fisher Scientific, Waltham, MA, USA). Sequencing primer and Sequel II DNA Polymerase were annealed and bound, respectively, to the final SMRTbell library. The library was loaded at an on-plate concentration of 55 pM using diffusion loading. SMRT sequencing was performed using a single 8-M SMRT Cell on the PacBio Sequel II System (PacBio Sequel II System, RRID:SCR_017990) with a Sequel II Sequencing Kit.

The sequencing for genome survey was performed according to the standard protocol provided by Illumina (San Diego, CA, USA). Using the extracted genomic DNA, small fragment library construction and sequencing were performed. Qualified genomic DNA was fragmented to the target fragment (350 bp) by physical fragmentation (ultrasonic vibration), followed by end repair, polyadenylation, adapter ligation, target fragment selection, and PCR [41]. The library was sequenced with paired-ended 150 bp (PE 150) using the Illumina NovaSeq 6000 platform (Illumina NovaSeq 6000 Sequencing System, RRID:SCR_016387).

Instructions of the VAHTS Universal V6 RNA-seq Library Prep Kit for Illumina (NR604-02; Vazyme, Nanjing China) were followed to construct the transcriptomic short reads library. The constructed library was sequenced on the Illumina NovaSeq 6000 platform. The transcriptomic long reads library was obtained after using the SMRTbell Template Prep Kit to perform damage repair, end repair, and ligation of the mixed products. The reaction was performed in a PCR thermal cycler or a constant temperature metal bath. After RNA reverse transcription and PCR amplification, the library was sequenced on a PacBio Sequel II System.

Hi-C fragment libraries were constructed as reported by Fu et al. [42]. The main procedures included cross-linking DNA, restriction enzyme digestion, end repair, DNA circularization, and DNA purification. This library was sequenced on the Illumina NovoSeq 6000 platform.

### Estimation of genome features

We estimated genome size using genome survey and flow cytometry before genome assembly, respectively. Genomic DNA was resequenced using the Illumina NovaSeq 6000 sequencing platform, and a total of 33.09 Gb of data was obtained for genome survey. The short reads were quality filtered using Fastp (RRID:SCR_016962) v0.23.0 with default parameters [43]. K-Mer Counter (KMC, RRID:SCR_001245) v3.0.0 with the parameters kmc -k17 -t24 -m64 -ci1 -cs20000 @FILES reads tmp and kmc_tools transform reads histogram reads.histo -cx20000 was used to obtain the *k*-mer file from the clean data [44]. GenomeScope (RRID:SCR_017014) 2.0 with the parameters genomescope.R -i reads.histo -o output -k 17 was used to estimate genome heterozygosity, repeat sequences, and size from the *k*-mer file [45].

For flow cytometry–based prediction, samples were placed in 500 µl nuclei extraction buffer, chopped with a sharp blade, and filtered through a 50-µm filter after 60 seconds. This was followed by the addition of 2,000 µl staining buffer with RNase for 15 minutes in the dark. Nuclei suspensions were analyzed by a CyFlow Space flow cytometer (Sysmex Partec, Muenster, Germany) and the corresponding FloMax software (RRID:SCR_014437). The genome size of *C. spinosa* var. *herbacea* was calculated according to the formula “peak (ref)/genome size (ref) = peak (*C. spinosa* var. *herbacea*)/genome size (*C. spinosa* var. *herbacea*)” using *Solanum pimpinellifolium* as a reference genome with a length of 807.6 Mb [46].

### Chromosome-level assembly with Hi-C data

BWA aligner v0.7.17 [47] was used to align the clean Hi-C reads to the assembly results, and uniquely alignable read pairs with mapping quality more than 20 were retained for further analysis. Invalid read pairs, including dangling ends and self-cyclization, religated, and dumped products, were filtered by HiC-Prov2.8.1 [48]. LACHESIS (RRID:SCR_017644) [49] was used for clustered, ordered, and oriented scaffolds onto chromosomes. Parameters for running LACHESIS were as follows: CLUSTER_MAX_LINK_DENSITY = 2; C-LUSTER_MIN_RE_SITES = 9; ORDER_MIN_N_RES_IN_SHREDS = 15; ORDER_MIN_N_RES_IN_TRUN = 15. Clean Hi-C reads, accounting for 100-fold coverage of the survey genome, and the final 28 scaffolds were anchored to chromosomes, accounting for 99.98% of the total length. The Hi-C interactions were used as evidence for contig proximity and scaffold/contig sequences.

### Genome assembly and evaluation

The raw PacBio sequencing reads were assembled using Hifiasm (RRID:SCR_021069) v0.14 [50] with the parameters -l 2 -n 4. Purge_dups (purge dups, RRID:SCR_021173) v1.2.5 (default parameters) [51] was used to identify and remove haplotypic duplication in the genome assembly.

Five methods were used to evaluate the quality of the genome assembly, including the second-generation data return ratio, CEGMA (RRID:SCR_015055) evaluation, BUSCO (RRID:SCR_015008) evaluation, Merqury, and LAI value evaluation. BWA-MEM v0.7.17 (default parameters) [47] was used to compare the short reads obtained from the Illumina HiSeq sequencing data with the reference genome. CEGMA v2.5 [52], which contains 458 conserved core eukaryotic genes, was used to evaluate the completeness of the genome assembly. The Embryophyta database of BUSCO v5.2.1 [53] contains 1,614 conserved core genes that were used to assess the integrity of the genome assembly. Assembly QV was calculated using Merqury v1.3 [54].

Full-length LTR retrotransposons (LTR-RTs) in the genome were identified by LTR_finder (LRRID:SCR_015247) v1.07 [55] and LTRharvest (RRID:SCR_018970) v1.6.1 [56]. LTR_retriever (RRID:SCR_017623) v2.9.0 [57] was then used to combine LTR-RTs, remove duplicates, and calculate the LAI value and calculate the insertion time of LTR-RTs. LTR_finder parameters were -D 40000 -d 100 -L 9000 -l 50 -p 20 -C -M 0.9. LTRharvest parameters were -minlenltr 100 -maxlenltr 40 000 -mintsd 4 -maxtsd 6 -motif TGCA -motifmis 1 -similar 85 -vic 10 -seed 20 -seqids yes. LTR_retriever was set with the parameter -u 7e-9, which is used to set the molecular clock r value to 7*10^−9^ [58].

### Identification of repeat sequences

TEs and TRs were identified separately. We combined homology-based and *de novo* approaches to identify TEs. We first customized a *de novo* repeat library of the genome using RepeatModeler2 v2.0.1 (default parameters) [59]. The *de novo* TE-sequence library and LTR-RT library described above were merged with the known Repbase (RRID:SCR_021169) v19.06, REXdb v3.0, and Dfam (RRID:SCR_021168) v3.2 databases. After removing redundant sequences using the seqkit (RRID:SCR_018926) v2.1.0 (parameter: rmdup -s) [60], a nonredundant species-specific TE library was constructed. TE sequence was identified and classified by RepeatMasker (RRID:SCR_012954) v4.1.1 (default parameters) [61]. TRs were identified by MISA (RRID:SCR_010765) v2.1 [62] with default parameters and TRF v4.09 [63] with the parameters 1 1 2 80 5 200 2000 -d -h.

### Gene prediction and annotation

The 3 approaches of *de novo* prediction, homology search, and transcript-based assembly were used to annotate protein-coding genes [42]. Augustus (RRID:SCR_008417) v3.1 (default parameters) [64] and SNAP 3 (RRID:SCR_009400) v2013-02-16 (default parameters) [65] were used for *de novo* prediction. Homologous species were predicted in GeMoMa (RRID:SCR_017646) v1.7 (default parameters) [66] using the reference gene models of *A. thaliana*, *Cannabis sativa*, *Eutrema salsugineum*, and *T. hassleriana*. For the transcript-based prediction, a total of 11.31 Gb Illumina NovaSeq 6000 RNA sequencing data were mapped to the reference genome using HISAT2 (RRID:SCR_015530) v2.2.0 (parameters: –max-intronlen 20000, –min-intronlen 20) [67] and assembled by StringTie (RRID:SCR_016323) v2.1.3b (default parameters) [68]. GeneMarkS-T (RRID:SCR_017648) v5. 1 (default parameters) [69] was used for gene prediction based on the assembled transcripts. A total of 1.52 Gb of PacBio transcriptome data were used for full-length transcriptome sequence analysis using PacBio Iso Seq3. The PASA (RRID:SCR_014656) v2.4.1 (default parameters) [70] was used to predict genes based on the unigenes (and full-length transcripts from the PacBio sequencing) assembled by Trinity (RRID:SCR_013048) v2.11.0 (parameters: -max_memory 100 g) [71]. Gene models from these different approaches were combined using the EVM (EVidenceModeler, RRID:SCR_014659) v1.1.1 (default parameters) [70] and updated by PASA.

The predicted gene sequences were used as queries for BLAST v2.2.31 72searches against the NR (202,009) [73], TrEMBL (202,005) [74], Pfam v33.1 [75], Swiss-Prot (202,005) [76], KOG (20,110,125) [77], GO (20,200,615) [78], and KEGG (20,191,220) [79] databases for gene annotation.

tRNA was identified using tRNAscan-SE (RRID:SCR_010835) v1.3.1 [80], rRNA was predicted based on the Rfam (RRID:SCR_007891) v12.0 database [81] and Barrnap (RRID:SCR_015995) v0.9 [82], microRNA (miRNA) was identified by the miRBase (mRRID:SCR_003152) v22 database [83], and snoRNA and snRNA were based on the Rfam database and predicted by Infernal v1.1 [84]. A total of 0 transfer RNAs, 2,722 rRNAs, and 100 miRNAs were predicted.

### WGD analysis

GenDup_finder-unique, the stringent mode of DupGen_finder [85], was used to identify genes derived from the different duplication types. DupGen_finder-unique divided the duplication types into 5 types: WGD, TD, PD (separated by fewer than 10 genes on the same chromosome), TRD, and DSD. The Ka, Ks, and Ka/Ks values of gene pairs were calculated with ParaAT v2.0 [86]. The proportion of each homologous gene to the 4DTv site was calculated using a Perl script. Genes with Ka/Ks >1 in the 5 duplication types were used for GO and KEGG enrichment analysis by clusterProfiler (RRID:SCR_016884) v4.2.0 [87].

### Gene family classification

The protein sequences of 16 species (*M. acuminata*, *T. sinense*, *C. micranthum*, *A. trichopoda*, *T. hassleriana*, *A. comosus*, *S. lycopersicum*, *T. cacao*, *A. thaliana*, *B. distachyon*, *N. nucifera*, *P. trichocarpa*, *V. vinifera*, *N. colorata*, *O. sativa*, and *C. spinosa* var. *herbacea*) were used for family classification using OrthoFinder v2.4 software (diamond comparison method, E-value 0.001) [88]. The encoding genes from a species were clustered into 6 groups—0 copies, 1 copy (single copy), 2 copies, 3 copies, 4 copies, and 4+ copies. A total of 306 genes were identified as single-copy genes. The obtained gene families were annotated using the PANTHER (RRID:SCR_004869) v15 database [89].

### Phylogenetic analysis and species divergence time estimation

Each single-copy gene family sequence was aligned using MAFFT (RRID:SCR_011811) v7.205 [90] (parameters: –localpair –maxiterate 1000). Gblocks (RRID:SCR_015945) v0.91b [91] (parameter: -b5 = h) was used to filter conserved sites, and all aligned gene family sequences of each species were finally connected end-to-end to obtain supergenes. The IQ-TREE (RRID:SCR_017254) v1.6.11 [92] model selection tool ModelFinder [93] was used for model selection. The best model obtained was JTT+F+I+G4, which was used to construct the phylogenetic tree by the maximum likelihood (ML) method with the bootstrap value set to 1,000. *A. trichopoda* was selected as the outgroup and the root of the tree [42]. The divergence time between species was estimated using the TimeTree (RRID:SCR_021162) [94]. Divergence times were as follows: *A. trichopoda*vs.*S. lycopersicum* at 164–194 MYA, *O. sativa*vs.*B. distachyon* at 42–60 MYA, *A. comosus*vs.*O. sativa* at 94–115 MYA, and *N. nucifera*vs.*V. vinifera* at 116–127 MYA. The gradient and Hessian parameters required for the divergence time were estimated using MCMCTree in PAML (RRID:SCR_014932) v4.9i [95]. The ML method, correlated molecular clock, and JC69 model were used to estimate divergence times. Two repeated calculations were performed to evaluate consistency. The Markov chain Monte Carlo iteration settings were as follows: burn-in, 2,000; sampfreq, 10; and nsample, 20,000. MCMCtreeR v1.1 [96] was used to graphically display the phylogenetic tree with divergence times.

### Gene family expansion and contraction

The results of the phylogenetic tree (with divergence times) and gene family clustering analyses were used to estimate the gene family expansion and contraction of species relative to their ancestors using CAFE (RRID:SCR_005983) v4.2 [97]. The criteria defining significant expansion or contraction of gene families were a family-wide *P* < 0.05 and a Viterbi *P* < 0.05.

### Genome collinearity analysis

To identify similar gene pairs, gene sequences of 2 species were compared using DIAMOND (RRID:SCR_016071) v0.9.29.130 (parameter: e<1e-5) [98]. JCVI (RRID:SCR_021641) v0.9.13 [99] was used to filter the BLAST results (parameter: C-score >0.5) and obtain all the genes in collinear blocks. JCVI was also used to plot the collinearity of the linear pattern of each species. Finally, the ggplot2 (RRID:SCR_014601) v3.3.5 R package [100] was used to display the collinearity results in the form of bar graphs.

### Genome information visualization

The sliding window file of the genome was constructed using BEDTools (RRID:SCR_006646) v2.29.2 [101], and the window size was set to 100 kb to calculate the gene density of each chromosome. The distribution of gene density, TE sequence, TRs, GC content, and collinearity on the chromosomes of the genome were visualized using Circos (RRID:SCR_011798) v0.69–8 [102].

### Correlation analysis of genomic distribution characteristics

We performed pairwise correlation analysis on the GC content, gene density, TE distribution, and TR distribution of the genome. Correlation analysis was performed using the Spearman method [103] using the cor function in R. The corrplot R package was used to visualize the correlation results.

## Data Availability

Raw data of genome assembly (PacBio HiFi and Hi-C sequences) and genome encoding gene prediction annotations (Illumina and PacBio sequences) have been deposited in the NCBI Sequence Read Archive (SRA) database under Bioproject ID: PRJNA792936. Illumina data were given accession SRR18458236 for the genome size survey. Data for genome PacBio HiFi data were given accession SRR17373648, and Hi-C data have accession SRR17373647. For encoding gene prediction annotations, Illumina data have accession SRR18512706, and PacBio data have accession SRR18512705. The genome annotations have been deposited at FigShare [104]. The whole-genome sequence data have been deposited in the Genome Warehouse at the National Genomics Data Center, Beijing Institute of Genomics, under accession number GWHBGXB00000000. The genome downloads address of other species used in this study are in Supplementary Table S9. All other supporting data and materials are available in the *GigaScience* GigaDB database [105].

## Additional Files

**Supplementary Table S1**. Genome assembly statistic information for *C. spinosa* var. *herbacea*.

**Supplementary Table S2**. BUSCO evaluation result.

**Supplementary Table S3**. Repeat sequence.

**Supplementary Table S4**. Genome encoding gene prediction

**Supplementary Table S5**. Gene’s annotation.

**Supplementary Table S6**. Duplication gene's distribution.

**Supplementary Table S7**. Genome synteny analysis results.

**Supplementary Table S8**. Expansion and specific genes KEGG enrichment results.

**Supplementary Table S9**. Download URLs for reference genomes of other species.

**Supplementary Figure S1**. Genome size estimation of *Capparis spinosa* var. *herbacea* by using genome survey and flow cytometry with *Solanum pimpinellifolium* as reference. (A) The 17-mer distribution of Illumina short reads in *C. spinosa* var. *herbacea*. The x-axis shows the frequency or the number of times of a given *k*-mer (*k*-mer depth). The y-axis shows the total number of *k*-mers with a given frequency (a given depth). Two peaks (blue line) were observed indicating heterozygosity in *C. spinosa* var. *herbacea*. (B) Main peaks of *Solanum pimpinellifolium* and *C. spinosa* var. *herbacea* (samples 1 and 2) were 356.73 and 123.27 (mean value = (122.72 + 123.82)/2), respectively. According to the formula “peak (ref)/genome size (ref) = peak (*C. spinosa* var. *herbacea*)/genome size (*C. spinosa* var. *herbacea*),” the mean value of the genome size of *C. spinosa* var. *herbacea* was estimated as 279.07 Mb.

**Supplementary Figure S2**. Hi-C interaction heat map. Hi-C heat map of 21 chromosomes.

**Supplementary Figure S3**. Correlation analysis of genomic distribution characteristics. (A) Correlation of the genomic GC content, gene density, TE distribution, and TR distribution. (B) Correlation analysis of TR distribution and GC content. Gene: gene density; GC: GC content; TR: distribution of tandem repeats; TE: distribution of transposable elements.

**Supplementary Figure S4**. Enrichment analysis of positively selected genes in gene duplication types. (A) GO enrichment analysis of positively selected genes in 4 duplication types. (B) KEGG enrichment analysis of positively selected genes in 5 duplication types.

**Supplementary Figure S5**. *Capparis spinosa* var. *herbacea* genome collinearity analysis. (A) Dot plots of paralogs in the *C. spinosa* var. *herbacea* genome. (B) *A. trichopoda*, *C. spinosa* var. *herbacea*, and *A. thaliana* gene-level collinearity analysis. (C) *T. cacao* and *C. spinosa* var. *herbacea* gene-level collinearity analysis. (D) *A. thaliana and C. spinosa* var. *herbacea* gene-level collinearity analysis. (E) *V. vinifera*, *C. spinosa* var. *herbacea*, and *S. lycopersicum* genome-level collinearity analysis. (F) *T. hassleriana*, *C. spinosa*var.*herbacea*, and *G. gynandra* genome block-level collinearity analysis. (The *T. hassleriana* genome uses the longest top 50 scaffolds. The order shown in the figure is the sequence numbering order after the top 50 are extracted.)

**Supplementary Figure S6**. KEGG enrichment analysis. (A) KEGG enrichment analysis of *C. spinosa* var. *herbacea* specific genes. (B) KEGG enrichment analysis of expansion genes.

giac106_GIGA-D-22-00058_Original_Submission

giac106_GIGA-D-22-00058_Revision_1

giac106_GIGA-D-22-00058_Revision_2

giac106_GIGA-D-22-00058_Revision_3

giac106_Response_to_Reviewer_Comments_Original_Submission

giac106_Response_to_Reviewer_Comments_Revision_1

giac106_Response_to_Reviewer_Comments_Revision_2

giac106_Reviewer_1_Report_Original_Submission Francesco Mercati -- 4/20/2022 Reviewed

giac106_Reviewer_1_Report_Revision_1 Francesco Mercati -- 7/11/2022 Reviewed

giac106_Reviewer_1_Report_Revision_2 Francesco Mercati -- 8/22/2022 Reviewed

giac106_Reviewer_2_Report_Original_Submission Eric Schranz -- 4/29/2022 Reviewed

giac106_Reviewer_2_Report_Revision_1 Eric Schranz -- 7/12/2022 Reviewed

giac106_Reviewer_2_Report_Revision_2 Eric Schranz -- 8/17/2022 Reviewed

giac106_Supplemental_Files

## Abbreviations

bp: base pair; BUSCO: Benchmarking Universal Single-Copy Orthologs; CAFE: computational analysis of gene family evolution; CCS: circular consensus sequencing; CEGMA: Core Eukaryote Gene Mapping Approach; DSD: dispersed duplication; Gb: gigabase; Hi-C: high-throughput chromosome conformation capture; HSP: heat shock protein; KEGG: Kyoto Encyclopedia of Genes and Genomes; LTR: long terminal repeat; LTR-RTs: long terminal repeat retrotransposons; Mb: megabase; miRNA: microRNA; ML: maximum likelihood; MYA: million years ago; PD: proximal duplication; QV: quality value; rRNA: ribosomal RNA; SRA: Sequence Read Archive; TD: tandem duplication; TE: transposable element; TRs: tandem repeats; TRD: transposed duplication; WGD: whole-genome duplication.

## Competing Interests

The authors declare that they have no conflicts of interest.

## Funding

This work was supported by the National Key Research and Development Program of China (No. 2018YFE0207200) and the Strategic Priority Research Program of the Chinese Academy of Sciences (XDA26010101).

## Authors’ Contributions

L.W.: methodology, sample collection, writing—original draft, writing—review & editing; L.F.: data analysis, methodology, writing—original draft; Z.Z.(Zhenyong Zhao): sample collection, investigation, methodology; L.J.: investigation, methodology; Z.Z.(Zhibin Zhang): data analysis; M.C.: data curation, visualization, supervision, writing—review; C.T.: project administration, supervision, resources, funding acquisition, writing—review & editing.
