# Supplementary material for: The Capparis spinosa var. herbacea genome provides the first genomic instrument for a diversity and evolution study of the Capparaceae family
Source: Gigascience. 2022 Oct 30;11:giac106. doi: 10.1093/gigascience/giac106 (PMC9618406; doi:10.1093/gigascience/giac106)
Supplement: giac106_GIGA-D-22-00058_Original_Submission [file giac106_giga-d-22-00058_original_submission.pdf]

GigaScience

The *Capparis spinosa* genome provides insight into genome evolution of Capparaceae

--Manuscript Draft--

**Manuscript Number:**	GIGA-D-22-00058
**Full Title:**	The *Capparis spinosa* genome provides insight into genome evolution of Capparaceae
**Article Type:**	Data Note
**Funding Information:**	National Key Research and Development Program of China (2018YFE0207200) — Dr. Changyan Tian
**Abstract:**	*Capparis spinosa* L., one of the most economically important species of *Capparaceae*, is a xerophytic shrub that is well adapted to drought and has promising potential for significant adaptation to harsh environments. However, genetic studies on this species are presently limited by the lack of a reference genome.<br>We sequenced and assembled the *C. spinosa* genome using the combination of PacBio CCS sequencing and Hi-C data. The final assembly genome was approximately 274.53 Mb (contig N50 length of 9.36 Mb, scaffold N50 length of 15.15 Mb), 99.23% of which has been assigned to 21 pseudochromosomes. In the genome sequence, tandem repeats accounted for 19.28%, and Transposable elements sequence accounted for 43.98%. The proportion of tandem repeats in the *C. spinosa* genome was much higher than the average of 8.55% in plants. A total of 21,577 protein-coding genes were predicted, with 98.82% being functionally annotated. The result of species divergence times showed that *C. spinosa* and *Tarenaya hassleriana* separated from a common ancestor 27.495 MYA.<br>In summary, this study reported high-quality reference genome assemblies and genome features for *Capparaceae* family for the first time. The assembled *C. spinosa* genome might provide a system for studying the diversity, speciation, and evolution of this family, and it is an important resource for understanding the mechanism of drought and high-temperature resistance.
**Corresponding Author:**	Mao Chai<br>Chinese Academy of Agricultural Sciences Cotton Research Institute<br>Zhengzhou, Henan CHINA
**Corresponding Author Secondary Information:**	
**Corresponding Author's Institution:**	Chinese Academy of Agricultural Sciences Cotton Research Institute
**Corresponding Author's Secondary Institution:**	
**First Author:**	Lei Wang
**First Author Secondary Information:**	
**Order of Authors:**	Lei Wang
	Fanli Qiang
	Zhenyong Zhao
	Zhibin Zhang
	Li Jiang
	Mao Chai
	Changyan Tian
**Order of Authors Secondary Information:**	
**Additional Information:**	

Powered by Editorial Manager® and ProduXion Manager® from Aries Systems Corporation

Question	Response
Are you submitting this manuscript to a special series or article collection?	No
**Experimental design and statistics**<br><br>Full details of the experimental design and statistical methods used should be given in the Methods section, as detailed in our Minimum Standards Reporting Checklist. Information essential to interpreting the data presented should be made available in the figure legends.<br><br>Have you included all the information requested in your manuscript?	Yes
**Resources**<br><br>A description of all resources used, including antibodies, cell lines, animals and software tools, with enough information to allow them to be uniquely identified, should be included in the Methods section. Authors are strongly encouraged to cite Research Resource Identifiers (RRIDs) for antibodies, model organisms and tools, where possible.<br><br>Have you included the information requested as detailed in our Minimum Standards Reporting Checklist?	Yes
**Availability of data and materials**<br><br>All datasets and code on which the conclusions of the paper rely must be either included in your submission or deposited in publicly available repositories (where available and ethically appropriate), referencing such data using a unique identifier in the references and in the "Availability of Data and Materials" section of your manuscript.	Yes

Have you have met the above requirement as detailed in our Minimum Standards Reporting Checklist?	

Manuscript
Click here to access/download;Manuscript;manuscript_GIGAscience.docx

The *Capparis spinosa* genome provides insight into genome evolution of *Capparaceae*

Lei Wang[a,b,1], Fanli Qiang[c,d,1], Zhenyong Zhao[a,b], Zhibin Zhang[c,d], Li Jiang[a,b], Mao Chai[c,d,*] and Changyan Tian[a,b,*]

[1] These authors contributed equally to this work.

[*] Corresponding authors.

Email addresses: chaimol@163.com (M. Chai); tianchy@ms.xjb.ac.cn (C. Tian)

[a] State Key Laboratory of Desert and Oasis Ecology, Xinjiang Institute of Ecology and Geography, Chinese Academy of Sciences, Urumqi 830011, China

[b] University of Chinese Academy of Sciences, Beijing 100049, China

[c] Institute of Cotton Research of the Chinese Academy of Agricultural Sciences, Anyang, Henan 455000, China

[d] Zhengzhou Research Base, State Key Laboratory of Cotton Biology, Zhengzhou University, Zhengzhou 450000, China

Abstract

Background

Capparis spinosa L., one of the most economically important species of *Capparaceae*, is a xerophytic shrub that is well adapted to drought and has promising potential for significant adaptation to harsh environments. However, genetic studies on this species are presently limited by the lack of a reference genome.

Findings

We sequenced and assembled the *C. spinosa* genome using the combination of PacBio CCS sequencing and Hi-C data. The final assembly genome was approximately 274.53 Mb (contig N50 length of 9.36 Mb, scaffold N50 length of 15.15 Mb), 99.23% of which has been assigned to 21 pseudochromosomes. In the genome sequence, tandem repeats accounted for 19.28%, and Transposable elements sequence accounted for 43.98%. The proportion of tandem repeats in the *C. spinosa* genome was much higher than the average of 8.55% in plants. A total of 21,577 protein-coding genes were predicted, with 98.82% being functionally annotated. The result of species divergence times showed that *C. spinosa* and *Tarenaya hassleriana* separated from a common ancestor 27.495 MYA.

Conclusions

In summary, this study reported high-quality reference genome assemblies and genome features for *Capparaceae* family for the first time. The assembled *C. spinosa* genome might provide a system for studying the diversity, speciation, and evolution of this family, and it is an important resource for understanding the mechanism of drought and high-temperature resistance.

Issue Section: Research

Keywords: *Capparis spinosa*; genome assembly; population evolution

Background

Capparis spinosa L., one of the most economically important species of *Capparaceae,* is a perennial

winter deciduous shrub with a wide range, typically growing in the Mediterranean countries and distributed in Iran, Iraq, Saudi Arabia, and China [1-3]. In China, it is mainly found in Xinjiang, Gansu, and Tibet [4].

As a drought-tolerant crop, *C. spinosa* has an extensive root system and a remarkably high root-to-stem ratio and thus has a strong ability to find and absorb water from the environment (especially deep in the soil) as well as a high efficiency of the use of water, resulting in significant adaptability to harsh environments [5, 6]. Besides, the roots, leaves, buds, fruits, bark, and seeds of *C. spinosa* contain a variety of bioactive compounds, such as flavonoid, phenolics, alkaloids, and vitamins that have long been used in the treatment of headaches, toothaches and kidney disease [7-12]. For example, previous study has shown that methanolic extracts prepared from the fruits and flower buds of *C. spinosa* have some anti-inflammatory and anti-thrombotic effects [13]. *C. spinosa* has a huge agricultural potential because of its medicinal properties and its ability to grow under drought conditions. So far, only a few chloroplasts [14-16], mitochondrial [17] genomes, and SSR sequences [18] of genus *Capparis* have been reported, and the taxonomy of the genus *Capparis* is still confusing, and the lack of genomic information hinders the genetic improvement and effective use of caper plants.

Here, we report a high-quality genome sequence of *C. spinosa* using PacBio HiFi sequencing and high-throughput chromosome conformation capture (Hi-C) technology. Detailed information on the *C.*

spinosa genome will help to elucidate the biogeography and evolution of genus *Capparis* plants and contribute to the understanding of the molecular basis of its resistance to stress as well as its medicinal uses.

Analysis

Genome size estimation

We used a single individual of *C. spinosa* that was collected from Xinjiang Institute of Ecology and Geography Chinese Academy of Sciences for whole-genome sequencing. To guide genome sequencing and assembly, we estimated the genome size of *C. spinosa* using flow cytometry [19], which showed that the genome size of *C. spinosa* was 276.44Mb (Fig. S1).

Genome sequencing and assembly

In this study, *C. spinosa* was selected for genome sequencing and the following assembly based on PacBio CCS long reads and Hi-C reads. Total 25,458,659,628 bp PacBio clean long reads with an average read length of 16,618 bp were generated for genome assembly, and a total of 30,635,641,570 bp Hi-C reads were generated for auxiliary genome assembly (Table S1). The primary contigs were assembled with PacBio CCS reads, and a 274.53 Mb genome assembly version was generated with contigs N50 of 11.04 Mb (Table S1). The Hi-C reads were employed for scaffolding into 21 pseudo-chromosomes (Fig. 2, Fig. S2). The final genome assembly version of *C. spinosa* was 274.53 Mb, consisting of 59 contigs and 29 scaffolds. Gene completeness reached up to 99.98%. The contig N50 is

9.36 Mb and the longest contig is 22.51 Mb, while the scaffold N50 is 15.15 Mb and the longest scaffold is 26.66 Mb (Table 1).

For genome quality assessment, BUSCO analysis of the final scaffold assembly showed that 96.80% complete BUSCOs (92.80% complete and single-copy BUSCOs and 4.00% complete and duplicated BUSCOs) were identified (Table S2). Using Merqury revealed a consensus quality score (QV) of 28.27 and assembly accuracy of 99.85% for the assembly. CEGMA were used to evaluate the completeness of the final genome assembly, 98.03% of the CEGMA genes are present in the genome. A total of 98.52% of the short sequences were successfully aligned to the genome. The genome LAI value is 17.19 of our assembly the genome, indicating that the assembly quality of *C. spinosa* reached the level of the reference genome. The above results show the high quality and completeness of the *C. spinosa* genome assembly.

Identification of genomic repetitive sequences

Moreover, a total of 120,748,115 bp (nearly half of the assembled genome length (43.98%)) bases of TE (transposable elements) repetitive sequences in the genome assemblies of *C. spinosa* were also identified by both homology-based and *de novo* methods (Table S3). Retroelement elements constituted the predominant repeat type, accounting for 31.24 % of the genome length. The LTR superfamily elements Copia and DNA transposon elements constituted 29,749,806 and 34,990,312 bp, corresponding to 10.84% and 12.75% of the genome length, respectively. LTR superfamily elements Gypsy and CACTA constituted 11,447,091 and 7,034,814 bp accounting for 4.17% and 2.56% of the genome length, respectively. The density of Copia elements decreased with the increasing density of genes, whereas the DNA transposon elements were distributed more evenly across the genome and showed no obvious patterns or relationships with the distribution of genes (Fig. 2).

The total length of the identified TR（Tandem repeats) is 52,920,691bp, accounting for 19.28% of the total length of the genome. The total length of the identified microsatellite (1-9 bp units) is 43,481,890 bp (15.84%), the total length of minisatellite (10-99 bp units) is 7,039,326 bp (2.56%); the total length of satellite (>=100 bp units) 2,399,475bp (0.87%).

We found the correlation between the distribution of TR sequence and GC content on the chromosome in the *C. spinosa* genome (Fig.2 C, E). So, use Spearman's algorithm to calculate the correlation. The correlation coefficient is -0.52, and the *P-value* is 2.2e-16. It shows that the distribution of TR sequences in the *C. spinosa* genome and the GC content of the sequences show a significant negative correlation.

Genome annotation

A total of 21,577 protein-coding genes (Table 1), 2,722 ribosome RNAs (rRNAs) and 100 microRNAs (miRNAs) (Table S4) were predicted, and more than 98.82% of genes were annotated based on sequence similarity using the following protein-related databases: GO (84.53%), KEGG (76.57%), KOG (59.33%), TrEMBL (98.63%), Pfam (87.75%), Swissprot (84.76%), eggNOG (87.90%), and Nr (98.69%) (Table S5), indicating the gene predictions were accurate.

Table 1. Assembly statistics of *C. spinosa* genome.

Category	Numbers	N50 (Mb)	Longest (Mb)	Size (Mb)	Percentage of assembly
Contigs	59	9.36	22.51	274.53	100
Scaffold	29	15.15	26.66	274.53	100
Anchored	28	15.15	26.66	274.49	99.98

Anchored and oriented	21	15.15	26.66	272.43	99.23
Gene annotated	21,577	NA	NA	64.26	23.42
Repeat sequence	NA	NA	NA	173.60	63.23

Dynamic changes of duplicated genes

Gene footprints in the duplicate events were observed. Duplicated genes were classified into five categories, WGD (whole-genome duplication), TD (tandem duplication), PD (proximal duplication), TRD (transposed duplication), and DSD (dispersed duplication) (Fig. 3A, Table S6). Of the 21,577 genes, 18,432 were identified as duplicate genes, including 9,603 derived from WGD (52.1%), 872 from TD (4.7%), 387 from PD (2.1%), 4,534 from TRD (24.6%), and 3,036 from DSD (16.5%). The Ka (the number of nonsynonymous substitutions per nonsynonymous site), Ks (the number of synonymous substitutions per synonymous site), and Ka/Ks ratio were calculated for different modes of duplication. Among the five replication types, the proportion of gene pairs with Ka/Ks>1 in *A. thaliana* is PD (5.1%), TD (3.3%), DSD (0.6%), TRD (0.3%), WGD (0.0%) However, the corresponding ratios in *C. spinosa* are PD (13.7%), TD (4.9%), DSD (1.3%), TRD (0.9%), WGD (1%). And results showed that PD and TD genes had qualitatively higher Ka/Ks ratios than genes derived from other modes of duplication (Fig. 3B). The PD and TD gene pairs had relatively smaller Ks values (Fig. 3C). In particular, PD content with Ka/Ks>1 in *C. spinosa* (13.7%) was significantly higher than that of *A. thaliana* (5.1%). Then, GO and KEGG enrichment analysis were performed on the Ka/Ks>1 genes in the five repeat types. Then GO enrichment analysis of all five types of duplicates genes found that the duplicates genes exhibited divergent functions. The TRD gene was not enriched to a significant GO term. WGD and DSD

were mainly enriched in the GO term of plastid stroma, chloroplast stroma, obsolete chloroplast part, organellar small ribosomal subunit, and organellar ribosome. While PD and TD shared more enriched GO terms related to pyrroline-5-carboxylate reductase activity, L-proline biosynthetic process, rRNA processing, protein disulfide oxidoreductase activity, peroxisome, cysteine-type peptidase activity, terpene synthase activity, magnesium ion binding, defense response to fungus, rRNA binding, response to wounding, small ribosomal subunit, compared with the others. KEGG enrichment analysis of PD and TD showed that these genes were mainly enriched in heat shock 70kDa protein 1/2/6/8, molecular chaperone HtpG, (-)-germacrene D synthase, KUP system potassium uptake protein, suggesting that the PD and TD genes in *C. spinosa* play important roles in environmental stress tolerance (Fig. S3).

Analyses of genome synteny and whole-genome duplication (WGD)

To analyze the evolution of *C. spinosa* genome, dot plots of longer syntenic blocks within *C. spinosa* genome were completed (Fig. S4A). Moreover, the syntenic blocks and colinear gene pairs between *C. spinosa* and *A. trichopoda*, *C. spinosa* and *A.thaliana*, *C. spinosa* and *V. vinifera*, *C. spinosa* and *S. lycopersicum* were implemented, respectively. Results showed that a total of 19,063 colinear gene pairs on 409 colinear blocks were inferred between *C. spinosa* and *A.thaliana*. 5,501 colinear gene pairs from 17 colinear blocks detected between *C. spinosa* and *A. trichopoda* (Fig. S4B). 16,747 colinear gene pairs from 260 colinear blocks were detected between *C. spinosa* and *T. cacao*. 15,352 colinear gene pairs from 222 colinear blocks detected between *C. spinosa* and *V. vinifera*, and 17,940 colinear gene pairs from 502 colinear blocks were detected between *C. spinosa* and *S. lycopersicum* (Fig. S4E). These results indicated that there were conserved syntenic relationships among all orthologous chromosomes in *V. vinifera*, *C. spinosa*, *S. lycopersicum*, *A. thaliana*, and *T. cacao*. Next, the species divergence times of them were evaluated (Fig. 4B). The result showed that *C. spinosa* and *T. hassleriana* separated from

a common ancestor 27.495 MYA, and the corresponding values for *T. cacao, V. vinifera*, and *M. acuminata* were 58.65 MYA, 87.87 MYA, and 122.46 MYA, respectively. The recent WGD time of *C. spinosa* was also calculated (0.17 MYA). Both the 4DTv and synonymous substitution rate (Ks) value distributions of *C. spinosa* paralogs showed that *C. spinosa* experienced two rounds of WGD, the core eudicot γ WGT event (Ks peak of 0.015 and 4DTv peak of 0.012) and a more recent WGD (Ks peak of 0.32 and 4DTv peak is 0.10) (Fig. 5).

Gene family contraction and expansion

Protein sequences of additional 15 sequenced species, namely *O. sativa, B. distachyon, A. comosus, M. acuminata, C. micranthum, N. nucifera, T. sinense, V. vinifera, S. lycopersicum, A. trichopoda, N. colorata, T. hassleriana, A. thaliana, T. cacao, P. trichocarpa,* together with *C. spinosa*, were downloaded for gene expansion and contraction analyses. As a result, all protein-coding genes were clustered into 49,850 orthographs based on sequence homology. 1,846 gene families were shared by all 16 species, and 142 *C. spinosa*-specific gene families were found (Fig. 4A). Moreover, the GO enrichment analyses revealed that species-specific genes were enriched in defense response to bacterium, oxidation-reduction pathways, and sterol biosynthetic (Fig. S5). Caper, as a medicinal plant, grows in the arid Gobi Desert, which is not conducive to plant growth. Defense response in *C. spinosa* may be enhanced by the retention of defense response-related genes.

Based on the 306 orthogroups of single-copy genes, the phylogenetic tree was constructed and used the MCMCTREE program in PAML to estimate divergence time. The phylogenetic tree identified the closest relationship of *C. spinosa* to *T. hassleriana*. Based on the time tree, the number of gene families that experienced expansion or contraction was estimated by computational analysis of gene family evolution (CAFE). Results showed that in almost species, except *B. distachyon* and *A. thaliana,* more

gene families experienced expansion than contraction. In *C. spinosa,* 26 gene families and 11 gene families expanded and contracted, respectively (Fig. 4B). GO enrichment analysis for the expanded gene families of *C. spinosa* showed that these genes were mainly enriched in chloroplast thylakoid, chloroplast envelope, thylakoid, chloroplast thylakoid membrane, response to abscisic acid, response to the hormone, and so on (Fig. 4C). For KEGG enrichment analysis, the result indicated that the photosynthesis-related of the chloroplast thylakoid membrane and photosynthesis, and response to abscisic acid of hormone-related pathways were enriched (Fig. S5). The function for these gene families expanded in *C. spinosa*, indicating that the expansion of the hormone response pathway and the photosynthesis pathway may have helped *C. spinosa* to generate more energy to adapt to the arid environments.

Discussion

Currently, genetic research in the *Capparaceae* family is limited by the lack of its own genomic resources, especially reference genome. Here, we report a chromosome-scale genome assembly of *C. spinosa*, with a contig N50 of 9.36 Mb and scaffold N50 of 15.15 Mb, providing the first reference genome for the *Capparaceae* family. The genome assembly was 274.53 Mb in length, and >99.23% of the assembled genome was placed on 21 chromosomes. This represents a more contiguous and higher-quality genome assembly than that of recently sequenced Brassicaceae species genomes, such as eight oilseed rape lines [20], with a contig N50 2.1~3.1 Mb, as well as field pennycress [21], with a contig N50 4.18 Mb. The high quality of our assembly can be attributed to the use of the combination of PacBio HiFi sequencing and Hi-C data.

However, since the proportion of TR in the *C. spinosa* genome is 19.28%, it is much higher than the average value of 8.55% in plants [22]. A higher TR ratio will seriously affect the accuracy of genome assembly. At the same time, we also found that the distribution of TR in the genome of *C. spinosa* is

inversely proportional to the content of GC. The local high GC content in the genome seriously affects the accuracy of Hi-C assembly. For example, the GC content of *C. spinosa* genome Chr06: 3700000-15800000 is 53.92%, which is much higher than the genome GC content 36.61%. High GC content may affect the accuracy of assembly of this segment on Chr06 chromosome. It may also be due to the high local GC content and high TR ratio that increase the difficulty of genome assembly. The *C. spinosa* genome assembled by us is the only genome of *Capparaceae* that has reached the chromosome level so far.

Whole-genome duplications are particularly prevalent in angiosperms and play important roles in the evolutionary history of angiosperms [23]. The present assembly of the *C. spinosa* genome has improved the understanding of the timing of WGD events in the *Capparaceae* family. Both the Ks and 4DTv value distributions showed that *C. spinosa* experienced two rounds of WGD: one was the ancient WGD event (Ks peak of 2.41 and 4DTv peak of 0.35) and the second was a modern WGD event (Ks peak of 0.36 and 4DTv peak is 0.11) (Fig. 5). Compared to *A. thaliana*, *S. lycopersicum, T. cacao, T. hassleriana* and *V. vinifera* species, the WGD times for *C. spinosa* were smaller (Fig. 4), which may be since smaller populations may have less time to expand, which is supported by the relatively young age (small Ks and 4DTv values) of the WGD in smaller populations [24].

As a medicinal plant, *C. spinosa* contains various bioactive compounds that have long been used in traditional medicine [7-12], which include terpenoids. In this study, GO enrichment analysis for positively selected genes revealed that seven genes associated with terpene synthase activity were involved in the top 20 enriched pathways (Figure S5). Among plant secondary metabolites, terpenoids constitute the most abundant and structurally diverse group [25]. TPSs are pivotal enzymes for the biosynthesis of terpenoids. Some research has been performed in terpenoid production through the expression of the TPS genes [26-32].

Over a long period of evolution, the drought and high temperature environments have resulted in *C. spinosa* that have become drought and heat tolerant. In this study, KEGG enrichment analysis for positively selected genes showed that five genes associated with heat shock protein (HSP) were involved in the top 20 enriched pathways (Figure S5). It has been shown that the ability of plants to use light energy through photosynthesis declines under stressful conditions, which leads to the production of a large amount of ROS because excess light energy has not been used for photosynthesis, and ultimately causes photoinhibition and oxidative damage to chloroplasts and other cell structures [33]. In vivo and in vitro studies showed that when plants are exposed to drought and heat stress, the expression of a series of HSP genes are induced, most of which interact with other proteins in the cell and alter their function, protecting against harmful insults [34-36], thus allowing the continuous accumulation of HSPs gene expression in *C. spinosa* under drought and high-temperature environments.

Methods

Plant materials

The source plant was an individual of *C. spinosa* grown in the field near the Turpan Eremophyte Botanical Garden, Xinjiang Institute of Ecology and Geography, Chinese Academy of Sciences (40°51′ N, 98 °11′ E, -75 m elevation). On September 14, 2020, fresh and healthy leaves were harvested and immediately frozen in liquid nitrogen, followed by storage at -80°C in the laboratory prior to DNA extraction.

PacBio library construction and sequencing

All procedures for PacBio library construction and sequencing follow the standard protocols provided

by PacBio, which involve shearing the DNA sample using g-TUBE, repairing the damaged DNA and DNA termini, connecting dumbbell-shaped adapters, performing exonuclease digestion, and target fragment screening using BluePippin. The PacBio II sequencing platform to obtain 25.46Gb CCS clean data, accounting for 100-fold coverage of the draft genome of *C. spinosa*.

Chromosome-scale assembly with Hi-C data

Hi-C fragment libraries were constructed as reported by Fu et al. [37]. De novo genome assembly based on chromatin interactions was performed using LACHESIS [38] with the following parameters: CLUSTER_MAX_LINK_DENSITY=2; C-LUSTER_MIN_RE_SITES=9; ORDER_MIN_N_RES_IN_SHREDS=15; ORDER_MIN_N_RES_IN_TRUN=15. Clean Hi-C reads, accounting for 100-fold coverage of the draft genome of *C. spinosa*, and the final 28 scaffolds were anchored to chromosomes, accounting for 99.98% of the total length. The Hi-C interactions were used as evidence for contig proximity and in scaffolding contig sequences.

Genome assembly and evaluation

The raw PacBio sequencing reads were assembled using Hifiasm V0.14 [39] with parameters -l 2 -n 4. Purge_dups V1.2.5 (default parameters) [40] was used to identify and remove haplotypic duplication in genome assemblies.

Four methods were used to evaluate the quality of genome assembly, including the second-generation data return ratio, CEGMA evaluation, BUSCO evaluation, ,Merqury and LAI value evaluation. Align the short sequence obtained by Illumina HiSeq with the reference genome using the default parameters of the BWA-MEM V0.7.17 [41]. CEGMA V2.5 (default parameters) [42] database

contains 458 conserved core genes in eukaryotes were used to evaluate the completeness of the final genome assembly. The embryophyta database of BUSCO V5.2.1 [43] contains 1,614 conserved core genes that were used to assess the integrity of the genome assembly. The assembly quality score (QV) was calculated using Merqury version 1.3 [44].

Full-length LTR repeat retrotransposons (LTR-RTs) in the genome were identified by LTR_finder V1.07 [45] and LTRharvest V1.6.1 [46]. LTR_retriever V2.9.0 [47] was then used to combine the LTR retrotransposons, remove duplicates and calculate the LAI value and calculate the insertion time of LTR-RTs. LTR_finder was set with the parameter: -D 40000 -d 100 -L 9000 -l 50 -p 20 -C -M 0.9. LTRharvest was set with the parameter: -minlenltr 100 -maxlenltr 40000 -mintsd 4 -maxtsd 6 -motif TGCA -motifmis 1 -similar 85 -vic 10 -seed 20 -seqids yes. LTR_retriever was set with the parameter -u 7e-9, which is used to set the molecular clock r value was $7*10^{-9}$ [48].

Repeat sequences identify

Transposon element (TE) and tandem repeat were identified separately. We combined of homology-based and de novo approaches to identify TE. We first customized a de novo repeat library of the genome using RepeatModeler2 V2.0.1 (default parameters). By combining the above de novo TE sequence library and LTR-RTs library with the known Repbase V19.06, REXdb V3.0, and Dfam V3.2 databases, a non-redundant species-specific TE library was constructed. TE sequence was identified and classified by RepeatMasker V4.1.1 (default parameters) [49]. Tandem repeats (TR) were identified by MISA V2.1 [50] with default parameters and TRF V4.09 [51] with the parameters: 1 1 2 80 5 200 2000 -d -h.

Gene prediction and annotation

Three approaches, de novo prediction, homology search, and transcript-based assembly to annotate protein-coding genes, as described by Fu et al.[37]. HISAT2 parameters were as follows: --max-intronlen 20000, --min-intronlen 20. For PASA, the parameters were as follows: -align_tools gmap, -maxIntronLen 20000. Other software use default parameters.

The predicted gene sequences were used as queries for BLAST V2.2.31 (Altschul et al., 1990) searches against the NR (202009) [52], TrEMBL (202005) [53], Pfam V33.1 [54], SwissProt (202005) [55], KOG (20110125) [56], GO (20200615) [57] and KEGG (20191220) [58] databases for gene annotation. The tRNAscan-SE V1.3.1 [59] was used to identify tRNA; rRNA prediction was mainly based on Rfam V12.0 [60] database and Barrnap V0.9 [61] was predicted; miRNA was identified by miRbase V22 [62] database; snoRNA and snRNA were based on Rfam V12.0 database and use Infenal V1.1 [63] to make predictions. A total of 0 tRNAs, 2,722 rRNAs, and 100 miRNAs were predicted.

Whole-genome gene duplication analysis

GenDup_finder-unique the stricter version of DupGen_finder [64] was used to identify genes derived from different modes of gene duplication: WGD, TD, PD (separated by fewer than ten genes on the same chromosome), TRD, and DSD. The Ka, Ks, and Ka/Ks values of gene pairs were calculated with ParaAT V2.0 [65]. The proportion of each homologous gene to the 4DTv site was calculated using Perl script. Genes with Ka/Ks>1 in different replication modes were used for GO and KEGG enrichment analysis by clusterProfiler V4.2.0 [66].

Gene family classification

The protein sequences of 16 species (*Musa acuminata, Tetracentron sinense, Combretum micranthum, Amborella trichopoda, Tarenaya hassleriana, Ananas comosus, Solanum lycopersicum, Theobroma cacao*, *Arabidopsis thaliana*, *Brachypodium distachyon, Nelumbo nucifera*, *Populus trichocarpa*, *Vitis vinifera*, *Nymphaea colorata*, *Oryza sativa,* and *C. spinosa*) were used to carry out family classification using Orthofinder V2.4 software [67] (diamond comparison method, E-value 0.001). The encoding genes from a species were clustered into six groups, including 0 copies, 1 copy (single-copy), 2 copies, 3 copies, 4 copies, and 4+ copies. A total of 306 genes were identified as single-copy genes. The obtained gene families were annotated using the PANTHER V15 database [68].

Phylogenetic analysis and species divergence time estimation

Use MAFFT V7.205 [69] to align each single-copy gene family sequence (parameter: --localpair --maxiterate 1000), and then use Gblocks V0. 91b [70] (parameter: -b5=h) filter the conserved sites, and finally all aligned genes of each species the family sequence was connected end to end to get the supergene, and then use IQ-TREE V1.6.11 [71] model detection tool ModelFinder [72] for model detection, the best model obtained was JTT+F+I+G4, and then using this best model, the evolutionary tree was constructed by the maximum likelihood (ML) method, and the number of bootstrap was set to 1000.

The MCMCTREE package in PAML V4.9i [73] software was used to calculate the divergence time with *A. trichopoda* as the outgroup of the root tree. Using TimeTree (http://www.timetree.org/), the divergence times were estimated as follows: *A. trichopoda* Vs *S. lycopersicum* at 164-194 MYA, *O. sativa* Vs *B. distachyon* at 42-60 MYA, *A. comosus* Vs *O. sativa* at 94-115 MYA, *N. nucifera* Vs *V.*

vinifera at 116-127 MYA. Then use the module MCMCTREE under PAML to estimate the gradient and Hessian parameters required for the divergence time; the maximum likelihood method and the correlated molecular clock and the JC69 model to estimate the divergence time. Two repeated calculations to observe the consistency (The correlation between the two repetitions of this experiment is 1). The number of iterations of the Markov chain is set to: burnin 5000000, sampfreq 30, nsample 10000000. MCMCTreeR V1.1 [74] was used to graphically display the evolutionary trees with differentiation times.

The expansion and contraction of gene family

Results of the phylogenetic tree with divergence time and gene family clustering were used to predict the contraction and expansion of the species' gene families relative to their ancestors using CAFE V4.2 [75]. The criteria defining significant expansion or contraction for gene families were a family-wide *P*-value < 0.05 and a Viterbi *P*-value < 0.05.

Genome collinearity analysis

Diamond V0.9.29.130 [76] (parameter: e<1e-5) was used to compare the gene sequences of two species to identify similar gene pairs. JCVI V0.9.13 [77] was used to filter the blast results (parameter: C-score>0.5) and obtain all the genes in collinear blocks. And then JCVI was performed to plot the collinearity of the linear pattern of each species. R package ggplot2 V3.3.5 [78] was finally applied to display the collinearity results in the form of bar graphs.

Genome information visualization

Bedtools V2.29.2 [79] was used to construct the sliding window file of the genome, the window size was set to 100kb, and it was used to calculate the gene density of each chromosome. Circos V0.69-8 [80] was used to visualize the distribution of gene density, TE sequence, Tandem repeats, GC content, and collinearity on the chromosomes of the genome.

Data Availability

Raw data of genome PacBio HiFi and Hi-C were deposited in the NCBI Sequence Read Archive (SRA) database under Bioproject ID: **PRJNA792936**. The genome annotations have been deposited at FigShare (https://doi.org/**10.6084/m9.figshare.17702051**). The whole genome sequence data have been deposited in the Genome Warehouse at the National Genomics Data Center, under accession number **GWHBGXB00000000**, which is publicly accessible at https://ngdc.cncb.ac.cn/gwh.

List of abbreviations

CAFE: computational analysis of gene family evolution; DSD: dispersed duplication; Hi-C: high-throughput chromosome conformation capture; HSP: heat shock protein; LTR-RTs: LTR repeat retrotransposons; MYA: million years ago; PD: proximal duplication; QV: quality score; SRA: Sequence Read Archive; TD: tandem duplication; TE: Transposon element; TR: Tandem repeats; TRD: transposed duplication; WGD: whole-genome duplication.

Ethical statement

Not applicable

Consent for publication

Not applicable

Competing interests

The authors declare that they have no conflict of interest.

Funding

This work was supported by the National Key Research and Development Program of China (No. 2018YFE0207200).

Authors' contributions

Lei Wang: Methodology, Sample collection, Writing - Original draft, Writing - Review & Editing. **Liqiang Fan**: Data analysis, Methodology. Writing - Original draft. **Zhenyong Zhao**: Sample collection, Investigation, Methodology. **Li Jiang**: Investigation, Methodology. **Zhibin Zhang**: Data analysis. **Mao Chai**: Data curation, Visualization, Supervision, Writing - Review. **Changyan Tian**: Project administration, Supervision, Resources, Funding acquisition, Writing - Review & Editing.

Acknowledgements

We thank Dr. Zhaoen Yang (State Key Laboratory of Cotton Biology, Institute of Cotton Research of the Chinese Academy of Agricultural Sciences, Zhengzhou, China) for providing help in data analysis.

References

1. Inocencio C, Rivera D, Obón MC, Alcaraz F and Barreña J-A. A systematic revision of capparis section Capparis (Capparaceae) 1, 2. Annals of the Missouri Botanical Garden. 2006;93 1:122-49.
2. Levizou E, Drilias P and Kyparissis A. Exceptional photosynthetic performance of Capparis spinosa L. under adverse conditions of Mediterranean summer. Photosynthetica. 2004;42 2:229-

35.

3. Özcan M and Akgül A. Influence of species, harvest date and size on composition of capers (Capparis spp.) flower buds. Food/Nahrung. 1998;42 02:102-5.
4. Yang T, Liu Y-Q, Wang C-H and Wang Z-T. Advances on investigation of chemical constituents, pharmacological activities and clinical applications of Capparis spinosa. Zhongguo Zhong yao za zhi= Zhongguo zhongyao zazhi= China journal of Chinese materia medica. 2008;33 21:2453-8.
5. Gan L, Zhang C, Yin Y, Lin Z, Huang Y, Xiang J, et al. Anatomical adaptations of the xerophilous medicinal plant, Capparis spinosa, to drought conditions. Horticulture, Environment, and Biotechnology. 2013;54 2:156-61.
6. Zuo W, Ma M, Ma Z, Gao R, Guo Y, Jiang W, et al. Study of photosynthetic physiological characteristics of desert plant Capparis spinosa L. Journal of Shihezi University (Natural Science). 2012;30 3:7.
7. Anwar F, Muhammad G, Hussain MA, Zengin G, Alkharfy KM, Ashraf M, et al. Capparis spinosa L.: A plant with high potential for development of functional foods and nutraceuticals/pharmaceuticals. International Journal of Pharmacology. 2016;12 3:201-19.
8. Arrar L, Benzidane N, Krache I, Charef N, Khennouf S and Baghiani A. Comparison between polyphenol contents and antioxidant activities of different parts of Capparis spinosa L. Pharmacognosy Communications. 2013;3 2:70.
9. Germano MP, De Pasquale R, D'angelo V, Catania S, Silvari V and Costa C. Evaluation of extracts and isolated fraction from Capparis spinosa L. buds as an antioxidant source. Journal of agricultural and food chemistry. 2002;50 5:1168-71.
10. Matthäus B and Özcan M. Glucosinolates and fatty acid, sterol, and tocopherol composition of seed oils from Capparis spinosa Var. spinosa and Capparis ovata Desf. Var. canescens (Coss.) Heywood. Journal of Agricultural and Food chemistry. 2005;53 18:7136-41.
11. Tlili N, Feriani A, Saadoui E, Nasri N and Khaldi A. Capparis spinosa leaves extract: Source of bioantioxidants with nephroprotective and hepatoprotective effects. Biomedicine & Pharmacotherapy. 2017;87:171-9.
12. Tlili N, Nasri N, Khaldi A, Triki S and MUNNÉ- BOSCH S. Phenolic compounds, tocopherols, carotenoids and vitamin C of commercial caper. Journal of Food Biochemistry. 2011;35 2:472-83.
13. Bektas N, Arslan R, Goger F, Kirimer N and Ozturk Y. Investigation for anti-inflammatory and anti-thrombotic activities of methanol extract of Capparis ovata buds and fruits. Journal of ethnopharmacology. 2012;142 1:48-52.
14. Hall JC. Systematics of Capparaceae and Cleomaceae: an evaluation of the generic delimitations

of Capparis and Cleome using plastid DNA sequence data. Botany. 2008;86 7:682-96.

15. Siragusa M and Carimi F. Development of specific primers for cpSSR analysis in caper, olive and grapevine using consensus chloroplast primer pairs. Scientia horticulturae. 2009;120 1:14-21.

16. Wang Q, Zhang M-L and Yin L-K. Phylogeographic structure of a tethyan relict Capparis spinosa (Capparaceae) traces Pleistocene geologic and climatic changes in the western Himalayas, Tianshan mountains, and adjacent desert regions. BioMed research international. 2016;2016:13.

17. Grewe F, Edger PP, Keren I, Sultan L, Pires JC, Ostersetzer-Biran O, et al. Comparative analysis of 11 Brassicales mitochondrial genomes and the mitochondrial transcriptome of Brassica oleracea. Mitochondrion. 2014;19:135-43.

18. Mercati F, Fontana I, Gristina AS, Martorana A, El Nagar M, De Michele R, et al. Transcriptome analysis and codominant markers development in caper, a drought tolerant orphan crop with medicinal value. Scientific reports. 2019;9 1:1-16.

19. DOLEŽEL J and BARTOŠ J. Plant DNA Flow Cytometry and Estimation of Nuclear Genome Size. Annals of Botany. 2005;95 1:99-110. doi:10.1093/aob/mci005.

20. Song JM, Guan Z, Hu J, Guo C and Guo L. Eight high-quality genomes reveal pan-genome architecture and ecotype differentiation of Brassica napus. Nature Plants. 2020;6 1:1-12.

21. Geng Y, Guan Y, Qiong, Lu S, An M, Crabbe M, et al. Genomic analysis of field pennycress (Thlaspi arvense) provides insights into mechanisms of adaptation to high elevation. BMC biology. 2021;19 1:143. doi:10.1186/s12915-021-01079-0.

22. Tørresen OK, Star B, Mier P, Andrade-Navarro MA, Bateman A, Jarnot P, et al. Tandem repeats lead to sequence assembly errors and impose multi-level challenges for genome and protein databases. Nucleic Acids Research. 2019;47 21:10994-1006. doi:10.1093/nar/gkz841.

23. Christenhusz MJM and Byng JW. The number of known plants species in the world and its annual increase. Phytotaxa. 2016;261 3:201-17.

24. Ren, Wang, HF, Guo, CC, Zhang, et al. Widespread Whole Genome Duplications Contribute to Genome Complexity and Species Diversity in Angiosperms. MOL PLANT. 2018;2018,11(3) -:414-28.

25. Bohlmann J, Meyer-Gauen G and Croteau R. Plant terpenoid synthases: Molecular biology and phylogenetic analysis. Proc Natl Acad Sci U S A. 1998;95 8:4126-33.

26. Berta A, Ana R, Marcos D and Leandro P. Genomic Analysis of Terpene Synthase Family and Functional Characterization of Seven Sesquiterpene Synthases from Citrus sinensis. Frontiers in Plant Science. 2017;8.

27. Chen C, Zheng Y, Zhong Y, Wu Y and Meng X. Transcriptome analysis and identification of

genes related to terpenoid biosynthesis in Cinnamomum camphora. BMC Genomics. 2018;19 1.

28. Chen H, Kllner TG, Li G, Wei G and Chen F. Combinatorial Evolution of a Terpene Synthase Gene Cluster Explains Terpene Variations in Oryza. Plant Physiology. 2019;182 1:pp.00948.2019.

29. Hansen NL, Heskes AM, Hamberger B, Olsen CE and Hamberger B. The terpene synthase gene family in Tripterygium wilfordii harbors a labdane-type diterpene synthase among the monoterpene synthase TPS-b subfamily. Plant Journal. 2017;89 3.

30. Karunanithi PS and Zerbe P. Terpene Synthases as Metabolic Gatekeepers in the Evolution of Plant Terpenoid Chemical Diversity. Frontiers in Plant Science. 2019;10:1166-.

31. Shu-Ye J, Jingjing J, Rajani S and Srinivasan R. A Comprehensive Survey on the Terpene Synthase Gene Family Provides New Insight into Its Evolutionary Patterns. Genome Biology and Evolution. 2019; 8:8.

32. Xiong W, Wu P, Jia Y, Wei X, Xu L, Yang Y, et al. Genome-wide analysis of the terpene synthase gene family in physic nut (Jatropha curcas L.) and functional identification of six terpene synthases. Tree Genetics & Genomes. 2016;12 5:97.

33. Singh AK and Singhal GS. Effect of Irradiance on the Thermal Stability of Thylakoid Membrane Isolated from Acclimated Wheat Leaves. Photosynthetica. 2001;39 1:23-7.

34. Ohama N, Sato H, Shinozaki K and Yamaguchi-Shinozaki K. Transcriptional Regulatory Network of Plant Heat Stress Response. Trends in Plant Science. 2017;22 1:53-65.

35. Ren S, Ma K, Lu Z, Chen G and Jin B. Transcriptomic and Metabolomic Analysis of the Heat-Stress Response of Populus tomentosa Carr. Forests. 2019;10 5:383.

36. Tereza T, Despina S, Anna K, Tereza V and Jozef Š. Multifaceted roles of HEAT SHOCK PROTEIN 90 molecular chaperones in plant development. Journal of Experimental Botany. 2020;71 14:20.

37. Fu A, Wang Q, Mu J, Ma L, Wen C, Zhao X, et al. Combined genomic, transcriptomic, and metabolomic analyses provide insights into chayote (Sechium edule) evolution and fruit development. Horticulture Research. 2021;8 1:35. doi:10.1038/s41438-021-00487-1.

38. Burton JN, Adey A, Patwardhan RP, Qiu R, Kitzman JO and Shendure J. Chromosome-scale scaffolding of de novo genome assemblies based on chromatin interactions. Nature Biotechnology. 2013;31 12:1119-25. doi:10.1038/nbt.2727.

39. Cheng H, Concepcion GT, Feng X, Zhang H and Li H. Haplotype-resolved de novo assembly using phased assembly graphs with hifiasm. Nature Methods. 2021;18 2:170-5. doi:10.1038/s41592-020-01056-5.

40. Guan D, McCarthy SA, Wood J, Howe K, Wang Y and Durbin R. Identifying and removing

haplotypic duplication in primary genome assemblies. Bioinformatics. 2020;36 9:2896-8. doi:10.1093/bioinformatics/btaa025.

41. Li H. Aligning sequence reads, clone sequences and assembly contigs with BWA-MEM. arXiv preprint arXiv:13033997. 2013.

42. Parra G, Bradnam K and Korf I. CEGMA: a pipeline to accurately annotate core genes in eukaryotic genomes. Bioinformatics. 2007;23 9:1061-7.

43. Manni M, Berkeley MR, Seppey M, Simão FA and Zdobnov EM. BUSCO Update: Novel and Streamlined Workflows along with Broader and Deeper Phylogenetic Coverage for Scoring of Eukaryotic, Prokaryotic, and Viral Genomes. Molecular Biology and Evolution. 2021;38 10:4647-54. doi:10.1093/molbev/msab199.

44. Rhie A, Walenz BP, Koren S and Phillippy AM. Merqury: reference-free quality, completeness, and phasing assessment for genome assemblies. Genome Biology. 2020;21 1:245. doi:10.1186/s13059-020-02134-9.

45. Xu Z and Wang H. LTR_FINDER: an efficient tool for the prediction of full-length LTR retrotransposons. Nucleic Acids Research. 2007;35 suppl_2:W265-W8. doi:10.1093/nar/gkm286.

46. Ellinghaus D, Kurtz S and Willhoeft U. LTRharvest, an efficient and flexible software for de novo detection of LTR retrotransposons. BMC bioinformatics. 2008;9 1:1-14.

47. Ou S and Jiang N. LTR_retriever: A Highly Accurate and Sensitive Program for Identification of Long Terminal Repeat Retrotransposons. Plant Physiology. 2018;176 2:1410-22. doi:10.1104/pp.17.01310.

48. Ossowski S, Schneeberger K, Lucas-Lledó JI, Warthmann N, Clark RM, Shaw RG, et al. The rate and molecular spectrum of spontaneous mutations in Arabidopsis thaliana. science. 2010;327 5961:92-4.

49. Tarailo- Graovac M and Chen N. Using RepeatMasker to identify repetitive elements in genomic sequences. Current protocols in bioinformatics. 2009;25 1:4.10.1-4..4.

50. Beier S, Thiel T, Münch T, Scholz U and Mascher M. MISA-web: a web server for microsatellite prediction. Bioinformatics. 2017;33 16:2583-5. doi:10.1093/bioinformatics/btx198.

51. Benson G. Tandem repeats finder: a program to analyze DNA sequences. Nucleic acids research. 1999;27 2:573-80.

52. Marchler-Bauer A, Lu S, Anderson JB, Chitsaz F, Derbyshire MK, DeWeese-Scott C, et al. CDD: a Conserved Domain Database for the functional annotation of proteins. Nucleic acids research. 2010;39 suppl_1:D225-D9.

53. Boeckmann B, Bairoch A, Apweiler R, Blatter M-C, Estreicher A, Gasteiger E, et al. The SWISS-PROT protein knowledgebase and its supplement TrEMBL in 2003. Nucleic acids

research. 2003;31 1:365-70.
54. Mistry J, Chuguransky S, Williams L, Qureshi M, Salazar GA, Sonnhammer EL, et al. Pfam: The protein families database in 2021. Nucleic Acids Research. 2021;49 D1:D412-D9.
55. Boutet E, Lieberherr D, Tognolli M, Schneider M, Bansal P, Bridge AJ, et al. UniProtKB/Swiss-Prot, the Manually Annotated Section of the UniProt KnowledgeBase: How to Use the Entry View. In: Edwards D, editor. Plant Bioinformatics: Methods and Protocols. New York, NY: Springer New York; 2016. p. 23-54.
56. Koonin EV, Fedorova ND, Jackson JD, Jacobs AR, Krylov DM, Makarova KS, et al. A comprehensive evolutionary classification of proteins encoded in complete eukaryotic genomes. Genome biology. 2004;5 2:R7.
57. Dimmer EC, Huntley RP, Alam-Faruque Y, Sawford T, O'Donovan C, Martin MJ, et al. The UniProt-GO annotation database in 2011. Nucleic acids research. 2012;40 D1:D565-D70.
58. Kanehisa M and Goto S. KEGG: kyoto encyclopedia of genes and genomes. Nucleic acids research. 2000;28 1:27-30.
59. Chan PP and Lowe TM. tRNAscan-SE: searching for tRNA genes in genomic sequences. Gene prediction. Springer; 2019. p. 1-14.
60. Nawrocki EP, Burge SW, Bateman A, Daub J, Eberhardt RY, Eddy SR, et al. Rfam 12.0: updates to the RNA families database. Nucleic acids research. 2015;43 D1:D130-D7.
61. Loman T. A Novel Method for Predicting Ribosomal RNA Genes in Prokaryotic Genomes. 2017.
62. Kozomara A, Birgaoanu M and Griffiths-Jones S. miRBase: from microRNA sequences to function. Nucleic acids research. 2019;47 D1:D155-D62.
63. Nawrocki EP and Eddy SR. Infernal 1.1: 100-fold faster RNA homology searches. Bioinformatics. 2013;29 22:2933-5.
64. Qiao X, Li Q, Yin H, Qi K, Li L, Wang R, et al. Gene duplication and evolution in recurring polyploidization–diploidization cycles in plants. Genome Biology. 2019;20 1:38. doi:10.1186/s13059-019-1650-2.
65. Zhang Z, Xiao J, Wu J, Zhang H, Liu G, Wang X, et al. ParaAT: a parallel tool for constructing multiple protein-coding DNA alignments. Biochemical and biophysical research communications. 2012;419 4:779-81.
66. Wu T, Hu E, Xu S, Chen M, Guo P, Dai Z, et al. clusterProfiler 4.0: A universal enrichment tool for interpreting omics data. The Innovation. 2021;2 3:100141.
67. Emms DM and Kelly S. OrthoFinder: phylogenetic orthology inference for comparative genomics. Genome Biology. 2019;20 1:238. doi:10.1186/s13059-019-1832-y.
68. Mi H, Muruganujan A, Ebert D, Huang X and Thomas PD. PANTHER version 14: more genomes, a new PANTHER GO-slim and improvements in enrichment analysis tools. Nucleic

Acids Research. 2018;47 D1:D419-D26. doi:10.1093/nar/gky1038.

69. Katoh K and Standley DM. MAFFT multiple sequence alignment software version 7: improvements in performance and usability. Molecular biology and evolution. 2013;30 4:772-80.

70. Talavera G and Castresana J. Improvement of phylogenies after removing divergent and ambiguously aligned blocks from protein sequence alignments. Systematic biology. 2007;56 4:564-77.

71. Nguyen L-T, Schmidt HA, Von Haeseler A and Minh BQ. IQ-TREE: a fast and effective stochastic algorithm for estimating maximum-likelihood phylogenies. Molecular biology and evolution. 2015;32 1:268-74.

72. Kalyaanamoorthy S, Minh BQ, Wong TK, Von Haeseler A and Jermiin LS. ModelFinder: fast model selection for accurate phylogenetic estimates. Nature methods. 2017;14 6:587-9.

73. Yang Z. PAML: a program package for phylogenetic analysis by maximum likelihood. Computer applications in the biosciences : CABIOS. 1997;13 5:555-6.

74. Puttick MN. MCMCtreeR: functions to prepare MCMCtree analyses and visualize posterior ages on trees. Bioinformatics. 2019;35 24:5321-2.

75. Han MV, Thomas GWC, Lugo-Martinez J and Hahn MW. Estimating Gene Gain and Loss Rates in the Presence of Error in Genome Assembly and Annotation Using CAFE 3. Molecular Biology and Evolution. 2013;30 8:1987-97. doi:10.1093/molbev/mst100.

76. Buchfink B, Xie C and Huson DH. Fast and sensitive protein alignment using DIAMOND. Nature methods. 2015;12 1:59-60.

77. Tang H, Krishnakumar V, Li J and Zhang X. jcvi: JCVI utility libraries. Zenodo(doi: 105281/zenodo 31631). 2015.

78. Villanueva RAM and Chen ZJ. ggplot2: elegant graphics for data analysis. Taylor & Francis, 2019.

79. Quinlan AR. BEDTools: the Swiss- army tool for genome feature analysis. Current protocols in bioinformatics. 2014;47 1:11.2. 1-.2. 34.

80. Krzywinski M, Schein J, Birol I, Connors J, Gascoyne R, Horsman D, et al. Circos: an information aesthetic for comparative genomics. Genome research. 2009;19 9:1639-45.

Figure legends

Fig. 1 Images of *Capparis spinosa*. A. mature *Capparis spinosa* plant. **B.** Flowers. **C.** Fruits. **D.** Stem. **E.** the tip of leaf.

Fig. 2 High-quality assembly of 21 chromosomes. A. chromosome ideograms. **B.** Transposon element (TE) repeat sequence density (window size 100 kb). **C.** Tandem repeat sequence density (window size 100 kb). **D.** Gene density (window size of 100 kb). **E.** GC content (window size of 100 kb). **F.** Relationship between syntenic blocks.

Fig. 3 Gene Duplication and evolution of *Capparis spinosa.* A. The number of genes and gene pairs of 5 duplication types; **B.** Distribution of Ka/Ks of 5 replication types; **C.** Distribution of Ks of 5 replication types; **D.** Distribution of 4DTv of 5 replication types.

Fig. 4 Evolution of the *Capparis spinosa* genome. A. A Venn diagram of specific and shared orthologs among 16 species (*O. sativa*, *B. distachyon*, *A. comosus*, *M. acuminata*, *C. micranthum*, *N. nucifera*, *T. sinense*, *V. vinifera*, *S. lycopersicum*, *P. trichocarpa*, *T. cacao*, *C. spinosa*, *T. hassleriana*, *A. thaliana*, *N. colorata*, *A. trichopoda*), identified based on gene family cluster analysis. Each number in the diagram represents the number of gene families within a group. **B.** Expansion and contraction of gene families. **C.** GO Enrichment analysis of expanded genes.

Fig. 5 Distribution of Ks, 4DTv and ages of LTR of *Capparis spinosa* and other species. A. Ks distribution of *C. spinosa* and other representative species; **B.** 4DTv distribution of *C. spinosa* and other representative species; **C.** Ages of LTR of *C. spinosa* and other species (Molecular clock r is $7*10^{-9}$).

Figure S1 Genome size estimation of *C. spinosa* by using flow cytometry with *Solanum lycopersicum* as reference. The main peaks of *Solanum lycopersicum* and *Capparis spinosa* (sample 1 and sample 2) were 356.73 and 123.27 (mean value = (122.72 + 123.82)/2), respectively. According to the formula "peak (ref)/genome size (ref) = peak (*Capparis spinosa*)/genome size (*Capparis spinosa*)", we estimated that the mean value of genome size of *Capparis spinosa* was 276.44 Mb.

Figure S2 Hi-C interaction heat map. The 21 chromosomes Hi-C heat map.

Figure S3 Gene duplication positive selection genes enrichment analysis. A. GO enrichment analysis of positive selection genes in 4 types of replications; **B.** KEGG enrichment analysis of positive selection genes in 5 types of replications.

Figure S4 Genome collinearity analysis of the *C. spinosa* genome. A. Dot plots of paralogs in the *C. spinosa* genome; **B.** *A. trichopoda*, *C. spinosa* and *A. thaliana* gene level collinearity analysis; **C.** *T. cacao* and *C. spinosa* gene level collinearity analysis; **D.** *A. thaliana and C. spinosa* gene level collinearity analysis; **E.** *V. vinifera, C. spinosa* and *S. lycopersicum* genome level collinearity analysis.

Figure S5 *C. spinosa* specific genes GO enrichment analysis and expansion genes KEGG enrichment analysis. A. GO Enrichment analysis of *C. spinosa* specific genes (Biological Process); **B.** KEGG enrichment analysis of expansion genes. (The genes Ka/Ks >1 was select as positive selection genes).

 Click here to access/download;Figure;Figure 1.pdf

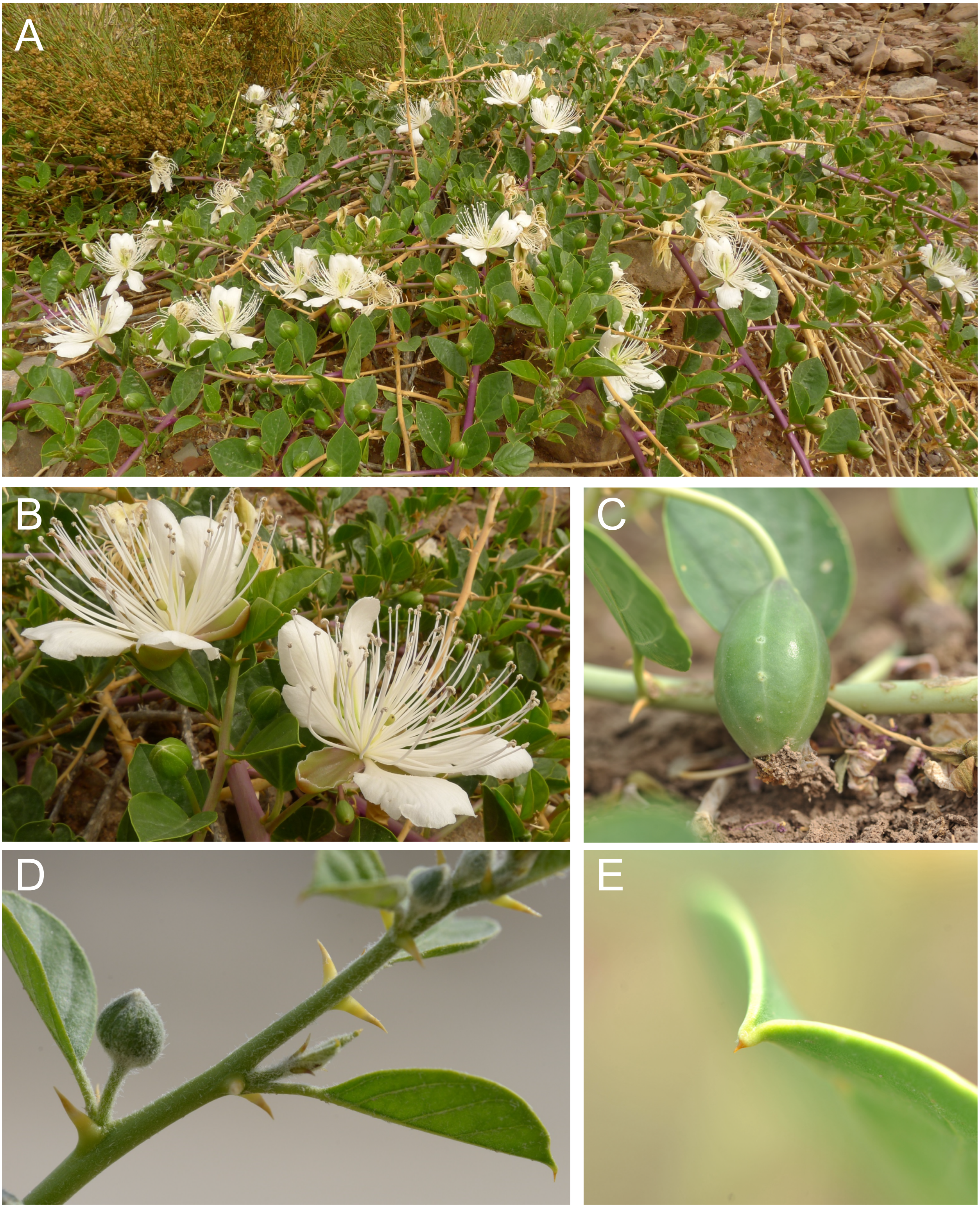

figure 2

Click here to access/download;Figure;Figure 2.pdf

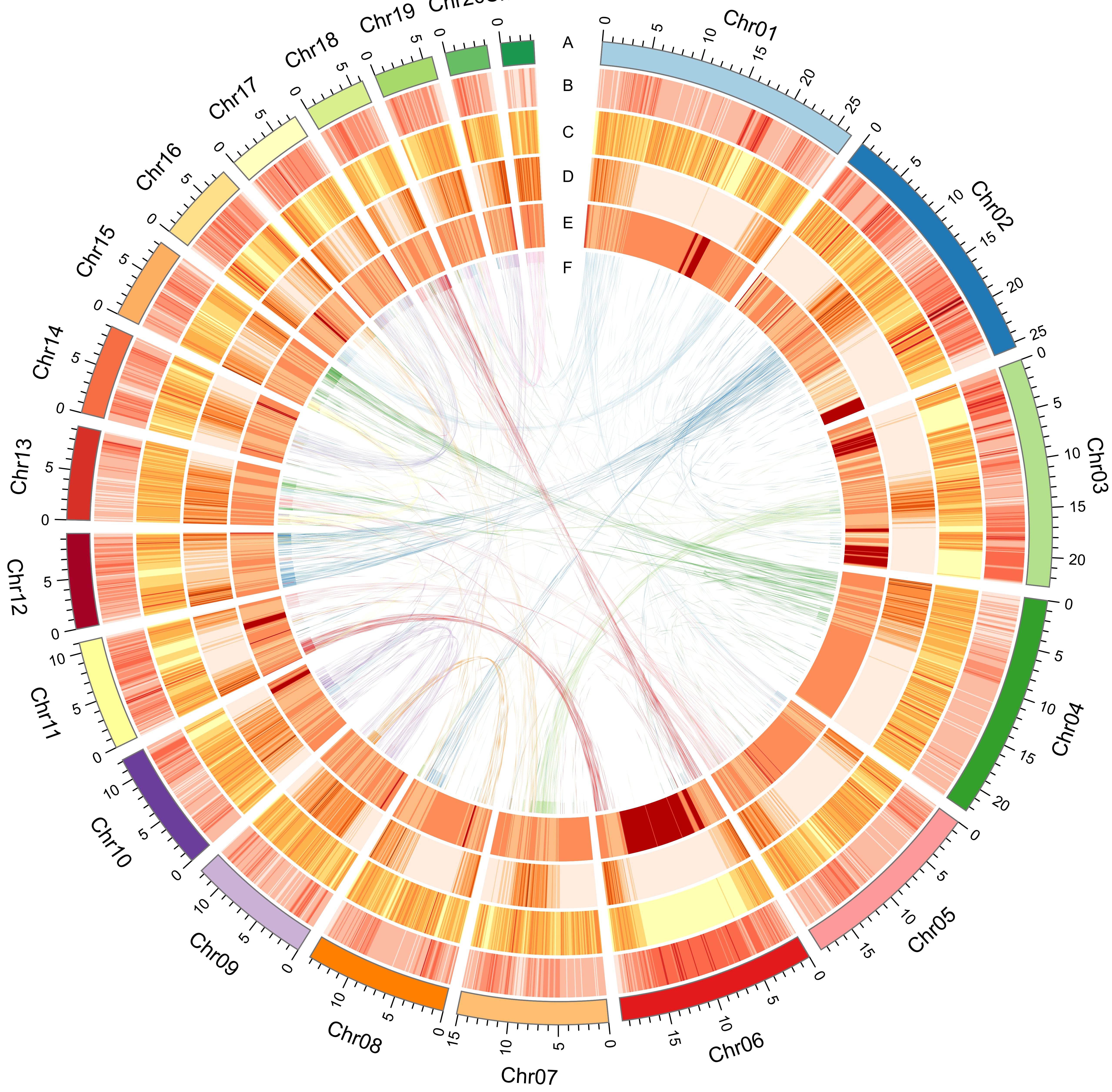

figure 3 Click here to access/download;Figure;Figure 3.pdf

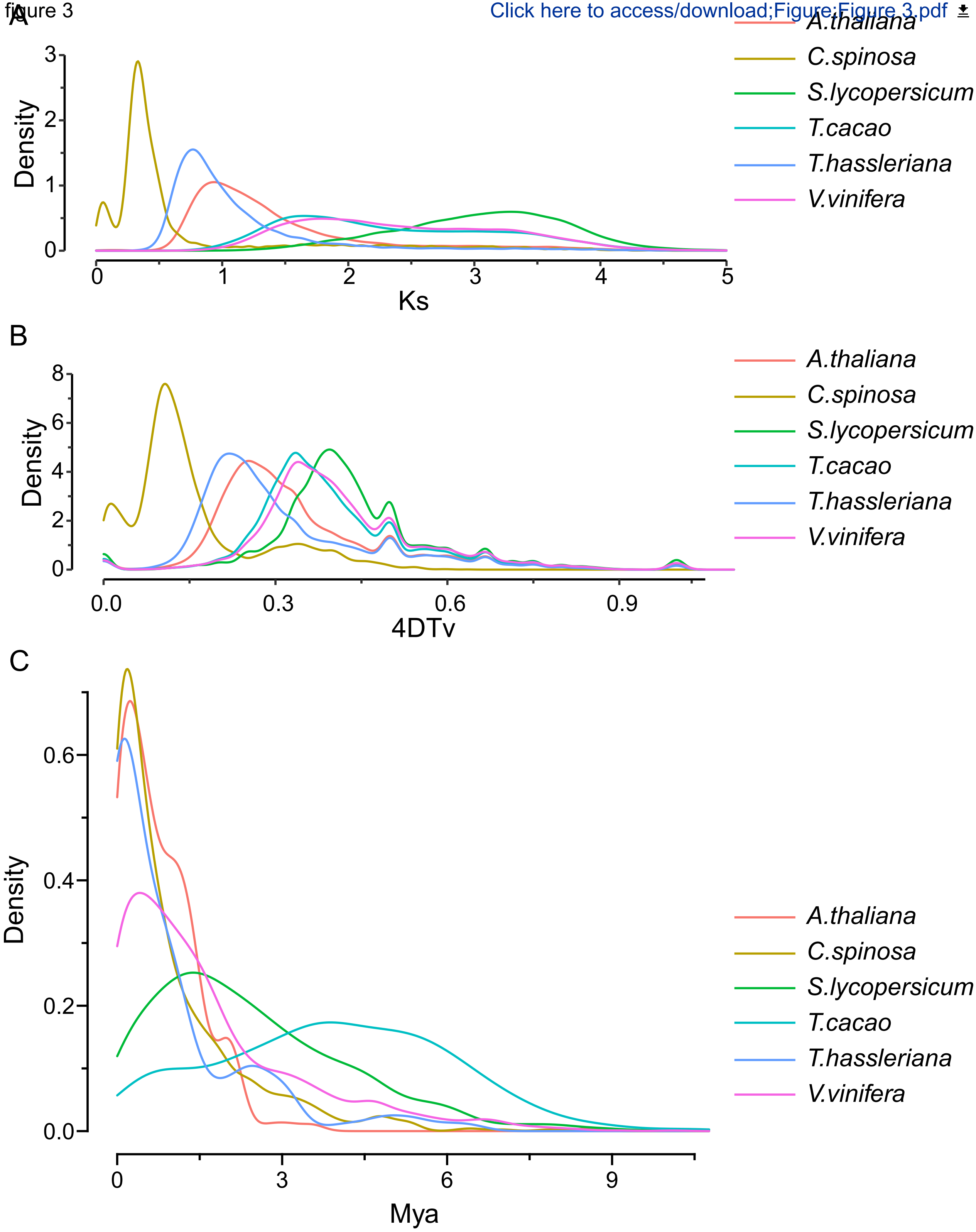

figure 4

Click here to access/download;Figure;Figure 4.pdf

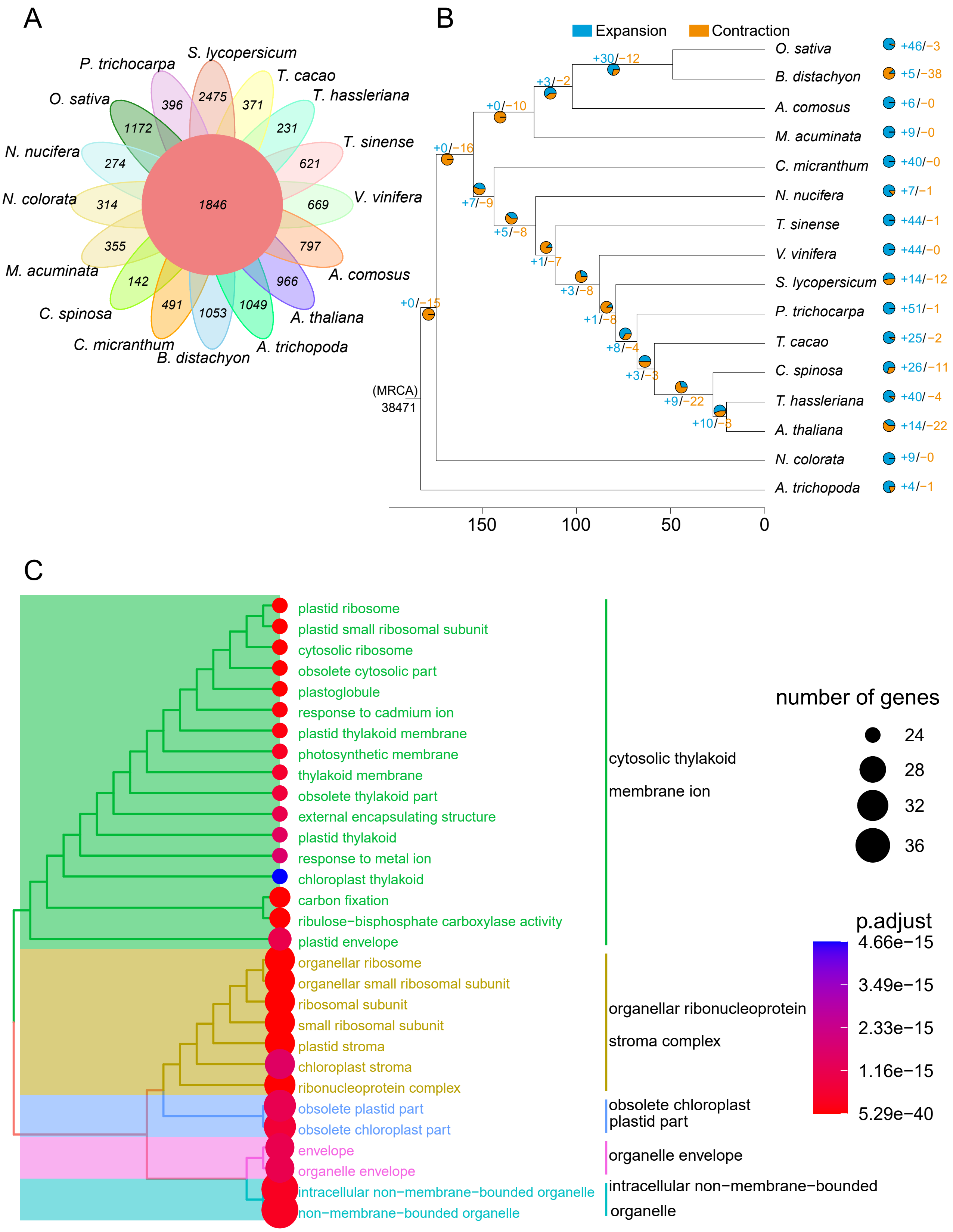

figure 5

Click here to access/download;Figure;Figure 5.pdf

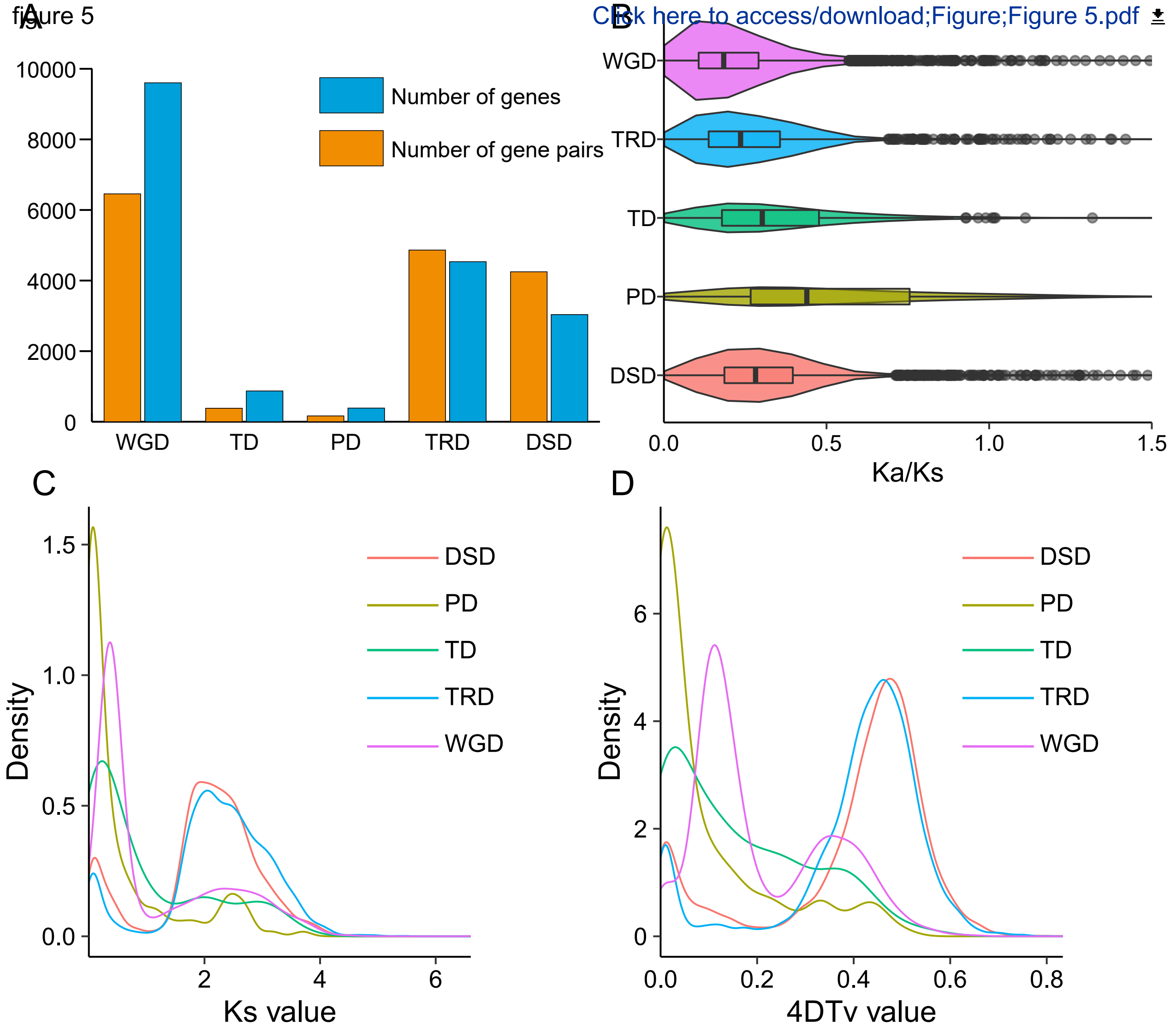

Supplymentary Tables

Click here to access/download
Supplementary Material
Supplyment Tables.docx

Supplementary Figure

Click here to access/download
Supplementary Material
Supply Figure.docx
