# Supplementary material for: The Capparis spinosa var. herbacea genome provides the first genomic instrument for a diversity and evolution study of the Capparaceae family
Source: Gigascience. 2022 Oct 30;11:giac106. doi: 10.1093/gigascience/giac106 (PMC9618406; doi:10.1093/gigascience/giac106)
Supplement: giac106_GIGA-D-22-00058_Revision_1 [file giac106_giga-d-22-00058_revision_1.pdf]

GigaScience

The Capparis spinosa genome provides insight into genome evolution of Capparaceae

--Manuscript Draft--

**Manuscript Number:**	GIGA-D-22-00058R1
**Full Title:**	The Capparis spinosa genome provides insight into genome evolution of Capparaceae
**Article Type:**	Data Note
**Funding Information:**	National Key Research and Development Program of China (2018YFE0207200) — Dr. Changyan Tian
**Abstract:**	Capparis spinosa L., one of the most economically important species of Capparaceae , is a xerophytic shrub that is well adapted to drought and has promising potential for significant adaptation to harsh environments. However, genetic studies on this species are presently limited by the lack of a reference genome.<br>We sequenced and assembled the C. spinosa genome using the combination of PacBio CCS sequencing and Hi-C data. The final assembly genome was approximately 274.53 Mb (contig N50 length of 9.36 Mb, scaffold N50 length of 15.15 Mb), 99.23% of which has been assigned to 21 pseudochromosomes. In the genome sequence, tandem repeats accounted for 19.28%, and Transposable elements sequence accounted for 43.98%. The proportion of tandem repeats in the C. spinosa genome was much higher than the average of 8.55% in plants. A total of 21,577 protein-coding genes were predicted, with 98.82% being functionally annotated. The result of species divergence times showed that C. spinosa and Tarenaya hassleriana separated from a common ancestor 27.495 MYA.<br>In summary, this study reported high-quality reference genome assemblies and genome features for Capparaceae family for the first time. The assembled C. spinosa genome might provide a system for studying the diversity, speciation, and evolution of this family, and it is an important resource for understanding the mechanism of drought and high-temperature resistance.
**Corresponding Author:**	Mao Chai<br>Chinese Academy of Agricultural Sciences Cotton Research Institute<br>Zhengzhou, Henan CHINA
**Corresponding Author Secondary Information:**	
**Corresponding Author's Institution:**	Chinese Academy of Agricultural Sciences Cotton Research Institute
**Corresponding Author's Secondary Institution:**	
**First Author:**	Lei Wang
**First Author Secondary Information:**	
**Order of Authors:**	Lei Wang
	Liqiang Fan
	Zhenyong Zhao
	Zhibin Zhang
	Li Jiang
	Mao Chai
	Changyan Tian
**Order of Authors Secondary Information:**	
**Response to Reviewers:**	Reviewer reports:<br>Reviewer #1: The manuscript titled "Capparis spinosa genome provides insight into

Powered by Editorial Manager® and ProduXion Manager® from Aries Systems Corporation

genome evolution of Capparaceae" reports the first reference genome assembled for Capparaceae family for the first time. Due to the current lacks in the genetic information about the species, this paper can be useful for future studies, however there are several gaps and unclear points in all section that the authors have to cover and modify.

Plant material

The first gap is related to the plant material sequenced. The C. spinosa species includes several subspecies and botanic varieties (such as C. spinosa subsp spinosa; C. spinosa subsp. rupestris, C. spinosa subsp spinosa var. spinosa, C. spinosa subsp spinosa var. herbacea…and more, see Fici et l. 2014, http://dx.doi.org/10.11646/phytotaxa.174.1.1). Therefore the authors must specify to which group the sequenced material belongs. Due to the complex origin of the different varieties, the generic "C. spinosa" is incorrect and has not meaning, more information is needed on the plant material used.

Reply: Thank you very much! The material samples of the assembled genome were deposited in the Herbarium of the Xinjiang Institute of Ecology and Geography, Chinese Academy of Sciences (XJBI). The samples used in this study were identified and confirmed by XJBI taxonomist Xiyong Wang. The species we sequence and assemble the genome is Capparis spinosa sub. spinosa var. herbacea. See line 333.

About the genome size evaluation, it is not clear what has been done. In figure S1, only the Capparis spinosa peaks are reported (two replicates). Usually both standard and target samples are included in the same run and the genome size is evaluated comparing the two peaks (also as reported in Doležel and Bartoš 2005, but also in Doležel et al. 1989...and more). The comparison between reference and target samples allows to have the genome size in pg, that can be directly convertible in Mb (1 pg = 978 Mb). Therefore, it is not clear how the genome size of "C. spinosa" sequenced (must to be specify what variety is) has been obtained. Please explain and add other information and references. This is another key point to evaluate the complexity of genome sequenced.

Reply: Thank you very much for your suggestion. First, flow cytometry experiment is a good method for genome size evaluation. There are two strategies for flow cytometry experiments, one as you mentioned where both standard and target samples are included in the same run, and the other is that the standard and target samples are run separately as in the articles Xie et al., 2020 (doi:10.1038/s41438-020-0328-y) so both strategies are feasible. We further read the peaks of the standard and target samples onto one graph to facilitate comparison of the two peaks to assess genome size.

Secondly, we did genome survey analysis and the results showed that the predicted size of Capparis spinosa var. herbacea genome was 245,974,629 bp, which turned out to be close to the size of the final genome assembled and proved the reliability of the genome size.

We have modified the content and images for the above results accordingly, please see line 376-390 and Fig S1.

Methods

The methods need to be improvement (see below) and revise, several sentence are pointless and unclear. In addition there is a lack in the annotation. Indeed, since the present paper is the first genome assembled in the Capparaceae family, the authors must to add the RNASeq data from different tissues (such as root, leaves, flowers, buds etc etc) to develop a correct and full annotation (this is mandatory).

Thank you very much! We performed second-generation Illumina and Pacbio third-generation transcriptome sequencing on mixed samples of roots, stems, leaves, flowers, and fruits for genome annotation. The relevant raw data have been uploaded to the NCBI SRA database, and the corresponding numbers are SRR18512706 and SRR18512705. At the same time, the relevant material information and transcriptome library and sequencing information are supplemented in line 343-346

Please add information on the genome size estimation protocols and methods.

There are not information about DNA extraction protocols used.

Thank you very much! We have added methods for genome size estimation, in line 376-390

We have supplemented the DNA extraction method at line 340-343.

The paragraph describing the PacBio library construction and sequencing is very short, providing only few information on the assembling process and on the pre-processing of reads. Please improve it, adding also information about the PacBio platform used.

Thank you very much! We have supplemented PacBio library construction information and added information on sequencing platforms in line 350-362 and Table 1.

	The authors wrote "accounting for 100-fold coverage of the draft genome of C. spinosa.", what does it mean? I would think that this coverage was evaluated using the estimated genome size and not the draft genome that was not available in the sequencing phase.<br>Thank you very much! Yes, you are right. Here we use the genome size estimated from the genome survey to evaluate. We have revised the relevant representation in line 403-404 in the manuscript.<br>The authors wrote "Hi-C fragment libraries were constructed as reported by Fu et al. [37]"…please provide more detailed reference for the protocols used and add more details on the methods.<br>Thank you very much! We have revised the relevant representation in line 392-401.<br>The authors wrote "using LACHESIS [38] with the following parameters:…. C-LUSTER_MIN_RE_SITES=9;". Parameters here used are slightly different from the reference cited. Please, modify the sentences and add details on the parameters selection step.<br>Thank you very much! we have improved Hi-c related information in line 392-401.<br>"Clean Hi-C reads, accounting for 100-fold coverage of the draft genome of C. spinosa, and the final 28 scaffolds were anchored to chromosomes, accounting for 99.98% of the total length.". What does it mean? The sentence is unclear, please rephrase it.<br>Thank you very much! Here we use the genome size estimated from the genome survey to evaluate. We have revised the relevant representation in line 377-381.<br><br>In the Genome assembly and evaluation section the authors wrote "Align the short sequence obtained by Illumina HiSeq with the reference genome using the default parameters of the BWA-MEM V0.7.17 [41]. CEGMA V2.5 (default parameters) [42] database contains 458 conserved core genes in eukaryotes were used to evaluate the completeness of the final genome assembly. ". These sentence are very confusing, rewrite please.<br>Thank you very much! We have revised this sentence in line 415-419.<br>Repeat sequences identify: Rephrase the title, maybe Repeat sequences detection? Identification? Masking?<br>Thank you very much! We have modified it to "Identification of repeat sequences". See in line 430.<br>The authors wrote "By combining the above de novo TE sequence library and LTR-RTs library with the known Repbase V19.06, REXdb V3.0, and Dfam V3.2 databases, a non-redundant species-specific TE library was constructed" - This part is unclear. How did you combine the libraries? What do you mean with non-redundant species-specific?<br>We have revised the presentation here. Merging multiple databases is done using the shell "cat" command. Since predictions from multiple databases may be duplicated, redundancy needs to be removed. Redundancy is removed using seqkit software.see line 433-438.<br>In the Gene prediction and annotation the authors wrote "Three approaches, de novo prediction, homology search, and transcript-based assembly to annotate protein-coding genes, as described by Fu et al.[37]." - According to your statement, you used transcript-based annotation, but there is no mention to RNASeq sequencing in the paper (see my first and mandatory comment for the methods section). You once again cite Fu et al. 2021 where they explicitly state they used RNASeq for the prediction of genetic structure. Furthermore, I am not sure of your use of the HISAT2 tool in this context. Please explain.<br>We have added transcriptome-related information. Includes materials, library construction, and sequencing information. We did use HISAT2 for transcriptome analysis.<br>"Other software use default parameters." - Please be consistent, in other paragraphs you used the "(default parameters)" for all tools.<br>We have revised here to detail the software and methods used. See line 444-454.<br>"rRNA prediction was mainly based on Rfam V12.0 [60] database and Barrnap V0.9 [61] was predicted" - Please rewrite this sentence.<br>We have modified it. See line 459-464.<br>"GenDup_finder-unique the stricter version of". Please rephrase this sentence in the Whole-genome gene duplication analysis<br>We have revised it. See line 466-468.<br>"GO and KEGG enrichment analysis by clusterProfiler V4.2.0 [66]". What universe did you use for the enrichment analysis? This may heavily affect the enrichment.

Genes with Ka/Ks>1 in five replication modes were used for GO and KEGG enrichment analysis, respectively, and the results were visualized in one figure. We have revised it. See line 471-472.
"The encoding genes from a species were clustered into six groups, including 0 copies, 1 copy (single-copy), 2 copies, 3 copies, 4 copies, and 4+ copies. A total of 306 genes were identified as single-copy genes. The obtained gene families were annotated using the PANTHER V15 database [68]." - this section is unclear. Did you follow a pipeline already published?
Orthofinder can perform these analyses, classifying genes into the above 6 types. This is to illustrate the source of the single copy genes that follow. We used the same analytical sequencing as Fu et al. (https://doi.org/10.1038/s41438-021-00487-1), whether in genome assembly, annotation, or comparative genomics. Only some software types and versions used are slightly different. Therefore, we cite Fu's literature in many places in the text.
"Use MAFFT V7.205 [69] to align each single-copy gene family sequence (parameter: --localpair - maxiterate 1000), and then use Gblocks V0. 91b [70] (parameter: -b5=h) filter the conserved sites, and finally all aligned genes of each species the family sequence was connected end to end to get the supergene, and then use IQ-TREE V1.6.11 [71] model detection tool ModelFinder [72] for model detection, the best model obtained was JTT+F+I+G4, and then using this best model, the evolutionary tree was constructed by the maximum likelihood (ML) method, and the number of bootstrap was set to 1000." - This sentence is too long and should be split. Furthermore, the methodology is not clear and confusing.
We have modified it in line 485-492.
"with A. trichopoda as the outgroup of the root tree" - How did you choose the outgroup? Add reference.
We have revised it in line 494.
"divergence times were estimated as follows: A. trichopoda Vs S. lycopersicum at 164-194 MYA, O. sativa Vs B. distachyon at 42-60 MYA, A. comosus Vs O. sativa at 94-115 MYA, N. nucifera Vs V. vinifera at 116-127 MYA". How did you estimate these? And how did you use this information in the divergence time estimation? This part is confusing and should be rephrased.
We have rewritten this part of the statement in line 494-499.
Results
The PacBio sequencing allowed a fairly good scaffolding with an N50> of 15 Mb, and Hi-ci helped in the cleaning of PacBio sequences, notoriously know as long but low quality sequences….this point should be highlighted by the authors. However, although the Illumina Hi-C sequencing favored the anchoring of the scaffolds in the pseudo-chromosomes, the lack of genetic maps leaves the anchor a bit weak.
Combined integration of optical mapping and Dovetail Genomics data could further advance the contiguity supporting the idea that optical mapping and Hi-C data are complementary and can help bridging different regions in the genome. (Wen-Biao Jiao ,et al. https://doi.org/10.1101/gr.213652.116 ;https://www.sciencedirect.com/science/article/pii/S1369526616301315)
In general the Genome sequencing and assembly paragraph is brief and provide only few information on the assembly results.
"The Hi-C reads were employed for scaffolding into 21 pseudo-chromosomes (Fig. 2, Fig. S2)" - Again, no info on how this was done. Furthermore, there is no image of the hi-c contact map generated.
We supplement Hi-c with more details in Methods in line 372-374. Hi-c contact map in Fig S2.
"Gene completeness reached up to 99.98%" - How did you evaluate this? Did you mean genome completeness?
Yes, you are right. We have revised it in line 100.
The genome quality assessment part is in the next part of the paragraph.
"indicating that the assembly quality of C. spinosa reached the level of the reference genome." What do you mean? Which reference genome?
Thank you very much! We have added detailed references and modified the description line 110-113.
In Identification of genomic repetitive sequences section the authors wrote "We found the correlation between the distribution of TR sequence and GC content on the chromosome in the C. spinosa genome (Fig.2 C, E). So, use Spearman's algorithm to calculate the correlation. The correlation coefficient is -0.52, and the P-value is 2.2e-16. It shows that the distribution of TR sequences in the C. spinosa genome and the

Powered by Editorial Manager® and ProduXion Manager® from Aries Systems Corporation

GC content of the sequences show a significant negative correlation." There is no mention in the methods. Please add a description of the algorithm used for this task. Furthermore, the sentence is confusing and should be rephrased.
In fact, we performed a pairwise correlation analysis on the GC content of the genome, gene density, TE distribution, and TR distribution. In the end, only the correlation between GC content and TR distribution was found, and no correlation was found between others. We use a function `cor` of basic R to calculate correlation. Cor provides three methods to calculate correlation, namely "pearson", "kendall", "spearman". We normalize the data. The distribution test does not conform to the normal distribution , so pearson cannot be used. After testing the latter two, the spearman method is finally selected. We have added the description of correlations to the Methods. See in line 128-138 and 529-531.
As already stated in the methods section, the Genome annotation pipeline is lacking clarity. The same can be said for this section of the results. As reported in methods section, the authors must to sequence RNASeq libraries developed from different tissues, in order to have a correct annotation.
"and more than 98.82% of genes were annotated based on sequence similarity using the following protein-related databases". Here you give a brief description of the blast results, but in the methods you state that you used a much more complex method for the final annotation. Where are the results? You should expand this paragraph, adding more information/details.
"Table 1." - This table should be moved to the genome assembly paragraph and a new table with annotation info should be produced (also as supplementary material).
Thank you for your suggestion. We adjusted the position of the Table1, and added a new table, Table S4, for the prediction statistics of genomic genes. At the same time, we revised the relevant sentences in the manuscript line 140-143.
The authors spoke about possible haplotypes in the duplication analysis, but the analysis is really weak. The authors have to improve this section using other instrument/comparing adding other information, such as the degree of heterozygosity.
In the same way, the evolutionary study is also weak. The authors have to improve it adding other comparing.
We have analyzed heterozygosity using genome survey. We have rewritten the results. See in line 202-226.

"a total of 19,063 colinear gene pairs on 409 colinear blocks were inferred between C. spinosa and A.thaliana. 5,501 colinear gene pairs from 17 colinear blocks detected between C. spinosa and A. trichopoda (Fig. S4B). 16,747 colinear gene pairs from 260 colinear blocks were detected between C. spinosa and T. cacao. 15,352 colinear gene pairs from 222 colinear blocks detected between C. spinosa and V. vinifera, and 17,940 colinear gene pairs from 502 colinear blocks were detected between C. spinosa and S. lycopersicum (Fig. S4E)". This could be summed up in a table to reduce the repetitive text.
We modify this content and organize the above information into Table S7. See in lines 189-193
"Next, the species divergence times of them were evaluated (Fig. 4B)"…please rewrite.
"Results showed that in almost species, except B. distachyon and A. thaliana, more gene families experienced expansion than contraction.". Please rephrase, and improve as suggested in a previous point.
In summary the authors have to improve all result sections (in the current form are really weak), adding other information through RNASeq experiment for the annotation.
We have added related experiments and descriptions. Please see the reply above.
Discussion
The discussion introduces a high number of genes and pathways that have not been listed/highlighted in the results. This is the consequence of the results chapter that is too short, unclear, and superficial. The improvement of the results section may aid the discussion section. Furthermore, the discussion lacks of a final conclusion chapter.
Reply: We have added the corresponding content in the results section and a conclusion section at the end of the article. Please see lines 325-330.
"The genome assembly was 274.53 Mb in length, and >99.23% of the assembled genome was placed on 21 chromosomes. This represents a more contiguous and higher-quality genome assembly than that of recently sequenced Brassicaceae species genomes, such as eight oilseed rape lines [20], with a contig N50 2.1~3.1 Mb, as well as field pennycress [21]". This sentence seems pretentious. The works cited shows higher Busco score and completeness percentage (98% and 95.9%

respectively) and a three-fold size genome assembly. Furthermore the scaffold N50 of both works cited is significantly higher (~50 and ~70 Mb) then that reported for this assembly ~15 Mb.
Reply: Thank you very much for your suggestion. We have improved this section. Please see lines 261-266.
"At the same time, we also found that the distribution of TR in the genome of C. spinosa is inversely proportional to the content of GC. The local high GC content in the genome seriously affects the accuracy of Hi-C assembly. For example, the GC content of C. spinosa genome Chr06: 3700000-15800000 is 53.92%, which is much higher than the genome GC content 36.61%. High GC content may affect the accuracy of assembly of this segment on Chr06 chromosome. It may also be due to the high local GC content and high TR ratio that increase the difficulty of genome assembly." - This part is full of repetitions and could be improved.
Reply: We have improved this section and merged it with the first paragraph in order to achieve a more appropriate description. Please see lines 267-288.
Finally the paper needs a full language revision from a native English speaker.
We use professional editing companies (TopEdit www.topeditsci.com) to polish the language of the article.

Reviewer #2: I am extremely excited and pleased to finally see a Capparaceae genome assembly of high quality. Overall, I find this a straightforward report of a genome sequencing project with a few nuggets of interesting biology included (such as the high rate of Tandem repeats and their negative correlations with GC content). However, I have a few major concerns/questions for the authors as well as a few minor suggestions for improvement.
Major questions:
1. There is almost no discussion about the past domestication history of Capparis spinosa. If this was done, then likely the authors would report that C. spinosa is thought to be of hybrid origin between two between C. orientalis and C. sicula (e.g. Rivera et al., 2002). The authors present no information on the degree of potential heterozygosity in their assembly and if for example som time of purge haplotypes steps was done. I would very much like to see some discussion about domestication and thus how past hybridisation was dealt with (this should be much more recent than the reported WGD).
Thank you very much ! The samples used in this study were identified and confirmed by Xinjiang Institute of Ecology and Geography, Chinese Academy of Sciences (XJBI) taxonomist Xiyong Wang. We performed genome surveys using illumina sequencing, and the estimated heterozygosity rate was 0.878%. A previous study indicated that C. spinosa is morphologically related to C. sicula Duhamel as well to C. orientalis Duhamel and overlaps with the latter (Inocencio et al., 2005). Recently a taxonomic revision has been conducted by Fici (2014, 2015) on the C. spinosa group widespread from the Mediterranean to central Asia. C. spinosa is recognized as a single species and is represented by four subspecies (i.e., C. spinosa subsp. spinosa; C. spinosa subsp. rupestris; C. spinosa subsp. cordifolia; C. spinosa subsp. himalayensis). C. spinosa subsp. spinosa is widely distributed eastwards from the Mediterranean to China and Nepal, showing inherited traits and great level of heterogeneity. Within this subspecies, some varieties are identified, namely var. herbacea and var. atlantica. (Stephanie Chedraoui et al., https://doi.org/10.3389/fpls.2017.01845). See in line 333 and 51-57.
2. The positioning of the WGD and the dating of divergence times is a bit confusing. There is quite some past work on the dating of say the At-alpha WGD in Brassicaceae and also the divergence of Brassicaceae from Cleomaceae and Capparaceae (e.g. see work for example of Jocelyn Hall). Also, placing the capparaceae duplication in light of Cleomaceae duplications (e.g. paper by Marbry et al 2021). Also, the "younger age" maybe due to Capparis being woody and most other compared species are herbaceous (except Grape). This is issue is known to cause dating problems and is well discussed in the literature.
Reply: Thank you very much for your suggestion. The complicated relationship between the three closely related families, Capparaceae, Cleomaceae, and Brassicaceae, has been extensively studied since the appearance of the theory of alternation of generation by Hofmeister (zayat et al., 2020). However the taxonomic relationships of these three families are still under discussion and much controversy exists, especially in different regions (zayat et al., 2020; Ahmadi, M and Saeidi, H, 2018). We believe that it is not rigorous to discuss the differentiation of the three

Powered by Editorial Manager® and ProduXion Manager® from Aries Systems Corporation

	families Capparaceae, Cleomaceae and Brassicaceae based only on the genomic information of C. spinosa, and it is more appropriate to analyze and discuss them after obtaining more genomic information of species in the family Capparaceae. At the same time when comparing the WGD of species, we are selecting species according to the evolutionary tree. And there are both woody and herbaceous, not only Grape, Theobroma cacao and Amborella trichopoda are also woody. We assembled species is Capparis spinosa sub. spinosa var. herbacea. And the evolutionary tree also shows that our species is relatively close to the herb Arabidopsis thaliana and Tarenaya hassleriana. We rewrote the content of WGD in line 183-219.<br>Ref<br>El zayat, M.A.S., Ali, M.E.S. & Amar, M.H. A systematic revision of Capparaceae and Cleomaceae in Egypt: an evaluation of the generic delimitations of Capparis and Cleome using ecological and genetic diversity. J Genet Eng Biotechnol 18, 58 (2020). https://doi.org/10.1186/s43141-020-00069-z<br>Ahmadi, M., Saeidi, H. Genetic diversity and structure of Capparis spinosa L. in Iran as revealed by ISSR markers. Physiol Mol Biol Plants 24, 483–491 (2018). https://doi.org/10.1007/s12298-018-0518-3<br>Fici S . A taxonomic revision of the Capparis spinosa group (Capparaceae) from the Mediterranean to Central Asia[J]. PHYTOTAXA, 2014, 2014,174(1)(-):1-24.<br><br>3. Ideally the seed material or at a minimum a herbarium sample of the genotype used for sequencing should be made clear. Can others access this material in some way? Also, having a line from a botanic garden means that plant came from somewhere else (e.g. in the Mediterranean basin). Where is this plant originally from? Also, there is a chance (see point 1) that this plant is NOT C. spinosa but another non-hybrid Capparis. Please have a skilled taxonomist check the plant and deposit a herbarium specimen.<br>The material samples of the assembled genome were deposited in the Herbarium of the Xinjiang Institute of Ecology and Geography, Chinese Academy of Sciences (XJBI). You can contact Lei Wang (The first author Email: egiwang@ms.xjb.ac.cn) for the original material.<br>Minor suggestions:<br>1. Do not use italics with family name: Capparaceae<br>Thank you very much! We have modified it.<br>2. When starting a sentence, don't say C. spinosa...then Capparis spinosa (e..g. line 50)<br>Thank you very much! We have modified it.<br>3. line 68: Change "Total..." to "A total..."<br>Thank you very much! We have modified it.<br>4. Line 114...this line "Gene footprints in the duplicate events were observed. " Is confusing to me (footprints in sequences often mean something else)...I suggest you just delete it.<br>Thank you very much! We have removed it in line 151.<br>5. You mention some chemicals used/present in Capparis, but never glucosinolates! This is the character that gives capers their flavour and make them clearly part of Brassicales.<br>Reply: Added. Please see lines 63-65.
**Additional Information:**	
**Question**	**Response**
Are you submitting this manuscript to a special series or article collection?	No
**Experimental design and statistics**<br><br>Full details of the experimental design and statistical methods used should be given in the Methods section, as detailed in our Minimum Standards Reporting Checklist. Information essential to interpreting the data presented should be made available	Yes

in the figure legends.<br><br>Have you included all the information requested in your manuscript?	
**Resources**<br><br>A description of all resources used, including antibodies, cell lines, animals and software tools, with enough information to allow them to be uniquely identified, should be included in the Methods section. Authors are strongly encouraged to cite Research Resource Identifiers (RRIDs) for antibodies, model organisms and tools, where possible.<br><br>Have you included the information requested as detailed in our Minimum Standards Reporting Checklist?	Yes
**Availability of data and materials**<br><br>All datasets and code on which the conclusions of the paper rely must be either included in your submission or deposited in publicly available repositories (where available and ethically appropriate), referencing such data using a unique identifier in the references and in the "Availability of Data and Materials" section of your manuscript.<br><br>Have you have met the above requirement as detailed in our Minimum Standards Reporting Checklist?	Yes

Manuscript
Click here to access/download;Manuscript;manuscript_clean_version.docx

The *Capparis spinosa* genome provides insight into Capparaceae genome evolution

Lei Wang[a,b,1], Liqiang Fan[c,d,1], Zhenyong Zhao[a,b], Zhibin Zhang[c,d], Li Jiang[a,b], Mao Chai[c,d,*] and Changyan Tian[a,b,*]

[1] These authors contributed equally to this work.

[*] Corresponding authors.

Email addresses: chaimol@163.com (M. Chai); tianchy@ms.xjb.ac.cn (C. Tian)

[a] State Key Laboratory of Desert and Oasis Ecology, Xinjiang Institute of Ecology and Geography, Chinese Academy of Sciences, Urumqi 830011, China

[b] University of Chinese Academy of Sciences, Beijing 100049, China

[c] Institute of Cotton Research of the Chinese Academy of Agricultural Sciences, Anyang, Henan 455000, China

[d] Zhengzhou Research Base, State Key Laboratory of Cotton Biology, Zhengzhou University, Zhengzhou 450000, China

Abstract

Background

Capparis spinosa L., one of the most economically important species of Capparaceae, is a xerophytic shrub that is well adapted to drought and harsh environments. However, genetic studies on this species are limited because of the lack of its reference genome.

Findings

We sequenced and assembled the *Capparis spinosa* subsp. *spinosa* var. *herbacea* genome using data obtained from the combination of PacBio circular consensus sequencing and high-throughput chromosome conformation capture. The final genome assembly was approximately 274.53 Mb (contig N50 length of 9.36 Mb, scaffold N50 of 15.15 Mb), 99.23% of which was assigned to 21 chromosomes. In the whole-genome sequence, tandem repeats accounted for 19.28%, and transposable element sequences accounted for 43.98%. The proportion of tandem repeats in the *C. spinosa* genome was much higher than the average of 8.55% in plant genomes. A total of 21,577 protein-coding genes were predicted, with 98.82% being functionally annotated. The result of species divergence times showed that *C. spinosa* and *Tarenaya hassleriana* separated from a common ancestor 27.495 MYA.

Conclusions

This study reported a high-quality reference genome assembly and genome features for the Capparaceae family. The assembled *C. spinosa* genome might provide a system for studying the diversity, speciation, and evolution of this family, and serve as an important resource for understanding the mechanism of drought and high-temperature resistance.

Issue Section: Data Note

Keywords: *Capparis spinosa*; genome assembly; population evolution

Background

Capparis spinosa L., one of the most economically important species of Capparaceae, is a perennial winter deciduous shrub with a wide range, typically growing in the Mediterranean countries and distributed in Iran, Iraq, Saudi Arabia, and China [1-3]. In China, it is mainly found in Xinjiang, Gansu, and Tibet regions [4]. The *C. spinosa* family Capparaceae from the Mediterranean to Central Asia has been taxonomically revised recently [5]. *C. spinosa* is considered a single species, represented by four subspecies—*C. spinosa* subsp. *spinosa*, *C. spinosa* subsp. *rupestris*, *C. spinosa* subsp. *cordifolia*, and *C. spinosa* subsp. *himalayensis*. *C. spinosa* subsp. *spinosa* is widely distributed from the east Mediterranean to China and Nepal and possesses a high degree of heterogeneity in different genetic traits. Within *C. spinosa* subsp. *spinosa* subspecies, some varieties are identified namely var. *herbacea* and var. *atlantica*. [6].

As a drought-tolerant crop, *C. spinosa* has an extensive root system and a remarkably high root-to-shoot ratio and thus has a strong ability to find and absorb water from the environment (especially deep in the soil), resulting in significant adaptation to harsh environments [7, 8] (Fig. 1). Besides the roots, other parts of *C. spinosa*, including leaves, buds, fruits, bark, and seeds, contain a variety of bioactive compounds, such as flavonoids, phenolics, alkaloids, glucosinolates, and vitamins that have long been used in the treatment of headaches, toothaches, and kidney disease, and play a role in preventing disease and reducing the risk of carcinogenesis [9-15]. For example, methanolic extracts prepared from the fruits and flower buds of *C. spinosa* have some anti-inflammatory and anti-thrombotic effects [16]. *Cappari. spinosa* has a huge agricultural potential because of its medicinal properties and its ability to grow under drought conditions. Thus far, only a few chloroplast genomes [17-21], mitochondrial genomes [22], and SSR sequences [23] of *Capparis* have been reported, and the taxonomy of the genus is still confusing, and the lack of genomic information hinders the genetic improvement and effective use of caper plants. Here, we report a high-quality whole-genome sequence of *C. spinosa* using PacBio HiFi sequencing and high-throughput chromosome conformation capture (Hi-C) technology. Detailed information on the *C. spinosa* genome can help elucidate the biogeography and evolution of *Capparis* plants, contribute to the understanding of the molecular basis of its resistance to stress and validate its medicinal uses.

Analysis

Genome size estimation

We used a single plant of *Capparis spinosa* var. *herbacea* that was collected from the Xinjiang Institute of Ecology and Geography Chinese Academy of Sciences for whole-genome sequencing. A total of 33.08 G genomic short-read data were obtained for the genome survey (Table 1). We generated the 17-mer distribution of sequencing reads from short libraries using the k-mer method. The estimated genome size was about 245.97 Mb, and the proportion of repeat sequences and the genome heterozygosity rate were determined to be approximately 49.5% and 0.878%, respectively (Fig. S1A). The flow cytometry [24] analysis result was 276.44 Mb (Fig. S1B).

Table 1. Sequencing data used for *Capparis spinosa* genome assembly and annotation.

Sequencing type	Application	Sequencing platform	Bases (Gb)	Reads
Genome short reads	Genome survey and assessment	Illumina NovaSeq 6000	33.08	221078842
Genome long reads	Contig assembly	PacBio Sequel II	25.46	1531982
Hi-C reads	Chromosome construction	Illumina NovaSeq 6000	30.64	204744634
Transcriptome long reads	Genome annotation	PacBio Sequel II	1.52	413148
Transcriptome short reads	Genome annotation	Illumina NovaSeq 6000	11.31	75789484

Genome sequencing and assembly

In this study, PacBio circular consensus sequencing (CCS) long reads and Hi-C reads were used for *C. spinosa* genome sequencing and assembly. A total of 25.46 Gb PacBio clean long reads with an average read length of 16,618 bp were generated for genome assembly, and 30.64 Gb Hi-C data were generated for auxiliary genome assembly (Table 1, Table S1). The primary contigs were assembled with PacBio CCS reads, and a 274.53-Mb genome assembly version was generated with contig N50 of 11.04 Mb (Table S1). Hi-C reads were used to generate chromosome-level assembly of the genome (Fig. 2, Fig. S2). The final genome assembly of *C. spinosa* was 274.53 Mb, consisting of 59 contigs and 29 scaffolds. Genome completeness reached up to 99.98%. The contig N50 was 9.36 Mb and the longest contig was 22.51 Mb, while the scaffold N50 was 15.15 Mb and the longest scaffold was 26.66 Mb (Table 2).

For genome quality assessment, BUSCO analysis of the final scaffold assembly showed that 96.80% complete BUSCO genes (92.80% complete and single-copy BUSCO genes, and 4.00% complete and duplicated BUSCO genes) were identified (Table S2). Merqury revealed a consensus quality value (QV) of 28.27 and assembly accuracy of 99.85%. Core Eukaryotic Genes Mapping Approach (CEGMA) was used to evaluate the completeness of the final genome assembly, and 98.03% of the CEGMA genes were present in the genome. A total of 98.52% short sequences were successfully aligned to the genome. The genome LAI value was 17.19 of the genome assembly. A LAI value greater than 10 and less than 20 indicates that the assembly quality has reached the reference genome level [25]. Thus, these results demonstrate the high quality and completeness of the *C. spinosa* genome assembly.

Table 2. Assembly statistics of the *C. spinosa* genome.

Category	Numbers	N50 (Mb)	Longest (Mb)	Size (Mb)	Percentage of assembly
Contigs	59	9.36	22.51	274.53	100

Scaffold	29	15.15	26.66	274.53	100
Anchored	28	15.15	26.66	274.49	99.98
Anchored and oriented	21	15.15	26.66	272.43	99.23
Gene annotated	21,577	NA	NA	64.26	23.42
Repeat sequence	NA	NA	NA	173.60	63.23

Identification of genomic repetitive sequences

Moreover, 120,748,115 bp (nearly half of the assembled genome length (43.98%)) of transposable element (TE) repetitive sequences in the genome assembly of *C. spinosa* were identified by both homology-based and *de novo* methods (Table S3). Retroelement elements constituted the predominant repeat type, accounting for 31.24% of the genome length. The long terminal repeat (LTR) superfamily elements Copia and DNA TEs constituted 29,749,806 and 34,990,312 bp, corresponding to 10.84% and 12.75% of the genome length, respectively. LTR superfamily elements Gypsy and CACTA constituted 11,447,091 and 7,034,814 bp, accounting for 4.17% and 2.56% of the genome length, respectively. The density of Copia elements decreased with the increasing density of genes, whereas the DNA TEs were distributed more evenly across the genome and showed no obvious patterns or relationships with the distribution of genes (Fig. 2).

The total length of the identified tandem repeats (TRs) was 52,920,691 bp, accounting for 19.28% of the total length of the genome. The total length of microsatellites (1–9 bp units) was 43,481,890 bp (15.84%), the total length of minisatellites (10–99 bp units) was 7,039,326 bp (2.56%), and the total length of satellites (≥100 bp units) was 2,399,475 bp (0.87%).

On analyzing the genome distribution features, we found a correlation between the distribution of TR sequences and GC content of the chromosomes of the *C. spinosa* genome (Fig. 2C, E; Fig. S3A).

Spearman rank correlation was used to determine the correlation, and the correlation coefficient was −0.52 and the *P*-value was 2.2e-16 (Fig. S3B), showing a negative correlation between the distribution of TR sequences in the *C. spinosa* genome and the GC content of the sequences.

Genome coding gene prediction and annotation

A total of 11.31 Gb transcriptome short reads and 1.52 Gb transcriptome long reads were used for gene prediction (Table 1). Combining the results by the three methods, 21,577 protein-coding genes were predicted (Table 2, Table S4). Over 98.82% of the protein-coding genes were annotated for gene function using the following databases: GO (84.53%), KEGG (76.57%), KOG (59.33%), TrEMBL (98.63%), Pfam (87.75%), Swiss-Prot (84.76%), eggNOG (87.90%), and Nr (98.69%) (Table S5), indicating that gene predictions were accurate.

Dynamic changes of duplicated genes

Duplicated genes were classified into five categories, whole-genome duplication (WGD), tandem duplication (TD), proximal duplication (PD), transposed duplication (TRD), and dispersed duplication (DSD) (Fig. 3A, Table S6). Of the 21,577 genes, 18,432 were identified as duplicated genes, including 9,603 derived from WGD (52.1%), 872 from TD (4.7%), 387 from PD (2.1%), 4,534 from TRD (24.6%), and 3,036 from DSD (16.5%). Ka (number of nonsynonymous substitutions per nonsynonymous site), Ks (number of synonymous substitutions per synonymous site), 4DTv (fourfold degenerate synonymous site), and the Ka/Ks ratio were calculated for the different duplication types. Among the five duplication types, the proportion of gene pairs with Ka/Ks >1 in *Arabidopsis thaliana* was PD (5.1%), TD (3.3%), DSD (0.6%), TRD (0.3%), and WGD (0.0%). However, the corresponding ratios in *C. spinosa* were PD

(13.7%), TD (4.9%), DSD (1.3%), TRD (0.9%), and WGD (1%). PD and TD genes had qualitatively higher Ka/Ks ratios than genes derived from the other duplication types (Fig. 3B). PD with Ka/Ks >1 in *C. spinosa* (13.7%) was significantly higher than that of *A. thaliana* (5.1%). The density distribution of Ks and 4DTv showed that all five duplication types of *C. spinosa* experienced two duplications (Fig. 3C, D). However, the five duplication types had different times when duplication occurred. PD experienced a duplication at the recent 3.89 MYA (Ks peak at 0.069, 4DTv peak at 0.013). This also explains the high proportion of positive selection in PD.

GO and KEGG enrichment analysis was performed on the Ka/Ks >1 genes in the five duplication types. In GO enrichment analysis, all five duplication types exhibited divergent functions. TRD was not enriched to a significant GO term. WGD and DSD were mainly enriched in the GO terms of plastid stroma, chloroplast stroma, obsolete chloroplast part, organellar small ribosomal subunit, and organellar ribosome. PD and TD shared more enriched GO terms related to pyrroline-5-carboxylate reductase activity, L-proline biosynthetic process, rRNA processing, protein disulfide oxidoreductase activity, peroxisome, cysteine-type peptidase activity, terpene synthase activity, magnesium ion binding, defense response to fungus, rRNA binding, response to wounding, and small ribosomal subunit compared with the other duplication types. KEGG enrichment analysis of PD and TD showed that these genes were mainly enriched in heat shock 70-kDa protein 1/2/6/8, molecular chaperone HtpG, (-)-germacrene D synthase, and KUP system potassium uptake protein, suggesting that the PD and TD genes in *C. spinosa* play important roles in environmental stress tolerance (Fig. S4).

Analyses of genome synteny and WGD

To analyze the evolution of the *C. spinosa* genome, dot plots of longer syntenic blocks within the *C. spinosa* genome were completed. *C. spinosa* undergoing WGD was clearly seen at Chr19 and Chr21

(Fig. S5A). Moreover, the syntenic blocks and collinear gene pairs between *C. spinosa* and *Amborella trichopoda*, *C. spinosa* and *A. thaliana*, *C. spinosa* and *Theobroma cacao*, *C. spinosa* and *Vitis vinifera*, and *C. spinosa* and *Solanum lycopersicum* were implemented, respectively (Fig. S5). The syntenic analysis results also showed more collinear gene pairs between *C. spinosa* and *A. thaliana* (Table S7), indicating that *C. spinosa* has a close evolutionary relationship with *A. thaliana*. At the same time, it can be seen from the stacking diagram of collinear genes on chromosomes that C. *spinosa* underwent WGD alone after divergence from *A. thaliana* (Fig. S5D).

Using the homologous gene pairs identified above, the 4DTv and Ks values were calculated for *C. spinosa*, *V. vinifera*, *S. lycopersicum*, *A. thaliana*, and *T. cacao*. The results showed that *C. spinosa* and *A. thaliana* separated at 53.00 MYA (Ks peak of 0.936 and 4DTv peak of 0.254). This is consistent with the divergence of *C. tomentosa* and *A. thaliana* at 50.1 MYA reported by Salariato et al. [26]. After divergence, *A. thaliana* experienced one WGD event, *C. spinosa* experienced two WGD events at 18.59 MYA (Ks peak at 0.328) and 2.946 MYA (Ks peak at 0.052). The results also showed that *C. spinosa* and *T. cacao* separated at 93.10 MYA (Ks peak of 1.644 and 4DTv peak of 0.336) (Fig. 4 A,B).

We compared the LTR insertion time of *A. thaliana*, *C. spinosa*, *S. lycopersicum*, *T. cacao*, *Tarenaya hassleriana*, and *V. vinifera* (Fig. 4C). The results indicated that LTR bursts the time peak of *C. spinosa* (peak at 0.178 MYA) between *A. thaliana* (peak at 0.236 MYA) and *T. hassleriana* (peak at 0.132 MYA), which was also consistent with the phylogenetic tree (Fig. 5B).

Gene family expansion and contraction

Protein sequences of 15 species, namely *Oryza sativa*, *Brachypodium distachyon*, *Ananas comosus*, *Musa acuminata*, *Cinnamomum micranthum*, *Nelumbo nucifera*, *Tetracentron sinense*, *V. vinifera*, *S. lycopersicum*, *A. trichopoda*, *Nymphaea colorata*, *T. hassleriana*, *A. thaliana*, *T. cacao*, *Populus*

trichocarpa, together with *C. spinosa*, were downloaded for gene family expansion and contraction analysis. As a result, all protein-coding genes were clustered into 49,850 orthogroups based on sequence homology. A total of 1,846 gene families were shared by all 16 species, and 142 *C. spinosa*-specific gene families were found (Fig. 5A). Moreover, the GO enrichment analysis revealed that species-specific genes were enriched in response to bacterium, oxidation–reduction pathways, and sterol biosynthetic process (Fig. S6A).

Based on the 306 orthogroups of single-copy genes, the phylogenetic tree was constructed and the MCMCTree program in PAML was used to estimate divergence times. The phylogenetic tree identified the closest relationship of *C. spinosa* to *T. hassleriana*. Based on the time tree, the number of gene families that experienced expansion or contraction was estimated by computational analysis of gene family evolution (CAFE). The results showed that in almost species, except *B. distachyon* and *A. thaliana*, more gene families experienced expansion rather than contraction. In *C. spinosa*, 26 gene families experienced expansion, while 11 gene families underwent contraction (Fig. 5B). GO enrichment analysis of the expanded gene families of *C. spinosa* showed that these genes were mainly enriched in chloroplast thylakoid, chloroplast envelope, thylakoid, chloroplast thylakoid membrane, response to abscisic acid, response to the hormone, and so forth (Fig. 5C). Moreover, based on KEGG enrichment analysis, the genes related to photosynthesis, chloroplast thylakoid membrane, and response to abscisic acid of hormone-related pathways were enriched (Fig. S6B). The function for these gene families expanded in *C. spinosa*, indicating that the expansion of the hormone response pathway and the photosynthesis pathway might have helped *C. spinosa* to generate more energy to adapt to arid environments.

Discussion

Currently, genetic research in the Capparaceae family is limited by the lack of its own genomic resources,

especially a reference genome. Here, we report a chromosome-level genome assembly of *C. spinosa*, with a contig N50 of 9.36 Mb and scaffold N50 of 15.15 Mb, providing the first reference genome for the Capparaceae family. The genome assembly was 274.53 Mb, and >99.23% of the assembled genome was on 21 chromosomes. This represents a contiguous and high-quality genome assembly similar to recently sequenced species genomes of Brassicaceae [27, 28]. The high quality of our assembly can be attributed to the use of the combination of PacBio HiFi sequencing and Hi-C data.

Interestingly, the high TR percentage and GC content of the genome can affect the accuracy of the genome assembly. In this study, the percentage of TR in the *C. spinosa* genome was 19.28%, which was much higher than the average value of 8.55% in plants [27]. In addition, we found localized high GC content in the *C. spinosa* genome, for example, the GC content of Chr06: 3700000–15800000 in the *C. spinosa* genome was 53.92%, much higher than the genomic GC content of 36.61%, which may affect the assembly accuracy of this segment on Chr06. The effect of assembly quality can be seen at the corresponding Chr06 position in the Hi-C contact map (Fig. S2). Although the Illumina Hi-C sequencing favored the anchoring of the scaffolds in the chromosomes, the lack of genetic maps leaves the anchor a bit weak.

WGDs are particularly prevalent in angiosperms and play important roles in the evolutionary history of angiosperms [28]. This *C. spinosa* genome assembly can improve the understanding of the timing of WGD events in the Capparaceae family. Because TR genes can affect the distribution of Ks peaks [29], and the *C. spinosa* genome had a high proportion of TRs, we calculated Ks and 4DTv separately for the five duplication types. The results show that the last duplication of WGD was before that of the other four duplication types. Compared with the other duplication types, PD had the highest ratio of Ka/Ks >1, indicating strong positive selection. The peaks of Ks (0.069) and 4DTv (0.013) also confirmed that the duplication of PD was very recent. Compared with *A. thaliana*, *S. lycopersicum*, *T.*

cacao, *T. hassleriana*, and *V. vinifera*, the WGD times for *C. spinosa* were smaller (Fig. 4), which might be because small populations had less time to expand, which is supported by the relatively recent (small Ks and 4DTv values) WGD in small populations [30].

As a medicinal plant, *C. spinosa* contains various bioactive compounds that have long been used in traditional medicine [9-14], which include terpenoids. The GO enrichment analysis of positively selected genes revealed that seven genes associated with terpene synthase activity were involved in the top 20 enriched pathways (Fig. S6).

Over a long period of evolution, *C. spinosa* has well adapted itself to drought and high temperature environments; for instance, KEGG enrichment analysis showed that five genes associated with heat shock protein (HSP) were involved in the top 20 enriched pathways (Fig. S6). The ability of plants to use light energy through photosynthesis declines under stressful conditions, which leads to the production of a large amount of reactive oxygen species because excess light energy has not been used for photosynthesis, and ultimately causes photoinhibition and oxidative damage to chloroplasts and other cell structures [39]. *In vivo* and *in vitro* studies showed that when plants are exposed to drought and heat stress, the expression of a series of HSP genes is induced, most of which interact with other proteins in the cell and alter their function, protecting against harmful effects [40-42], thus finding the enrichment of HSP genes in *C. spinosa* is explaining their role in determining drought and high-temperature stress tolerance in *C. spinosa*.

In this study, we also presented a chromosome-level genome assembly of *C. spinosa* using the combination of PacBio CCS and Hi-C data. The final genome assembly was grouped into 21 chromosomes with a size of 274.53 Mb. The high-quality reference *C. spinosa* genome assembled in this study is the first reported genomic resource for the Capparaceae family and can facilitate future studies on the mechanisms of drought and high-temperature resistance in this species, providing a

system for studying the diversity, speciation, and evolution of this family.

Methods

Plant materials and nucleic acid extraction

The source plant (Fig. 1) was an individual of *Capparis spinosa* sub. *spinosa* var. *herbacea* grown in the field near the Turpan Eremophytes Botanical Garden, Xinjiang Institute of Ecology and Geography, Chinese Academy of Sciences (40°51′ N, 98°11′ E, −75 m elevation). The sample was identified and confirmed by taxonomist Xiyong Wang of Xinjiang Institute of Ecology and Geography, Chinese Academy of Sciences, and deposited in its herbarium. On September 14, 2020, fresh and healthy leaves were harvested and immediately frozen in liquid nitrogen, followed by storage at −80°C in the laboratory before DNA and RNA extraction.

Genomic DNA was extracted from the fresh leaf tissue (200 mg) that had been ground in liquid nitrogen using cetyltrimethylammonium bromide buffer (60 min incubation at 65°C), followed by phenol/chloroform/isoamyl purification (25:24:1), and isopropanol and ethanol precipitation. The resulting purified DNA was resuspended in Tris-EDTA buffer for subsequent sequencing [43]. Total RNA was extracted from the fresh samples of roots, stems, leaves, flowers, and fruits according the instructions of RNAprep Pure Plant Plus Kit (DP441, Tiangen, China). The RNA from the above tissues was mixed in equal amounts and used for RNA sequencing library construction.

Library construction and sequencing

PacBio library construction and sequencing were performed following the standard protocols provided by PacBio. Genomic DNA was sheared into ~15 kb fragments by Megaruptor 2. The SMRTbell library

was constructed using the SMRTbell Express Template Prep Kit 2.0 (Pacific Biosciences, CA, USA). Library size and quantity were assessed using the FEMTO Pulse and the Qubit dsDNA HS reagents Assay kit (Thermo Fisher Scientific, Waltham, MA, USA). Sequencing primer and Sequel II DNA Polymerase were annealed and bound, respectively, to the final SMRTbell library. The library was loaded at an on-plate concentration of 55 pM using diffusion loading. SMRT sequencing was performed using a single 8M SMRT Cell on the PacBio Sequel II System with Sequel II Sequencing Kit.

The sequencing for genome survey was performed according to the standard protocol provided by Illumina. Using the extracted genomic DNA, small fragment library construction and sequencing were performed. Qualified genomic DNA was fragmented to the target fragment (350 bp) by physical fragmentation (ultrasonic vibration), followed by end repair, polyadenylation, adapter ligation, target fragment selection, and PCR [44]. The library was sequenced with paired-ended 150 bp (PE 150) using the Illumina NovaSeq 6000 platform.

Instructions of the VAHTS Universal V6 RNA-seq Library Prep Kit for Illumina (NR604-02; Vazyme, China) were followed to construct the transcriptomic short reads library. The constructed library was sequenced on the Illumina NovaSeq 6000 platform. The transcriptomic long reads library was obtained after using the SMRTbell Template Prep Kit to perform damage repair, end repair, and ligation of the mixed products. The reaction was performed in a PCR thermal cycler or a constant temperature metal bath. After RNA reverse transcription and PCR amplification, the library was sequenced on a PacBio Sequel II system.

Hi-C fragment libraries were constructed as reported by Fu et al. [45]. The main procedures included cross-linking DNA, restriction enzyme digestion, end repair, DNA circularization, and DNA purification. This library was sequenced on the Illumina NovoSeq 6000 platform.

Estimation of genome features

Genome survey was performed on the Illumina NovaSeq 6000 sequencing platform. The short reads were quality filtered using Fastp v0.23.0 with default parameters [46]. K-Mer Counter (KMC) v3.0.0 with the parameters kmc -k17 -t24 -m64 -ci1 -cs20000 @FILES reads tmp and kmc_tools transform reads histogram reads.histo -cx20000 was used to obtain the K-mer file from the clean data [47].

GenomeScope 2.0 with the parameters genomescope.R -i reads.histo -o output -k 17 was used to estimate genome heterozygosity, repeat sequences, and size from the k-mer file [48].

For flow cytometry-based prediction, samples were placed in 500 µl nuclei extraction buffer, chopped with a sharp blade, and filtered through a 50-µm filter after 60 s. This was followed by the addition of 2000 µl of staining buffer with RNase for 15 min in dark. Nuclei suspensions were analyzed by CyFlow Space flow cytometer (Sysmex Partec, Muenster, Germany) and the corresponding FloMax software. The genome size of *C. spinosa* was calculated according to the formula "peak (ref)/genome size (ref) = peak (*Capparis spinosa*)/genome size (*Capparis spinosa*)" using *Solanum pimpinellifolium* as a reference genome with length 800 Mb [49].

Chromosome-level assembly with Hi-C data

BWA aligner v0.7.17 [50] was used to align the clean Hi-C reads to the assembly results, and uniquely alignable read pairs with mapping quality more than 20 were retained for further analysis. Invalid read pairs, including dangling ends and self-cyclization, re-ligated, and dumped products, were filtered by HiC-Prov2.8.1 [51]. LACHESIS [52] was used for clustered, ordered, and oriented scaffolds onto chromosomes. Parameters for running LACHESIS were as follows:

CLUSTER_MAX_LINK_DENSITY=2; C-LUSTER_MIN_RE_SITES=9; ORDER_MIN_N_RES_IN_SHREDS=15; ORDER_MIN_N_RES_IN_TRUN=15. Clean Hi-C reads, accounting for 100-fold coverage of the survey genome, and the final 28 scaffolds were anchored to chromosomes, accounting for 99.98% of the total length. The Hi-C interactions were used as evidence for contig proximity and scaffold/contig sequences.

Genome assembly and evaluation

The raw PacBio sequencing reads were assembled using Hifiasm v0.14 [53] with the parameters -l 2 -n 4. Purge_dups v1.2.5 (default parameters) [54] was used to identify and remove haplotypic duplication in the genome assembly.

Five methods were used to evaluate the quality of the genome assembly, including the second-generation data return ratio, CEGMA evaluation, BUSCO evaluation, Merqury, and LAI value evaluation. BWA-MEM v0.7.17 (default parameters) [50] was used to compare the short reads obtained from the Illumina HiSeq sequencing data with the reference genome. CEGMA v2.5 [55], which contains 458 conserved core eukaryotic genes, was used to evaluate the completeness of the genome assembly. The Embryophyta database of BUSCO v5.2.1 [56] contains 1,614 conserved core genes that were used to assess the integrity of the genome assembly. Assembly QV was calculated using Merqury v1.3 [57].

Full-length LTR retrotransposons (LTR-RTs) in the genome were identified by LTR_finder v1.07 [58] and LTRharvest v1.6.1 [59]. LTR_retriever v2.9.0 [60] was then used to combine LTR-RTs, remove duplicates, and calculate the LAI value and calculate the insertion time of LTR-RTs. LTR_finder parameters were -D 40000 -d 100 -L 9000 -l 50 -p 20 -C -M 0.9. LTRharvest parameters were -minlenltr 100 -maxlenltr 40000 -mintsd 4 -maxtsd 6 -motif TGCA -motifmis 1 -similar 85 -vic 10 -seed 20 -seqids yes. LTR_retriever was set with the parameter -u 7e-9, which is used to set the molecular clock r value

to 7*10^{-9} [61].

Identification of repeat sequences

TEs and TRs were identified separately. We combined homology-based and *de novo* approaches to identify TEs. We first customized a *de novo* repeat library of the genome using RepeatModeler2 v2.0.1 (default parameters) [62]. The *de novo* TE-sequence library and LTR-RT library described above were merged with the known Repbase v19.06, REXdb v3.0 and Dfam v3.2 databases. After removing redundant sequences using the seqkit v2.1.0 (parameter: rmdup -s) [63], a non-redundant species-specific TE library was constructed. TE sequence was identified and classified by RepeatMasker v4.1.1 (default parameters) [64]. TRs were identified by MISA v2.1 [65] with default parameters and TRF v4.09 [66] with the parameters 1 1 2 80 5 200 2000 -d -h.

Gene prediction and annotation

The three approaches of *de novo* prediction, homology search, and transcript-based assembly were used to annotate protein-coding genes [47]. Augustus v3.1 (default parameters) [67] and SNAP v2013-02-16 (default parameters) [68] were used for *de novo* prediction. Homologous species were predicted in GeMoMa v1.7 (default parameters) [69] using the reference gene models of *A. thaliana*, *Cannabis sativa*, *Eutrema salsugineum*, and *T. hassleriana*. For the transcript-based prediction, RNA-sequencing data were mapped to the reference genome using HISAT2 v2.2.0 (parameters: --max-intronlen 20000, --min-intronlen 20) [70] and assembled by Stringtie v2.1.3b (default parameters) [71]. GeneMarkS-T v5. 1 (default parameters) [72] was used for gene prediction based on the assembled transcripts. The PASA v2.4.1 (default parameters) [73] was used to predict genes based on the unigenes (and full-length

transcripts from the PacBio sequencing) assembled by Trinity v2.11.0 (parameters: -max_memory 100g) [74]. Gene models from these different approaches were combined using the EVM v1.1.1 (default parameters) [73] and updated by PASA.

The predicted gene sequences were used as queries for BLAST v2.2.31 (Altschul et al., 1990) searches against the NR (202009) [75], TrEMBL (202005) [76], Pfam v33.1 [77], Swiss-Prot (202005) [78], KOG (20110125) [79], GO (20200615) [80], and KEGG (20191220) [81] databases for gene annotation. tRNA was identified using tRNAscan-SE v1.3.1 [82], rRNA was predicted based on the Rfam v12.0 database [83] and Barrnap v0.9 [84], miRNA was identified by the miRbase v22 database [85], and snoRNA and snRNA were based on the Rfam database and predicted by Infernal v1.1 [86]. A total of 0 tRNAs, 2,722 rRNAs, and 100 miRNAs were predicted.

WGD analysis

GenDup_finder-unique, the stringent mode of DupGen_finder [87], was used to identify genes derived from the different duplication types. : DupGen_finder-unique divided the duplication types into five types, namely WGD, TD, PD (separated by fewer than ten genes on the same chromosome), TRD, and DSD. The Ka, Ks, and Ka/Ks values of gene pairs were calculated with ParaAT v2.0 [88]. The proportion of each homologous gene to the 4DTv site was calculated using a Perl script. Genes with Ka/Ks >1 in the five duplication types were used for GO and KEGG enrichment analysis by clusterProfiler v4.2.0 [89].

Gene family classification

The protein sequences of 16 species (*M. acuminata, T. sinense*, *C. micranthum, A. trichopoda, T.*

hassleriana, *A. comosus*, *S. lycopersicum*, *T. cacao*, *A. thaliana*, *B. distachyon*, *N. nucifera*, *P. trichocarpa*, *V. vinifera*, *N. colorata*, *O. sativa*, and *C. spinosa*) were used for family classification using OrthoFinder v2.4 software (diamond comparison method, E-value 0.001) [90]. The encoding genes from a species were clustered into six groups—0 copies, 1 copy (single-copy), 2 copies, 3 copies, 4 copies, and 4+ copies. A total of 306 genes were identified as single-copy genes. The obtained gene families were annotated using the PANTHER v15 database [91].

Phylogenetic analysis and species divergence time estimation

Each single-copy gene family sequence was aligned using MAFFT v7.205 [92] (parameters: --localpair --maxiterate 1000). Gblocks v0.91b [93] (parameter: -b5=h) was used to filter conserved sites, and all aligned gene family sequences of each species were finally connected end-to-end to obtain supergenes. The IQ-TREE v1.6.11 [94] model selection tool ModelFinder [95] was used for model selection. The best model obtained was JTT+F+I+G4, which was used to construct the phylogenetic tree by the maximum likelihood (ML) method with the bootstrap value set to 1000. *A. trichopoda* was selected as the outgroup and the root of the tree [45]. The divergence time between species was estimated using the TimeTree website (http://www.timetree.org/) [96]. Divergence times were as follows: *A. trichopoda vs S. lycopersicum* at 164–194 MYA, *O. sativa vs B. distachyon* at 42–60 MYA, *A. comosus vs O. sativa* at 94–115 MYA, and *N. nucifera vs V. vinifera* at 116–127 MYA. The gradient and Hessian parameters required for the divergence time were estimated using MCMCTree in PAML v4.9i [97]. The ML method, correlated molecular clock, and JC69 model were used to estimate divergence times. Two repeated calculations were performed to evaluate consistency. The Markov Chain Monte Carlo (MCMC) iteration settings were burn-in 2000, sampfreq 10, and nsample 20000. MCMCtreeR v1.1 [98] was used to graphically display the phylogenetic tree with divergence times.

Gene family expansion and contraction

The results of the phylogenetic tree (with divergence times) and gene family clustering analyses were used to estimate the gene family expansion and contraction of species relative to their ancestors using CAFE v4.2 [99]. The criteria defining significant expansion or contraction of gene families were a family-wide *P*-value < 0.05 and a Viterbi *P*-value < 0.05.

Genome collinearity analysis

To identify similar gene pairs, gene sequences of two species were compared using Diamond v0.9.29.130 (parameter: e<1e-5) [100]. JCVI v0.9.13 [101] was used to filter the BLAST results (parameter: C-score >0.5) and obtain all the genes in collinear blocks. JCVI was also used to plot the collinearity of the linear pattern of each species. Finally, the ggplot2 v3.3.5 R package [102] was used to display the collinearity results in the form of bar graphs.

Genome information visualization

The sliding window file of the genome was constructed using Bedtools v2.29.2 [103], and the window size was set to 100 kb to calculate the gene density of each chromosome. The distribution of gene density, TE sequence, TRs, and GC content, and collinearity on the chromosomes of the genome were visualized using Circos v0.69-8 [104].

Correlation analysis of genomic distribution characteristics

We performed pairwise correlation analysis on the GC content, gene density, TE distribution, and TR

distribution of the genome. Correlation analysis was performed using the Spearman method [105] using the cor function in R. The corrplot R package was used to visualize the correlation results.

Data availability

Raw data of genome assembly (PacBio HiFi and Hi-C sequences) have been deposited in the NCBI Sequence Read Archive (SRA) database under Bioproject ID: **PRJNA792936**. The genome annotations have been deposited at FigShare (https://doi.org/**10.6084/m9.figshare.17702051**). The whole-genome sequence data have been deposited in the Genome Warehouse at the National Genomics Data Center, Beijing Institute of Genomics, under accession number **GWHBGXB00000000**, and are publicly accessible at https://ngdc.cncb.ac.cn/gwh.

List of abbreviations

CAFE: computational analysis of gene family evolution; DSD: dispersed duplication; Hi-C: high-throughput chromosome conformation capture; HSP: heat shock protein; LTR-RTs: LTR retrotransposons; MYA: million years ago; PD: proximal duplication; QV: quality value; SRA: Sequence Read Archive; TD: tandem duplication; TE: transposable element; TRs: tandem repeats; TRD: transposed duplication; WGD: whole-genome duplication

Ethical statement

Not applicable

Consent for publication

Not applicable

Competing interests

The authors declare that they have no conflicts of interest.

Funding

This work was supported by the National Key Research and Development Program of China (No. 2018YFE0207200).

Authors' contributions

Lei Wang: Methodology, Sample collection, Writing - Original draft, Writing - Review & Editing. **Liqiang Fan**: Data analysis, Methodology. Writing - Original draft. **Zhenyong Zhao**: Sample collection, Investigation, Methodology. **Li Jiang**: Investigation, Methodology. **Zhibin Zhang**: Data analysis. **Mao Chai**: Data curation, Visualization, Supervision, Writing - Review. **Changyan Tian**: Project administration, Supervision, Resources, Funding acquisition, Writing - Review & Editing.

Acknowledgements

We thank Dr. Zhaoen Yang (State Key Laboratory of Cotton Biology, Institute of Cotton Research of the Chinese Academy of Agricultural Sciences, Zhengzhou, China) for providing help in data analysis. We also thank TopEdit (www.topeditsci.com) for linguistic assistance during the preparation of this manuscript.

References

Uncategorized References

1. Inocencio C, Rivera D, Obón MC, Alcaraz F and Barreña J-A. A

systematic revision of capparis section Capparis (Capparaceae) 1, 2. Annals of the Missouri Botanical Garden. 2006;93 1:122-49.

2. Levizou E, Drilias P and Kyparissis A. Exceptional photosynthetic performance of Capparis spinosa L. under adverse conditions of Mediterranean summer. Photosynthetica. 2004;42 2:229-35.

3. Özcan M and Akgül A. Influence of species, harvest date and size on composition of capers (Capparis spp.) flower buds. Food/Nahrung. 1998;42 02:102-5.

4. Yang T, Liu Y-Q, Wang C-H and Wang Z-T. Advances on investigation of chemical constituents, pharmacological activities and clinical applications of Capparis spinosa. Zhongguo Zhong yao za zhi= Zhongguo zhongyao zazhi= China journal of Chinese materia medica. 2008;33 21:2453-8.

5. Fici S. A taxonomic revision of the Capparis spinosa group (Capparaceae) from the Mediterranean to Central Asia. Phytotaxa. 2014;174 1:1–24-1–.

6. Chedraoui S, Abi-Rizk A, El-Beyrouthy M, Chalak L, Ouaini N and

Rajjou L. Capparis spinosa L. in A Systematic Review: A Xerophilous Species of Multi Values and Promising Potentialities for Agrosystems under the Threat of Global Warming. Frontiers in Plant Science. 2017;8 doi:10.3389/fpls.2017.01845.

7. Gan L, Zhang C, Yin Y, Lin Z, Huang Y, Xiang J, et al. Anatomical adaptations of the xerophilous medicinal plant, Capparis spinosa, to drought conditions. Horticulture, Environment, and Biotechnology. 2013;54 2:156-61.

8. Zuo W, Ma M, Ma Z, Gao R, Guo Y, Jiang W, et al. Study of photosynthetic physiological characteristics of desert plant Capparis spinosa L. Journal of Shihezi University (Natural Science). 2012;30 3:7.

9. Anwar F, Muhammad G, Hussain MA, Zengin G, Alkharfy KM, Ashraf M, et al. Capparis spinosa L.: A plant with high potential for development of functional foods and nutraceuticals/pharmaceuticals. International Journal of Pharmacology. 2016;12 3:201-19.

10. Arrar L, Benzidane N, Krache I, Charef N, Khennouf S and Baghiani

A. Comparison between polyphenol contents and antioxidant activities of different parts of Capparis spinosa L. Pharmacognosy Communications. 2013;3 2:70.

11. Germano MP, De Pasquale R, D'angelo V, Catania S, Silvari V and Costa C. Evaluation of extracts and isolated fraction from Capparis spinosa L. buds as an antioxidant source. Journal of agricultural and food chemistry. 2002;50 5:1168-71.

12. Matthäus B and Özcan M. Glucosinolates and fatty acid, sterol, and tocopherol composition of seed oils from Capparis spinosa Var. spinosa and Capparis ovata Desf. Var. canescens (Coss.) Heywood. Journal of Agricultural and Food chemistry. 2005;53 18:7136-41.

13. Tlili N, Feriani A, Saadoui E, Nasri N and Khaldi A. Capparis spinosa leaves extract: Source of bioantioxidants with nephroprotective and hepatoprotective effects. Biomedicine & Pharmacotherapy. 2017;87:171-9.

14. Tlili N, Nasri N, Khaldi A, Triki S and MUNNÉ-BOSCH S. Phenolic compounds, tocopherols, carotenoids and vitamin C of commercial

caper. Journal of Food Biochemistry. 2011;35 2:472-83.

15. Zhang H and Ma ZF. Phytochemical and Pharmacological Properties of Capparis spinosa as a Medicinal Plant. Nutrients. 2018;10 2:116.

16. Bektas N, Arslan R, Goger F, Kirimer N and Ozturk Y. Investigation for anti-inflammatory and anti-thrombotic activities of methanol extract of Capparis ovata buds and fruits. Journal of ethnopharmacology. 2012;142 1:48-52.

17. Hall JC. Systematics of Capparaceae and Cleomaceae: an evaluation of the generic delimitations of Capparis and Cleome using plastid DNA sequence data. Botany. 2008;86 7:682-96.

18. Siragusa M and Carimi F. Development of specific primers for cpSSR analysis in caper, olive and grapevine using consensus chloroplast primer pairs. Scientia horticulturae. 2009;120 1:14-21.

19. Wang Q, Zhang ML and Yin LK. Phylogeographic structure of a tethyan relict Capparis spinosa (Capparaceae) traces Pleistocene geologic and climatic changes in the western Himalayas, Tianshan

mountains, and adjacent desert regions. BioMed research international. 2016;2016:13.

20. Maurya S, Darshetkar AM, Datar MN, Tamhankar S, Li P and Choudhary RK. Plastome data provide insights into intra and interspecific diversity and ndh gene loss in Capparis (Capparaceae). Phytotaxa. 2020;432 2:206-20.

21. Alzahrani D, Albokhari E, Yaradua S and Abba A. The complete plastome sequence for the medicinal species Capparis spinosa L. (Capparaceae). Gene Reports. 2021;23:101059. doi:https://doi.org/10.1016/j.genrep.2021.101059.

22. Grewe F, Edger PP, Keren I, Sultan L, Pires JC, Ostersetzer-Biran O, et al. Comparative analysis of 11 Brassicales mitochondrial genomes and the mitochondrial transcriptome of Brassica oleracea. Mitochondrion. 2014;19:135-43.

23. Mercati F, Fontana I, Gristina AS, Martorana A, El Nagar M, De Michele R, et al. Transcriptome analysis and codominant markers development in caper, a drought tolerant orphan crop with medicinal

value. Scientific reports. 2019;9 1:1-16.

24. DOLEŽEL J and BARTOŠ J. Plant DNA Flow Cytometry and Estimation of Nuclear Genome Size. Annals of Botany. 2005;95 1:99-110. doi:10.1093/aob/mci005.

25. Ou S, Chen J and Jiang N. Assessing genome assembly quality using the LTR Assembly Index (LAI). Nucleic Acids Research. 2018;46 21:e126-e. doi:10.1093/nar/gky730.

26. Salariato DL, Zuloaga FO, Franzke A, Mummenhoff K and Al-Shehbaz IA. Diversification patterns in the CES clade (Brassicaceae tribes Cremolobeae, Eudemeae, Schizopetaleae) in Andean South America. Botanical Journal of the Linnean Society. 2016;181 4:543-66.

27. Tørresen OK, Star B, Mier P, Andrade-Navarro MA, Bateman A, Jarnot P, et al. Tandem repeats lead to sequence assembly errors and impose multi-level challenges for genome and protein databases. Nucleic Acids Research. 2019;47 21:10994-1006. doi:10.1093/nar/gkz841.

28. Christenhusz MJM and Byng JW. The number of known plants species in the world and its annual increase. Phytotaxa. 2016;261 3:201-17.

29. Taikui Zhang ZY. Progress in plant paleogenomics. Hereditas(Beijing). 2018;40 1:44-56. doi:10.16288/j.yczz.17-191.

30. Ren, Wang, HF, Guo, CC, Zhang, et al. Widespread Whole Genome Duplications Contribute to Genome Complexity and Species Diversity in Angiosperms. MOL PLANT. 2018;2018,11(3) -:414-28.

31. Bohlmann J, Meyer-Gauen G and Croteau R. Plant terpenoid synthases: Molecular biology and phylogenetic analysis. Proc Natl Acad Sci U S A. 1998;95 8:4126-33.

32. Berta A, Ana R, Marcos D and Leandro P. Genomic Analysis of Terpene Synthase Family and Functional Characterization of Seven Sesquiterpene Synthases from Citrus sinensis. Frontiers in Plant Science. 2017;8.

33. Chen C, Zheng Y, Zhong Y, Wu Y and Meng X. Transcriptome analysis and identification of genes related to terpenoid biosynthesis

in Cinnamomum camphora. BMC Genomics. 2018;19 1.

34. Chen H, Kllner TG, Li G, Wei G and Chen F. Combinatorial Evolution of a Terpene Synthase Gene Cluster Explains Terpene Variations in Oryza. Plant Physiology. 2019;182 1:pp.00948.2019.

35. Hansen NL, Heskes AM, Hamberger B, Olsen CE and Hamberger B. The terpene synthase gene family in Tripterygium wilfordii harbors a labdane-type diterpene synthase among the monoterpene synthase TPS-b subfamily. Plant Journal. 2017;89 3.

36. Karunanithi PS and Zerbe P. Terpene Synthases as Metabolic Gatekeepers in the Evolution of Plant Terpenoid Chemical Diversity. Frontiers in Plant Science. 2019;10:1166-.

37. Shu-Ye J, Jingjing J, Rajani S and Srinivasan R. A Comprehensive Survey on the Terpene Synthase Gene Family Provides New Insight into Its Evolutionary Patterns. Genome Biology and Evolution. 2019; 8:8.

38. Xiong W, Wu P, Jia Y, Wei X, Xu L, Yang Y, et al. Genome-wide analysis of the terpene synthase gene family in physic nut (Jatropha

curcas L.) and functional identification of six terpene synthases. Tree Genetics & Genomes. 2016;12 5:97.

39. Tanveer, M. (2019). Role of 24-Epibrassinolide in inducing thermo-tolerance in plants. Journal of Plant Growth Regulation, 38(3), 945-955.

40. Ohama N, Sato H, Shinozaki K and Yamaguchi-Shinozaki K. Transcriptional regulatory network of plant heat stress response. Trends in plant science. 2017;22 1:53-65.

41. Ren S, Ma K, Lu Z, Chen G and Jin B. Transcriptomic and Metabolomic Analysis of the Heat-Stress Response of Populus tomentosa Carr. Forests. 2019;10 5:383.

42. Tereza T, Despina S, Anna K, Tereza V and Jozef Š. Multifaceted roles of HEAT SHOCK PROTEIN 90 molecular chaperones in plant development. Journal of Experimental Botany. 2020;71 14:20.

43. Fu J, Wan L, Song L, He L, Jiang N, Long H, et al. Chromosome-Level Genome Assembly of the Hemiparasitic Taxillus chinensis (DC.) Danser. Genome Biology and Evolution. 2022;14 5

doi:10.1093/gbe/evac060.

44. Jiang S, An H, Xu F and Zhang X. Chromosome-level genome assembly and annotation of the loquat (Eriobotrya japonica) genome. GigaScience. 2020;9 3 doi:10.1093/gigascience/giaa015.

45. Fu A, Wang Q, Mu J, Ma L, Wen C, Zhao X, et al. Combined genomic, transcriptomic, and metabolomic analyses provide insights into chayote (Sechium edule) evolution and fruit development. Horticulture Research. 2021;8 1:35. doi:10.1038/s41438-021-00487-1.

46. Chen S, Zhou Y, Chen Y and Gu J. fastp: an ultra-fast all-in-one FASTQ preprocessor. Bioinformatics. 2018;34 17:i884-i90.

47. Kokot M, Długosz M and Deorowicz S. KMC 3: counting and manipulating k-mer statistics. Bioinformatics. 2017;33 17:2759-61. doi:10.1093/bioinformatics/btx304.

48. Ranallo-Benavidez TR, Jaron KS and Schatz MC. GenomeScope 2.0 and Smudgeplot for reference-free profiling of polyploid genomes. Nature Communications. 2020;11 1:1432.

doi:10.1038/s41467-020-14998-3.

49. Wang X, Gao L, Jiao C, Stravoravdis S, Hosmani PS, Saha S, et al. Genome of Solanum pimpinellifolium provides insights into structural variants during tomato breeding. Nature Communications. 2020;11 1:5817. doi:10.1038/s41467-020-19682-0.

50. Li H. Aligning sequence reads, clone sequences and assembly contigs with BWA-MEM. arXiv preprint arXiv:13033997. 2013.

51. Servant N, Varoquaux N, Lajoie BR, Viara E, Chen C-J, Vert J-P, et al. HiC-Pro: an optimized and flexible pipeline for Hi-C data processing. Genome Biology. 2015;16 1:259. doi:10.1186/s13059-015-0831-x.

52. Burton JN, Adey A, Patwardhan RP, Qiu R, Kitzman JO and Shendure J. Chromosome-scale scaffolding of de novo genome assemblies based on chromatin interactions. Nature Biotechnology. 2013;31 12:1119-25. doi:10.1038/nbt.2727.

53. Cheng H, Concepcion GT, Feng X, Zhang H and Li H. Haplotype-resolved de novo assembly using phased assembly graphs with

hifiasm. Nature Methods. 2021;18 2:170-5. doi:10.1038/s41592-020-01056-5.

54. Guan D, McCarthy SA, Wood J, Howe K, Wang Y and Durbin R. Identifying and removing haplotypic duplication in primary genome assemblies. Bioinformatics. 2020;36 9:2896-8. doi:10.1093/bioinformatics/btaa025.

55. Parra G, Bradnam K and Korf I. CEGMA: a pipeline to accurately annotate core genes in eukaryotic genomes. Bioinformatics. 2007;23 9:1061-7.

56. Manni M, Berkeley MR, Seppey M, Simão FA and Zdobnov EM. BUSCO Update: Novel and Streamlined Workflows along with Broader and Deeper Phylogenetic Coverage for Scoring of Eukaryotic, Prokaryotic, and Viral Genomes. Molecular Biology and Evolution. 2021;38 10:4647-54. doi:10.1093/molbev/msab199.

57. Rhie A, Walenz BP, Koren S and Phillippy AM. Merqury: reference-free quality, completeness, and phasing assessment for genome assemblies. Genome Biology. 2020;21 1:245. doi:10.1186/s13059-

020-02134-9.
58. Xu Z and Wang H. LTR_FINDER: an efficient tool for the prediction of full-length LTR retrotransposons. Nucleic Acids Research. 2007;35 suppl_2:W265-W8. doi:10.1093/nar/gkm286.
59. Ellinghaus D, Kurtz S and Willhoeft U. LTRharvest, an efficient and flexible software for de novo detection of LTR retrotransposons. BMC Bioinformatics. 2008;9 1:18. doi:10.1186/1471-2105-9-18.
60. Ou S and Jiang N. LTR_retriever: A Highly Accurate and Sensitive Program for Identification of Long Terminal Repeat Retrotransposons. Plant Physiology. 2018;176 2:1410-22. doi:10.1104/pp.17.01310.
61. Ossowski S, Schneeberger K, Lucas-Lledó JI, Warthmann N, Clark RM, Shaw RG, et al. The rate and molecular spectrum of spontaneous mutations in Arabidopsis thaliana. science. 2010;327 5961:92-4.
62. Flynn JM, Hubley R, Goubert C, Rosen J, Clark AG, Feschotte C, et al. RepeatModeler2 for automated genomic discovery of

transposable element families. Proceedings of the National Academy of Sciences. 2020;117 17:9451. doi:10.1073/pnas.1921046117.

63. Shen W, Le S, Li Y and Hu F. SeqKit: A Cross-Platform and Ultrafast Toolkit for FASTA/Q File Manipulation. PLOS ONE. 2016;11 10:e0163962. doi:10.1371/journal.pone.0163962.

64. Tarailo-Graovac M and Chen N. Using RepeatMasker to identify repetitive elements in genomic sequences. Current protocols in bioinformatics. 2009;25 1:4.10.1-4..4.

65. Beier S, Thiel T, Münch T, Scholz U and Mascher M. MISA-web: a web server for microsatellite prediction. Bioinformatics. 2017;33 16:2583-5. doi:10.1093/bioinformatics/btx198.

66. Benson G. Tandem repeats finder: a program to analyze DNA sequences. Nucleic acids research. 1999;27 2:573-80.

67. Keller O, Kollmar M, Stanke M and Waack S. A novel hybrid gene prediction method employing protein multiple sequence alignments. Bioinformatics. 2011;27 6:757-63. doi:10.1093/bioinformatics/btr010.

68. Korf I. Gene finding in novel genomes. BMC Bioinformatics. 2004;5 1:59. doi:10.1186/1471-2105-5-59.

69. Keilwagen J, Hartung F, Paulini M, Twardziok SO and Grau J. Combining RNA-seq data and homology-based gene prediction for plants, animals and fungi. BMC Bioinformatics. 2018;19 1:189. doi:10.1186/s12859-018-2203-5.

70. Pertea M, Kim D, Pertea GM, Leek JT and Salzberg SL. Transcript-level expression analysis of RNA-seq experiments with HISAT, StringTie and Ballgown. Nature Protocols. 2016;11 9:1650-67. doi:10.1038/nprot.2016.095.

71. Pertea M, Pertea GM, Antonescu CM, Chang T-C, Mendell JT and Salzberg SL. StringTie enables improved reconstruction of a transcriptome from RNA-seq reads. Nature biotechnology. 2015;33 3:290-5.

72. Tang S, Lomsadze A and Borodovsky M. Identification of protein coding regions in RNA transcripts. Nucleic Acids Research. 2015;43 12:e78-e. doi:10.1093/nar/gkv227.

73. Haas BJ, Salzberg SL, Zhu W, Pertea M, Allen JE, Orvis J, et al. Automated eukaryotic gene structure annotation using EVidenceModeler and the Program to Assemble Spliced Alignments. Genome Biology. 2008;9 1:R7. doi:10.1186/gb-2008-9-1-r7.

74. Grabherr MG, Haas BJ, Yassour M, Levin JZ, Thompson DA, Amit I, et al. Full-length transcriptome assembly from RNA-Seq data without a reference genome. Nature Biotechnology. 2011;29 7:644-52. doi:10.1038/nbt.1883.

75. Marchler-Bauer A, Lu S, Anderson JB, Chitsaz F, Derbyshire MK, DeWeese-Scott C, et al. CDD: a Conserved Domain Database for the functional annotation of proteins. Nucleic acids research. 2010;39 suppl_1:D225-D9.

76. Boeckmann B, Bairoch A, Apweiler R, Blatter M-C, Estreicher A, Gasteiger E, et al. The SWISS-PROT protein knowledgebase and its supplement TrEMBL in 2003. Nucleic acids research. 2003;31 1:365-70.

77. Mistry J, Chuguransky S, Williams L, Qureshi M, Salazar GA,

Sonnhammer EL, et al. Pfam: The protein families database in 2021. Nucleic Acids Research. 2021;49 D1:D412-D9.

78. Boutet E, Lieberherr D, Tognolli M, Schneider M, Bansal P, Bridge AJ, et al. UniProtKB/Swiss-Prot, the Manually Annotated Section of the UniProt KnowledgeBase: How to Use the Entry View. In: Edwards D, editor. Plant Bioinformatics: Methods and Protocols. New York, NY: Springer New York; 2016. p. 23-54.

79. Koonin EV, Fedorova ND, Jackson JD, Jacobs AR, Krylov DM, Makarova KS, et al. A comprehensive evolutionary classification of proteins encoded in complete eukaryotic genomes. Genome biology. 2004;5 2:R7.

80. Dimmer EC, Huntley RP, Alam-Faruque Y, Sawford T, O'Donovan C, Martin MJ, et al. The UniProt-GO annotation database in 2011. Nucleic acids research. 2012;40 D1:D565-D70.

81. Kanehisa M and Goto S. KEGG: kyoto encyclopedia of genes and genomes. Nucleic acids research. 2000;28 1:27-30.

82. Chan PP and Lowe TM. tRNAscan-SE: searching for tRNA genes in

genomic sequences. Gene prediction. Springer; 2019. p. 1-14.

83. Nawrocki EP, Burge SW, Bateman A, Daub J, Eberhardt RY, Eddy SR, et al. Rfam 12.0: updates to the RNA families database. Nucleic acids research. 2015;43 D1:D130-D7.

84. Loman T. A Novel Method for Predicting Ribosomal RNA Genes in Prokaryotic Genomes. 2017.

85. Kozomara A, Birgaoanu M and Griffiths-Jones S. miRBase: from microRNA sequences to function. Nucleic acids research. 2019;47 D1:D155-D62.

86. Nawrocki EP and Eddy SR. Infernal 1.1: 100-fold faster RNA homology searches. Bioinformatics. 2013;29 22:2933-5.

87. Qiao X, Li Q, Yin H, Qi K, Li L, Wang R, et al. Gene duplication and evolution in recurring polyploidization–diploidization cycles in plants. Genome Biology. 2019;20 1:38. doi:10.1186/s13059-019-1650-2.

88. Zhang Z, Xiao J, Wu J, Zhang H, Liu G, Wang X, et al. ParaAT: a parallel tool for constructing multiple protein-coding DNA alignments. Biochemical and biophysical research communications. 2012;419

4:779-81.

89. Wu T, Hu E, Xu S, Chen M, Guo P, Dai Z, et al. clusterProfiler 4.0: A universal enrichment tool for interpreting omics data. The Innovation. 2021;2 3:100141.

90. Emms DM and Kelly S. OrthoFinder: phylogenetic orthology inference for comparative genomics. Genome Biology. 2019;20 1:238. doi:10.1186/s13059-019-1832-y.

91. Mi H, Muruganujan A, Ebert D, Huang X and Thomas PD. PANTHER version 14: more genomes, a new PANTHER GO-slim and improvements in enrichment analysis tools. Nucleic Acids Research. 2018;47 D1:D419-D26. doi:10.1093/nar/gky1038.

92. Katoh K and Standley DM. MAFFT multiple sequence alignment software version 7: improvements in performance and usability. Molecular biology and evolution. 2013;30 4:772-80.

93. Talavera G and Castresana J. Improvement of phylogenies after removing divergent and ambiguously aligned blocks from protein sequence alignments. Systematic biology. 2007;56 4:564-77.

94. Nguyen L-T, Schmidt HA, Von Haeseler A and Minh BQ. IQ-TREE: a fast and effective stochastic algorithm for estimating maximum-likelihood phylogenies. Molecular biology and evolution. 2015;32 1:268-74.

95. Kalyaanamoorthy S, Minh BQ, Wong TK, Von Haeseler A and Jermiin LS. ModelFinder: fast model selection for accurate phylogenetic estimates. Nature methods. 2017;14 6:587-9.

96. Kumar S, Stecher G, Suleski M and Hedges SB. TimeTree: A Resource for Timelines, Timetrees, and Divergence Times. Molecular biology and evolution. 2017;34 7:1812-9. doi:10.1093/molbev/msx116.

97. Yang Z. PAML: a program package for phylogenetic analysis by maximum likelihood. Computer applications in the biosciences : CABIOS. 1997;13 5:555-6.

98. Puttick MN. MCMCtreeR: functions to prepare MCMCtree analyses and visualize posterior ages on trees. Bioinformatics. 2019;35 24:5321-2.

99. Han MV, Thomas GWC, Lugo-Martinez J and Hahn MW. Estimating Gene Gain and Loss Rates in the Presence of Error in Genome Assembly and Annotation Using CAFE 3. Molecular Biology and Evolution. 2013;30 8:1987-97. doi:10.1093/molbev/mst100.

100. Buchfink B, Xie C and Huson DH. Fast and sensitive protein alignment using DIAMOND. Nature methods. 2015;12 1:59-60.

101. Tang H, Krishnakumar V, Li J and Zhang X. jcvi: JCVI utility libraries. Zenodo(doi: 105281/zenodo 31631). 2015.

102. Villanueva RAM and Chen ZJ. ggplot2: elegant graphics for data analysis. Taylor & Francis, 2019.

103. Quinlan AR. BEDTools: the Swiss-army tool for genome feature analysis. Current protocols in bioinformatics. 2014;47 1:11.2. 1-.2. 34.

104. Krzywinski M, Schein J, Birol I, Connors J, Gascoyne R, Horsman D, et al. Circos: an information aesthetic for comparative genomics. Genome research. 2009;19 9:1639-45.

105. Spearman Rank Correlation Coefficient. The Concise Encyclopedia

of Statistics. New York, NY: Springer New York; 2008. p. 502-5.

Figure legends

Fig. 1. Images of *Capparis spinosa*. A. Mature *C. spinosa* plant. **B.** Flowers. **C.** Fruits. **D.** Stem. **E.** Leaf tip.

Fig. 2. High-quality assembly of 21 chromosomes. A. Chromosome ideograms. **B.** Transposable element (TE) repeat sequence density (window size 100 kb). **C.** Tandem repeat sequence density (100 kb window size). **D.** Gene density (100 kb window size). **E.** GC content (100 kb window size). **F.** Relationship between syntenic blocks.

Fig. 3. Gene duplication and evolution of *Capparis spinosa*. A. Number of genes and gene pairs of five duplication types. **B.** Distribution of Ka/Ks of five duplication types. **C.** Distribution of Ks of five duplication types. **D.** Distribution of 4DTv of five duplication types.

Fig. 4. Distribution of Ks, 4DTv, and ages of LTR of *Capparis spinosa* and other species. A. Ks distribution of *C. spinosa* and other representative species. **B.** 4DTv distribution of *C. spinosa* and other representative species. **C.** Ages of LTR of *C. spinosa* and other species (molecular clock r is $7*10^{-9}$).

Fig. 5. Evolution of the *Capparis spinosa* genome. A. Venn diagram of specific and shared orthologs among 16 species (*O. sativa*, *B. distachyon*, *A. comosus*, *M. acuminata*, *C. micranthum*, *N. nucifera*, *T. sinense*, *V. vinifera*, *S. lycopersicum*, *P. trichocarpa*, *T. cacao*, *C. spinosa*, *T. hassleriana*, *A. thaliana*, *N. colorata*, and *A. trichopoda*) identified based on gene family cluster analysis. Each number in the diagram represents the number of gene families within a group. **B.** Expansion and contraction of gene families. **C.** GO enrichment analysis of genes from expanded families.

Figure S1. Genome size estimation of *C. spinosa* by using genome survey and flow cytometry with *Solanum pimpinellifolium* as reference.

A. The 17-mer distribution of Illumina short reads in *C. spinosa*. The x-axis shows the frequency or the number of times of a given k-mer (k-mer depth). The y-axis shows the total number of k-mers with a given frequency (a given depth). Two peaks (blue line) were observed indicating heterozygosity in *C. spinosa***. B.** Main peaks of *Solanum pimpinellifolium* and *Capparis spinosa* (samples 1 and 2) were 356.73 and 123.27 (mean value = (122.72 + 123.82)/2), respectively. According to the formula "peak (ref)/genome size (ref) = peak (*Capparis spinosa*)/genome size (*Capparis spinosa*)", the mean value of the genome size of *C. spinosa* was estimated as 276.44 Mb.

Figure S2. Hi-C interaction heat map. Hi-C heat map of 21 chromosomes.

Figure S3. Correlation analysis of genomic distribution characteristics.

Gene: gene density; GC: GC content; TR: distribution of tandem repeats; TE: distribution of transposable elements.

A. Correlation of the genomic GC content, gene density, TE distribution, and TR distribution. **B**. Correlation analysis of TR distribution and GC content.

Figure S4. Enrichment analysis of positively selected genes in gene duplication types. A. GO enrichment analysis of positively selected genes in four duplication types. **B.** KEGG enrichment analysis of positively selected genes in five duplication types.

Figure S5. *C. spinosa* genome collinearity analysis. A. Dot plots of paralogs in the *C. spinosa* genome. **B.** *A. trichopoda*, *C. spinosa*, and *A. thaliana* gene level collinearity analysis. **C.** *T. cacao* and *C. spinosa*

gene level collinearity analysis. **D.** *A. thaliana and C. spinosa* gene level collinearity analysis. **E.** *V. vinifera*, *C. spinosa*, and *S. lycopersicum* genome level collinearity analysis.

Figure S6. GO and KEGG enrichment analysis. A. GO enrichment analysis of *C. spinosa* specific genes (biological process). **B.** KEGG enrichment analysis of expansion genes (Genes with Ka/Ks >1 were positively selected genes).

Click here to access/download;Figure;Figure 1.pdf

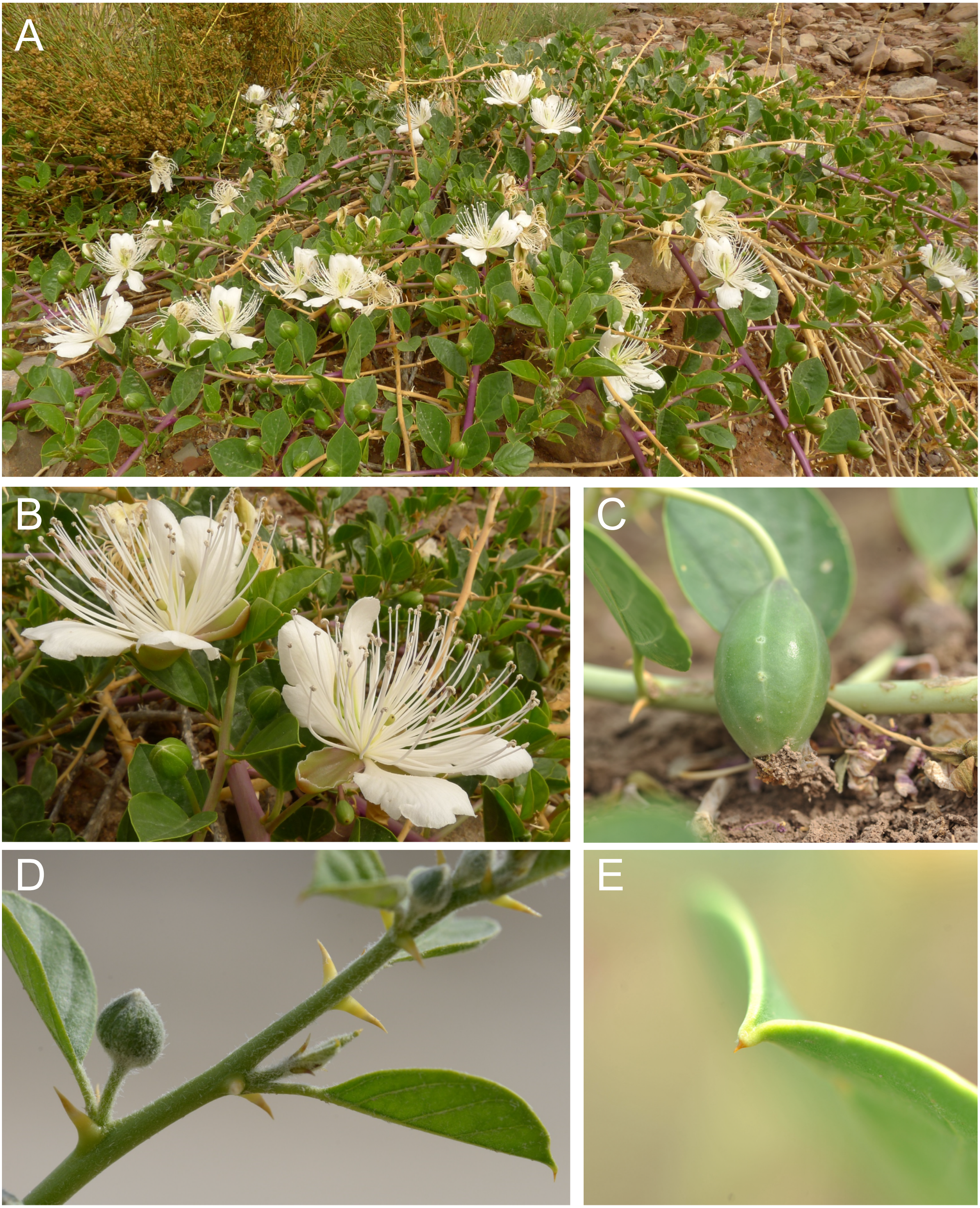

figure 2

Click here to access/download;Figure;Figure 2.pdf

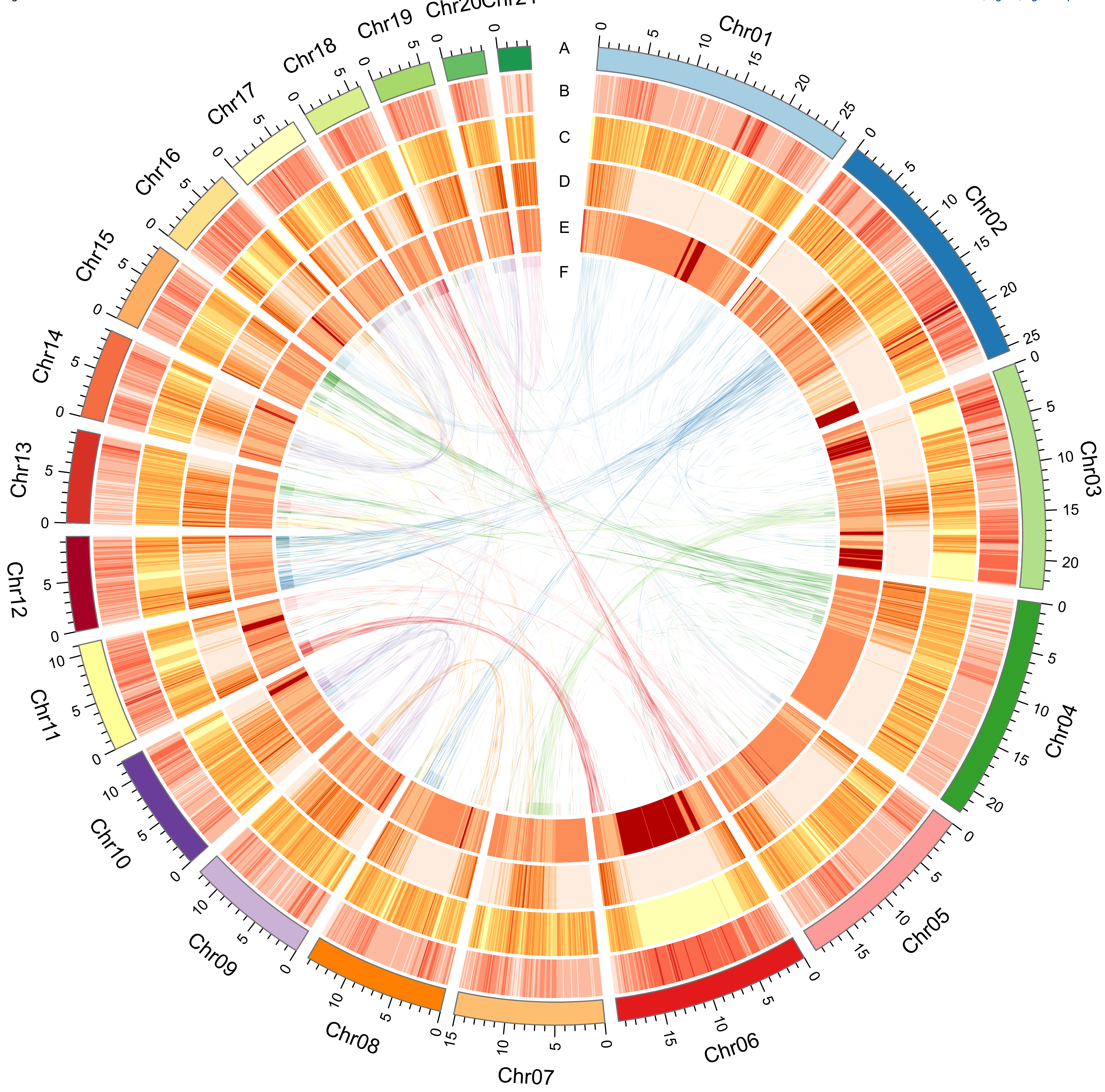

Figure 3

Click here to access/download;Figure;Figure 3.pdf

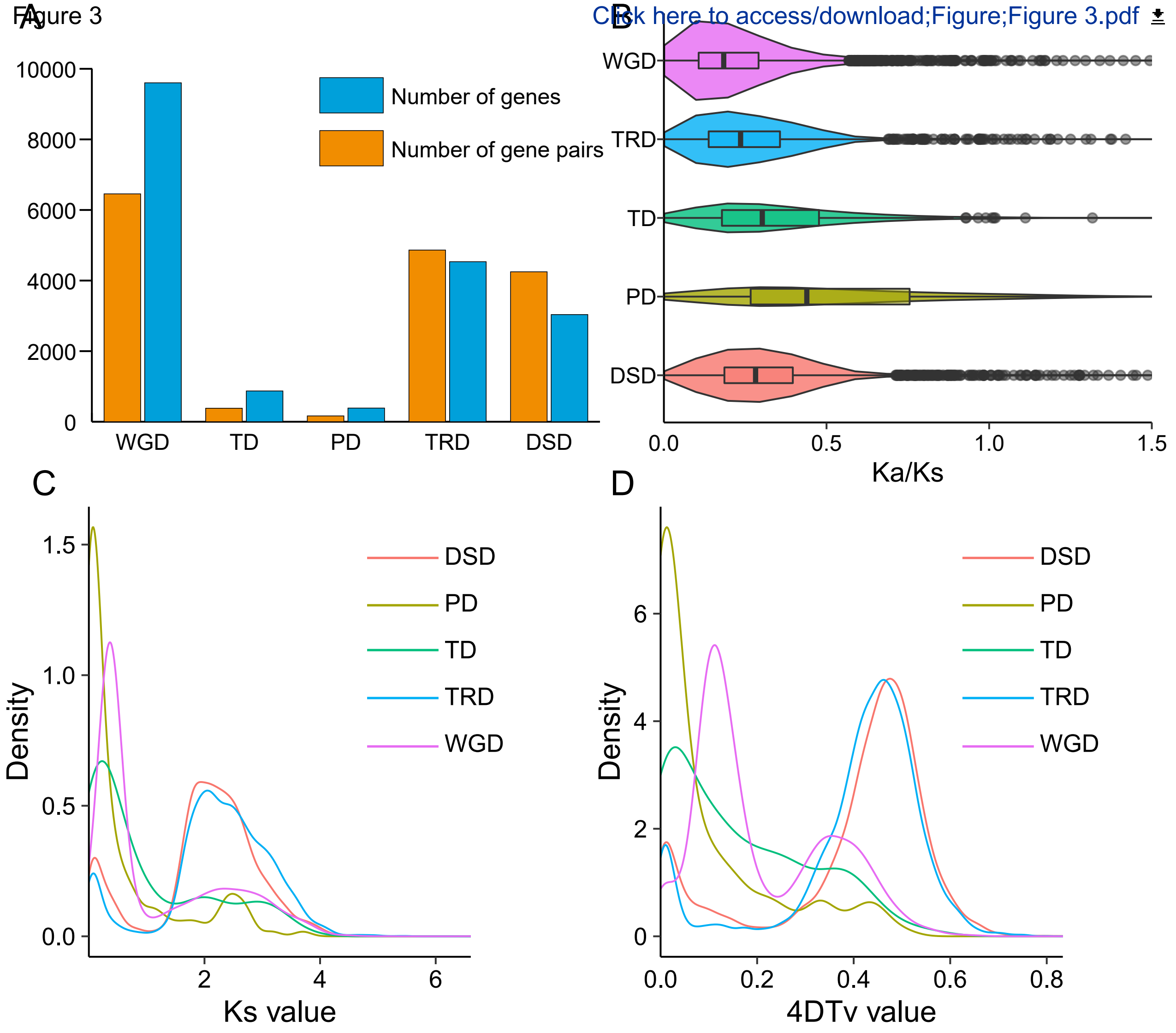

Figure 4

Click here to access/download;Figure;Figure 4.pdf

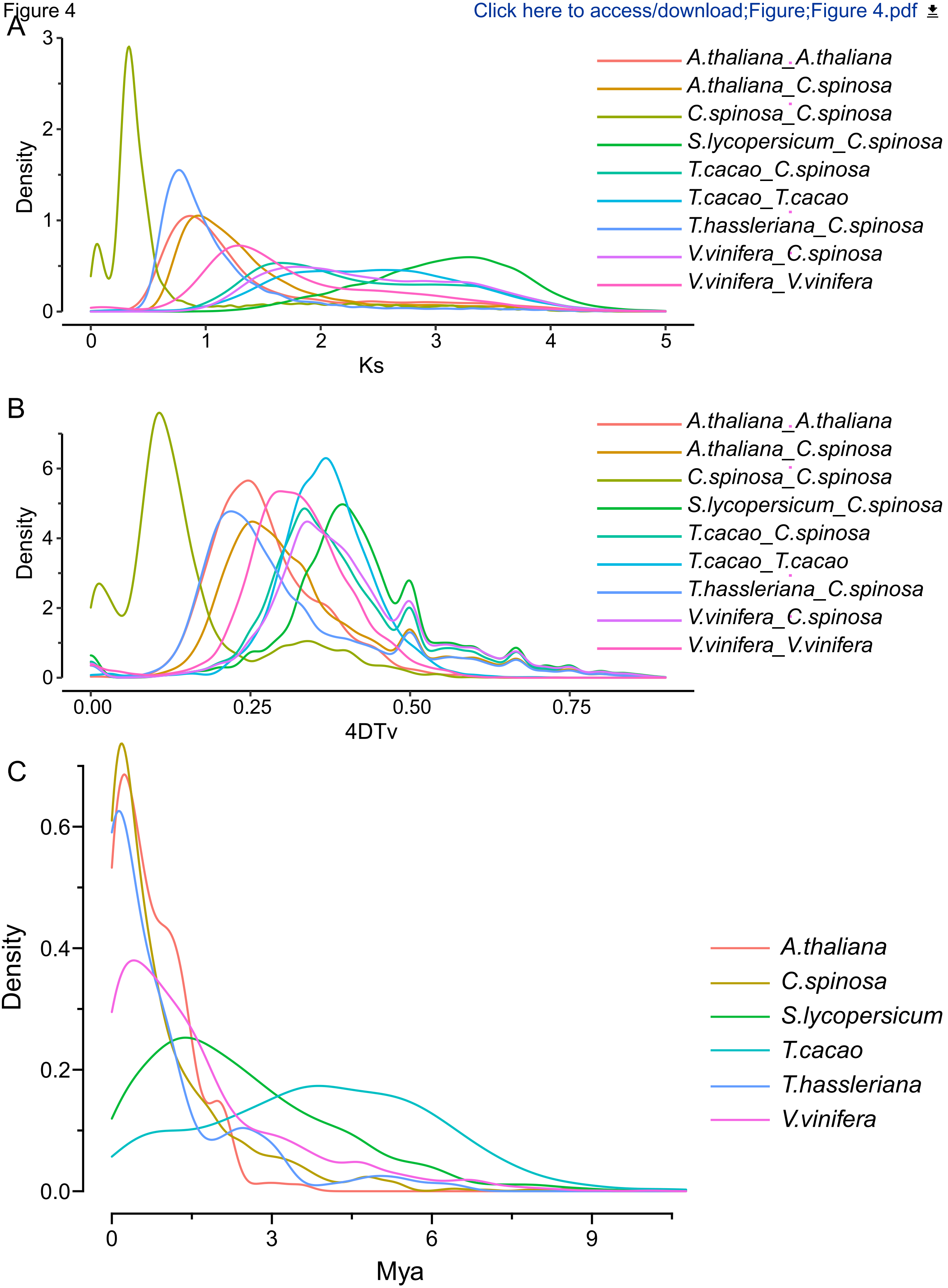

Figure 5

Click here to access/download;Figure;Figure 5.pdf

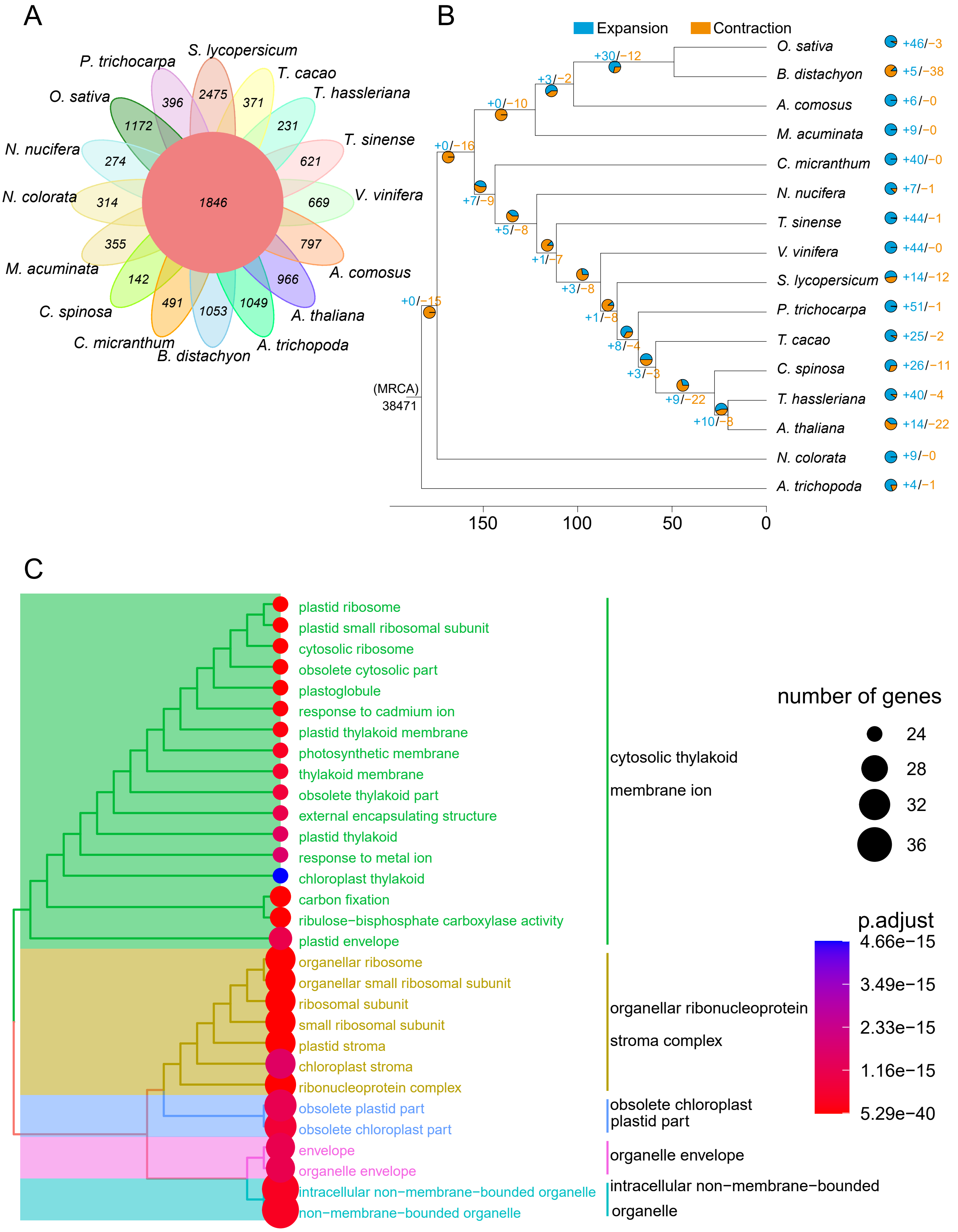

Supplementary Figure

Click here to access/download
Supplementary Material
Supply Figure.docx

Supplementary Table

Click here to access/download
Supplementary Material
Supplyment Tables.docx

Manuscript modify version

Click here to access/download
Supplementary Material
manuscript_modify.pdf
