# Supplementary material for: The Capparis spinosa var. herbacea genome provides the first genomic instrument for a diversity and evolution study of the Capparaceae family
Source: Gigascience. 2022 Oct 30;11:giac106. doi: 10.1093/gigascience/giac106 (PMC9618406; doi:10.1093/gigascience/giac106)
Supplement: giac106_Supplemental_Files [file giac106_supplemental_files.zip › Supply Figure.docx]

**The** ***Capparis spinosa* *var. herbacea* genome provides insight into genome evolution of Capparaceae**

## Supply Figure


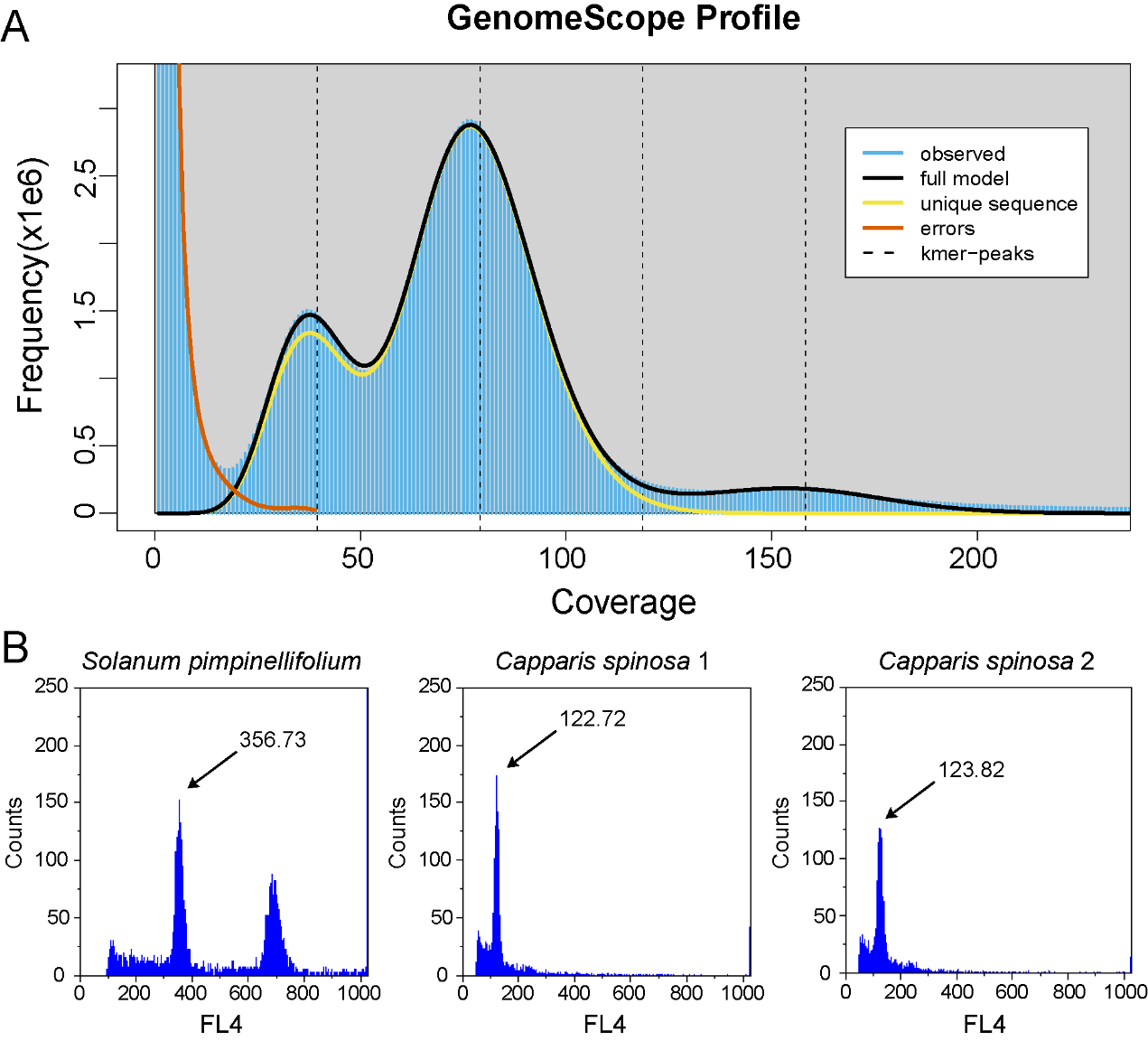


# Figure S1. Genome size estimation of *C. spinosa var. herbacea* by using genome survey and flow cytometry with *Solanum pimpinellifolium* as reference.

**A.** The 17-mer distribution of Illumina short reads in *C. spinosa var. herbacea*. The x-axis shows the frequency or the number of times of a given k-mer (k-mer depth). The y-axis shows the total number of k-mers with a given frequency (a given depth). Two peaks (blue line) were observed indicating heterozygosity in *C. spinosa var. herbacea***. B.** Main peaks of *Solanum pimpinellifolium* and *Capparis spinosa* *var. herbacea* (samples 1 and 2) were 356.73 and 123.27 (mean value = (122.72 + 123.82)/2), respectively. According to the formula “peak (ref)/genome size (ref) = peak (*Capparis spinosa var. herbacea*)/genome size (*Capparis spinosa var. herbacea*)”, the mean value of the genome size of *C. spinosa var. herbacea* was estimated as 279.07 Mb.


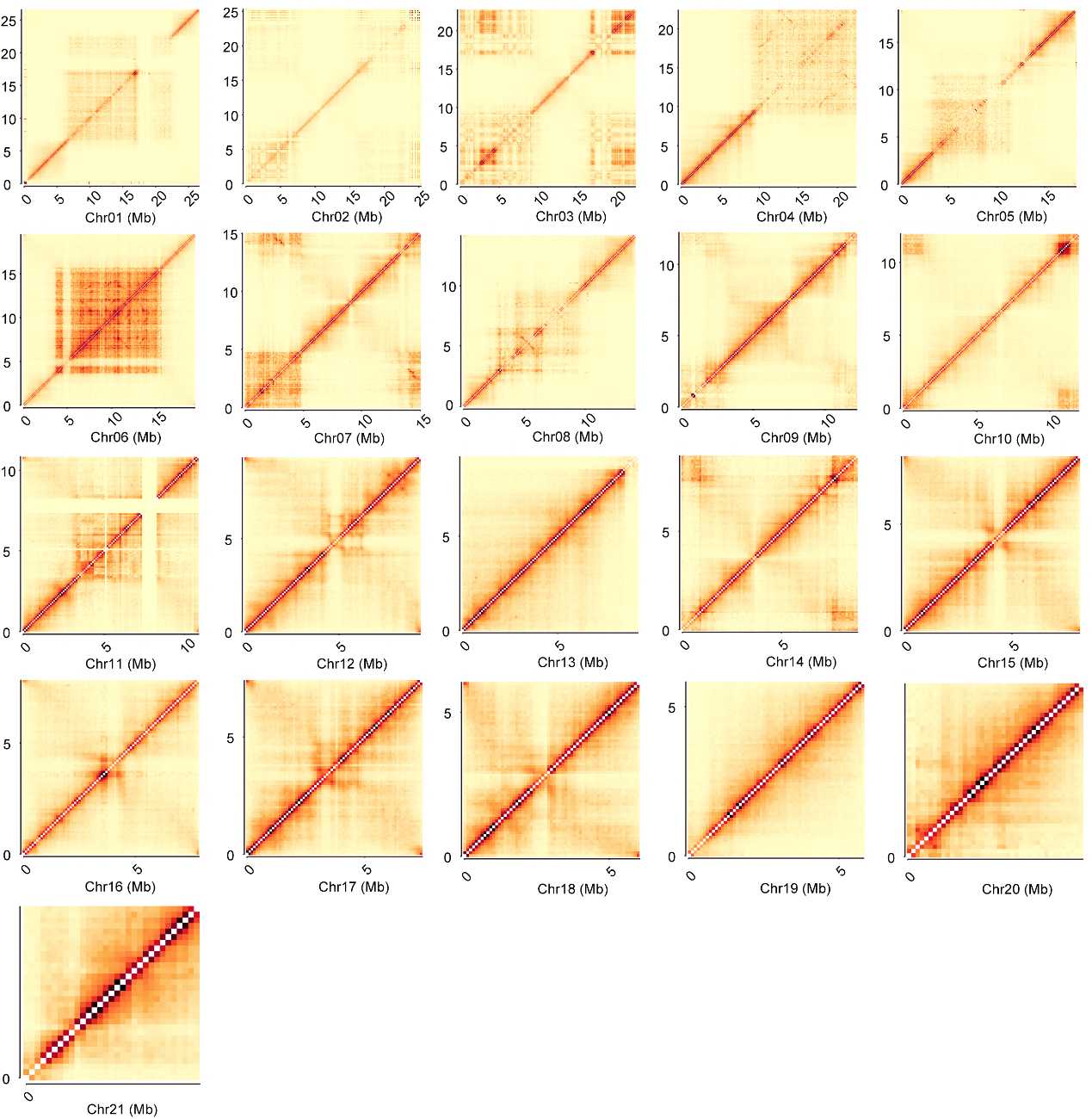


# Figure S2 Hi-C interaction heat map.

The 21 chromosomes Hi-C heat map.


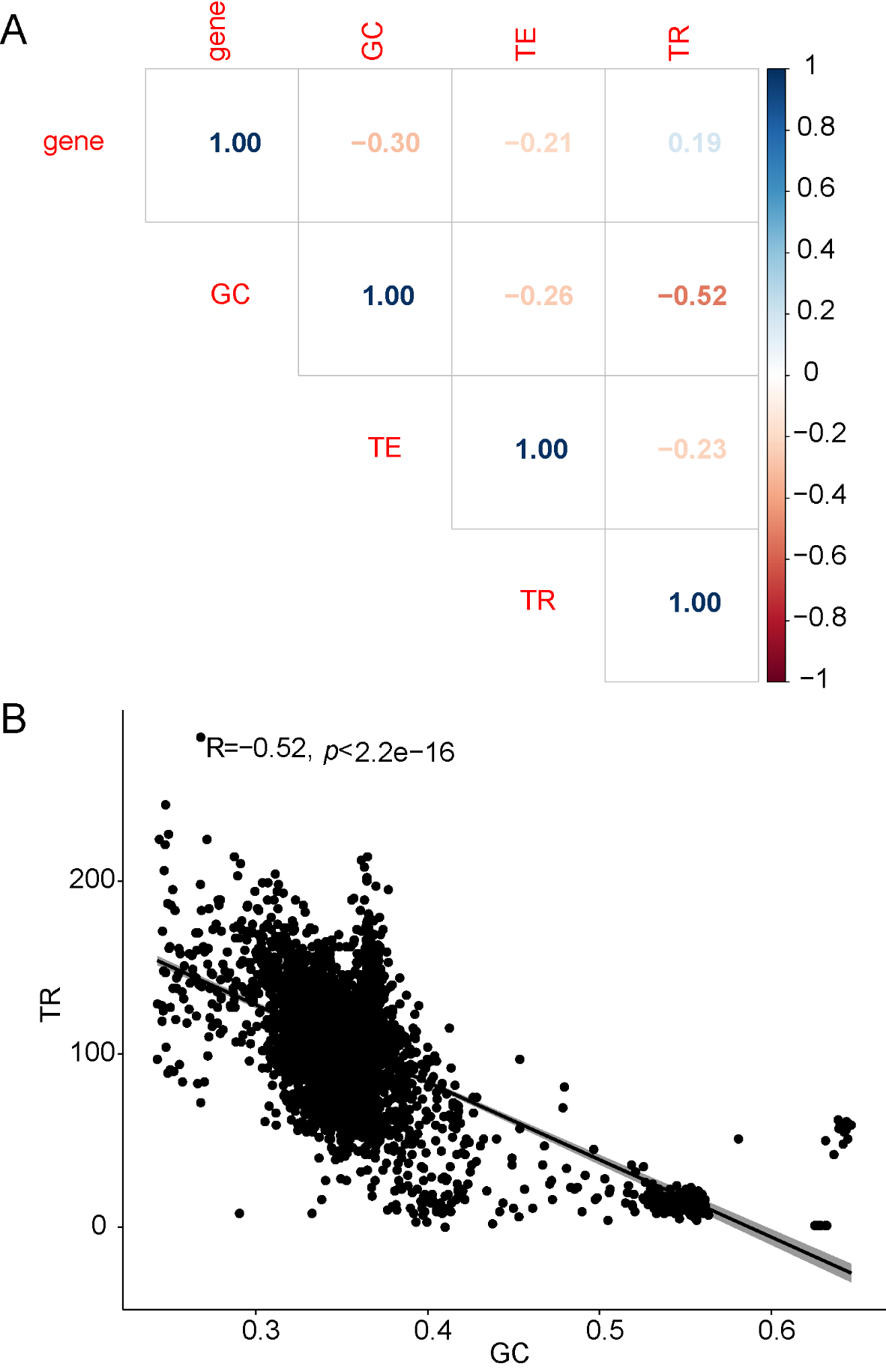


# Figure S3. Correlation analysis of genomic distribution characteristics.

Gene: gene density; GC: GC content; TR: distribution of tandem repeats; TE: distribution of transposable elements.

**A**. Correlation of the genomic GC content, gene density, TE distribution, and TR distribution. **B**. Correlation analysis of TR distribution and GC content.


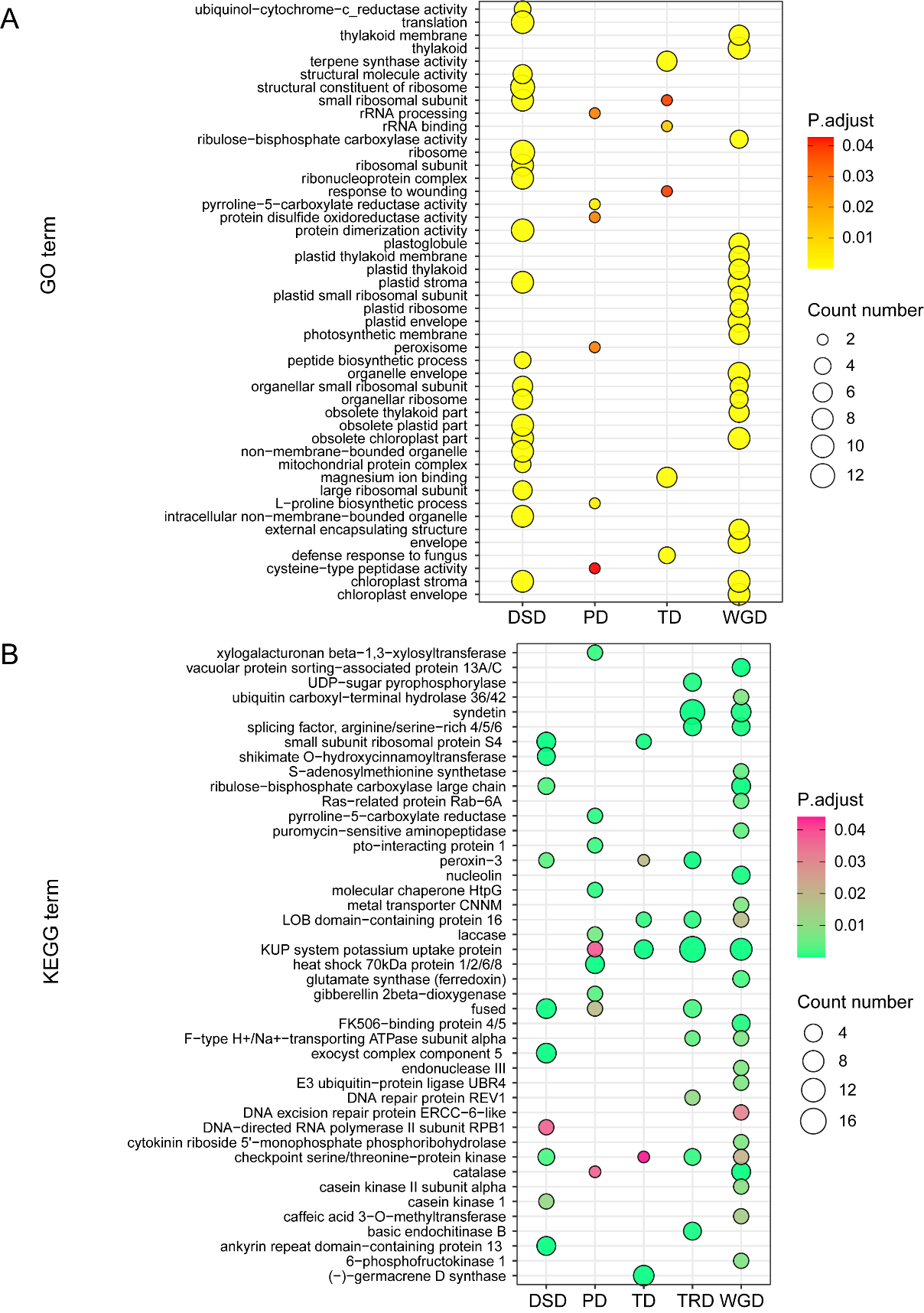


# Figure S4. Enrichment analysis of positively selected genes in gene duplication types.

**A.** GO enrichment analysis of positively selected genes in four duplication types. **B.** KEGG enrichment analysis of positively selected genes in five duplication types.


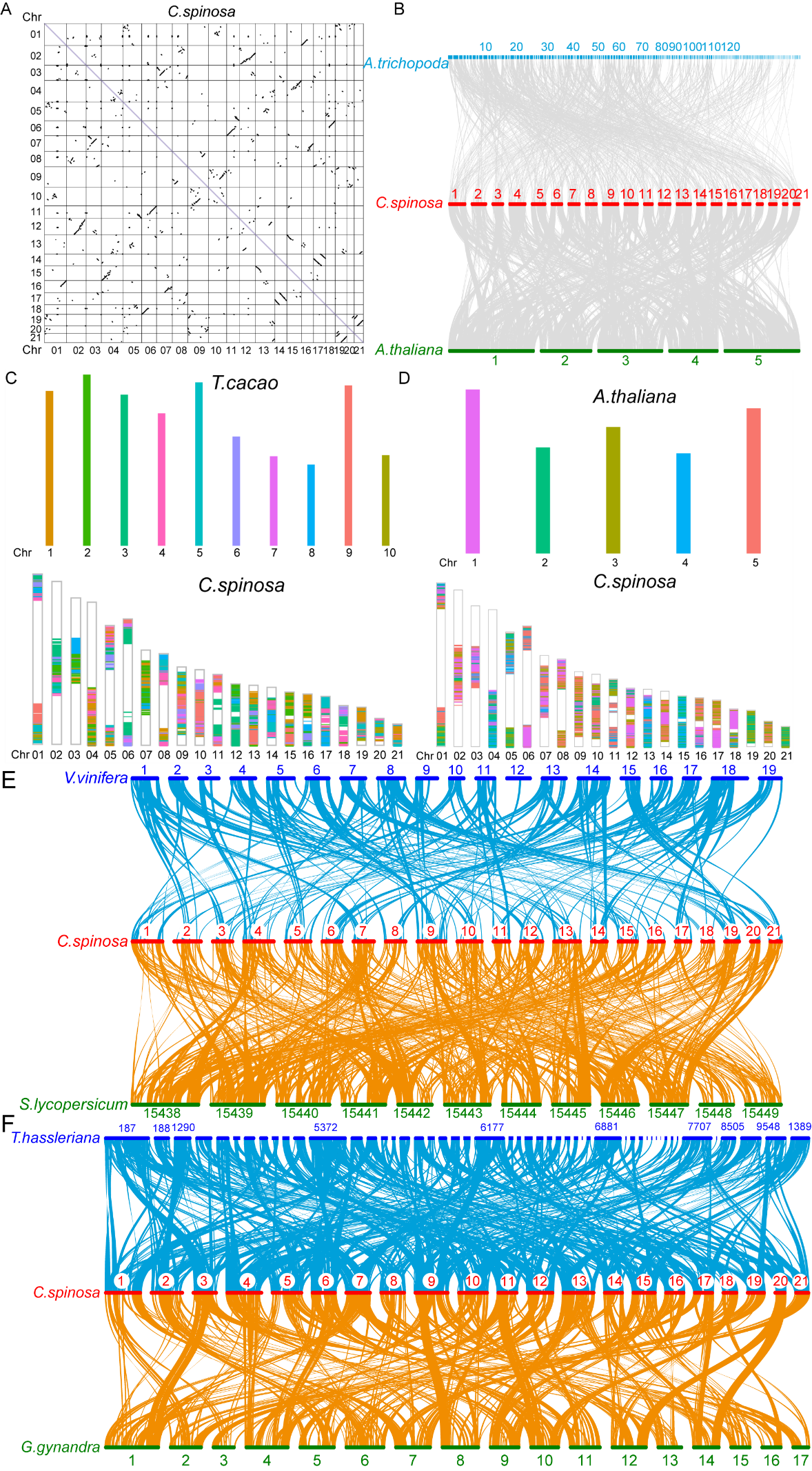


# Figure S5. *C. spinosa var. herbacea* genome collinearity analysis.

**A.** Dot plots of paralogs in the *C. spinosa var. herbacea* genome. **B.** *A. trichopoda*, *C. spinosa var. herbacea*, and *A. thaliana* gene level collinearity analysis. **C.** *T. cacao* and *C. spinosa* *var. herbacea* gene level collinearity analysis. **D.** *A. thaliana and C. spinosa var. herbacea* gene level collinearity analysis. **E.** *V. vinifera*, *C. spinosa var. herbacea*, and *S.* *lycopersicum* genome block level collinearity analysis. **F**. *T.hassleriana*, *C.spinosa var. herbacea*, and *G.* *gynandra* genome block level collinearity analysis(The *T.hassleriana* genome uses the longest Top 50 scaffolds. The order shown in the figure is the sequence numbering order after Top50 is extracted.).


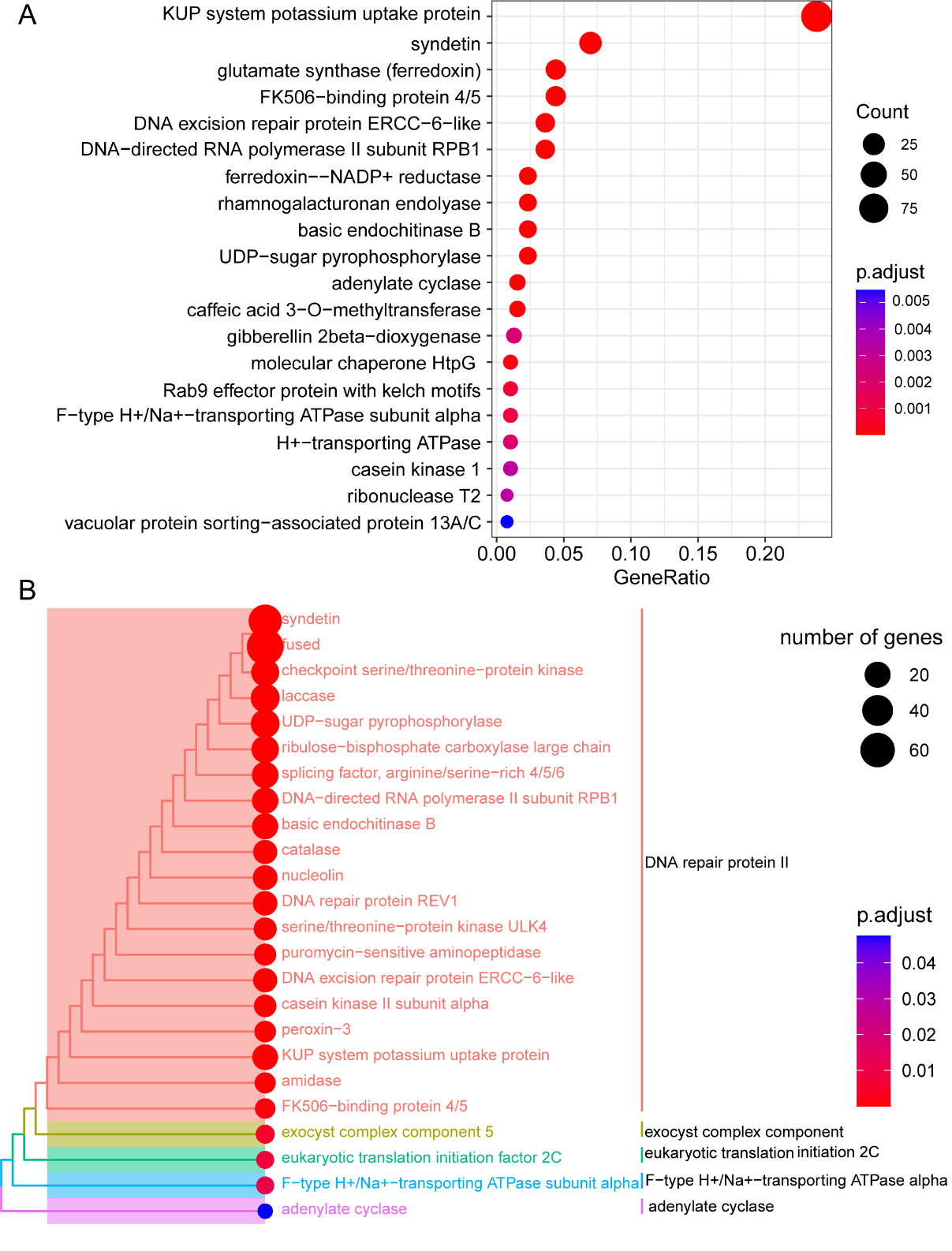


# Figure S6. KEGG enrichment analysis.

**A.** KEGG enrichment analysis of *C. spinosa* var. *herbacea* specific genes. **B.** KEGG enrichment analysis of expansion genes.
