# Supplementary material for: The Capparis spinosa var. herbacea genome provides the first genomic instrument for a diversity and evolution study of the Capparaceae family
Source: Gigascience. 2022 Oct 30;11:giac106. doi: 10.1093/gigascience/giac106 (PMC9618406; doi:10.1093/gigascience/giac106)
Supplement: giac106_Supplemental_Files [file giac106_supplemental_files.zip › Supplyment Tables.docx]

**The** ***Capparis spinosa* var. herbacea genome provides insight into genome evolution of Capparaceae**

Lei Wang^a,b,1^, Liqiang Fan^c,d,1^, Zhenyong Zhao^a,b^, Zhibin Zhang^c,d^, Li Jiang^a,b^, Mao Chai^c,d,*^ and Changyan Tian^a,b,*^

# Table S1. Genome assembly statistic information for *C.* *spinosa* var. *herbacea.*

|  | **Type** | **Size(bp)** | **Number** |
| --- | --- | --- | --- |
| scaffold | N10 | 25,493,351 | 2 |
|  | N20 | 22,916,255 | 3 |
|  | N30 | 22,507,087 | 4 |
|  | N40 | 19,687,438 | 5 |
|  | N50 | 15,147,718 | 7 |
|  | N60 | 14,276,932 | 8 |
|  | N70 | 11,036,859 | 11 |
|  | N80 | 9,363,759 | 13 |
|  | N90 | 7,609,129 | 17 |
|  | smallest | 25,179 |  |
|  | longest | 26,663,249 |  |
| contig | N10 | 19,687,438 | 2 |
|  | N20 | 14,525,310 | 3 |
|  | N30 | 14,276,932 | 5 |
|  | N40 | 12,959,451 | 7 |
|  | N50 | 9,363,759 | 10 |
|  | N60 | 8,296,278 | 13 |
|  | N70 | 6,169,771 | 17 |
|  | N80 | 5,418,639 | 21 |
|  | N90 | 3,897,678 | 27 |
|  | smallest | 25,179 |  |
|  | longest | 22,507,087 |  |
| Illumina | Total_reads | 221,078,842 |  |
|  | Mapped_reads | 217,799,051 |  |
|  | Mapped (%) | 98.52 |  |
|  | Properly_mapped_reads | 211,370,050 |  |
|  | Properly_mapped | 95.61% |  |
| PacBio | Reads_num | 1,531,982 |  |
|  | Reads_base | 25.46Gb |  |
|  | Reads_LenN50 | 17.00Kb |  |
|  | Reads_LenMean | 16.62Kb |  |
|  | Reads_LenMax | 42.12Kb |  |
|  | Contig number | 45 |  |
|  | Contig length | 274.53Mb |  |
|  | Contig N50 | 11.04Mb |  |
|  | Contig N90 | 5.21Mb |  |
|  | Contig max | 22.51Mb |  |
|  | GC content | 36.61% |  |

# Table S2. BUSCO evaluation result.

| **Type** | **Genome** |
| --- | --- |
| Complete BUSCOs(C) | 1562 (96.80%) |
| Complete and single-copy BUSCOs(S) | 1497 (92.80%) |
| Complete and duplicated BUSCOs(D) | 65 (4.00%) |
| Fragmented BUSCOs(F) | 7 (0.40%) |
| Missing BUSCOs(M) | 45 (2.8%) |
| Total Lineage BUSCOs | 1614 |

# Table S3. Repeat sequence.

| **Type** | **Number** | **Length** | **Rate (%)** |
| --- | --- | --- | --- |
| ClassI:Retroelement | 39,450 | 85,757,803 | 31.24 |
| ClassI/DIRS | 262 | 12,878 | 0 |
| ClassI/LINE | 7,315 | 2,505,821 | 0.91 |
| ClassI/LTR/Cassandra | 32 | 5,741 | 0 |
| ClassI/LTR/Caulimovirus | 424 | 1,239,783 | 0.45 |
| ClassI/LTR/Copia | 9,976 | 29,749,806 | 10.84 |
| ClassI/LTR/ERV | 4,162 | 422,464 | 0.15 |
| ClassI/LTR/Gypsy | 9,276 | 11,447,091 | 4.17 |
| ClassI/LTR/Pao | 479 | 35,212 | 0.01 |
| ClassI/LTR/Unknown | 6,108 | 40,162,543 | 14.63 |
| ClassI/LTR/Viper | 19 | 2,542 | 0 |
| ClassI/SINE | 1,397 | 173,922 | 0.06 |
| ClassII:DNA transposon | 40,660 | 34,990,312 | 12.75 |
| ClassII/Academ | 20 | 2,896 | 0 |
| ClassII/CACTA | 3,814 | 7,034,814 | 2.56 |
| ClassII/Crypton | 487 | 44,810 | 0.02 |
| ClassII/Dada | 323 | 72,224 | 0.03 |
| ClassII/Ginger | 198 | 12,242 | 0 |
| ClassII/Helitron | 607 | 254,383 | 0.09 |
| ClassII/IS3EU | 198 | 11,171 | 0 |
| ClassII/Kolobok | 310 | 18,127 | 0.01 |
| ClassII/MITE | 24 | 1,889 | 0 |
| ClassII/Maverick | 351 | 20,538 | 0.01 |
| ClassII/Merlin | 51 | 7,236 | 0 |
| ClassII/Mutator | 1,389 | 330,253 | 0.12 |
| ClassII/Novosib | 263 | 14,948 | 0.01 |
| ClassII/P | 276 | 15,448 | 0.01 |
| ClassII/PIF-Harbinger | 851 | 624,204 | 0.23 |
| ClassII/PiggyBac | 330 | 20,095 | 0.01 |
| ClassII/Sola | 132 | 10,105 | 0 |
| ClassII/Tc1-Mariner | 1,951 | 299,390 | 0.11 |
| ClassII/Unknown | 22,064 | 22,567,797 | 8.22 |
| ClassII/Zator | 26 | 1,419 | 0 |
| ClassII/Zisupton | 309 | 19,815 | 0.01 |
| ClassII/hAT | 6,686 | 3,606,508 | 1.31 |
| Total | 80,110 | 120,748,115 | 43.98 |

# Table S4. Genome encoding gene prediction

| Method | Software | Species | Gene number |
| --- | --- | --- | --- |
| Ab initio | Augustus | - | 20,608 |
|  | SNAP | - | 36,037 |
| Homology-based | GeMoMa | *A. thaliana* | 22,459 |
|  |  | *C. sativa* | 25,756 |
|  |  | *E. salsugineum* | 22,617 |
|  |  | *T. hassleriana* | 23,747 |
| RNAseq | GeneMarkS-T | - | 16,655 |
|  | PASA | - | 11,132 |
| Integration | EVM | - | 21,577 |

# Table S5. Gene’s annotation.

| **Anno_Database** | **Annotated_Number** | **Annotated_Ratio** |
| --- | --- | --- |
| GO_Annotation | 18,238 | 84.53 |
| KEGG_Annotation | 16,521 | 76.57 |
| KOG_Annotation | 12,801 | 59.33 |
| Pfam_Annotation | 18,934 | 87.75 |
| Swissprot_Annotation | 18,288 | 84.76 |
| TrEMBL_Annotation | 21,281 | 98.63 |
| eggNOG_Annotation | 18,967 | 87.9 |
| nr_Annotation | 21,295 | 98.69 |
| All_Annotated | 21,323 | 98.82 |

# Table S6. Duplication gene's distribution.

| **Duplication type** | **Number of gene pairs** | **Number of genes** | **Percentage of number of gene pairs** | **Percentage of number of genes** |
| --- | --- | --- | --- | --- |
| whole-genome duplication (WGD) | 6,456 | 9,603 | 40.1% | 52.1% |
| tandem duplication (TD) | 382 | 872 | 2.4% | 4.7% |
| proximal duplication (PD) | 165 | 387 | 1.0% | 2.1% |
| transposed duplication (TRD) | 4,864 | 4,534 | 30.2% | 24.6% |
| dispersed duplication (DSD) | 4,248 | 3,036 | 26.4% | 16.5% |

# Table S7. Genome synteny analysis results

| Group | colinear gene pairs | colinear blocks |
| --- | --- | --- |
| *A.thaliana_C. spinosa* var. *herbacea* | 19,063 | 409 |
| *A. trichopoda_C. spinosa* var. *herbacea* | 5,501 | 17 |
| *T. cacao_C. spinosa* var. *herbacea* | 16,747 | 260 |
| *V. vinifera_C. spinosa* var. *herbacea* | 15,352 | 222 |
| *T.hassleriana_C. spinosa var. herbacea* | 24,456 | 519 |
| *G.gynandra_C. spinosa var. herbacea* | 13,366 | 346 |
| *S. lycopersicum_C. spinosa* var. *herbacea* | 17,940 | 502 |

# Table S8. Expansion and specific genes KEGG enrichment results

See in excel file TableS8.expansion and specific genes kegg enrichment results.xlsx

# Table S9. Download URLs for reference genomes of other species

| Species | download address |
| --- | --- |
| *Amborella trichopoda* | https://www.ncbi.nlm.nih.gov/assembly/GCF_000471905.2 |
| *Nymphaea colorata* | https://www.ncbi.nlm.nih.gov/assembly/GCF_008831285.1 |
| *Cinnamomum micranthum* | https://www.ncbi.nlm.nih.gov/assembly/GCA_003546025.1/ |
| *Musa acuminata* | https://www.ncbi.nlm.nih.gov/genome/10976?genome_assembly_id=262489 |
| *Ananas comosus* | https://www.ncbi.nlm.nih.gov/genome/13945?genome_assembly_id=271638 |
| *Oryza sativa* | ftp://ftp.gramene.org/pub/gramene/release-61/fasta/oryza_sativa/dna/Oryza_sativa.IRGSP-1.0.dna.toplevel.fa.gz |
| *Brachypodium distachyon* | https://phytozome.jgi.doe.gov/pz/portal.html#!info?alias=Org_Bdistachyon |
| *Nelumbo nucifera* | http://nelumbo.biocloud.net/nelumbo/download/download |
| *Tetracentron sinense* | https://www.ncbi.nlm.nih.gov/assembly/GCA_015143295.1/ |
| *Arabidopsis thaliana* | https://www.ncbi.nlm.nih.gov/assembly/GCF_000001735.4 |
| *Populus trichocarpa* | https://www.ncbi.nlm.nih.gov/assembly/GCF_000002775.4 |
| *Vitis vinifera* | ftp://ftp.ensemblgenomes.org/pub/plants/release-25/fasta/vitis_vinifera/ |
| *Theobroma cacao* | https://www.ncbi.nlm.nih.gov/assembly/GCF_000208745.1 |
| *Solanum lycopersicum* | https://www.ncbi.nlm.nih.gov/assembly/GCF_000188115.5 |
| *Tarenaya hassleriana* | https://www.ncbi.nlm.nih.gov/assembly/GCF_000463585.1/ |
| *Gynandropsis gynandra* | https://genomevolution.org/coge/GenomeInfo.pl?gid=58728 |
