# Supplementary material for: The Capparis spinosa var. herbacea genome provides the first genomic instrument for a diversity and evolution study of the Capparaceae family
Source: Gigascience. 2022 Oct 30;11:giac106. doi: 10.1093/gigascience/giac106 (PMC9618406; doi:10.1093/gigascience/giac106)
Supplement: giac106_GIGA-D-22-00058_Revision_3 [file giac106_giga-d-22-00058_revision_3.pdf]

GigaScience

T he Capparis spinosa var. herbacea genome provides the first genomic instrument for diversity and evolution study of Capparaceae family

--Manuscript Draft--

**Manuscript Number:**	GIGA-D-22-00058R3
**Full Title:**	T he Capparis spinosa var. herbacea genome provides the first genomic instrument for diversity and evolution study of Capparaceae family
**Article Type:**	Data Note
**Funding Information:**	National Key Research and Development Program of China (2018YFE0207200) — Dr. Changyan Tian
**Abstract:**	Capparis spinosa L., one of the most economically important species of Capparaceae, is a xerophytic shrub that is well adapted to drought and harsh environments. However, genetic studies on this species are limited because of the lack of its reference genome.<br>We sequenced and assembled the Capparis spinosa var. herbacea (Willd.) (NCBI:txid2717819) genome using data obtained from the combination of PacBio circular consensus sequencing and high-throughput chromosome conformation capture. The final genome assembly was approximately 274.53 Mb (contig N50 length of 9.36 Mb, scaffold N50 of 15.15 Mb), 99.23% of which was assigned to 21 chromosomes. In the whole-genome sequence, tandem repeats accounted for 19.28%, and transposable element sequences accounted for 43.98%. The proportion of tandem repeats in the C. spinosa var. herbacea genome was much higher than the average of 8.55% in plant genomes. A total of 21,577 protein-coding genes were predicted, with 98.82% being functionally annotated. The result of species divergence times showed that C. spinosa var. herbacea and Tarenaya hassleriana separated from a common ancestor 43.31 MYA.<br>This study reported a high-quality reference genome assembly and genome features for the Capparaceae family. The assembled C. spinosa var. herbacea genome might provide a system for studying the diversity, speciation, and evolution of this family, and serve as an important resource for understanding the mechanism of drought and high-temperature resistance.
**Corresponding Author:**	Mao Chai<br>Chinese Academy of Agricultural Sciences Cotton Research Institute<br>Zhengzhou, Henan CHINA
**Corresponding Author Secondary Information:**	
**Corresponding Author's Institution:**	Chinese Academy of Agricultural Sciences Cotton Research Institute
**Corresponding Author's Secondary Institution:**	
**First Author:**	Lei Wang
**First Author Secondary Information:**	
**Order of Authors:**	Lei Wang
	Liqiang Fan
	Zhenyong Zhao
	Zhibin Zhang
	Li Jiang
	Mao Chai
	Changyan Tian
**Order of Authors Secondary Information:**	

Powered by Editorial Manager® and ProduXion Manager® from Aries Systems Corporation

**Response to Reviewers:**	Dear Editors and Reviewers:<br>GIGA-D-22-00058R2<br>The Capparis spinosa var. herbacea genome provides insight into Capparaceae genome evolution<br>Lei Wang; Liqiang Fan; Zhenyong Zhao; Zhibin Zhang; Li Jiang; Mao Chai; Changyan Tian<br>GigaScience<br><br>In this version of revised manuscript, we have addressed carefully all the concerns of reviewer 1 point by point, mainly including the new addition of comparative genome analysis of T.hassleriana and G.gynandra, and the redrawing of Fig4A and B to make the lines more easily distinguishable. Besides, we have redrawn FigS5E and added FigS5F, both drawn with collinear blocks. The number of collinear gene pairs and blocks of newly added species has been added to TableS7. The data download address for the species involved in the comparative genomes analysis have been added to TableS9. We hope our revisions will meet the expectation of editor and reviewer.<br><br>Reviewer reports:<br>Reviewer #1: The authors improved the paper following the suggestions provided in the last revision. However there are still some lacks.<br>The authors explained how the genome size estimation has been carried out, using the contigs assembly from Wang et al. 2020 (807.6 Mb). I am not completely agree, but the choice can be accepted.<br>The description about annotation was unclear in the previous version and, probably, with some missing data. Now the authors used RNASeq data for the genes prediction (in my opinion new data, because all information about this point - methods, results, discussion - were previously missed in all sections). Unfortunately the section reported for genes prediction and annotation ("Genome coding gene prediction and annotation") are still poor. The authors have to add some additional information, that can be used also in the discussion.<br>Reply: Thank you very much! We have added a description of the PacBio full-length transcripts analysis method to the Materials Methods section, as follows: "A total of 1.52Gb of PacBio transcriptome data was used for full-length transcriptome sequence analysis using PacBio Iso Seq3." Please see lines 396-397 in the revised manuscript.<br><br>In addition, I suggest to the authors to change the title. I am not agree about the possibility that the paper is able to "provides insight into Capparaceae genome evolution". The authors describe the first genome in the C. spinosa group without strong comparison in its and/or sister families (due to also the lack of information available). Therefore, although this study is an interest beginning point, that provides new information for future studies, the title is pretentious. The authors need to change it in (e.g.) "The Capparis spinosa var. herbacea genome provides the first genomic instrument for diversity and evolution study of Capparaceae family"…or something similar.<br>Reply: Thank you very much for your suggestion. We believe that your suggestion is well suited to this study and therefore changed the title to “The Capparis spinosa var. herbacea genome provides the first genomic instrument for diversity and evolution study of Capparaceae family”.<br><br>The authors have to improve the Background section, adding some information about the distribution of C. spinosa var herbacea in the world, in order to justify why the authors have chosen this variety.<br>Reply: Thank you very much. We have added a description of the worldwide distribution of C. spinosa var herbacea, as follows: "C. spinosa var. herbacea is mainly distributed in Turkey, Kazakhstan, Uzbekistan, Afghanistan and western China [7]. According to the morphological characteristics of China reported by previous researchers [5], C. spinosa var. herbacea is mainly distributed in Xinjiang, Tibet and Gansu in China." Please see lines 57-60 in the revised manuscript.<br><br>In the "Analyses of genome synteny and WGD" (line 161-172), using the species compared the results obtained is obvious ("The syntenic analysis results also showed more collinear gene pairs between C. spinosa var. herbacea and A. thaliana (Table S7), indicating that C. spinosa var. herbacea has a close evolutionary relationship with

	A. thaliana"). In my opinion to have more information the authors have to add in the same analysis also T. hassleriana (in this version it is used only for LTR insertion evaluation) and Gynandropsis gynandra, both belonging to Cleomaceae. The last one should be also added at the gene family expansion and contraction evaluation and in the phylogenetic analysis and species divergence time estimation.<br>Reply: Thank you very much for your suggestion. We added the collinear analysis of T. hassleriana vs C. spinosa var. herbacea and Gynandropsis gynandra vs C. spinosa var. herbacea to FigS5F. At the same time, the Ks and 4DTv analysis of Gynandropsis gynandra vs C. spinosa var. herbacea was newly added to Fig4 A-B and the peak value of Ks was used to evaluate the diverge time. A description of the above results is shown below "The results showed that C. spinosa var. herbacea separated from T. cacao, G. gynandra, A. thaliana, and T. hassleriana in order at 93.10 MYA (Ks peak of 1.644 and 4DTv peak of 0.336), 58.46 MYA (Ks peak of 1.032 and 4DTv peak of 0.266), 53.00 MYA (Ks peak of 0.936 and 4DTv peak of 0.254), and 43.31 MYA (Ks peak of 0.765 and 4DTv peak of 0.220). Besides, C. spinosa var. herbacea experienced an α WGD event at 18.59 MYA (Ks peak at 0.328).". Please see lines 181-189 in the revised manuscript.<br>Our newly added Ks, 4DTv and collinearity analysis showed that the divergence of C. spinosa var. herbacea from T.hassleriana and A.thaliana occurred later than that of C. spinosa var. herbacea and G.gynandra. This indicates a closer evolutionary relationship between T.hassleriana, A.thaliana and C. spinosa var. herbacea.<br><br>Finally, in the "Data availability" all links included in this version don't work. In addition the sequences obtained from transcriptome (both long and short reads) has not been deposited and/or the link has not been reported. The authors must to provide all information/data/results developed (this is mandatory).<br>Reply: Thank you very much! Due to the long review cycle, some of the links initially provided may not be accessible. Here we re-provide the latest accessible links. We will release the data once the manuscript is accepted. Until then, the data access links in the manuscript are temporarily unavailable. You can view the data using the reviewer link we have provided. In the revised manuscript, we have added SRR numbers for specific data, as follows: "Raw data of genome assembly (PacBio HiFi and Hi-C sequences) and genome encoding gene prediction annotations (Illumina and PacBio sequences) have been deposited in the NCBI Sequence Read Archive (SRA) database under Bioproject ID: PRJNA792936. Illumina data SRR18458236 for genome size survey. Data for genome PacBio HiFi data SRR17373648, Hi-C data SRR17373647. For encoding gene prediction annotations Illumina data SRR18512706, PacBio data SRR18512705.". Please see lines 464-468，472-473.<br><br>The reviewer links are listed below:<br>PRJNA792936<br>https://dataview.ncbi.nlm.nih.gov/object/PRJNA792936?reviewer=i6b35k4shugbjhh7cj038hoddt<br>NGDC<br>https://ngdc.cncb.ac.cn/gwh/Assembly/reviewer/ABXaDepVIMevgXXRUQKeTtPRmIRoiRwtqnjlWkSsWpxHlSfDWyDGzkmljzwHuQIz<br>Figshare<br>https://figshare.com/s/705006848f5610ac5ea9<br>FlowCytometry<br>http://flowrepository.org/id/RvFrcGmI9K1WuA4tX6QXzVuqZjkYYDUCq87SWDDLn6TKFpGYx8fKcNPheBNepSLi
**Additional Information:**	
**Question**	**Response**
Are you submitting this manuscript to a special series or article collection?	No
**Experimental design and statistics**<br><br>Full details of the experimental design and statistical methods used should be given	Yes

in the Methods section, as detailed in our Minimum Standards Reporting Checklist. Information essential to interpreting the data presented should be made available in the figure legends.<br><br>Have you included all the information requested in your manuscript?	
**Resources**<br><br>A description of all resources used, including antibodies, cell lines, animals and software tools, with enough information to allow them to be uniquely identified, should be included in the Methods section. Authors are strongly encouraged to cite Research Resource Identifiers (RRIDs) for antibodies, model organisms and tools, where possible.<br><br>Have you included the information requested as detailed in our Minimum Standards Reporting Checklist?	Yes
**Availability of data and materials**<br><br>All datasets and code on which the conclusions of the paper rely must be either included in your submission or deposited in publicly available repositories (where available and ethically appropriate), referencing such data using a unique identifier in the references and in the “Availability of Data and Materials” section of your manuscript.<br><br>Have you have met the above requirement as detailed in our Minimum Standards Reporting Checklist?	Yes

Manuscript Click here to access/download;Manuscript;manuscript_Oct 12.docx

The *Capparis spinosa* var. *herbacea* genome provides the first genomic instrument for diversity and evolution study of Capparaceae family

Lei Wang[a,b,1], Liqiang Fan[c,d,1], Zhenyong Zhao[a,b], Zhibin Zhang[c,d], Li Jiang[a,b], Mao Chai[c,d,*] and Changyan Tian[a,b,*]

[1] These authors contributed equally to this work.

[*] Corresponding authors.

Email addresses: chaimol@163.com (M. Chai); tianchy@ms.xjb.ac.cn (C. Tian)

[a] State Key Laboratory of Desert and Oasis Ecology, Xinjiang Institute of Ecology and Geography, Chinese Academy of Sciences, Urumqi 830011, China

[b] University of Chinese Academy of Sciences, Beijing 100049, China

[c] Institute of Cotton Research of the Chinese Academy of Agricultural Sciences, Anyang, Henan 455000, China

[d] Zhengzhou Research Base, State Key Laboratory of Cotton Biology, Zhengzhou University, Zhengzhou 450000, China

Lei Wang [0000-0002-8253-7295];

Mao Chai [0000-0001-6502-2137];

Changyan Tian [0000-0002-8994-386X]

Abstract

Background

The caper bush *Capparis spinosa* L., one of the most economically important species of Capparaceae, is a xerophytic shrub that is well adapted to drought and harsh environments. However, genetic studies on this species are limited because of the lack of its reference genome.

Findings

We sequenced and assembled the *Capparis spinosa* var. *herbacea* (Willd.) genome using data obtained from the combination of PacBio circular consensus sequencing and high-throughput chromosome conformation capture. The final genome assembly was approximately 274.53 Mb (contig N50 length of 9.36 Mb, scaffold N50 of 15.15 Mb), 99.23% of which was assigned to 21 chromosomes. In the whole-genome sequence, tandem repeats accounted for 19.28%, and transposable element sequences accounted for 43.98%. The proportion of tandem repeats in the *C. spinosa* var. *herbacea* genome was much higher than the average of 8.55% in plant genomes. A total of 21,577 protein-coding genes were predicted, with 98.82% being functionally annotated. The result of species divergence times showed that *C. spinosa* var. *herbacea* and *Tarenaya hassleriana* separated from a common ancestor 43.31 MYA.

Conclusions

This study reported a high-quality reference genome assembly and genome features for the Capparaceae family. The assembled *C. spinosa* var. *herbacea* genome might provide a system for studying the diversity, speciation, and evolution of this family, and serve as an important resource for understanding the mechanism of drought and high-temperature resistance.

Issue Section: Data Note

Keywords: *Capparis spinosa* var. *herbacea*; genome assembly; population evolution

Background

The caper bush *Capparis spinosa*（NCBI:txid2717819), one of the most economically important species of Capparaceae, is a perennial winter deciduous shrub with a wide range, typically growing in the Mediterranean countries and distributed in Iran, Iraq, Saudi Arabia, and China [1-3]. In China, it is mainly found in Xinjiang, Gansu, and Tibet regions [4]. The *C. spinosa* family Capparaceae from the Mediterranean to Central Asia has been taxonomically revised recently [5]. *C. spinosa* is considered a single species, represented by four subspecies—*C. spinosa* subsp. *spinosa*, *C. spinosa* subsp. *rupestris*, *C. spinosa* subsp. *cordifolia*, and *C. spinosa* subsp. *himalayensis*. *C. spinosa* subsp. *spinosa* is widely distributed from the east Mediterranean to China and Nepal and possesses a high degree of heterogeneity in different genetic traits. Within *C. spinosa* subsp. *spinosa* subspecies, some varieties are identified namely *C. spinosa* var. *herbacea* and *C. spinosa* var. *atlantica* [6]. *C. spinosa* var. *herbacea* is mainly distributed in Turkey, Kazakhstan, Uzbekistan, Afghanistan and western China [7]. According to the morphological characteristics of China reported by previous researchers [5], *C. spinosa* var. *herbacea* is mainly distributed in Xinjiang, Tibet and Gansu in China.

As a drought-tolerant crop, *C. spinosa* has an extensive root system and a remarkably high root-to-shoot ratio and thus has a strong ability to find and absorb water from the environment (especially deep in the soil), resulting in significant adaptation to harsh environments [8, 9] (Fig. 1). Besides the roots, other parts of *C. spinosa*, including leaves, buds, fruits, bark, and seeds, contain a variety of bioactive compounds, such as flavonoids, phenolics, alkaloids, glucosinolates, and vitamins that have long been used in the treatment of headaches, toothaches, and kidney disease, and play a role in preventing disease and reducing the risk of carcinogenesis [10-16]. For example, methanolic extracts prepared from the fruits and flower buds of *C. spinosa* have some anti-inflammatory and anti-thrombotic effects [17]. *C. spinosa* has a huge agricultural potential because of its medicinal properties and its ability to grow under

drought conditions. Thus far, only a few chloroplast genomes [7, 18-20], mitochondrial genomes [21], and SSR sequences [22] of *Capparis* have been reported, and the lack of genomic information hinders the genetic improvement and effective use of caper plants.

Here, we report a high-quality whole-genome sequence of *C. spinosa* var. *herbacea* using PacBio HiFi sequencing and high-throughput chromosome conformation capture (Hi-C) technology. Detailed information on the *C. spinosa* var. *herbacea* genome can help elucidate the biogeography and evolution of *Capparis* plants, contribute to the understanding of the molecular basis of its resistance to stress and validate its medicinal uses.

Analysis

Genome size estimation

We used a single plant of *Capparis spinosa* var. *herbacea* that was collected from the Xinjiang Institute of Ecology and Geography Chinese Academy of Sciences for whole-genome sequencing. A total of 33.08 G genomic short-read data were obtained for the genome survey (Table 1). We generated the 17-mer distribution of sequencing reads from short libraries using the k-mer method. The estimated genome size was about 245.97 Mb, and the proportion of repeat sequences and the genome heterozygosity rate were determined to be approximately 49.5% and 0.878%, respectively (Fig. S1A). The flow cytometry [23] analysis result was 279.07 Mb (Fig. S1B).

Table 1. Sequencing data used for *Capparis spinosa* var. *herbacea* genome assembly and annotation.

Sequencing type	Application	Sequencing platform	Bases (Gb)	Reads
Genome short reads	Genome survey and assessment	Illumina NovaSeq 6000	33.08	221,078,842
Genome long reads	Contig assembly	PacBio Sequel II	25.46	1,531,982
Hi-C reads	Chromosome construction	Illumina NovaSeq 6000	30.64	204,744,634

Transcriptome long reads	Genome annotation	PacBio Sequel II	1.52	413,148
Transcriptome short reads	Genome annotation	Illumina NovaSeq 6000	11.31	75,789,484

Genome sequencing and assembly

In this study, PacBio circular consensus sequencing (CCS) long reads and Hi-C reads were used for *C. spinosa* var. *herbacea* genome sequencing and assembly. A total of 25.46 Gb PacBio clean long reads with an average read length of 16,618 bp were generated for genome assembly, and 30.64 Gb Hi-C data were generated for auxiliary genome assembly (Table 1, Table S1). The primary contigs were assembled with PacBio CCS reads, and a 274.53 Mb genome assembly version was generated with contig N50 of 11.04 Mb (Table S1). Hi-C reads were used to generate chromosome-level assembly of the genome (Fig. 2, Fig. S2). The final genome assembly of *C. spinosa* var. *herbacea* was 274.53 Mb, consisting of 59 contigs and 29 scaffolds. The contig N50 was 9.36 Mb and the longest contig was 22.51 Mb, while the scaffold N50 was 15.15 Mb and the longest scaffold was 26.66 Mb (Table 2).

For genome quality assessment, BUSCO analysis of the final scaffold assembly showed that 96.80% complete BUSCO genes (92.80% complete and single-copy BUSCO genes, and 4.00% complete and duplicated BUSCO genes) were identified (Table S2). Merqury revealed a consensus quality value (QV) of 28.27 and assembly accuracy of 99.85%. Core Eukaryotic Genes Mapping Approach (CEGMA) was used to evaluate the completeness of the final genome assembly, and 98.03% of the CEGMA genes were present in the genome. A total of 98.52% short sequences were successfully aligned to the genome. The genome LAI value was 17.19 of the genome assembly. A LAI value greater than 10 and less than 20 indicates that the assembly quality has reached the reference genome level [24]. Thus, these results demonstrate the high quality and completeness of the *C. spinosa* var. *herbacea* genome assembly.

Table 2. Assembly statistics of the *Capparis spinosa* var. *herbacea* genome.

Category	Numbers	N50 (Mb)	Longest (Mb)	Size (Mb)	Percentage of assembly
Contigs	59	9.36	22.51	274.53	100
Scaffold	29	15.15	26.66	274.53	100
Anchored	28	15.15	26.66	274.49	99.98
Anchored and oriented	21	15.15	26.66	272.43	99.23
Gene annotated	21,577	NA	NA	64.26	23.42
Repeat sequence	NA	NA	NA	173.60	63.23

Identification of genomic repetitive sequences

Moreover, 120,748,115 bp (nearly half of the assembled genome length (43.98%)) of transposable element (TE) repetitive sequences in the genome assembly of *C. spinosa* var. *herbacea* were identified by both homology-based and *de novo* methods (Table S3). Retroelement elements constituted the predominant repeat type, accounting for 31.24% of the genome length. The long terminal repeat (LTR) superfamily elements Copia and DNA TEs constituted 29,749,806 and 34,990,312 bp, corresponding to 10.84% and 12.75% of the genome length, respectively. LTR superfamily elements Gypsy and CACTA constituted 11,447,091 and 7,034,814 bp, accounting for 4.17% and 2.56% of the genome length, respectively. The density of Copia elements decreased with the increasing density of genes, whereas the DNA TEs were distributed more evenly across the genome and showed no obvious patterns or relationships with the distribution of genes (Fig. 2).

The total length of the identified tandem repeats (TRs) was 52,920,691 bp, accounting for 19.28% of the total length of the genome. The total length of microsatellites (1–9 bp units) was 43,481,890 bp (15.84%), the total length of minisatellites (10–99 bp units) was 7,039,326 bp (2.56%), and the total length of satellites (≥100 bp units) was 2,399,475 bp (0.87%).

On analyzing the genome distribution features, we found a correlation between the distribution of

TR sequences and GC content of the chromosomes of the *C. spinosa* var. *herbacea* genome (Fig. 2C, E; Fig. S3A). Spearman rank correlation was used to determine the correlation, and the correlation coefficient was −0.52 and the *P*-value was 2.2e-16 (Fig. S3B), showing a negative correlation between the distribution of TR sequences in the *C. spinosa* var. *herbacea* genome and the GC content of the sequences.

Genome coding gene prediction and annotation

A total of 11.31 Gb transcriptome short reads and 1.52 Gb transcriptome long reads were used for gene prediction (Table 1). Combining the results by the three methods, 21,577 protein-coding genes were predicted (Table 2, Table S4). Over 98.82% of the protein-coding genes were annotated for gene function using the following databases: GO (84.53%), KEGG (76.57%), KOG (59.33%), TrEMBL (98.63%), Pfam (87.75%), Swiss-Prot (84.76%), eggNOG (87.90%), and Nr (98.69%) (Table S5), indicating that gene predictions were accurate.

Dynamic changes of duplicated genes

Duplicated genes were classified into five categories, whole-genome duplication (WGD), tandem duplication (TD), proximal duplication (PD), transposed duplication (TRD), and dispersed duplication (DSD) (Fig. 3A, Table S6). Of the 21,577 genes, 18,432 were identified as duplicated genes, including 9,603 derived from WGD (52.1%), 872 from TD (4.7%), 387 from PD (2.1%), 4,534 from TRD (24.6%), and 3,036 from DSD (16.5%). Ka (number of nonsynonymous substitutions per nonsynonymous site), Ks (number of synonymous substitutions per synonymous site), 4DTv (fourfold degenerate synonymous site), and the Ka/Ks ratio were calculated for the different duplication types. Among the five duplication types, the proportion of gene pairs with Ka/Ks >1 in *Arabidopsis thaliana* was PD (5.1%), TD (3.3%), DSD (0.6%), TRD (0.3%), and WGD (0.0%). However, the corresponding ratios in *C. spinosa* var. *herbacea* were PD (13.7%), TD (4.9%), DSD (1.3%), TRD (0.9%), and WGD (1%). PD and TD genes

had qualitatively higher Ka/Ks ratios than genes derived from the other duplication types (Fig. 3B). PD with Ka/Ks >1 in *C. spinosa* (13.7%) was significantly higher than that of *A. thaliana* (5.1%). The density distribution of Ks and 4DTv showed that all five duplication types of *C. spinosa* var. *herbacea* experienced two duplications (Fig. 3C, D). However, the five duplication types had different times when duplication occurred. PD experienced a duplication at the recent 3.89 MYA (Ks peak at 0.069, 4DTv peak at 0.013). This also explains the high proportion of positive selection in PD.

GO and KEGG enrichment analysis was performed on the Ka/Ks >1 genes in the five duplication types. In GO enrichment analysis, all five duplication types exhibited divergent functions. TRD was not enriched to a significant GO term. WGD and DSD were mainly enriched in the GO terms of plastid stroma, chloroplast stroma, obsolete chloroplast part, organellar small ribosomal subunit, and organellar ribosome. PD and TD shared more enriched GO terms related to pyrroline-5-carboxylate reductase activity, L-proline biosynthetic process, rRNA processing, protein disulfide oxidoreductase activity, peroxisome, cysteine-type peptidase activity, terpene synthase activity, magnesium ion binding, defense response to fungus, rRNA binding, response to wounding, and small ribosomal subunit compared with the other duplication types. KEGG enrichment analysis of PD and TD showed that these genes were mainly enriched in heat shock 70-kDa protein 1/2/6/8, molecular chaperone HtpG, (-)-germacrene D synthase, and KUP system potassium uptake protein, suggesting that the PD and TD genes in *C. spinosa* var. *herbacea* play important roles in environmental stress tolerance (Fig. S4).

Analyses of genome synteny and WGD

To analyze the evolution of the *C. spinosa* var. *herbacea* genome, dot plots of longer syntenic blocks within the *C. spinosa* var. *herbacea* genome were completed. *C. spinosa* var. *herbacea* undergoing WGD was clearly seen at Chr19 and Chr21 (Fig. S5A). Moreover, the syntenic blocks and collinear gene pairs between *C. spinosa* var. *herbacea* and *Amborella trichopoda*, *C. spinosa* var. *herbacea* and *A. thaliana*,

C. spinosa var. *herbacea* and *Theobroma cacao*, *C. spinosa* var. *herbacea* and *Vitis vinifera*, *C. spinosa* var. *herbacea* and *Solanum lycopersicum*, *T. hassleriana* and *C. spinosa* var. *herbacea,* and *Gynandropsis gynandra* and *C. spinosa* var. *herbacea* were implemented, respectively (Fig. S5). The syntenic analysis results also showed more collinear gene pairs between *C. spinosa* var. *herbacea* vs. *A. thaliana* and *C. spinosa* var. *herbacea* vs. *T. hassleriana* (Table S7), indicating that *C. spinosa* var. *herbacea* has a close evolutionary relationship with *A. thaliana* and *T. hassleriana*. At the same time, it can be seen from the stacking diagram of collinear genes on chromosomes that *C. spinosa* var. *herbacea* underwent WGD alone after divergence from *A. thaliana* (Fig. S5D).

Using the homologous gene pairs identified above, the 4DTv and Ks values were calculated for *C. spinosa* var. *herbacea*, *V. vinifera*, *S. lycopersicum*, *A. thaliana*, *T. hassleriana*, *Gynandropsis gynandra* and *T. cacao*. The results showed that *C. spinosa* var. *herbacea* separated from *T. cacao*, *G. gynandra, A. thaliana*, and *T. hassleriana* in order at 93.10 MYA (Ks peak of 1.644 and 4DTv peak of 0.336), 58.46 MYA (Ks peak of 1.032 and 4DTv peak of 0.266), 53.00 MYA (Ks peak of 0.936 and 4DTv peak of 0.254), and 43.31 MYA (Ks peak of 0.765 and 4DTv peak of 0.220). Besides, *C. spinosa* var. *herbacea* experienced an α WGD event at 18.59 MYA (Ks peak at 0.328) (Fig. 4 A, B).

We compared the LTR insertion time of *A. thaliana*, *C. spinosa* var. *herbacea*, *S. lycopersicum*, *T. cacao*, *Tarenaya hassleriana*, and *V. vinifera* (Fig. 4C). The results indicated that LTR bursts the time peak of *C. spinosa* var. *herbacea* (peak at 0.178 MYA) between *A. thaliana* (peak at 0.236 MYA) and *T. hassleriana* (peak at 0.132 MYA), which was also consistent with the phylogenetic tree (Fig. 5B).

Gene family expansion and contraction

Protein sequences of 15 species, namely *Oryza sativa*, *Brachypodium distachyon*, *Ananas comosus*, *Musa acuminata*, *Cinnamomum micranthum*, *Nelumbo nucifera*, *Tetracentron sinense*, *V. vinifera*, *S. lycopersicum*, *A. trichopoda*, *Nymphaea colorata*, *T. hassleriana*, *A. thaliana*, *T. cacao*, *Populus*

trichocarpa, together with *C. spinosa* var. *herbacea*, were downloaded for gene family expansion and contraction analysis. As a result, all protein-coding genes were clustered into 49,850 orthogroups based on sequence homology. A total of 1,846 gene families were shared by all 16 species, and 142 *C. spinosa* var. *herbacea* specific gene families were found (Fig. 5A). Moreover, the KEGG enrichment analysis revealed that species-specific genes were enriched in DNA kinase ATPase repair, MFS transporter, peroxin−3, disease resistance protein RPM1 and zinc finger SWIM domain−containing protein 3 (Fig. S6A).

Based on the 306 orthogroups of single-copy genes, the phylogenetic tree was constructed and the MCMCTree program in PAML was used to estimate divergence times. The phylogenetic tree identified the closest relationship of *C. spinosa* var. *herbacea* to *T. hassleriana*. Based on the time tree, the number of gene families that experienced expansion or contraction was estimated by computational analysis of gene family evolution (CAFE). The results showed that in almost species, except *B. distachyon* and *A. thaliana*, more gene families experienced expansion rather than contraction. In *C. spinosa* var. *herbacea*, 26 gene families experienced expansion, while 11 gene families underwent contraction (Fig. 5B). GO enrichment analysis of the expanded gene families of *C. spinosa* var. *herbacea* showed that these genes were mainly enriched in chloroplast thylakoid, chloroplast envelope, thylakoid, chloroplast thylakoid membrane, response to abscisic acid, response to the hormone, and so forth (Fig. 5C). Moreover, based on KEGG enrichment analysis, the genes related to photosynthesis, chloroplast thylakoid membrane, and response to abscisic acid of hormone-related pathways were enriched (Fig. S6B). The function for these gene families expanded in *C. spinosa* var. *herbacea*, indicating that the expansion of the hormone response pathway and the photosynthesis pathway might have helped *C. spinosa* var. *herbacea* to generate more energy to adapt to arid environments.

Discussion

It is well known that *Capparis spinosa* has many subspecies and varieties [7, 25], and the identification of samples is often controversial. According to the taxonomic characteristics of *C. spinosa* var. *herbacea*, the branchlets are usually white-tomentose in the upper part and the stipules are straight, horizontal or slightly curved, and yellowish [5]. The samples used for genome sequencing in this study matched the above taxonomic characteristics (Fig. 1D). Besides, the location where the samples were collected in this study is consistent with the geographical distribution of *C. spinosa* var. *herbacea* in China reported by Maurya et al. [7]. Based on the above, there is no dispute that the species used for genome sequencing in this study was *Capparis spinosa* var. *herbacea*.

Currently, genetic research in the Capparaceae family is limited by the lack of its own genomic resources, especially a reference genome. Here, we report a chromosome-level genome assembly of *C. spinosa* var. *herbacea*, with a contig N50 of 9.36 Mb and scaffold N50 of 15.15 Mb, providing the first reference genome for the Capparaceae family. Interestingly, the high TR percentage and GC content of the genome can affect the accuracy of the genome assembly. In this study, the percentage of TR in the *C. spinosa* var. *herbacea* genome was 19.28%, which was much higher than the average value of 8.55% in plants [26]. In addition, we found localized high GC content in the *C. spinosa* var. *herbacea* genome, for example, the GC content of Chr06: 3700000–15800000 in the *C. spinosa* var. *herbacea* genome was 53.92%, much higher than the genomic GC content of 36.61%, which may affect the assembly accuracy of this segment on Chr06. The effect of assembly quality can be seen at the corresponding Chr06 position in the Hi-C contact map (Fig. S2). Although the Illumina Hi-C sequencing favored the anchoring of the scaffolds in the chromosomes, the lack of genetic maps leaves the anchor a bit weak.

Both the chemical systems [27] and chloroplast DNA [28] evidence demonstrated a relatively recent evolutionary relationship between Capparaceae and Brassicaceae and Cleomaceae. The

phylogenetic tree of single-copy genes (Fig. 5B) indicated that C. *spinosa* var. *herbacea* (Capparaceae) was close to *Arabidopsis thaliana* (Brassicaceae) and *Tarenaya hassleriana* (Cleomaceae) in evolutionary relationship, which was consistent with these findings. WGDs are particularly prevalent in angiosperms and play important roles in the evolutionary history of angiosperms [29]. This *C. spinosa* var. *herbacea* genome assembly can improve the understanding of the timing of WGD events in the Capparaceae family. Because TR genes can affect the distribution of Ks peaks [30], and the *C. spinosa* var. *herbacea* genome had a high proportion of TRs, we calculated Ks and 4DTv separately for the five duplication types. The results show that the last duplication of WGD was before that of the other four duplication types. Compared with the other duplication types, PD had the highest ratio of Ka/Ks >1, indicating strong positive selection. The peaks of Ks (0.069) and 4DTv (0.013) also confirmed that the duplication of PD was very recent. WGD and Ks results showed that *C. spinosa* var. *herbacea* underwent three WGD events (γ-β-α). γ WGD occurred at 128.64-150.54 MYA (Ks peak 2.272-2.659), β WGD occurred at 92.65-102.56 MYA (Ks peak 1.636-1.811), and α WGD occurred at 18.59 MYA (Ks peak 0.328). The α WGD peak was consistent with the results (Ks ~0.3) reported by Makenzie et al. in the Capparaceae family [31]. The separation times of *C. spinosa* var. *herbacea* and *A. thaliana* and *T. hassleriana* were 53.00 MYA (Ks peak 0.936) and 43.31 MYA (Ks peak 0.765).

As a medicinal plant, *C. spinosa* contains various bioactive compounds that have long been used in traditional medicine [10-15], including secondary metabolites such as phenolic compounds and flavonoids, which often play a role in abiotic stress responses and are broadly associated with heat tolerance [6, 32]. The bioactive components of *C. spinosa* from different geographical origins are quite different [33]. The KEGG enrichment analysis of *C. spinosa* var. *herbacea* specific genes (Fig. S6A) and expansion genes (Fig. S6B) jointly enriched for two major classes of DNA repair protein and peroxin-3 pathways, including the KUP system potassium uptake protein, syndetin, FK506-binding

protein 4/5, DNA excision repair protein ERCC-6-like, DNA-directed RNA polymerase II subunit RPB1, basic endochitinase B, UDP-sugar pyrophosphorylase, F-type H+/Na+-transporting ATPase subunit alpha, DNA repair protein REV1 and peroxin-3. The KUP family plays critical roles in K+ acquisition and transport, growth and development, and responses to stress [34]. Dehydrin-FK506-binding protein complex could enhance drought tolerance through the ABA-mediated signaling pathway [35]. Besides, specific gene KEGG enrichment analysis also enriched the MFS transporter, disease resistance protein RPM1, and zinc finger SWIM domain−containing protein 3 pathway (Table S8).

Over a long period of evolution, *C. spinosa* var. *herbacea* has well adapted itself to drought and high temperature environments; for instance, five genes associated with heat shock protein (HSP) were involved in the top 20 KEGG enriched pathways (Fig. S6). The ability of plants to use light energy through photosynthesis declines under stressful conditions, which leads to the production of a large amount of reactive oxygen species because excess light energy has not been used for photosynthesis, and ultimately causes photoinhibition and oxidative damage to chloroplasts and other cell structures [36]. *In vivo* and *in vitro* studies showed that when plants are exposed to drought and heat stress, the expression of a series of HSP genes is induced, most of which interact with other proteins in the cell and alter their function, protecting against harmful effects [37-39], thus finding the enrichment of HSP genes in *C. spinosa* var. *herbacea* is explaining their role in determining drought and high-temperature stress tolerance in *C. spinosa* var. *herbacea*.

In this study, we also presented a chromosome-level genome assembly of *C. spinosa* var. *herbacea* using the combination of PacBio CCS and Hi-C data. The final genome assembly was grouped into 21 chromosomes with a size of 274.53 Mb. The high-quality reference *C. spinosa* var. *herbacea* genome assembled in this study is the first reported genomic resource for the Capparaceae family and can facilitate future studies on the mechanisms of drought and high-temperature resistance in this species,

providing a system for studying the diversity, speciation, and evolution of this family.

Methods

Plant materials and nucleic acid extraction

The source plant (Fig. 1) is an individual of *Capparis spinosa* var. *herbacea* (Willd.) Fici (NCBI:txid2717819) collected from the wild in Gaochang District, Turpan City, China (42°55′ N, 89°10′ E) and identified and confirmed by taxonomist Xiyong Wang of Xinjiang Institute of Ecology and Geography, Chinese Academy of Sciences. The material samples of the assembled genome were deposited in the Specimen Museum of Xinjiang Institute of Ecology and Geography, Chinese Academy of Sciences, Urumqi 830011, China (NO. XJBI 00108198).

On September 14, 2020, fresh and healthy leaves were harvested and immediately frozen in liquid nitrogen, followed by storage at −80°C in the laboratory before DNA and RNA extraction. Genomic DNA was extracted from the fresh leaf tissue (200 mg) that had been ground in liquid nitrogen using cetyltrimethylammonium bromide buffer (60 min incubation at 65°C), followed by phenol/chloroform/isoamyl purification (25:24:1), and isopropanol and ethanol precipitation. The resulting purified DNA was resuspended in Tris-EDTA buffer for subsequent sequencing [40]. Total RNA was extracted from the fresh samples of roots, stems, leaves, flowers, and fruits according the instructions of RNAprep Pure Plant Plus Kit (DP441, Tiangen, China). The RNA from the above tissues was mixed in equal amounts and used for RNA sequencing library construction.

Library construction and sequencing

PacBio library construction and sequencing were performed following the standard protocols provided by PacBio. Genomic DNA was sheared into ~15 kb fragments by Megaruptor 2. The SMRTbell library was constructed using the SMRTbell Express Template Prep Kit 2.0 (Pacific Biosciences, CA, USA). Library size and quantity were assessed using the FEMTO Pulse and the Qubit dsDNA HS reagents

Assay kit (Thermo Fisher Scientific, Waltham, MA, USA). Sequencing primer and Sequel II DNA Polymerase were annealed and bound, respectively, to the final SMRTbell library. The library was loaded at an on-plate concentration of 55 pM using diffusion loading. SMRT sequencing was performed using a single 8M SMRT Cell on the PacBio Sequel II System (PacBio Sequel II System, RRID:SCR_017990) with Sequel II Sequencing Kit.

The sequencing for genome survey was performed according to the standard protocol provided by Illumina. Using the extracted genomic DNA, small fragment library construction and sequencing were performed. Qualified genomic DNA was fragmented to the target fragment (350 bp) by physical fragmentation (ultrasonic vibration), followed by end repair, polyadenylation, adapter ligation, target fragment selection, and PCR [41]. The library was sequenced with paired-ended 150 bp (PE 150) using the Illumina NovaSeq 6000 platform (Illumina NovaSeq 6000 Sequencing System, RRID:SCR_016387).

Instructions of the VAHTS Universal V6 RNA-seq Library Prep Kit for Illumina (NR604-02; Vazyme, China) were followed to construct the transcriptomic short reads library. The constructed library was sequenced on the Illumina NovaSeq 6000 platform. The transcriptomic long reads library was obtained after using the SMRTbell Template Prep Kit to perform damage repair, end repair, and ligation of the mixed products. The reaction was performed in a PCR thermal cycler or a constant temperature metal bath. After RNA reverse transcription and PCR amplification, the library was sequenced on a PacBio Sequel II system.

Hi-C fragment libraries were constructed as reported by Fu et al. [42]. The main procedures included cross-linking DNA, restriction enzyme digestion, end repair, DNA circularization, and DNA purification. This library was sequenced on the Illumina NovoSeq 6000 platform.

Estimation of genome features

We estimated genome size using genome survey and flow cytometry before genome assembly, respectively. Genomic DNA was re-sequenced using the Illumina NovaSeq 6000 sequencing platform and a total of 33.09 G of data was obtained for genome survey. The short reads were quality filtered using Fastp (fastp, RRID:SCR_016962) v0.23.0 with default parameters [43]. K-Mer Counter (KMC, RRID:SCR_001245) v3.0.0 with the parameters kmc -k17 -t24 -m64 -ci1 -cs20000 @FILES reads tmp and kmc_tools transform reads histogram reads.histo -cx20000 was used to obtain the K-mer file from the clean data [44]. GenomeScope (GenomeScope, RRID:SCR_017014) 2.0 with the parameters genomescope.R -i reads.histo -o output -k 17 was used to estimate genome heterozygosity, repeat sequences, and size from the k-mer file [45].

For flow cytometry-based prediction, samples were placed in 500 µl nuclei extraction buffer, chopped with a sharp blade, and filtered through a 50-µm filter after 60 s. This was followed by the addition of 2000 µl of staining buffer with RNase for 15 min in dark. Nuclei suspensions were analyzed by CyFlow Space flow cytometer (Sysmex Partec, Muenster, Germany) and the corresponding FloMax software (FloMax, RRID:SCR_014437). The genome size of *C. spinosa* var. *herbacea* was calculated according to the formula "peak (ref)/genome size (ref) = peak (*C. spinosa* var. *herbacea*)/genome size (*C. spinosa* var. *herbacea*)" using *Solanum pimpinellifolium* as a reference genome with length 807.6 Mb [46].

Chromosome-level assembly with Hi-C data

BWA aligner v0.7.17 [47] was used to align the clean Hi-C reads to the assembly results, and uniquely alignable read pairs with mapping quality more than 20 were retained for further analysis. Invalid read pairs, including dangling ends and self-cyclization, re-ligated, and dumped products, were filtered by HiC-Prov2.8.1 [48]. LACHESIS (LACHESIS, RRID:SCR_017644) [49] was used for clustered,

ordered, and oriented scaffolds onto chromosomes. Parameters for running LACHESIS were as follows: CLUSTER_MAX_LINK_DENSITY=2; C-LUSTER_MIN_RE_SITES=9; ORDER_MIN_N_RES_IN_SHREDS=15; ORDER_MIN_N_RES_IN_TRUN=15. Clean Hi-C reads, accounting for 100-fold coverage of the survey genome, and the final 28 scaffolds were anchored to chromosomes, accounting for 99.98% of the total length. The Hi-C interactions were used as evidence for contig proximity and scaffold/contig sequences.

Genome assembly and evaluation

The raw PacBio sequencing reads were assembled using Hifiasm (Hifiasm, RRID:SCR_021069) v0.14 [50] with the parameters -l 2 -n 4. Purge_dups (purge dups, RRID:SCR_021173) v1.2.5 (default parameters) [51] was used to identify and remove haplotypic duplication in the genome assembly.

Five methods were used to evaluate the quality of the genome assembly, including the second-generation data return ratio, CEGMA (CEGMA, RRID:SCR_015055) evaluation, BUSCO (BUSCO, RRID:SCR_015008) evaluation, Merqury, and LAI value evaluation. BWA-MEM v0.7.17 (default parameters) [47] was used to compare the short reads obtained from the Illumina HiSeq sequencing data with the reference genome. CEGMA v2.5 [52], which contains 458 conserved core eukaryotic genes, was used to evaluate the completeness of the genome assembly. The Embryophyta database of BUSCO v5.2.1 [53] contains 1,614 conserved core genes that were used to assess the integrity of the genome assembly. Assembly QV was calculated using Merqury v1.3 [54].

Full-length LTR retrotransposons (LTR-RTs) in the genome were identified by LTR_finder (LTR_Finder, RRID:SCR_015247) v1.07 [55] and LTRharvest (LTRharvest, RRID:SCR_018970) v1.6.1 [56]. LTR_retriever (LTR_retriever, RRID:SCR_017623) v2.9.0 [57] was then used to combine LTR-RTs, remove duplicates, and calculate the LAI value and calculate the insertion time of LTR-RTs. LTR_finder parameters were -D 40000 -d 100 -L 9000 -l 50 -p 20 -C -M 0.9.

LTRharvest parameters were -minlenltr 100 -maxlenltr 40000 -mintsd 4 -maxtsd 6 -motif TGCA -motifmis 1 -similar 85 -vic 10 -seed 20 -seqids yes. LTR_retriever was set with the parameter -u 7e-9, which is used to set the molecular clock r value to $7*10^{-9}$ [58].

Identification of repeat sequences

TEs and TRs were identified separately. We combined homology-based and *de novo* approaches to identify TEs. We first customized a *de novo* repeat library of the genome using RepeatModeler2 v2.0.1 (default parameters) [59]. The *de novo* TE-sequence library and LTR-RT library described above were merged with the known Repbase (Repbase, RRID:SCR_021169) v19.06, REXdb v3.0 and Dfam (Dfam, RRID:SCR_021168) v3.2 databases. After removing redundant sequences using the seqkit (SeqKit, RRID:SCR_018926) v2.1.0 (parameter: rmdup -s) [60], a non-redundant species-specific TE library was constructed. TE sequence was identified and classified by RepeatMasker (RepeatMasker, RRID:SCR_012954) v4.1.1 (default parameters) [61]. TRs were identified by MISA (MISA, RRID:SCR_010765) v2.1 [62] with default parameters and TRF v4.09 [63] with the parameters 1 1 2 80 5 200 2000 -d -h.

Gene prediction and annotation

The three approaches of *de novo* prediction, homology search, and transcript-based assembly were used to annotate protein-coding genes [42]. Augustus (Augustus, RRID:SCR_008417) v3.1 (default parameters) [64] and SNAP (SNAP 3, RRID:SCR_009400) v2013-02-16 (default parameters) [65] were used for *de novo* prediction. Homologous species were predicted in GeMoMa (GeMoMa, RRID:SCR_017646) v1.7 (default parameters) [66] using the reference gene models of *A. thaliana*, *Cannabis sativa*, *Eutrema salsugineum*, and *T. hassleriana*. For the transcript-based prediction, total of 11.31 Gb Illumina NovaSeq 6000 RNA-sequencing data were mapped to the reference genome using HISAT2 (HISAT2, RRID:SCR_015530) v2.2.0 (parameters: --max-intronlen 20000, --min-intronlen

20) [67] and assembled by Stringtie (StringTie, RRID:SCR_016323) v2.1.3b (default parameters) [68]. GeneMarkS-T (GeneMarkS-T, RRID:SCR_017648) v5. 1 (default parameters) [69] was used for gene prediction based on the assembled transcripts. A total of 1.52Gb of PacBio transcriptome data was used for full-length transcriptome sequence analysis using PacBio Iso Seq3. The PASA (PASA, RRID:SCR_014656) v2.4.1 (default parameters) [70] was used to predict genes based on the unigenes (and full-length transcripts from the PacBio sequencing) assembled by Trinity (Trinity, RRID:SCR_013048) v2.11.0 (parameters: -max_memory 100g) [71]. Gene models from these different approaches were combined using the EVM (EVidenceModeler, RRID:SCR_014659) v1.1.1 (default parameters) [70] and updated by PASA.

The predicted gene sequences were used as queries for BLAST v2.2.31 (Altschul et al., 1990) searches against the NR (202009) [72], TrEMBL (202005) [73], Pfam v33.1 [74], Swiss-Prot (202005) [75], KOG (20110125) [76], GO (20200615) [77], and KEGG (20191220) [78] databases for gene annotation.

tRNA was identified using tRNAscan-SE (tRNAscan-SE, RRID:SCR_010835) v1.3.1 [79], rRNA was predicted based on the Rfam (Rfam, RRID:SCR_007891) v12.0 database [80] and Barrnap (Barrnap, RRID:SCR_015995) v0.9 [81], miRNA was identified by the miRbase (miRBase, RRID:SCR_003152) v22 database [82], and snoRNA and snRNA were based on the Rfam database and predicted by Infernal v1.1 [83]. A total of 0 tRNAs, 2,722 rRNAs, and 100 miRNAs were predicted.

WGD analysis

GenDup_finder-unique, the stringent mode of DupGen_finder [84], was used to identify genes derived from the different duplication types. : DupGen_finder-unique divided the duplication types into five types, namely WGD, TD, PD (separated by fewer than ten genes on the same chromosome), TRD, and DSD. The Ka, Ks, and Ka/Ks values of gene pairs were calculated with ParaAT v2.0 [85]. The proportion

of each homologous gene to the 4DTv site was calculated using a Perl script. Genes with Ka/Ks >1 in the five duplication types were used for GO and KEGG enrichment analysis by clusterProfiler (clusterProfiler, RRID:SCR_016884) v4.2.0 [86].

Gene family classification

The protein sequences of 16 species (*M. acuminata*, *T. sinense*, *C. micranthum*, *A. trichopoda*, *T. hassleriana*, *A. comosus*, *S. lycopersicum*, *T. cacao*, *A. thaliana*, *B. distachyon*, *N. nucifera*, *P. trichocarpa*, *V. vinifera*, *N. colorata*, *O. sativa*, and *C. spinosa* var. *herbacea*) were used for family classification using OrthoFinder v2.4 software (diamond comparison method, E-value 0.001) [87]. The encoding genes from a species were clustered into six groups—0 copies, 1 copy (single-copy), 2 copies, 3 copies, 4 copies, and 4+ copies. A total of 306 genes were identified as single-copy genes. The obtained gene families were annotated using the PANTHER (PANTHER, RRID:SCR_004869) v15 database [88].

Phylogenetic analysis and species divergence time estimation

Each single-copy gene family sequence was aligned using MAFFT (MAFFT, RRID:SCR_011811) v7.205 [89] (parameters: --localpair --maxiterate 1000). Gblocks (Gblocks, RRID:SCR_015945) v0.91b [90] (parameter: -b5=h) was used to filter conserved sites, and all aligned gene family sequences of each species were finally connected end-to-end to obtain supergenes. The IQ-TREE (IQ-TREE, RRID:SCR_017254) v1.6.11 [91] model selection tool ModelFinder [92] was used for model selection. The best model obtained was JTT+F+I+G4, which was used to construct the phylogenetic tree by the maximum likelihood (ML) method with the bootstrap value set to 1000. *A. trichopoda* was selected as the outgroup and the root of the tree [42]. The divergence time between species was estimated using the TimeTree (TimeTree, RRID:SCR_021162) [93]. Divergence times were as follows: *A. trichopoda vs S. lycopersicum* at 164–194 MYA, *O. sativa vs B. distachyon* at 42–60 MYA, *A. comosus vs O. sativa* at

94–115 MYA, and *N. nucifera vs V. vinifera* at 116–127 MYA. The gradient and Hessian parameters required for the divergence time were estimated using MCMCTree in PAML (PAML, RRID:SCR_014932) v4.9i [94]. The ML method, correlated molecular clock, and JC69 model were used to estimate divergence times. Two repeated calculations were performed to evaluate consistency. The Markov Chain Monte Carlo (MCMC) iteration settings were burn-in 2000, sampfreq 10, and nsample 20000. MCMCtreeR v1.1 [95] was used to graphically display the phylogenetic tree with divergence times.

Gene family expansion and contraction

The results of the phylogenetic tree (with divergence times) and gene family clustering analyses were used to estimate the gene family expansion and contraction of species relative to their ancestors using CAFE (CAFE, RRID:SCR_005983) v4.2 [96]. The criteria defining significant expansion or contraction of gene families were a family-wide *P*-value < 0.05 and a Viterbi *P*-value < 0.05.

Genome collinearity analysis

To identify similar gene pairs, gene sequences of two species were compared using Diamond (DIAMOND, RRID:SCR_016071) v0.9.29.130 (parameter: e<1e-5) [97]. JCVI (jcvi, RRID:SCR_021641) v0.9.13 [98] was used to filter the BLAST results (parameter: C-score >0.5) and obtain all the genes in collinear blocks. JCVI was also used to plot the collinearity of the linear pattern of each species. Finally, the ggplot2 (ggplot2, RRID:SCR_014601) v3.3.5 R package [99] was used to display the collinearity results in the form of bar graphs.

Genome information visualization

The sliding window file of the genome was constructed using Bedtools (BEDTools, RRID:SCR_006646) v2.29.2 [100], and the window size was set to 100 kb to calculate the gene density of each chromosome. The distribution of gene density, TE sequence, TRs, and GC content, and

collinearity on the chromosomes of the genome were visualized using Circos (Circos, RRID:SCR_011798) v0.69-8 [101].

Correlation analysis of genomic distribution characteristics

We performed pairwise correlation analysis on the GC content, gene density, TE distribution, and TR distribution of the genome. Correlation analysis was performed using the Spearman method [102] using the cor function in R. The corrplot R package was used to visualize the correlation results.

Data availability

Raw data of genome assembly (PacBio HiFi and Hi-C sequences) and genome encoding gene prediction annotations (Illumina and PacBio sequences) have been deposited in the NCBI Sequence Read Archive (SRA) database under Bioproject ID: PRJNA792936. Illumina data given accession SRR18458236 for the genome size survey. Data for genome PacBio HiFi data given accession SRR17373648, and Hi-C data has accession SRR17373647. For encoding gene prediction annotations Illumina data accession SRR18512706, and PacBio data accession SRR18512705. The genome annotations have been deposited at FigShare [103]. The whole-genome sequence data have been deposited in the Genome Warehouse at the National Genomics Data Center, Beijing Institute of Genomics, under accession number GWHBGXB00000000. The genome downloads address of other species used in this study are in Table S9. All other supporting data and materials are available in the *GigaScience* GigaDB database [104].

List of abbreviations

CAFE: computational analysis of gene family evolution; DSD: dispersed duplication; Hi-C: high-throughput chromosome conformation capture; HSP: heat shock protein; LTR-RTs: LTR retrotransposons; MYA: million years ago; PD: proximal duplication; QV: quality value; SRA: Sequence Read Archive; TD: tandem duplication; TE: transposable element; TRs: tandem repeats; TRD:

transposed duplication; WGD: whole-genome duplication

Ethical statement

Not applicable

Consent for publication

Not applicable

Competing interests

The authors declare that they have no conflicts of interest.

Funding

This work was supported by the National Key Research and Development Program of China (No. 2018YFE0207200).

Authors' contributions

Lei Wang: Methodology, Sample collection, Writing - Original draft, Writing - Review & Editing. **Liqiang Fan**: Data analysis, Methodology. Writing - Original draft. **Zhenyong Zhao**: Sample collection, Investigation, Methodology. **Li Jiang**: Investigation, Methodology. **Zhibin Zhang**: Data analysis. **Mao Chai**: Data curation, Visualization, Supervision, Writing - Review. **Changyan Tian**: Project administration, Supervision, Resources, Funding acquisition, Writing - Review & Editing.

Acknowledgements

We thank Dr. Zhaoen Yang (State Key Laboratory of Cotton Biology, Institute of Cotton Research of the Chinese Academy of Agricultural Sciences, Zhengzhou, China) for providing help in data analysis. We also thank TopEdit for linguistic assistance during the preparation of this manuscript. We would also like to thank reviewers Dr. Francesco Mercati and Dr. Eric Schranz. Francesco Mercati has been instrumental in the annotation and comparison of genomes. Eric Schranz has given great help in species classification and evolutionary analysis.

References

1. Inocencio C, Rivera D, Obón MC, Alcaraz F and Barreña J-A. A systematic revision of capparis section Capparis (Capparaceae) 1, 2. Annals of the Missouri Botanical Garden. 2006;93 1:122-49.
2. Levizou E, Drilias P and Kyparissis A. Exceptional photosynthetic performance of Capparis spinosa L. under adverse conditions of Mediterranean summer. Photosynthetica. 2004;42 2:229-35.
3. Özcan M and Akgül A. Influence of species, harvest date and size on composition of capers (Capparis spp.) flower buds. Food/Nahrung. 1998;42 02:102-5.
4. Yang T, Liu Y-Q, Wang C-H and Wang Z-T. Advances on investigation of chemical constituents, pharmacological activities and clinical applications of Capparis spinosa. Zhongguo Zhong yao za zhi= Zhongguo zhongyao zazhi= China journal of Chinese materia medica. 2008;33 21:2453-8.
5. Fici S. A taxonomic revision of the Capparis spinosa group (Capparaceae) from the Mediterranean to Central Asia. Phytotaxa. 2014;174 1:1–24-1–.
6. Chedraoui S, Abi-Rizk A, El-Beyrouthy M, Chalak L, Ouaini N and Rajjou L. Capparis spinosa L. in A Systematic Review: A Xerophilous Species of Multi Values and Promising Potentialities for Agrosystems under the Threat of Global Warming. Frontiers in Plant Science. 2017;8 doi:10.3389/fpls.2017.01845.
7. Maurya S, Darshetkar AM, Datar MN, Tamhankar S, Li P and Choudhary RK. Plastome data provide insights into intra and interspecific diversity and ndh gene loss in Capparis (Capparaceae). Phytotaxa. 2020;432 2:206-20.
8. Gan L, Zhang C, Yin Y, Lin Z, Huang Y, Xiang J, et al. Anatomical adaptations of the xerophilous medicinal plant, Capparis spinosa, to drought conditions. Horticulture, Environment, and Biotechnology. 2013;54 2:156-61.
9. Zuo W, Ma M, Ma Z, Gao R, Guo Y, Jiang W, et al. Study of photosynthetic physiological characteristics of desert plant Capparis spinosa L. Journal of Shihezi University (Natural Science). 2012;30 3:7.
10. Anwar F, Muhammad G, Hussain MA, Zengin G, Alkharfy KM, Ashraf M, et al. Capparis spinosa L.: A plant with high potential for development of functional foods and nutraceuticals/pharmaceuticals. International Journal of Pharmacology. 2016;12 3:201-19.
11. Arrar L, Benzidane N, Krache I, Charef N, Khennouf S and Baghiani A. Comparison between polyphenol contents and antioxidant activities of different parts of Capparis spinosa L. Pharmacognosy Communications. 2013;3 2:70.

12. Germano MP, De Pasquale R, D'angelo V, Catania S, Silvari V and Costa C. Evaluation of extracts and isolated fraction from Capparis spinosa L. buds as an antioxidant source. Journal of agricultural and food chemistry. 2002;50 5:1168-71.
13. Matthäus B and Özcan M. Glucosinolates and fatty acid, sterol, and tocopherol composition of seed oils from Capparis spinosa Var. spinosa and Capparis ovata Desf. Var. canescens (Coss.) Heywood. Journal of Agricultural and Food chemistry. 2005;53 18:7136-41.
14. Tlili N, Feriani A, Saadoui E, Nasri N and Khaldi A. Capparis spinosa leaves extract: Source of bioantioxidants with nephroprotective and hepatoprotective effects. Biomedicine & Pharmacotherapy. 2017;87:171-9.
15. Tlili N, Nasri N, Khaldi A, Triki S and MUNNÉ-BOSCH S. Phenolic compounds, tocopherols, carotenoids and vitamin C of commercial caper. Journal of Food Biochemistry. 2011;35 2:472-83.
16. Zhang H and Ma ZF. Phytochemical and Pharmacological Properties of Capparis spinosa as a Medicinal Plant. Nutrients. 2018;10 2:116.
17. Bektas N, Arslan R, Goger F, Kirimer N and Ozturk Y. Investigation for anti-inflammatory and anti-thrombotic activities of methanol extract of Capparis ovata buds and fruits. Journal of ethnopharmacology. 2012;142 1:48-52.
18. Siragusa M and Carimi F. Development of specific primers for cpSSR analysis in caper, olive and grapevine using consensus chloroplast primer pairs. Scientia horticulturae. 2009;120 1:14-21.
19. Wang Q, Zhang M-L and Yin L-K. Phylogeographic structure of a tethyan relict Capparis spinosa (Capparaceae) traces Pleistocene geologic and climatic changes in the western Himalayas, Tianshan mountains, and adjacent desert regions. BioMed research international. 2016;2016:13.
20. Alzahrani D, Albokhari E, Yaradua S and Abba A. The complete plastome sequence for the medicinal species Capparis spinosa L. (Capparaceae). Gene Reports. 2021;23:101059. https://doi.org/10.1016/j.genrep.2021.101059.
21. Grewe F, Edger PP, Keren I, Sultan L, Pires JC, Ostersetzer-Biran O, et al. Comparative analysis of 11 Brassicales mitochondrial genomes and the mitochondrial transcriptome of Brassica oleracea. Mitochondrion. 2014;19:135-43.
22. Mercati F, Fontana I, Gristina AS, Martorana A, El Nagar M, De Michele R, et al. Transcriptome analysis and codominant markers development in caper, a drought tolerant orphan crop with medicinal value. Scientific reports. 2019;9 1:1-16.
23. DOLEŽEL J and BARTOŠ J. Plant DNA Flow Cytometry and Estimation of Nuclear Genome

Size. Annals of Botany. 2005;95 1:99-110. doi:10.1093/aob/mci005.

24. Ou S, Chen J and Jiang N. Assessing genome assembly quality using the LTR Assembly Index (LAI). Nucleic Acids Research. 2018;46 21:e126-e. doi:10.1093/nar/gky730.

25. Rhimi A, Mnasri S, Ben Ayed R, Bel Hajj Ali I, Hjaoujia S and Boussaid M. Genetic relationships among subspecies of Capparis spinosa L. from Tunisia by using ISSR markers. Molecular Biology Reports. 2019;46 2:2209-19. doi:10.1007/s11033-019-04676-z.

26. Tørresen OK, Star B, Mier P, Andrade-Navarro MA, Bateman A, Jarnot P, et al. Tandem repeats lead to sequence assembly errors and impose multi-level challenges for genome and protein databases. Nucleic Acids Research. 2019;47 21:10994-1006. doi:10.1093/nar/gkz841.

27. Leite PM and Castilho RO. Chemosystematics of Brassicales. Biochemical Systematics and Ecology. 2017;71:205-11. https://doi.org/10.1016/j.bse.2017.02.011.

28. Alzahrani DA, Albokhari EJ, Yaradua SS and Abba A. Comparative Analysis of Chloroplast Genomes of Four Medicinal Capparaceae Species: Genome Structures, Phylogenetic Relationships and Adaptive Evolution. Plants. 2021;10 6:1229.

29. Christenhusz MJM and Byng JW. The number of known plants species in the world and its annual increase. Phytotaxa. 2016;261 3:201-17.

30. Taikui Zhang ZY. Progress in plant paleogenomics. Hereditas(Beijing). 2018;40 1:44-56. doi:10.16288/j.yczz.17-191.

31. Mabry ME, Brose JM, Blischak PD, Sutherland B, Dismukes WT, Bottoms CA, et al. Phylogeny and multiple independent whole-genome duplication events in the Brassicales. American Journal of Botany. 2020;107 8:1148-64.:https://doi.org/10.1002/ajb2.1514.

32. Wahid A. Physiological implications of metabolite biosynthesis for net assimilation and heat-stress tolerance of sugarcane (Saccharum officinarum) sprouts. Journal of Plant Research. 2007;120 2:219-28. doi:10.1007/s10265-006-0040-5.

33. Stefanucci A, Zengin G, Locatelli M, Macedonio G, Wang C-K, Novellino E, et al. Impact of different geographical locations on varying profile of bioactives and associated functionalities of caper (*Capparis spinosa* L.). Food and Chemical Toxicology. 2018;118:181-9. doi:https://doi.org/10.1016/j.fct.2018.05.003.

34. Yang T, Lu X, Wang Y, Xie Y, Ma J, Cheng X, et al. HAK/KUP/KT family potassium transporter genes are involved in potassium deficiency and stress responses in tea plants (*Camellia sinensis* L.): expression and functional analysis. BMC Genomics. 2020;21 1:556. doi:10.1186/s12864-020-06948-6.

35. Tiwari P, Indoliya Y, Singh PK, Singh PC, Chauhan PS, Pande V, et al. Role of dehydrin-FK506-binding protein complex in enhancing drought tolerance through the ABA-mediated signaling

pathway. Environmental and Experimental Botany. 2019;158:136-49. doi:https://doi.org/10.1016/j.envexpbot.2018.10.031.

36. Singh AK and Singhal GS. Effect of Irradiance on the Thermal Stability of Thylakoid Membrane Isolated from Acclimated Wheat Leaves. Photosynthetica. 2001;39 1:23-7.

37. Ohama N, Sato H, Shinozaki K and Yamaguchi-Shinozaki K. Transcriptional regulatory network of plant heat stress response. Trends in plant science. 2017;22 1:53-65.

38. Ren S, Ma K, Lu Z, Chen G and Jin B. Transcriptomic and Metabolomic Analysis of the Heat-Stress Response of Populus tomentosa Carr. Forests. 2019;10 5:383.

39. Tereza T, Despina S, Anna K, Tereza V and Jozef Š. Multifaceted roles of HEAT SHOCK PROTEIN 90 molecular chaperones in plant development. Journal of Experimental Botany. 2020;71 14:20.

40. Fu J, Wan L, Song L, He L, Jiang N, Long H, et al. Chromosome-Level Genome Assembly of the Hemiparasitic Taxillus chinensis (DC.) Danser. Genome Biology and Evolution. 2022;14 5 doi:10.1093/gbe/evac060.

41. Jiang S, An H, Xu F and Zhang X. Chromosome-level genome assembly and annotation of the loquat (Eriobotrya japonica) genome. GigaScience. 2020;9 3 doi:10.1093/gigascience/giaa015.

42. Fu A, Wang Q, Mu J, Ma L, Wen C, Zhao X, et al. Combined genomic, transcriptomic, and metabolomic analyses provide insights into chayote (*Sechium edule*) evolution and fruit development. Horticulture Research. 2021;8 1:35. doi:10.1038/s41438-021-00487-1.

43. Chen S, Zhou Y, Chen Y and Gu J. fastp: an ultra-fast all-in-one FASTQ preprocessor. Bioinformatics. 2018;34 17:i884-i90.

44. Kokot M, Długosz M and Deorowicz S. KMC 3: counting and manipulating k-mer statistics. Bioinformatics. 2017;33 17:2759-61. doi:10.1093/bioinformatics/btx304.

45. Ranallo-Benavidez TR, Jaron KS and Schatz MC. GenomeScope 2.0 and Smudgeplot for reference-free profiling of polyploid genomes. Nature Communications. 2020;11 1:1432. doi:10.1038/s41467-020-14998-3.

46. Wang X, Gao L, Jiao C, Stravoravdis S, Hosmani PS, Saha S, et al. Genome of Solanum pimpinellifolium provides insights into structural variants during tomato breeding. Nature Communications. 2020;11 1:5817. doi:10.1038/s41467-020-19682-0.

47. Li H. Aligning sequence reads, clone sequences and assembly contigs with BWA-MEM. arXiv preprint arXiv:13033997. 2013.

48. Servant N, Varoquaux N, Lajoie BR, Viara E, Chen C-J, Vert J-P, et al. HiC-Pro: an optimized and flexible pipeline for Hi-C data processing. Genome Biology. 2015;16 1:259. doi:10.1186/s13059-015-0831-x.

49. Burton JN, Adey A, Patwardhan RP, Qiu R, Kitzman JO and Shendure J. Chromosome-scale scaffolding of de novo genome assemblies based on chromatin interactions. Nature Biotechnology. 2013;31 12:1119-25. doi:10.1038/nbt.2727.

50. Cheng H, Concepcion GT, Feng X, Zhang H and Li H. Haplotype-resolved de novo assembly using phased assembly graphs with hifiasm. Nature Methods. 2021;18 2:170-5. doi:10.1038/s41592-020-01056-5.

51. Guan D, McCarthy SA, Wood J, Howe K, Wang Y and Durbin R. Identifying and removing haplotypic duplication in primary genome assemblies. Bioinformatics. 2020;36 9:2896-8. doi:10.1093/bioinformatics/btaa025.

52. Parra G, Bradnam K and Korf I. CEGMA: a pipeline to accurately annotate core genes in eukaryotic genomes. Bioinformatics. 2007;23 9:1061-7.

53. Manni M, Berkeley MR, Seppey M, Simão FA and Zdobnov EM. BUSCO Update: Novel and Streamlined Workflows along with Broader and Deeper Phylogenetic Coverage for Scoring of Eukaryotic, Prokaryotic, and Viral Genomes. Molecular Biology and Evolution. 2021;38 10:4647-54. doi:10.1093/molbev/msab199.

54. Rhie A, Walenz BP, Koren S and Phillippy AM. Merqury: reference-free quality, completeness, and phasing assessment for genome assemblies. Genome Biology. 2020;21 1:245. doi:10.1186/s13059-020-02134-9.

55. Xu Z and Wang H. LTR_FINDER: an efficient tool for the prediction of full-length LTR retrotransposons. Nucleic Acids Research. 2007;35 suppl_2:W265-W8. doi:10.1093/nar/gkm286.

56. Ellinghaus D, Kurtz S and Willhoeft U. LTRharvest, an efficient and flexible software for de novo detection of LTR retrotransposons. BMC Bioinformatics. 2008;9 1:18. doi:10.1186/1471-2105-9-18.

57. Ou S and Jiang N. LTR_retriever: A Highly Accurate and Sensitive Program for Identification of Long Terminal Repeat Retrotransposons. Plant Physiology. 2018;176 2:1410-22. doi:10.1104/pp.17.01310.

58. Ossowski S, Schneeberger K, Lucas-Lledó JI, Warthmann N, Clark RM, Shaw RG, et al. The rate and molecular spectrum of spontaneous mutations in Arabidopsis thaliana. science. 2010;327 5961:92-4.

59. Flynn JM, Hubley R, Goubert C, Rosen J, Clark AG, Feschotte C, et al. RepeatModeler2 for automated genomic discovery of transposable element families. Proceedings of the National Academy of Sciences. 2020;117 17:9451. doi:10.1073/pnas.1921046117.

60. Shen W, Le S, Li Y and Hu F. SeqKit: A Cross-Platform and Ultrafast Toolkit for FASTA/Q File

Manipulation. PLOS ONE. 2016;11 10:e0163962. doi:10.1371/journal.pone.0163962.
61. Tarailo-Graovac M and Chen N. Using RepeatMasker to identify repetitive elements in genomic sequences. Current protocols in bioinformatics. 2009;25 1:4.10.1-4..4.
62. Beier S, Thiel T, Münch T, Scholz U and Mascher M. MISA-web: a web server for microsatellite prediction. Bioinformatics. 2017;33 16:2583-5. doi:10.1093/bioinformatics/btx198.
63. Benson G. Tandem repeats finder: a program to analyze DNA sequences. Nucleic acids research. 1999;27 2:573-80.
64. Keller O, Kollmar M, Stanke M and Waack S. A novel hybrid gene prediction method employing protein multiple sequence alignments. Bioinformatics. 2011;27 6:757-63. doi:10.1093/bioinformatics/btr010.
65. Korf I. Gene finding in novel genomes. BMC Bioinformatics. 2004;5 1:59. doi:10.1186/1471-2105-5-59.
66. Keilwagen J, Hartung F, Paulini M, Twardziok SO and Grau J. Combining RNA-seq data and homology-based gene prediction for plants, animals and fungi. BMC Bioinformatics. 2018;19 1:189. doi:10.1186/s12859-018-2203-5.
67. Pertea M, Kim D, Pertea GM, Leek JT and Salzberg SL. Transcript-level expression analysis of RNA-seq experiments with HISAT, StringTie and Ballgown. Nature Protocols. 2016;11 9:1650-67. doi:10.1038/nprot.2016.095.
68. Pertea M, Pertea GM, Antonescu CM, Chang T-C, Mendell JT and Salzberg SL. StringTie enables improved reconstruction of a transcriptome from RNA-seq reads. Nature biotechnology. 2015;33 3:290-5.
69. Tang S, Lomsadze A and Borodovsky M. Identification of protein coding regions in RNA transcripts. Nucleic Acids Research. 2015;43 12:e78-e. doi:10.1093/nar/gkv227.
70. Haas BJ, Salzberg SL, Zhu W, Pertea M, Allen JE, Orvis J, et al. Automated eukaryotic gene structure annotation using EVidenceModeler and the Program to Assemble Spliced Alignments. Genome Biology. 2008;9 1:R7. doi:10.1186/gb-2008-9-1-r7.
71. Grabherr MG, Haas BJ, Yassour M, Levin JZ, Thompson DA, Amit I, et al. Full-length transcriptome assembly from RNA-Seq data without a reference genome. Nature Biotechnology. 2011;29 7:644-52. doi:10.1038/nbt.1883.
72. Marchler-Bauer A, Lu S, Anderson JB, Chitsaz F, Derbyshire MK, DeWeese-Scott C, et al. CDD: a Conserved Domain Database for the functional annotation of proteins. Nucleic acids research. 2010;39 suppl_1:D225-D9.
73. Boeckmann B, Bairoch A, Apweiler R, Blatter M-C, Estreicher A, Gasteiger E, et al. The SWISS-PROT protein knowledgebase and its supplement TrEMBL in 2003. Nucleic acids research.

2003;31 1:365-70.
74. Mistry J, Chuguransky S, Williams L, Qureshi M, Salazar GA, Sonnhammer EL, et al. Pfam: The protein families database in 2021. Nucleic Acids Research. 2021;49 D1:D412-D9.
75. Boutet E, Lieberherr D, Tognolli M, Schneider M, Bansal P, Bridge AJ, et al. UniProtKB/Swiss-Prot, the Manually Annotated Section of the UniProt KnowledgeBase: How to Use the Entry View. In: Edwards D, editor. Plant Bioinformatics: Methods and Protocols. New York, NY: Springer New York; 2016. p. 23-54.
76. Koonin EV, Fedorova ND, Jackson JD, Jacobs AR, Krylov DM, Makarova KS, et al. A comprehensive evolutionary classification of proteins encoded in complete eukaryotic genomes. Genome biology. 2004;5 2:R7.
77. Dimmer EC, Huntley RP, Alam-Faruque Y, Sawford T, O'Donovan C, Martin MJ, et al. The UniProt-GO annotation database in 2011. Nucleic acids research. 2012;40 D1:D565-D70.
78. Kanehisa M and Goto S. KEGG: kyoto encyclopedia of genes and genomes. Nucleic acids research. 2000;28 1:27-30.
79. Chan PP and Lowe TM. tRNAscan-SE: searching for tRNA genes in genomic sequences. Gene prediction. Springer; 2019. p. 1-14.
80. Nawrocki EP, Burge SW, Bateman A, Daub J, Eberhardt RY, Eddy SR, et al. Rfam 12.0: updates to the RNA families database. Nucleic acids research. 2015;43 D1:D130-D7.
81. Loman T. A Novel Method for Predicting Ribosomal RNA Genes in Prokaryotic Genomes. 2017.
82. Kozomara A, Birgaoanu M and Griffiths-Jones S. miRBase: from microRNA sequences to function. Nucleic acids research. 2019;47 D1:D155-D62.
83. Nawrocki EP and Eddy SR. Infernal 1.1: 100-fold faster RNA homology searches. Bioinformatics. 2013;29 22:2933-5.
84. Qiao X, Li Q, Yin H, Qi K, Li L, Wang R, et al. Gene duplication and evolution in recurring polyploidization–diploidization cycles in plants. Genome Biology. 2019;20 1:38. doi:10.1186/s13059-019-1650-2.
85. Zhang Z, Xiao J, Wu J, Zhang H, Liu G, Wang X, et al. ParaAT: a parallel tool for constructing multiple protein-coding DNA alignments. Biochemical and biophysical research communications. 2012;419 4:779-81.
86. Wu T, Hu E, Xu S, Chen M, Guo P, Dai Z, et al. clusterProfiler 4.0: A universal enrichment tool for interpreting omics data. The Innovation. 2021;2 3:100141.
87. Emms DM and Kelly S. OrthoFinder: phylogenetic orthology inference for comparative genomics. Genome Biology. 2019;20 1:238. doi:10.1186/s13059-019-1832-y.
88. Mi H, Muruganujan A, Ebert D, Huang X and Thomas PD. PANTHER version 14: more genomes,

a new PANTHER GO-slim and improvements in enrichment analysis tools. Nucleic Acids Research. 2018;47 D1:D419-D26. doi:10.1093/nar/gky1038.

89. Katoh K and Standley DM. MAFFT multiple sequence alignment software version 7: improvements in performance and usability. Molecular biology and evolution. 2013;30 4:772-80.

90. Talavera G and Castresana J. Improvement of phylogenies after removing divergent and ambiguously aligned blocks from protein sequence alignments. Systematic biology. 2007;56 4:564-77.

91. Nguyen L-T, Schmidt HA, Von Haeseler A and Minh BQ. IQ-TREE: a fast and effective stochastic algorithm for estimating maximum-likelihood phylogenies. Molecular biology and evolution. 2015;32 1:268-74.

92. Kalyaanamoorthy S, Minh BQ, Wong TK, Von Haeseler A and Jermiin LS. ModelFinder: fast model selection for accurate phylogenetic estimates. Nature methods. 2017;14 6:587-9.

93. Kumar S, Stecher G, Suleski M and Hedges SB. TimeTree: A Resource for Timelines, Timetrees, and Divergence Times. Molecular biology and evolution. 2017;34 7:1812-9. doi:10.1093/molbev/msx116.

94. Yang Z. PAML: a program package for phylogenetic analysis by maximum likelihood. Computer applications in the biosciences : CABIOS. 1997;13 5:555-6.

95. Puttick MN. MCMCtreeR: functions to prepare MCMCtree analyses and visualize posterior ages on trees. Bioinformatics. 2019;35 24:5321-2.

96. Han MV, Thomas GWC, Lugo-Martinez J and Hahn MW. Estimating Gene Gain and Loss Rates in the Presence of Error in Genome Assembly and Annotation Using CAFE 3. Molecular Biology and Evolution. 2013;30 8:1987-97. doi:10.1093/molbev/mst100.

97. Buchfink B, Xie C and Huson DH. Fast and sensitive protein alignment using DIAMOND. Nature methods. 2015;12 1:59-60.

98. Tang H, Krishnakumar V, Li J and Zhang X. jcvi: JCVI utility libraries. Zenodo(doi: 105281/zenodo 31631). 2015.

99. Villanueva RAM and Chen ZJ. ggplot2: elegant graphics for data analysis. Taylor & Francis, 2019.

100. Quinlan AR. BEDTools: the Swiss-army tool for genome feature analysis. Current protocols in bioinformatics. 2014;47 1:11.2. 1-.2. 34.

101. Krzywinski M, Schein J, Birol I, Connors J, Gascoyne R, Horsman D, et al. Circos: an information aesthetic for comparative genomics. Genome research. 2009;19 9:1639-45.

102. Spearman Rank Correlation Coefficient. The Concise Encyclopedia of Statistics. New York, NY: Springer New York; 2008. p. 502-5.

103 Wang L, Fan L, Zhao Z, Zhang Z, Jiang L, Chai M, et al. The Capparis spinosa var. herbacea genome provides the first genomic instrument for diversity and evolution study of Capparaceae family. Figshare Database. 2022. https://doi.org/10.6084/m9.figshare.17702051.

104 Wang L, Fan L, Zhao Z, Zhang Z, Jiang L, Chai M, et al. Supporting data for "The Capparis spinosa genome provides insight into genome evolution of Capparaceae". GigaScience Database. 2022. http://dx.doi.org/10.5524/102321.

Figure legends

Fig. 1. Growth of *Capparis spinosa* var. *herbacea* in wild collection sites. A. Mature *C. spinosa* var. *herbacea* plant. **B.** Flowers. **C.** Fruits. **D.** Stem. **E.** Leaf tip.

Fig. 2. High-quality assembly of 21 chromosomes. A. Chromosome ideograms. **B.** Transposable element (TE) repeat sequence density (window size 100 kb). **C.** Tandem repeat sequence density (100 kb window size). **D.** Gene density (100 kb window size). **E.** GC content (100 kb window size). **F.** Relationship between syntenic blocks.

Fig. 3. Gene duplication and evolution of *Capparis spinosa* var. *herbacea*. A. Number of genes and gene pairs of five duplication types. **B.** Distribution of Ka/Ks of five duplication types. **C.** Distribution of Ks of five duplication types. **D.** Distribution of 4DTv of five duplication types.

Fig. 4. Distribution of Ks, 4DTv, and ages of LTR of *Capparis spinosa* var. *herbacea* and other species. A. Ks distribution of *C. spinosa* var. *herbacea* and other representative species. **B.** 4DTv distribution of *C. spinosa* var. *herbacea* and other representative species. **C.** Ages of LTR of *C. spinosa* var. *herbacea* and other species (molecular clock r is $7*10^{-9}$).

Fig. 5. Evolution of the *Capparis spinosa* var. *herbacea* genome. A. Venn diagram of specific and shared orthologs among 16 species (*O. sativa*, *B. distachyon*, *A. comosus*, *M. acuminata*, *C. micranthum*, *N. nucifera*, *T. sinense*, *V. vinifera*, *S. lycopersicum*, *P. trichocarpa*, *T. cacao*, *C. spinosa* var. *herbacea*,

T. hassleriana, *A. thaliana*, *N. colorata*, and *A. trichopoda*) identified based on gene family cluster analysis. Each number in the diagram represents the number of gene families within a group. **B.** Expansion and contraction of gene families. **C.** GO enrichment analysis of genes from expanded families.

Figure S1. Genome size estimation of *Capparis spinosa* var. *herbacea* by using genome survey and flow cytometry with *Solanum pimpinellifolium* as reference.

A. The 17-mer distribution of Illumina short reads in *C. spinosa* var. *herbacea*. The x-axis shows the frequency or the number of times of a given k-mer (k-mer depth). The y-axis shows the total number of k-mers with a given frequency (a given depth). Two peaks (blue line) were observed indicating heterozygosity in *C. spinosa* var. *herbacea***. B.** Main peaks of *Solanum pimpinellifolium* and *C. spinosa* var. *herbacea* (samples 1 and 2) were 356.73 and 123.27 (mean value = (122.72 + 123.82)/2), respectively. According to the formula "peak (ref)/genome size (ref) = peak (*C. spinosa* var. *herbacea*)/genome size (*C. spinosa* var. *herbacea*)", the mean value of the genome size of *C. spinosa* var. *herbacea* was estimated as 279.07Mb.

Figure S2. Hi-C interaction heat map. Hi-C heat map of 21 chromosomes.

Figure S3. Correlation analysis of genomic distribution characteristics.

Gene: gene density; GC: GC content; TR: distribution of tandem repeats; TE: distribution of transposable elements.

A. Correlation of the genomic GC content, gene density, TE distribution, and TR distribution. **B**. Correlation analysis of TR distribution and GC content.

Figure S4. Enrichment analysis of positively selected genes in gene duplication types. A. GO enrichment analysis of positively selected genes in four duplication types. **B.** KEGG enrichment analysis

of positively selected genes in five duplication types.

Figure S5. *Capparis spinosa* var. *herbacea* genome collinearity analysis. A. Dot plots of paralogs in the *C. spinosa* var. *herbacea* genome. **B.** *A. trichopoda*, *C. spinosa* var. *herbacea*, and *A. thaliana* gene level collinearity analysis. **C.** *T. cacao* and *C. spinosa* var. *herbacea* gene level collinearity analysis. **D.** *A. thaliana and C. spinosa* var. *herbacea* gene level collinearity analysis. **E.** *V. vinifera*, *C. spinosa* var. *herbacea*, and *S. lycopersicum* genome level collinearity analysis. **F**. *T.hassleriana*, *C.spinosa var. herbacea*, and *G. gynandra* genome block level collinearity analysis(The *T.hassleriana* genome uses the longest Top 50 scaffolds. The order shown in the figure is the sequence numbering order after Top50 is extracted).

Figure S6. KEGG enrichment analysis. A. KEGG enrichment analysis of *C. spinosa* var. *herbacea* specific geness. **B.** KEGG enrichment analysis of expansion genes.
