# Supplementary material for: The Capparis spinosa var. herbacea genome provides the first genomic instrument for a diversity and evolution study of the Capparaceae family
Source: Gigascience. 2022 Oct 30;11:giac106. doi: 10.1093/gigascience/giac106 (PMC9618406; doi:10.1093/gigascience/giac106)
Supplement: giac106_Response_to_Reviewer_Comments_Original_Submission [file giac106_response_to_reviewer_comments_original_submission.pdf]

Author's Response To Reviewer Comments

Close

Reviewer reports:
Reviewer #1: The manuscript titled "Capparis spinosa genome provides insight into genome evolution of Capparaceae" reports the first reference genome assembled for Capparaceae family for the first time. Due to the current lacks in the genetic information about the species, this paper can be useful for future studies, however there are several gaps and unclear points in all section that the authors have to cover and modify.
Plant material
The first gap is related to the plant material sequenced. The C. spinosa species includes several subspecies and botanic varieties (such as C. spinosa subsp spinosa; C. spinosa subsp. rupestris, C. spinosa subsp spinosa var. spinosa, C. spinosa subsp spinosa var. herbacea…and more, see Fici et l. 2014, http://dx.doi.org/10.11646/phytotaxa.174.1.1). Therefore the authors must specify to which group the sequenced material belongs. Due to the complex origin of the different varieties, the generic "C. spinosa" is incorrect and has not meaning, more information is needed on the plant material used.
Reply: Thank you very much! The material samples of the assembled genome were deposited in the Herbarium of the Xinjiang Institute of Ecology and Geography, Chinese Academy of Sciences (XJBI). The samples used in this study were identified and confirmed by XJBI taxonomist Xiyong Wang. The species we sequence and assemble the genome is Capparis spinosa sub. spinosa var. herbacea. See line 333.
About the genome size evaluation, it is not clear what has been done. In figure S1, only the Capparis spinosa peaks are reported (two replicates). Usually both standard and target samples are included in the same run and the genome size is evaluated comparing the two peaks (also as reported in Doležel and Bartoš 2005, but also in Doležel et al. 1989...and more). The comparison between reference and target samples allows to have the genome size in pg, that can be directly convertible in Mb (1 pg = 978 Mb). Therefore, it is not clear how the genome size of "C. spinosa" sequenced (must to be specify what variety is) has been obtained. Please explain and add other information and references. This is another key point to evaluate the complexity of genome sequenced.
Reply: Thank you very much for your suggestion. First, flow cytometry experiment is a good method for genome size evaluation. There are two strategies for flow cytometry experiments, one as you mentioned where both standard and target samples are included in the same run, and the other is that the standard and target samples are run separately as in the articles Xie et al., 2020 (doi:10.1038/s41438-020-0328-y) so both strategies are feasible. We further read the peaks of the standard and target samples onto one graph to facilitate comparison of the two peaks to assess genome size.
Secondly, we did genome survey analysis and the results showed that the predicted size of Capparis spinosa var. herbacea genome was 245,974,629 bp, which turned out to be close to the size of the final genome assembled and proved the reliability of the genome size.
We have modified the content and images for the above results accordingly, please see line 376-390 and Fig S1.
Methods
The methods need to be improvement (see below) and revise, several sentence are pointless and unclear. In addition there is a lack in the annotation. Indeed, since the present paper is the first genome assembled in the Capparaceae family, the authors must to add the RNASeq data from different tissues (such as root, leaves, flowers, buds etc etc) to develop a correct and full annotation (this is mandatory).
Thank you very much! We performed second-generation Illumina and Pacbio third-generation transcriptome sequencing on mixed samples of roots, stems, leaves, flowers, and fruits for genome annotation. The relevant raw data have been uploaded to the NCBI SRA database, and the corresponding numbers are SRR18512706 and SRR18512705. At the same time, the relevant material information and transcriptome library and sequencing information are supplemented in line 343-346
Please add information on the genome size estimation protocols and methods.
There are not information about DNA extraction protocols used.
Thank you very much! We have added methods for genome size estimation, in line 376-390
We have supplemented the DNA extraction method at line 340-343.
The paragraph describing the PacBio library construction and sequencing is very short, providing only few information on the assembling process and on the pre-processing of reads. Please improve it, adding also information about the PacBio platform used.

Thank you very much! We have supplemented PacBio library construction information and added information on sequencing platforms in line 350-362 and Table 1.
The authors wrote "accounting for 100-fold coverage of the draft genome of C. spinosa.", what does it mean? I would think that this coverage was evaluated using the estimated genome size and not the draft genome that was not available in the sequencing phase.
Thank you very much! Yes, you are right. Here we use the genome size estimated from the genome survey to evaluate. We have revised the relevant representation in line 403-404 in the manuscript.
The authors wrote "Hi-C fragment libraries were constructed as reported by Fu et al. [37]"…please provide more detailed reference for the protocols used and add more details on the methods.
Thank you very much! We have revised the relevant representation in line 392-401.
The authors wrote "using LACHESIS [38] with the following parameters:…. C-LUSTER_MIN_RE_SITES=9;". Parameters here used are slightly different from the reference cited. Please, modify the sentences and add details on the parameters selection step.
Thank you very much! we have improved Hi-c related information in line 392-401.
"Clean Hi-C reads, accounting for 100-fold coverage of the draft genome of C. spinosa, and the final 28 scaffolds were anchored to chromosomes, accounting for 99.98% of the total length.". What does it mean? The sentence is unclear, please rephrase it.
Thank you very much! Here we use the genome size estimated from the genome survey to evaluate. We have revised the relevant representation in line 377-381.

In the Genome assembly and evaluation section the authors wrote "Align the short sequence obtained by Illumina HiSeq with the reference genome using the default parameters of the BWA-MEM V0.7.17 [41]. CEGMA V2.5 (default parameters) [42] database contains 458 conserved core genes in eukaryotes were used to evaluate the completeness of the final genome assembly. ". These sentence are very confusing, rewrite please.
Thank you very much! We have revised this sentence in line 415-419.
Repeat sequences identify: Rephrase the title, maybe Repeat sequences detection? Identification? Masking?
Thank you very much! We have modified it to "Identification of repeat sequences". See in line 430.
The authors wrote "By combining the above de novo TE sequence library and LTR-RTs library with the known Repbase V19.06, REXdb V3.0, and Dfam V3.2 databases, a non-redundant species-specific TE library was constructed" - This part is unclear. How did you combine the libraries? What do you mean with non-redundant species-specific?
We have revised the presentation here. Merging multiple databases is done using the shell "cat" command. Since predictions from multiple databases may be duplicated, redundancy needs to be removed. Redundancy is removed using seqkit software.see line 433-438.
In the Gene prediction and annotation the authors wrote "Three approaches, de novo prediction, homology search, and transcript-based assembly to annotate protein-coding genes, as described by Fu et al.[37]." - According to your statement, you used transcript-based annotation, but there is no mention to RNASeq sequencing in the paper (see my first and mandatory comment for the methods section). You once again cite Fu et al. 2021 where they explicitly state they used RNASeq for the prediction of genetic structure. Furthermore, I am not sure of your use of the HISAT2 tool in this context. Please explain.
We have added transcriptome-related information. Includes materials, library construction, and sequencing information. We did use HISAT2 for transcriptome analysis.
"Other software use default parameters." - Please be consistent, in other paragraphs you used the "(default parameters)" for all tools.
We have revised here to detail the software and methods used. See line 444-454.
"rRNA prediction was mainly based on Rfam V12.0 [60] database and Barrnap V0.9 [61] was predicted" - Please rewrite this sentence.
We have modified it. See line 459-464.
"GenDup_finder-unique the stricter version of". Please rephrase this sentence in the Whole-genome gene duplication analysis
We have revised it. See line 466-468.
"GO and KEGG enrichment analysis by clusterProfiler V4.2.0 [66]". What universe did you use for the enrichment analysis? This may heavily affect the enrichment.
Genes with Ka/Ks>1 in five replication modes were used for GO and KEGG enrichment analysis, respectively, and the results were visualized in one figure. We have revised it. See line 471-472.
"The encoding genes from a species were clustered into six groups, including 0 copies, 1 copy (single-copy), 2 copies, 3 copies, 4 copies, and 4+ copies. A total of 306 genes were identified as single-copy genes. The obtained gene families were annotated using the PANTHER V15 database [68]." - this section is unclear. Did you follow a pipeline already published?

Orthofinder can perform these analyses, classifying genes into the above 6 types. This is to illustrate the source of the single copy genes that follow. We used the same analytical sequencing as Fu et al. (https://doi.org/10.1038/s41438-021-00487-1), whether in genome assembly, annotation, or comparative genomics. Only some software types and versions used are slightly different. Therefore, we cite Fu's literature in many places in the text.
"Use MAFFT V7.205 [69] to align each single-copy gene family sequence (parameter: --localpair -maxiterate 1000), and then use Gblocks V0. 91b [70] (parameter: -b5=h) filter the conserved sites, and finally all aligned genes of each species the family sequence was connected end to end to get the supergene, and then use IQ-TREE V1.6.11 [71] model detection tool ModelFinder [72] for model detection, the best model obtained was JTT+F+I+G4, and then using this best model, the evolutionary tree was constructed by the maximum likelihood (ML) method, and the number of bootstrap was set to 1000." - This sentence is too long and should be split. Furthermore, the methodology is not clear and confusing.
We have modified it in line 485-492.
"with A. trichopoda as the outgroup of the root tree" - How did you choose the outgroup? Add reference.
We have revised it in line 494.
"divergence times were estimated as follows: A. trichopoda Vs S. lycopersicum at 164-194 MYA, O. sativa Vs B. distachyon at 42-60 MYA, A. comosus Vs O. sativa at 94-115 MYA, N. nucifera Vs V. vinifera at 116-127 MYA". How did you estimate these? And how did you use this information in the divergence time estimation? This part is confusing and should be rephrased.
We have rewritten this part of the statement in line 494-499.
Results
The PacBio sequencing allowed a fairly good scaffolding with an N50> of 15 Mb, and Hi-ci helped in the cleaning of PacBio sequences, notoriously know as long but low quality sequences….this point should be highlighted by the authors. However, although the Illumina Hi-C sequencing favored the anchoring of the scaffolds in the pseudo-chromosomes, the lack of genetic maps leaves the anchor a bit weak. Combined integration of optical mapping and Dovetail Genomics data could further advance the contiguity supporting the idea that optical mapping and Hi-C data are complementary and can help bridging different regions in the genome. (Wen-Biao Jiao ,et al. https://doi.org/10.1101/gr.213652.116 ;https://www.sciencedirect.com/science/article/pii/S1369526616301315)
In general the Genome sequencing and assembly paragraph is brief and provide only few information on the assembly results.
"The Hi-C reads were employed for scaffolding into 21 pseudo-chromosomes (Fig. 2, Fig. S2)" - Again, no info on how this was done. Furthermore, there is no image of the hi-c contact map generated.
We supplement Hi-c with more details in Methods in line 372-374. Hi-c contact map in Fig S2.
"Gene completeness reached up to 99.98%" - How did you evaluate this? Did you mean genome completeness?
Yes, you are right. We have revised it in line 100.
The genome quality assessment part is in the next part of the paragraph.
"indicating that the assembly quality of C. spinosa reached the level of the reference genome." What do you mean? Which reference genome?
Thank you very much! We have added detailed references and modified the description line 110-113.
In Identification of genomic repetitive sequences section the authors wrote "We found the correlation between the distribution of TR sequence and GC content on the chromosome in the C. spinosa genome (Fig.2 C, E). So, use Spearman's algorithm to calculate the correlation. The correlation coefficient is -0.52, and the P-value is 2.2e-16. It shows that the distribution of TR sequences in the C. spinosa genome and the GC content of the sequences show a significant negative correlation." There is no mention in the methods. Please add a description of the algorithm used for this task. Furthermore, the sentence is confusing and should be rephrased.
In fact, we performed a pairwise correlation analysis on the GC content of the genome, gene density, TE distribution, and TR distribution. In the end, only the correlation between GC content and TR distribution was found, and no correlation was found between others. We use a function `cor` of basic R to calculate correlation. Cor provides three methods to calculate correlation, namely "pearson", "kendall", "spearman". We normalize the data. The distribution test does not conform to the normal distribution , so pearson cannot be used. After testing the latter two, the spearman method is finally selected. We have added the description of correlations to the Methods. See in line 128-138 and 529-531.
As already stated in the methods section, the Genome annotation pipeline is lacking clarity. The same can be said for this section of the results. As reported in methods section, the authors must to sequence RNASeq libraries developed from different tissues, in order to have a correct annotation.
"and more than 98.82% of genes were annotated based on sequence similarity using the following protein-related databases". Here you give a brief description of the blast results, but in the methods you state that you used a much more complex method for the final annotation. Where are the results? You

should expand this paragraph, adding more information/details.
"Table 1." - This table should be moved to the genome assembly paragraph and a new table with annotation info should be produced (also as supplementary material).
Thank you for your suggestion. We adjusted the position of the Table1, and added a new table, Table S4, for the prediction statistics of genomic genes. At the same time, we revised the relevant sentences in the manuscript line 140-143.
The authors spoke about possible haplotypes in the duplication analysis, but the analysis is really weak. The authors have to improve this section using other instrument/comparing adding other information, such as the degree of heterozygosity.
In the same way, the evolutionary study is also weak. The authors have to improve it adding other comparing.
We have analyzed heterozygosity using genome survey. We have rewritten the results. See in line 202-226.

"a total of 19,063 colinear gene pairs on 409 colinear blocks were inferred between C. spinosa and A.thaliana. 5,501 colinear gene pairs from 17 colinear blocks detected between C. spinosa and A. trichopoda (Fig. S4B). 16,747 colinear gene pairs from 260 colinear blocks were detected between C. spinosa and T. cacao. 15,352 colinear gene pairs from 222 colinear blocks detected between C. spinosa and V. vinifera, and 17,940 colinear gene pairs from 502 colinear blocks were detected between C. spinosa and S. lycopersicum (Fig. S4E)". This could be summed up in a table to reduce the repetitive text.
We modify this content and organize the above information into Table S7. See in lines 189-193
"Next, the species divergence times of them were evaluated (Fig. 4B)"…please rewrite.
"Results showed that in almost species, except B. distachyon and A. thaliana, more gene families experienced expansion than contraction.". Please rephrase, and improve as suggested in a previous point.
In summary the authors have to improve all result sections (in the current form are really weak), adding other information through RNASeq experiment for the annotation.
We have added related experiments and descriptions. Please see the reply above.
Discussion
The discussion introduces a high number of genes and pathways that have not been listed/highlighted in the results. This is the consequence of the results chapter that is too short, unclear, and superficial. The improvement of the results section may aid the discussion section. Furthermore, the discussion lacks of a final conclusion chapter.
Reply: We have added the corresponding content in the results section and a conclusion section at the end of the article. Please see lines 325-330.
"The genome assembly was 274.53 Mb in length, and >99.23% of the assembled genome was placed on 21 chromosomes. This represents a more contiguous and higher-quality genome assembly than that of recently sequenced Brassicaceae species genomes, such as eight oilseed rape lines [20], with a contig N50 2.1~3.1 Mb, as well as field pennycress [21]". This sentence seems pretentious. The works cited shows higher Busco score and completeness percentage (98% and 95.9% respectively) and a three-fold size genome assembly. Furthermore the scaffold N50 of both works cited is significantly higher (~50 and ~70 Mb) then that reported for this assembly ~15 Mb.
Reply: Thank you very much for your suggestion. We have improved this section. Please see lines 261-266.
"At the same time, we also found that the distribution of TR in the genome of C. spinosa is inversely proportional to the content of GC. The local high GC content in the genome seriously affects the accuracy of Hi-C assembly. For example, the GC content of C. spinosa genome Chr06: 3700000-15800000 is 53.92%, which is much higher than the genome GC content 36.61%. High GC content may affect the accuracy of assembly of this segment on Chr06 chromosome. It may also be due to the high local GC content and high TR ratio that increase the difficulty of genome assembly." - This part is full of repetitions and could be improved.
Reply: We have improved this section and merged it with the first paragraph in order to achieve a more appropriate description. Please see lines 267-288.
Finally the paper needs a full language revision from a native English speaker.
We use professional editing companies (TopEdit www.topeditsci.com) to polish the language of the article.

Reviewer #2: I am extremely excited and pleased to finally see a Capparaceae genome assembly of high quality. Overall, I find this a straightforward report of a genome sequencing project with a few nuggets of interesting biology included (such as the high rate of Tandem repeats and their negative correlations with GC content). However, I have a few major concerns/questions for the authors as well as a few

minor suggestions for improvement.
Major questions:
1. There is almost no discussion about the past domestication history of Capparis spinosa. If this was done, then likely the authors would report that C. spinosa is thought to be of hybrid origin between two between C. orientalis and C. sicula (e.g. Rivera et al., 2002). The authors present no information on the degree of potential heterozygosity in their assembly and if for example som time of purge haplotypes steps was done. I would very much like to see some discussion about domestication and thus how past hybridisation was dealt with (this should be much more recent than the reported WGD).
Thank you very much ! The samples used in this study were identified and confirmed by Xinjiang Institute of Ecology and Geography, Chinese Academy of Sciences (XJBI) taxonomist Xiyong Wang. We performed genome surveys using illumina sequencing, and the estimated heterozygosity rate was 0.878%. A previous study indicated that C. spinosa is morphologically related to C. sicula Duhamel as well to C. orientalis Duhamel and overlaps with the latter (Inocencio et al., 2005). Recently a taxonomic revision has been conducted by Fici (2014, 2015) on the C. spinosa group widespread from the Mediterranean to central Asia. C. spinosa is recognized as a single species and is represented by four subspecies (i.e., C. spinosa subsp. spinosa; C. spinosa subsp. rupestris; C. spinosa subsp. cordifolia; C. spinosa subsp. himalayensis). C. spinosa subsp. spinosa is widely distributed eastwards from the Mediterranean to China and Nepal, showing inherited traits and great level of heterogeneity. Within this subspecies, some varieties are identified, namely var. herbacea and var. atlantica. (Stephanie Chedraoui et al., https://doi.org/10.3389/fpls.2017.01845). See in line 333 and 51-57.
2. The positioning of the WGD and the dating of divergence times is a bit confusing. There is quite some past work on the dating of say the At-alpha WGD in Brassicaceae and also the divergence of Brassicaceae from Cleomaceae and Capparaceae (e.g. see work for example of Jocelyn Hall). Also, placing the capparaceae duplication in light of Cleomaceae duplications (e.g. paper by Marbry et al 2021). Also, the "younger age" maybe due to Capparis being woody and most other compared species are herbaceous (except Grape). This is issue is known to cause dating problems and is well discussed in the literature.
Reply: Thank you very much for your suggestion. The complicated relationship between the three closely related families, Capparaceae, Cleomaceae, and Brassicaceae, has been extensively studied since the appearance of the theory of alternation of generation by Hofmeister (zayat et al., 2020). However the taxonomic relationships of these three families are still under discussion and much controversy exists, especially in different regions (zayat et al., 2020; Ahmadi, M and Saeidi, H, 2018). We believe that it is not rigorous to discuss the differentiation of the three families Capparaceae, Cleomaceae and Brassicaceae based only on the genomic information of C. spinosa, and it is more appropriate to analyze and discuss them after obtaining more genomic information of species in the family Capparaceae. At the same time when comparing the WGD of species, we are selecting species according to the evolutionary tree. And there are both woody and herbaceous, not only Grape, Theobroma cacao and Amborella trichopoda are also woody. We assembled species is Capparis spinosa sub. spinosa var. herbacea. And the evolutionary tree also shows that our species is relatively close to the herb Arabidopsis thaliana and Tarenaya hassleriana. We rewrote the content of WGD in line 183-219.
Ref
El zayat, M.A.S., Ali, M.E.S. & Amar, M.H. A systematic revision of Capparaceae and Cleomaceae in Egypt: an evaluation of the generic delimitations of Capparis and Cleome using ecological and genetic diversity. J Genet Eng Biotechnol 18, 58 (2020). https://doi.org/10.1186/s43141-020-00069-z
Ahmadi, M., Saeidi, H. Genetic diversity and structure of Capparis spinosa L. in Iran as revealed by ISSR markers. Physiol Mol Biol Plants 24, 483–491 (2018). https://doi.org/10.1007/s12298-018-0518-3
Fici S . A taxonomic revision of the Capparis spinosa group (Capparaceae) from the Mediterranean to Central Asia[J]. PHYTOTAXA, 2014, 2014,174(1)(-):1-24.

3. Ideally the seed material or at a minimum a herbarium sample of the genotype used for sequencing should be made clear. Can others access this material in some way? Also, having a line from a botanic garden means that plant came from somewhere else (e.g. in the Mediterranean basin). Where is this plant originally from? Also, there is a chance (see point 1) that this plant is NOT C. spinosa but another non-hybrid Capparis. Please have a skilled taxonomist check the plant and deposit a herbarium specimen.
The material samples of the assembled genome were deposited in the Herbarium of the Xinjiang Institute of Ecology and Geography, Chinese Academy of Sciences (XJBI). You can contact Lei Wang (The first author Email: egiwang@ms.xjb.ac.cn) for the original material.
Minor suggestions:
1. Do not use italics with family name: Capparaceae
Thank you very much! We have modified it.
2. When starting a sentence, don't say C. spinosa...then Capparis spinosa (e..g. line 50)

Thank you very much! We have modified it.
3. line 68: Change "Total..." to "A total..."
Thank you very much! We have modified it.
4. Line 114...this line "Gene footprints in the duplicate events were observed. " Is confusing to me (footprints in sequences often mean something else)...I suggest you just delete it.
Thank you very much! We have removed it in line 151.
5. You mention some chemicals used/present in Capparis, but never glucosinolates! This is the character that gives capers their flavour and make them clearly part of Brassicales.
Reply: Added. Please see lines 63-65.

Close
