# Supplementary material for: The Capparis spinosa var. herbacea genome provides the first genomic instrument for a diversity and evolution study of the Capparaceae family
Source: Gigascience. 2022 Oct 30;11:giac106. doi: 10.1093/gigascience/giac106 (PMC9618406; doi:10.1093/gigascience/giac106)
Supplement: giac106_Response_to_Reviewer_Comments_Revision_1 [file giac106_response_to_reviewer_comments_revision_1.pdf]

Author's Response To Reviewer Comments

Close

GIGA-D-22-00058R1
The Capparis spinosa genome provides insight into genome evolution of Capparaceae
Lei Wang; Liqiang Fan; Zhenyong Zhao; Zhibin Zhang; Li Jiang; Mao Chai; Changyan Tian
GigaScience

Reviewer reports:
Reviewer #1: The authors improved the manuscript in object, following the comments. However there are still some points that need to be improved.

- What taxonomic revision has been used to define the species sequenced? Although the authors have been supported by an expert on taxonomy, since there are different visions, they have to cite some references describing the taxonomy of the genus. In addition, the authors have to change in the title, text, all figures and tables C. spinosa whit C. spinosa sub spinosa var. herbacea (or an acronym) to highlight immediately the variety studied.

Reply: We apologize for this mistake and thank you very much for your suggestion. The full taxonomic name of the material used to assemble the genome is Capparis spinosa var. herbacea (Willd.) (NCBI:txid2717819), so it should be stated in the manuscript as Capparis spinosa var. herbacea or C. spinosa var. herbacea, not C. spinosa. We have revised the manuscript in all places accordingly. Moreover, we have added information on the identification of the samples to the manuscript.
Plant materials and nucleic acid extraction (Lines 294-303)
The source plant (Fig. 1) is an individual of Capparis spinosa var. herbacea (Willd.) Fici (NCBI:txid2717819) collected from the wild in Gaochang District, Turpan City, China (42°55' N, 89°10' E) and identified and confirmed by taxonomist Xiyong Wang of Xinjiang Institute of Ecology and Geography, Chinese Academy of Sciences. The material samples of the assembled genome were deposited in the Specimen Museum of Xinjiang Institute of Ecology and Geography, Chinese Academy of Sciences, Urumqi 830011, China (NO. XJBI 00108198).
Discussion (Lines 214-221)
It is well known that Capparis spinosa has many subspecies and varieties [19, 25], and the identification of samples is often controversial. According to the taxonomic characteristics of C. spinosa var. herbacea, the branchlets are usually white-tomentose in the upper part and the stipules are straight, horizontal or slightly curved, and yellowish [5]. The samples used for genome sequencing in this study matched the above taxonomic characteristics (Fig. 1D). Besides, the location where the samples were collected in this study is consistent with the geographical distribution of C. spinosa var. herbacea in China reported by Maurya et al. [19]. Based on the above, there is no dispute that the species used for genome sequencing in this study was Capparis spinosa var. herbacea.

- I agree that the flow cytometry approach is a good method to define the genome size. However in the work that the authors cited (doi:10.1038/s41438-020-0328-y), Xie and collaborators used Dolezel, J. & Bartos, 2005, where Soybean was used as internal references standard, therefore in the same run and not separately. Therefore it is not clear what is the method where "the standard and target samples are run separately", please explain.
Reply: We are very sorry that our last reply was not very clear. When reference sample and experimental samples are mixed for flow cytometry, the peaks obtained are on the same graph, and this belongs to the internal reference method. When this method was used in this study, there seemed to be interference between the fluorescence signals of the reference sample and the experimental samples and this could not be successfully performed, so the external reference method was used, i.e. immediately after detecting the fluorescence signal of the reference sample, experimental sample 1 and experimental sample 2 were tested in turn. The internal environment of the CyFlow Space flow cytometer was identical throughout the experiment, and the amplification system kept at a constant voltage and gain throughout the analysis. Although the final peak plots are presented separately and not combined, the comparison between the results is reliable.
The study by Xie et al., 2020 mentioned in our last response presented their flow cytometry results plot

(Figure S1) in the same way as ours, so we speculate that the authors may have used the same strategy as we did. The actual methods, instrumentation and software used for flow cytometry in our study were described in our manuscript. Please see lines 349-355.

In addition it is not clear how the authors have done a "survey analysis" for the genome size prediction of Capparis spinosa var. herbacea. If it a posterior prediction (after sequencing), the value is only an rough estimate. Please add details!
Reply: Thank you very much for your suggestion. We have added the sentence "We estimated genome size using genome survey and flow cytometry before genome assembly, respectively. Genomic DNA was re-sequenced using the Illumina NovaSeq 6000 sequencing platform and a total of 33.09 G of data was obtained for genome survey." to the manuscript, please see lines 340-342.

Finally, it is not clear how the authors obtained the value of 276.44 Mb. Since the ref is Solanum pimpinellifolium, with genome size of 923 Mb (Wang et al. 2020 - https://www.nature.com/articles/s41467-020-19682-0) and peak 356.73, and the peak of C. spinosa sub. spinosa var. herbacea is 123.27, following the formula "peak (ref)/genome size (ref) = peak (Capparis spinosa)/genome size (Capparis spinosa)" the value is 318,94 Mb, therefore the coverage obtained and genome completeness is nearly 77% (and not the 99.98%). Please explain and/or modify all sentences and statistics related to this point.
Reply: Thank you very much for your query. When performing the formula conversion, the reference genome size was selected as the genome size determined after 453 contigs assembly (807.6 Mb, Supplementary Data 1), rather than selecting the results of the genome survey (923 Mb, Supplementary Fig. 1) and 385 contigs were clustered into 12 pseudomolecules (800M, Fig. 1a).
Genome survey results are generally not that accurate and therefore need to be cross-validated with flow cytometry results. If the reference genome size is selected for genome survey (923 Mb), the results obtained from the conversion are no longer relevant.
In previous version, we used 800M as the reference genome size. This is the size of the genome at the chromosome level, discarding some unassembled contigs, so it is smaller than the actual genome size. As the authors state in their paper "Finally, 385 contigs with a total length of ~800 Mb, accounting for 99.0% of the assembly, were clustered into 12 pseudomolecules", we used 807.6 M as the reference genome size, which was converted by the formula to obtain a genome size of 279.07, accounting for 98.37% of the assembly.

- Regarding the transcriptomic data, why in the previous version this data was not included? Regarding the material, usually the tissues are sequenced both separately and mixed, and the mix is developed without leaves, because this tissue shows the more representative genes, therefore the genes with low expression in other tissues could be missed. Please explain the choice.
Reply: Thank you very much for your query. We are very sorry that we did not describe the method of genome coding gene prediction clearly in the previous version of the manuscript. In fact, we have used transcriptome sequencing data from the very beginning, and due to cost saving considerations, the transcriptome data was used in a mixed pool and no additional leaves were sequenced separately.

The authors improved the annotation using the RNASeq data, but it is strange that the genes identified is lower (in number) than the blast obtained from the other approaches used in previous version. Explain and/or check. It is also singular that using the RNASeq data for the annotation, the results showed in this version (e.g. Figure 4) are the same of previous version, in term of Ks, ortholog genes etc. Check please.
Reply: Thank you very much for your suggestion. As mentioned in the previous reply, transcriptome data has been used for genome-encoded gene prediction from the very beginning. The number of coding genes predicted by the transcriptome is lower than by other means, which may indeed be the case as you suggest, as the leaf samples are mixed with other tissues and the gene expression in the leaves masks the low expressed genes in the other tissues.

- Finally the discussion must to be improved. In particular the authors have to better compare their results to other genomes available and they have to add other information about the genes isolated, e.g. in the present version there is only one section for terpenoids (three lines) and HSP and nothing else…did the authors find only these genes of interest? Also the evolutionary history is weak, please improve this section.
Reply: Thank you very much for your suggestion. In the discussion section, we first add a discussion on Capparis spinosa var. herbacea to clearly articulate its taxonomic information. Secondly, we have added a discussion on the differentiation of the species and the WGD related to Capparis spinosa var. herbacea. Finally, we have added a discussion of the results of KEGG related to abiotic stress responses in Capparis

spinosa var. herbacea. Please see lines 238-242, 250-273.

Reviewer #2: As reviewer #2 from the first round, I am not at all satisfied with the efforts of the authors to improve the manuscript based on my suggestions last time. It is not enough to simply write rebuttal pararaphs in the response to the reviewers, I would have expected and wanted actual changes to the manuscript.
Again, I do not think the actual subspecies sequenced or info about domestication are sufficiently covered in the actual manuscript. There needs to be more info given and explained.
Reply: Thank you very much for your suggestion. The full taxonomic name of the material used to assemble the genome is Capparis spinosa var. herbacea (Willd.) (NCBI:txid2717819), so it should be stated in the manuscript as Capparis spinosa var. herbacea or C. spinosa var. herbacea, not C. spinosa. We have revised the manuscript in all places accordingly.
Plant materials and nucleic acid extraction (Lines 295-303)
The source plant (Fig. 1) is an individual of Capparis spinosa var. herbacea (Willd.) Fici (NCBI:txid2717819) collected from the wild in Gaochang District, Turpan City, China (42°55' N, 89°10' E) and identified and confirmed by taxonomist Xiyong Wang of Xinjiang Institute of Ecology and Geography, Chinese Academy of Sciences. The material samples of the assembled genome were deposited in the Specimen Museum of Xinjiang Institute of Ecology and Geography, Chinese Academy of Sciences, Urumqi 830011, China (NO. XJBI 00108198).
Discussion (Lines 214-221)
It is well known that Capparis spinosa has many subspecies and varieties [19, 25], and the identification of samples is often controversial. According to the taxonomic characteristics of C. spinosa var. herbacea, the branchlets are usually white-tomentose in the upper part and the stipules are straight, horizontal or slightly curved, and yellowish [5]. The samples used for genome sequencing in this study matched the above taxonomic characteristics (Fig. 1D). Besides, the location where the samples were collected in this study is consistent with the geographical distribution of C. spinosa var. herbacea in China reported by Maurya et al. [19]. Based on the above, there is no dispute that the species used for genome sequencing in this study was Capparis spinosa var. herbacea.

Also, the question of if there is a family Capparaceae or not is not controversial or uncertain. It is now accepted by the Angiosperm Phylogeny Group and therefore has to be adopted in this paper (I strongly believe).
Reply: Thank you very much! We have removed these sentences. Please see lines 67.

Also, data is given in paper for Cleome spinosa, but after taxonomic revisions this is now known to be Tarenaya hasseleriana...therefore this needs to be updated as well.
Reply: Thank you very much! We have removed it.

Similarly, it is not enough to tell me the reviewer that a herbarium specimen exists and that I can write the botanic garden...It must be clearly stated in the paper and include exactly where the herbarium specimen can be found (e.g. what is the herbarium and accession sheet).
Reply: Thank you very much for your suggestion. We have added relevant information in the section "Methods" of the manuscript.
Plant materials and nucleic acid extraction (Lines 295-303)
The source plant (Fig. 1) is an individual of Capparis spinosa var. herbacea (Willd.) Fici (NCBI:txid2717819) collected from the wild in Gaochang District, Turpan City, China (42°55' N, 89°10' E) and identified and confirmed by taxonomist Xiyong Wang of Xinjiang Institute of Ecology and Geography, Chinese Academy of Sciences. The material samples of the assembled genome were deposited in the Specimen Museum of Xinjiang Institute of Ecology and Geography, Chinese Academy of Sciences, Urumqi 830011, China (NO. XJBI 00108198).

Close
