# Supplementary material for: The Capparis spinosa var. herbacea genome provides the first genomic instrument for a diversity and evolution study of the Capparaceae family
Source: Gigascience. 2022 Oct 30;11:giac106. doi: 10.1093/gigascience/giac106 (PMC9618406; doi:10.1093/gigascience/giac106)
Supplement: giac106_Response_to_Reviewer_Comments_Revision_2 [file giac106_response_to_reviewer_comments_revision_2.pdf]

Author's Response To Reviewer Comments

Close

Dear Editors and Reviewers:
GIGA-D-22-00058R2
The Capparis spinosa var. herbacea genome provides insight into Capparaceae genome evolution
Lei Wang; Liqiang Fan; Zhenyong Zhao; Zhibin Zhang; Li Jiang; Mao Chai; Changyan Tian
GigaScience

In this version of revised manuscript, we have addressed carefully all the concerns of reviewer 1 point by point, mainly inc
the new addition of comparative genome analysis of T.hassleriana and G.gynandra, and the redrawing of Fig4A and B to m
the lines more easily distinguishable. Besides, we have redrawn FigS5E and added FigS5F, both drawn with collinear block
number of collinear gene pairs and blocks of newly added species has been added to TableS7. The data download address
the species involved in the comparative genomes analysis have been added to TableS9. We hope our revisions will meet t
expectation of editor and reviewer.

Reviewer reports:
Reviewer #1: The authors improved the paper following the suggestions provided in the last revision. However there are s
some lacks.
The authors explained how the genome size estimation has been carried out, using the contigs assembly from Wang et al.
(807.6 Mb). I am not completely agree, but the choice can be accepted.
The description about annotation was unclear in the previous version and, probably, with some missing data. Now the aut
used RNASeq data for the genes prediction (in my opinion new data, because all information about this point - methods, r
discussion - were previously missed in all sections). Unfortunately the section reported for genes prediction and annotatio
("Genome coding gene prediction and annotation") are still poor. The authors have to add some additional information, th
be used also in the discussion.
Reply: Thank you very much! We have added a description of the PacBio full-length transcripts analysis method to the Ma
Methods section, as follows: "A total of 1.52Gb of PacBio transcriptome data was used for full-length transcriptome sequen
analysis using PacBio Iso Seq3." Please see lines 396-397 in the revised manuscript.

In addition, I suggest to the authors to change the title. I am not agree about the possibility that the paper is able to "pro
insight into Capparaceae genome evolution". The authors describe the first genome in the C. spinosa group without strong
comparison in its and/or sister families (due to also the lack of information available). Therefore, although this study is an
interest beginning point, that provides new information for future studies, the title is pretentious. The authors need to cha
in (e.g.) "The Capparis spinosa var. herbacea genome provides the first genomic instrument for diversity and evolution stu
Capparaceae family"…or something similar.
Reply: Thank you very much for your suggestion. We believe that your suggestion is well suited to this study and therefor
changed the title to "The Capparis spinosa var. herbacea genome provides the first genomic instrument for diversity and
evolution study of Capparaceae family".

The authors have to improve the Background section, adding some information about the distribution of C. spinosa var he
in the world, in order to justify why the authors have chosen this variety.
Reply: Thank you very much. We have added a description of the worldwide distribution of C. spinosa var herbacea, as fol
"C. spinosa var. herbacea is mainly distributed in Turkey, Kazakhstan, Uzbekistan, Afghanistan and western China [7]. Ac
to the morphological characteristics of China reported by previous researchers [5], C. spinosa var. herbacea is mainly dist
in Xinjiang, Tibet and Gansu in China." Please see lines 57-60 in the revised manuscript.

In the "Analyses of genome synteny and WGD" (line 161-172), using the species compared the results obtained is obvious
syntenic analysis results also showed more collinear gene pairs between C. spinosa var. herbacea and A. thaliana (Table S
indicating that C. spinosa var. herbacea has a close evolutionary relationship with A. thaliana"). In my opinion to have mo
information the authors have to add in the same analysis also T. hassleriana (in this version it is used only for LTR insertic
evaluation) and Gynandropsis gynandra, both belonging to Cleomaceae. The last one should be also added at the gene fa
expansion and contraction evaluation and in the phylogenetic analysis and species divergence time estimation.
Reply: Thank you very much for your suggestion. We added the collinear analysis of T. hassleriana vs C. spinosa var. herb
and Gynandropsis gynandra vs C. spinosa var. herbacea to FigS5F. At the same time, the Ks and 4DTv analysis of Gynand
gynandra vs C. spinosa var. herbacea was newly added to Fig4 A-B and the peak value of Ks was used to evaluate the div

time. A description of the above results is shown below "The results showed that C. spinosa var. herbacea separated from cacao, G. gynandra, A. thaliana, and T. hassleriana in order at 93.10 MYA (Ks peak of 1.644 and 4DTv peak of 0.336), 58 MYA (Ks peak of 1.032 and 4DTv peak of 0.266), 53.00 MYA (Ks peak of 0.936 and 4DTv peak of 0.254), and 43.31 MYA peak of 0.765 and 4DTv peak of 0.220). Besides, C. spinosa var. herbacea experienced an α WGD event at 18.59 MYA (Ks at 0.328).". Please see lines 181-189 in the revised manuscript.
Our newly added Ks, 4DTv and collinearity analysis showed that the divergence of C. spinosa var. herbacea from T.hassle and A.thaliana occurred later than that of C. spinosa var. herbacea and G.gynandra. This indicates a closer evolutionary relationship between T.hassleriana, A.thaliana and C. spinosa var. herbacea.

Finally, in the "Data availability" all links included in this version don't work. In addition the sequences obtained from transcriptome (both long and short reads) has not been deposited and/or the link has not been reported. The authors mus provide all information/data/results developed (this is mandatory).
Reply: Thank you very much! Due to the long review cycle, some of the links initially provided may not be accessible. Her re-provide the latest accessible links. We will release the data once the manuscript is accepted. Until then, the data access in the manuscript are temporarily unavailable. You can view the data using the reviewer link we have provided. In the rev manuscript, we have added SRR numbers for specific data, as follows: "Raw data of genome assembly (PacBio HiFi and Hi sequences) and genome encoding gene prediction annotations (Illumina and PacBio sequences) have been deposited in th Sequence Read Archive (SRA) database under Bioproject ID: PRJNA792936. Illumina data SRR18458236 for genome size survey. Data for genome PacBio HiFi data SRR17373648, Hi-C data SRR17373647. For encoding gene prediction annotatio Illumina data SRR18512706, PacBio data SRR18512705.". Please see lines 464-468, 472-473.

The reviewer links are listed below:
PRJNA792936
https://dataview.ncbi.nlm.nih.gov/object/PRJNA792936?reviewer=i6b35k4shugbjhh7cj038hoddt
NGDC
https://ngdc.cncb.ac.cn/gwh/Assembly/reviewer/ABXaDepVlMevgXXRUQKeTtPRmIRoiRwtqnjlWkSsWpxHlSfDWyDGzkmljzv
Figshare
https://figshare.com/s/705006848f5610ac5ea9
FlowCytometry
http://flowrepository.org/id/RvFrcGmI9K1WuA4tX6QXzVuqZjkYYDUCq87SWDDLn6TKFpGYx8fKcNPheBNepSLi

Close
