# Supplementary material for: The Capparis spinosa var. herbacea genome provides the first genomic instrument for a diversity and evolution study of the Capparaceae family
Source: Gigascience. 2022 Oct 30;11:giac106. doi: 10.1093/gigascience/giac106 (PMC9618406; doi:10.1093/gigascience/giac106)
Supplement: giac106_Reviewer_1_Report_Original_Submission — Francesco Mercati -- 4/20/2022 Reviewed [file giac106_reviewer_1_report_original_submission.pdf]

Reviewer Report

Title: T he Capparis spinosa var. herbacea genome provides the first genomic instrument for diversity and evolution study of Capparaceae family

Version: Original Submission **Date:** 4/20/2022

Reviewer name: Francesco Mercati

Reviewer Comments to Author:

The manuscript titled "Capparis spinosa genome provides insight into genome evolution of Capparaceae" reports the first reference genome assembled for Capparaceae family for the first time. Due to the current lacks in the genetic information about the species, this paper can be useful for future studies, however there are several gaps and unclear points in all section that the authors have to cover and modify.

Plant material

The first gap is related to the plant material sequenced. The C. spinosa species includes several subspecies and botanic varieties (such as C. spinosa subsp spinosa; C. spinosa subsp. rupestris, C. spinosa subsp spinosa var. spinosa, C. spinosa subsp spinosa var. herbacea…and more, see Fici et l. 2014, http://dx.doi.org/10.11646/phytotaxa.174.1.1). Therefore the authors must specify to which group the sequenced material belongs. Due to the complex origin of the different varieties, the generic "C. spinosa" is incorrect and has not meaning, more information is needed on the plant material used. About the genome size evaluation, it is not clear what has been done. In figure S1, only the Capparis spinosa peaks are reported (two replicates). Usually both standard and target samples are included in the same run and the genome size is evaluated comparing the two peaks (also as reported in DoleÅ¾el and BartoÅ¡ 2005, but also in DoleÅ¾el et al. 1989...and more). The comparison between reference and target samples allows to have the genome size in pg, that can be directly convertible in Mb (1 pg = 978 Mb). Therefore, it is not clear how the genome size of "C. spinosa" sequenced (must to be specify what variety is) has been obtained. Please explain and add other information and references. This is another key point to evaluate the complexity of genome sequenced.

Methods

The methods need to be improvement (see below) and revise, several sentence are pointless and unclear. In addition there is a lack in the annotation. Indeed, since the present paper is the first genome assembled in the Capparaceae family, the authors must to add the RNASeq data from different tissues (such as root, leaves, flowers, buds etc etc) to develop a correct and full annotation (this is mandatory).

Please add information on the genome size estimation protocols and methods.

There are not information about DNA extraction protocols used.

The paragraph describing the PacBio library construction and sequencing is very short, providing only few information on the assembling process and on the pre-processing of reads. Please improve it, adding also information about the PacBio platform used.

The authors wrote "accounting for 100-fold coverage of the draft genome of C. spinosa.", what does it mean? I would think that this coverage was evaluated using the estimated genome size and not the

draft genome that was not available in the sequencing phase.
The authors wrote "Hi-C fragment libraries were constructed as reported by Fu et al. [37]"…please provide more detailed reference for the protocols used and add more details on the methods.
The authors wrote "using LACHESIS [38] with the following parameters:…. C-LUSTER_MIN_RE_SITES=9;". Parameters here used are slightly different from the reference cited. Please, modify the sentences and add details on the parameters selection step.
"Clean Hi-C reads, accounting for 100-fold coverage of the draft genome of C. spinosa, and the final 28 scaffolds were anchored to chromosomes, accounting for 99.98% of the total length.". What does it mean? The sentence is unclear, please rephrase it.
In the Genome assembly and evaluation section the authors wrote "Align the short sequence obtained by Illumina HiSeq with the reference genome using the default parameters of the BWA-MEM V0.7.17 [41]. CEGMA V2.5 (default parameters) [42] database contains 458 conserved core genes in eukaryotes were used to evaluate the completeness of the final genome assembly. ". These sentence are very confusing, rewrite please.
Repeat sequences identify: Rephrase the title, maybe Repeat sequences detection? Identification? Masking?
The authors wrote "By combining the above de novo TE sequence library and LTR-RTs library with the known Repbase V19.06, REXdb V3.0, and Dfam V3.2 databases, a non-redundant species-specific TE library was constructed" - This part is unclear. How did you combine the libraries? What do you mean with non-redundant species-specific?
In the Gene prediction and annotation the authors wrote "Three approaches, de novo prediction, homology search, and transcript-based assembly to annotate protein-coding genes, as described by Fu et al.[37]." - According to your statement, you used transcript-based annotation, but there is no mention to RNASeq sequencing in the paper (see my first and mandatory comment for the methods section). You once again cite Fu et al. 2021 where they explicitly state they used RNASeq for the prediction of genetic structure. Furthermore, I am not sure of your use of the HISAT2 tool in this context. Please explain.
"Other software use default parameters." - Please be consistent, in other paragraphs you used the "(default parameters)" for all tools.
"rRNA prediction was mainly based on Rfam V12.0 [60] database and Barrnap V0.9 [61] was predicted" - Please rewrite this sentence.
"GenDup_finder-unique the stricter version of". Please rephrase this sentence in the Whole-genome gene duplication analysis
"GO and KEGG enrichment analysis by clusterProfiler V4.2.0 [66]". What universe did you use for the enrichment analysis? This may heavily affect the enrichment.
"The encoding genes from a species were clustered into six groups, including 0 copies, 1 copy (single-copy), 2 copies, 3 copies, 4 copies, and 4+ copies. A total of 306 genes were identified as single-copy genes. The obtained gene families were annotated using the PANTHER V15 database [68]." - this section is unclear. Did you follow a pipeline already published?
"Use MAFFT V7.205 [69] to align each single-copy gene family sequence (parameter: --localpair -maxiterate 1000), and then use Gblocks V0. 91b [70] (parameter: -b5=h) filter the conserved sites, and finally all aligned genes of each species the family sequence was connected end to end to get the supergene, and then use IQ-TREE V1.6.11 [71] model detection tool ModelFinder [72] for model

detection, the best model obtained was JTT+F+I+G4, and then using this best model, the evolutionary tree was constructed by the maximum likelihood (ML) method, and the number of bootstrap was set to 1000." - This sentence is too long and should be split. Furthermore, the methodology is not clear and confusing.

"with A. trichopoda as the outgroup of the root tree" - How did you choose the outgroup? Add reference.

"divergence times were estimated as follows: A. trichopoda Vs S. lycopersicum at 164-194 MYA, O. sativa Vs B. distachyon at 42-60 MYA, A. comosus Vs O. sativa at 94-115 MYA, N. nucifera Vs V. vinifera at 116-127 MYA". How did you estimate these? And how did you use this information in the divergence time estimation? This part is confusing and should be rephrased.

Results

The PacBio sequencing allowed a fairly good scaffolding with an N50> of 15 Mb, and Hi-ci helped in the cleaning of PacBio sequences, notoriously know as long but low quality sequences….this point should be highlighted by the authors. However, although the Illumina Hi-C sequencing favored the anchoring of the scaffolds in the pseudo-chromosomes, the lack of genetic maps leaves the anchor a bit weak.

In general the Genome sequencing and assembly paragraph is brief and provide only few information on the assembly results.

"The Hi-C reads were employed for scaffolding into 21 pseudo-chromosomes (Fig. 2, Fig. S2)" - Again, no info on how this was done. Furthermore, there is no image of the hi-c contact map generated.

"Gene completeness reached up to 99.98%" - How did you evaluate this? Did you mean genome completeness? The genome quality assessment part is in the next part of the paragraph.

"indicating that the assembly quality of C. spinosa reached the level of the reference genome." What do you mean? Which reference genome?

In Identification of genomic repetitive sequences section the authors wrote "We found the correlation between the distribution of TR sequence and GC content on the chromosome in the C. spinosa genome (Fig.2 C, E). So, use Spearman's algorithm to calculate the correlation. The correlation coefficient is -0.52, and the P-value is 2.2e-16. It shows that the distribution of TR sequences in the C. spinosa genome and the GC content of the sequences show a significant negative correlation." There is no mention in the methods. Please add a description of the algorithm used for this task. Furthermore, the sentence is confusing and should be rephrased.

As already stated in the methods section, the Genome annotation pipeline is lacking clarity. The same can be said for this section of the results. As reported in methods section, the authors must to sequence RNASeq libraries developed from different tissues, in order to have a correct annotation.

"and more than 98.82% of genes were annotated based on sequence similarity using the following protein-related databases". Here you give a brief description of the blast results, but in the methods you state that you used a much more complex method for the final annotation. Where are the results? You should expand this paragraph, adding more information/details.

"Table 1." - This table should be moved to the genome assembly paragraph and a new table with annotation info should be produced (also as supplementary material).

The authors spoke about possible haplotypes in the duplication analysis, but the analysis is really weak. The authors have to improve this section using other instrument/comparing adding other information, such as the degree of heterozygosity.

In the same way, the evolutionary study is also weak. The authors have to improve it adding other comparing.
"a total of 19,063 colinear gene pairs on 409 colinear blocks were inferred between C. spinosa and A.thaliana. 5,501 colinear gene pairs from 17 colinear blocks detected between C. spinosa and A. trichopoda (Fig. S4B). 16,747 colinear gene pairs from 260 colinear blocks were detected between C. spinosa and T. cacao. 15,352 colinear gene pairs from 222 colinear blocks detected between C. spinosa and V. vinifera, and 17,940 colinear gene pairs from 502 colinear blocks were detected between C. spinosa and S. lycopersicum (Fig. S4E)". This could be summed up in a table to reduce the repetitive text.
"Next, the species divergence times of them were evaluated (Fig. 4B)"…please rewrite.
"Results showed that in almost species, except B. distachyon and A. thaliana, more gene families experienced expansion than contraction.". Please rephrase, and improve as suggested in a previous point.
In summary the authors have to improve all result sections (in the current form are really weak), adding other information through RNASeq experiment for the annotation.
Discussion
The discussion introduces a high number of genes and pathways that have not been listed/highlighted in the results. This is the consequence of the results chapter that is too short, unclear, and superficial. The improvement of the results section may aid the discussion section. Furthermore, the discussion lacks of a final conclusion chapter.
"The genome assembly was 274.53 Mb in length, and >99.23% of the assembled genome was placed on 21 chromosomes. This represents a more contiguous and higher-quality genome assembly than that of recently sequenced Brassicaceae species genomes, such as eight oilseed rape lines [20], with a contig N50 2.1~3.1 Mb, as well as field pennycress [21]". This sentence seems pretentious. The works cited shows higher Busco score and completeness percentage (98% and 95.9% respectively) and a three-fold size genome assembly. Furthermore the scaffold N50 of both works cited is significantly higher (~50 and ~70 Mb) then that reported for this assembly ~15 Mb.
"At the same time, we also found that the distribution of TR in the genome of C. spinosa is inversely proportional to the content of GC. The local high GC content in the genome seriously affects the accuracy of Hi-C assembly. For example, the GC content of C. spinosa genome Chr06: 3700000-15800000 is 53.92%, which is much higher than the genome GC content 36.61%. High GC content may affect the accuracy of assembly of this segment on Chr06 chromosome. It may also be due to the high local GC content and high TR ratio that increase the difficulty of genome assembly." - This part is full of repetitions and could be improved.
Finally the paper needs a full language revision from a native English speaker.

Level of Interest

Please indicate how interesting you found the manuscript: Choose an item.

Quality of Written English

Please indicate the quality of language in the manuscript: Choose an item.

Declaration of Competing Interests

Please complete a declaration of competing interests, considering the following questions:

- Have you in the past five years received reimbursements, fees, funding, or salary from an organisation that may in any way gain or lose financially from the publication of this manuscript, either now or in the future?
- Do you hold any stocks or shares in an organisation that may in any way gain or lose financially from the publication of this manuscript, either now or in the future?
- Do you hold or are you currently applying for any patents relating to the content of the manuscript?
- Have you received reimbursements, fees, funding, or salary from an organization that holds or has applied for patents relating to the content of the manuscript?
- Do you have any other financial competing interests?
- Do you have any non-financial competing interests in relation to this paper?

If you can answer no to all of the above, write 'I declare that I have no competing interests' below. If your reply is yes to any, please give details below.

I declare that I have no competing interests

I agree to the open peer review policy of the journal. I understand that my name will be included on my report to the authors and, if the manuscript is accepted for publication, my named report including any attachments I upload will be posted on the website along with the authors' responses. I agree for my report to be made available under an Open Access Creative Commons CC-BY license (http://creativecommons.org/licenses/by/4.0/). I understand that any comments which I do not wish to be included in my named report can be included as confidential comments to the editors, which will not be published.

Choose an item.

To further support our reviewers, we have joined with Publons, where you can gain additional credit to further highlight your hard work (see: https://publons.com/journal/530/gigascience). On publication of this paper, your review will be automatically added to Publons, you can then choose whether or not to claim your Publons credit. I understand this statement.

Yes Choose an item.
