# Supplementary material for: The Capparis spinosa var. herbacea genome provides the first genomic instrument for a diversity and evolution study of the Capparaceae family
Source: Gigascience. 2022 Oct 30;11:giac106. doi: 10.1093/gigascience/giac106 (PMC9618406; doi:10.1093/gigascience/giac106)
Supplement: giac106_Reviewer_1_Report_Revision_1 — Francesco Mercati -- 7/11/2022 Reviewed [file giac106_reviewer_1_report_revision_1.pdf]

Reviewer Report

Title: T he Capparis spinosa var. herbacea genome provides the first genomic instrument for diversity and evolution study of Capparaceae family

Version: Revision 1 **Date:** 7/11/2022

Reviewer name: Francesco Mercati

Reviewer Comments to Author:

The authors improved the manuscript in object, following the comments. However there are still some points that need to be improved.
- What taxonomic revision has been used to define the species sequenced? Although the authors have been supported by an expert on taxonomy, since there are different visions, they have to cite some references describing the taxonomy of the genus. In addition, the authors have to change in the title, text, all figures and tables C. spinosa whit C. spinosa sub spinosa var. herbacea (or an acronym) to highlight immediately the variety studied.
- I agree that the flow cytometry approach is a good method to define the genome size. However in the work that the authors cited (doi:10.1038/s41438-020-0328-y), Xie and collaborators used Dolezel, J. & Bartos, 2005, where Soybean was used as internal references standard, therefore in the same run and not separately. Therefore it is not clear what is the method where "the standard and target samples are run separately", please explain.
In addition it is not clear how the authors have done a "survey analysis" for the genome size prediction of
Capparis spinosa var. herbacea. If it a posterior prediction (after sequencing), the value is only an rough estimate. Please add details!
Finally, it is not clear how the authors obtained the value of 276.44 Mb. Since the ref is Solanum pimpinellifolium, with genome size of 923 Mb (Wang et al. 2020 - https://www.nature.com/articles/s41467-020-19682-0) and peak 356.73, and the peak of C. spinosa sub. spinosa var. herbacea is 123.27, following the formula "peak (ref)/genome size (ref) = peak (Capparis spinosa)/genome size (Capparis spinosa)" the value is 318,94 Mb, therefore the coverage obtained and genome completeness is nearly 77% (and not the 99.98%). Please explain and/or modify all sentences and statistics related to this point.
- Regarding the transcriptomic data, why in the previous version this data was not included? Regarding the material, usually the tissues are sequenced both separately and mixed, and the mix is developed without leaves, because this tissue shows the more representative genes, therefore the genes with low expression in other tissues could be missed. Please explain the choice.
The authors improved the annotation using the RNASeq data, but it is strange that the genes identified is lower (in number) than the blast obtained from the other approaches used in previous version. Explain and/or check. It is also singular that using the RNASeq data for the annotation, the results showed in this version (e.g. Figure 4) are the same of previous version, in term of Ks, ortholog genes etc. Check please.

- Finally the discussion must to be improved. In particular the authors have to better compare their results to other genomes available and they have to add other information about the genes isolated, e.g. in the present version there is only one section for terpenoids (three lines) and HSP and nothing else…did the authors find only these genes of interest? Also the evolutionary history is weak, please improve this section.

Level of Interest

Please indicate how interesting you found the manuscript: Choose an item.

Quality of Written English

Please indicate the quality of language in the manuscript: Choose an item.

Declaration of Competing Interests

Please complete a declaration of competing interests, considering the following questions:

- Have you in the past five years received reimbursements, fees, funding, or salary from an organisation that may in any way gain or lose financially from the publication of this manuscript, either now or in the future?
- Do you hold any stocks or shares in an organisation that may in any way gain or lose financially from the publication of this manuscript, either now or in the future?
- Do you hold or are you currently applying for any patents relating to the content of the manuscript?
- Have you received reimbursements, fees, funding, or salary from an organization that holds or has applied for patents relating to the content of the manuscript?
- Do you have any other financial competing interests?
- Do you have any non-financial competing interests in relation to this paper?

If you can answer no to all of the above, write 'I declare that I have no competing interests' below. If your reply is yes to any, please give details below.

I declare that I have no competing interests

I agree to the open peer review policy of the journal. I understand that my name will be included on my report to the authors and, if the manuscript is accepted for publication, my named report including any attachments I upload will be posted on the website along with the authors' responses. I agree for my report to be made available under an Open Access Creative Commons CC-BY license (http://creativecommons.org/licenses/by/4.0/). I understand that any comments which I do not wish to be included in my named report can be included as confidential comments to the editors, which will not be published.

Choose an item.

To further support our reviewers, we have joined with Publons, where you can gain additional credit to further highlight your hard work (see: https://publons.com/journal/530/gigascience). On publication of this paper, your review will be automatically added to Publons, you can then choose whether or not to claim your Publons credit. I understand this statement.

Yes Choose an item.
