# Supplementary material for: The Capparis spinosa var. herbacea genome provides the first genomic instrument for a diversity and evolution study of the Capparaceae family
Source: Gigascience. 2022 Oct 30;11:giac106. doi: 10.1093/gigascience/giac106 (PMC9618406; doi:10.1093/gigascience/giac106)
Supplement: giac106_Reviewer_1_Report_Revision_2 — Francesco Mercati -- 8/22/2022 Reviewed [file giac106_reviewer_1_report_revision_2.pdf]

**Reviewer Report**

**Title: T he Capparis spinosa var. herbacea genome provides the first genomic instrument for diversity and evolution study of Capparaceae family**

**Version: Revision 2** **Date:** 8/22/2022

**Reviewer name: Francesco Mercati**

**Reviewer Comments to Author:**

The authors improved the paper following the suggestions provided in the last revision. However there are still some lacks.
The authors explained how the genome size estimation has been carried out, using the contigs assembly from Wang et al. 2020 (807.6 Mb). I am not completely agree, but the choice can be accepted.
The description about annotation was unclear in the previous version and, probably, with some missing data. Now the authors used RNASeq data for the genes prediction (in my opinion new data, because all information about this point - methods, results, discussion - were previously missed in all sections). Unfortunately the section reported for genes prediction and annotation ("Genome coding gene prediction and annotation") are still poor. The authors have to add some additional information, that can be used also in the discussion.
In addition, I suggest to the authors to change the title. I am not agree about the possibility that the paper is able to "provides insight into Capparaceae genome evolution". The authors describe the first genome in the C. spinosa group without strong comparison in its and/or sister families (due to also the lack of information available). Therefore, although this study is an interest beginning point, that provides new information for future studies, the title is pretentious. The authors need to change it in (e.g.) "The Capparis spinosa var. herbacea genome provides the first genomic instrument for diversity and evolution study of Capparaceae family"…or something similar.
The authors have to improve the Background section, adding some information about the distribution of C. spinosa var herbacea in the world, in order to justify why the authors have chosen this variety.
In the "Analyses of genome synteny and WGD" (line 161-172), using the species compared the results obtained is obvious ("The syntenic analysis results also showed more collinear gene pairs between C. spinosa var. herbacea and A. thaliana (Table S7), indicating that C. spinosa var. herbacea has a close evolutionary relationship with A. thaliana"). In my opinion to have more information the authors have to add in the same analysis also T. hassleriana (in this version it is used only for LTR insertion evaluation) and Gynandropsis gynandra, both belonging to Cleomaceae. The last one should be also added at the gene family expansion and contraction evaluation and in the phylogenetic analysis and species divergence time estimation.
Finally, in the "Data availability" all links included in this version don't work. In addition the sequences obtained from transcriptome (both long and short reads) has not been deposited and/or the link has not been reported. The authors must to provide all information/data/results developed (this is mandatory).

**Level of Interest**

Please indicate how interesting you found the manuscript: Choose an item.

**Quality of Written English**

Please indicate the quality of language in the manuscript: Choose an item.

**Declaration of Competing Interests**

Please complete a declaration of competing interests, considering the following questions:

- Have you in the past five years received reimbursements, fees, funding, or salary from an organisation that may in any way gain or lose financially from the publication of this manuscript, either now or in the future?
- Do you hold any stocks or shares in an organisation that may in any way gain or lose financially from the publication of this manuscript, either now or in the future?
- Do you hold or are you currently applying for any patents relating to the content of the manuscript?
- Have you received reimbursements, fees, funding, or salary from an organization that holds or has applied for patents relating to the content of the manuscript?
- Do you have any other financial competing interests?
- Do you have any non-financial competing interests in relation to this paper?

If you can answer no to all of the above, write 'I declare that I have no competing interests' below. If your reply is yes to any, please give details below.

I declare that I have no competing interests

I agree to the open peer review policy of the journal. I understand that my name will be included on my report to the authors and, if the manuscript is accepted for publication, my named report including any attachments I upload will be posted on the website along with the authors' responses. I agree for my report to be made available under an Open Access Creative Commons CC-BY license (http://creativecommons.org/licenses/by/4.0/). I understand that any comments which I do not wish to be included in my named report can be included as confidential comments to the editors, which will not be published.

Choose an item.

To further support our reviewers, we have joined with Publons, where you can gain additional credit to further highlight your hard work (see: https://publons.com/journal/530/gigascience). On publication of this paper, your review will be automatically added to Publons, you can then choose whether or not to claim your Publons credit. I understand this statement.

Yes Choose an item.
