# Supplementary material for: The Capparis spinosa var. herbacea genome provides the first genomic instrument for a diversity and evolution study of the Capparaceae family
Source: Gigascience. 2022 Oct 30;11:giac106. doi: 10.1093/gigascience/giac106 (PMC9618406; doi:10.1093/gigascience/giac106)
Supplement: giac106_Reviewer_2_Report_Original_Submission — Eric Schranz -- 4/29/2022 Reviewed [file giac106_reviewer_2_report_original_submission.pdf]

Reviewer Report

Title: T he Capparis spinosa var. herbacea genome provides the first genomic instrument for diversity and evolution study of Capparaceae family

Version: Original Submission Date: 4/29/2022

Reviewer name: Eric Schranz

Reviewer Comments to Author:

I am extremely excited and pleased to finally see a Capparaceae genome assembly of high quality. Overall, I find this a straightforward report of a genome sequencing project with a few nuggets of interesting biology included (such as the high rate of Tandem repeats and their negative correlations with GC content). However, I have a few major concerns/questions for the authors as well as a few minor suggestions for improvement.

Major questions:

1. There is almost no discussion about the past domestication history of Capparis spinosa. If this was done, then likely the authors would report that C. spinosa is thought to be of hybrid origin between two between C. orientalis and C. sicula (e.g. Rivera et al., 2002). The authors present no information on the degree of potential heterozygosity in their assembly and if for example som time of purge haplotypes steps was done. I would very much like to see some discussion about domestication and thus how past hybridisation was dealt with (this should be much more recent than the reported WGD).
2. The positioning of the WGD and the dating of divergence times is a bit confusing. There is quite some past work on the dating of say the At-alpha WGD in Brassicaceae and also the divergence of Brassicaceae from Cleomaceae and Capparaceae (e.g. see work for example of Jocelyn Hall). Also, placing the capparaceae duplication in light of Cleomaceae duplications (e.g. paper by Marbry et al 2021). Also, the "younger age" maybe due to Capparis being woody and most other compared species are herbaceous (except Grape). This is issue is known to cause dating problems and is well discussed in the literature.
3. Ideally the seed material or at a minimum a herbarium sample of the genotype used for sequencing should be made clear. Can others access this material in some way? Also, having a line from a botanic garden means that plant came from somewhere else (e.g. in the Mediterranean basin). Where is this plant originally from? Also, there is a chance (see point 1) that this plant is NOT C. spinosa but another non-hybrid Capparis. Please have a skilled taxonomist check the plant and deposit a herbarium specimen.

Minor suggestions:

1. Do not use italics with family name: Capparaceae
2. When starting a sentence, don't say C. spinosa...then Capparis spinosa (e..g. line 50)
3. line 68: Change "Total..." to "A total..."
4. Line 114...this line "Gene footprints in the duplicate events were observed. " Is confusing to me (footprints in sequences often mean something else)...I suggest you just delete it.

5. You mention some chemicals used/present in Capparis, but never glucosinolates! This is the character that gives capers their flavour and make them clearly part of Brassicales.

Level of Interest

Please indicate how interesting you found the manuscript: Choose an item.

Quality of Written English

Please indicate the quality of language in the manuscript: Choose an item.

Declaration of Competing Interests

Please complete a declaration of competing interests, considering the following questions:

- Have you in the past five years received reimbursements, fees, funding, or salary from an organisation that may in any way gain or lose financially from the publication of this manuscript, either now or in the future?
- Do you hold any stocks or shares in an organisation that may in any way gain or lose financially from the publication of this manuscript, either now or in the future?
- Do you hold or are you currently applying for any patents relating to the content of the manuscript?
- Have you received reimbursements, fees, funding, or salary from an organization that holds or has applied for patents relating to the content of the manuscript?
- Do you have any other financial competing interests?
- Do you have any non-financial competing interests in relation to this paper?

If you can answer no to all of the above, write 'I declare that I have no competing interests' below. If your reply is yes to any, please give details below.

'I declare that I have no competing interests'

I agree to the open peer review policy of the journal. I understand that my name will be included on my report to the authors and, if the manuscript is accepted for publication, my named report including any attachments I upload will be posted on the website along with the authors' responses. I agree for my report to be made available under an Open Access Creative Commons CC-BY license (http://creativecommons.org/licenses/by/4.0/). I understand that any comments which I do not wish to be included in my named report can be included as confidential comments to the editors, which will not be published.

Choose an item.

To further support our reviewers, we have joined with Publons, where you can gain additional credit to further highlight your hard work (see: https://publons.com/journal/530/gigascience). On publication of this paper, your review will be automatically added to Publons, you can then choose whether or not to claim your Publons credit. I understand this statement.

Yes Choose an item.
