# Supplementary material for: The Capparis spinosa var. herbacea genome provides the first genomic instrument for a diversity and evolution study of the Capparaceae family
Source: Gigascience. 2022 Oct 30;11:giac106. doi: 10.1093/gigascience/giac106 (PMC9618406; doi:10.1093/gigascience/giac106)
Supplement: giac106_Reviewer_2_Report_Revision_1 — Eric Schranz -- 7/12/2022 Reviewed [file giac106_reviewer_2_report_revision_1.pdf]

Reviewer Report

Title: T he Capparis spinosa var. herbacea genome provides the first genomic instrument for diversity and evolution study of Capparaceae family

Version: Revision 1 **Date:** 7/12/2022

Reviewer name: Eric Schranz

Reviewer Comments to Author:

As reviewer #2 from the first round, I am not at all satisfied with the efforts of the authors to improve the manuscript based on my suggestions last time. It is not enough to simply write rebuttal pararaphs in the response to the reviewers, I would have expected and wanted actual changes to the manuscript. Again, I do not think the actual subspecies sequenced or info about domestication are sufficiently covered in the actual manuscript. There needs to be more info given and explained.
Also, the question of if there is a family Capparaceae or not is not controversial or uncertain. It is now accepted by the Angiosperm Phylogeny Group and therefore has to be adopted in this paper (I strongly believe). Also, data is given in paper for Cleome spinosa, but after taxonomic revisions this is now known to be Tarenaya hasseleriana...therefore this needs to be updated as well.
Similarly, it is not enough to tell me the reviewer that a herbarium specimen exists and that I can write the botanic garden...It must be clearly stated in the paper and include exactly where the herbarium specimen can be found (e.g. what is the herbarium and accession sheet).
Please actually make appropriate revisions to the manuscript rather than just write to me the reviewer in the rebuttal!

Level of Interest

Please indicate how interesting you found the manuscript: Choose an item.

Quality of Written English

Please indicate the quality of language in the manuscript: Choose an item.

Declaration of Competing Interests

Please complete a declaration of competing interests, considering the following questions:

- Have you in the past five years received reimbursements, fees, funding, or salary from an organisation that may in any way gain or lose financially from the publication of this manuscript, either now or in the future?
- Do you hold any stocks or shares in an organisation that may in any way gain or lose financially from the publication of this manuscript, either now or in the future?

- Do you hold or are you currently applying for any patents relating to the content of the manuscript?
- Have you received reimbursements, fees, funding, or salary from an organization that holds or has applied for patents relating to the content of the manuscript?
- Do you have any other financial competing interests?
- Do you have any non-financial competing interests in relation to this paper?

If you can answer no to all of the above, write 'I declare that I have no competing interests' below. If your reply is yes to any, please give details below.

'I declare that I have no competing interests'

I agree to the open peer review policy of the journal. I understand that my name will be included on my report to the authors and, if the manuscript is accepted for publication, my named report including any attachments I upload will be posted on the website along with the authors' responses. I agree for my report to be made available under an Open Access Creative Commons CC-BY license (http://creativecommons.org/licenses/by/4.0/). I understand that any comments which I do not wish to be included in my named report can be included as confidential comments to the editors, which will not be published.

Choose an item.

To further support our reviewers, we have joined with Publons, where you can gain additional credit to further highlight your hard work (see: https://publons.com/journal/530/gigascience). On publication of this paper, your review will be automatically added to Publons, you can then choose whether or not to claim your Publons credit. I understand this statement.

Yes Choose an item.
